# Visible light-induced single bond rotation in azoarenes triggered by self-sensitized oxidation

**DOI:** 10.1039/d6sc06305e

**Published:** 2026-09-15

**Authors:** Zoe Nonie Scheller, Jan Schulte, Christoph Wölper, Gebhard Haberhauer

**Affiliations:** a Institut für Organische Chemie, Universität Duisburg-Essen, Universitätsstr. 7 D-45117 Essen Germany zoe.scheller@uni-due.de gebhard.haberhauer@uni-due.de

## Abstract

Controlled molecular motion is a cornerstone of both biological function and artificial dynamic systems. While light-induced isomerization of double bonds is well-established, selective rotation around single bonds without concurrent double bond isomerization remains largely unexplored. Here, we present a class of chalcogen-substituted azoarenes that undergo light-induced N–C single bond rotation, independent of N=N isomerization. This rotation is triggered by self-sensitized photooxidation of an *ortho*-tellurium center, which switches the preferred binding site of a β-hydroxy group from the azo unit to the tellurium σ hole. Experimental evidence from UV/Vis and NMR spectroscopy, supported by quantum chemical calculations, confirms the light-induced rotation and its complete reversibility upon chemical reduction. Importantly, the process is accessible under both UV and sunlight. This study establishes a new mechanism for directing molecular motion, offering a versatile strategy for designing photo- and redox-responsive molecular switches.

## Abstract 2

Light-induced oxidation of tellurium center in hydroxy azoarenes triggers selective N–C bond rotation. The reactivity of heavy chalcogens enables a new strategy for controlling molecular conformation through coupled photochemical and redox processes.

## Graphical abstract

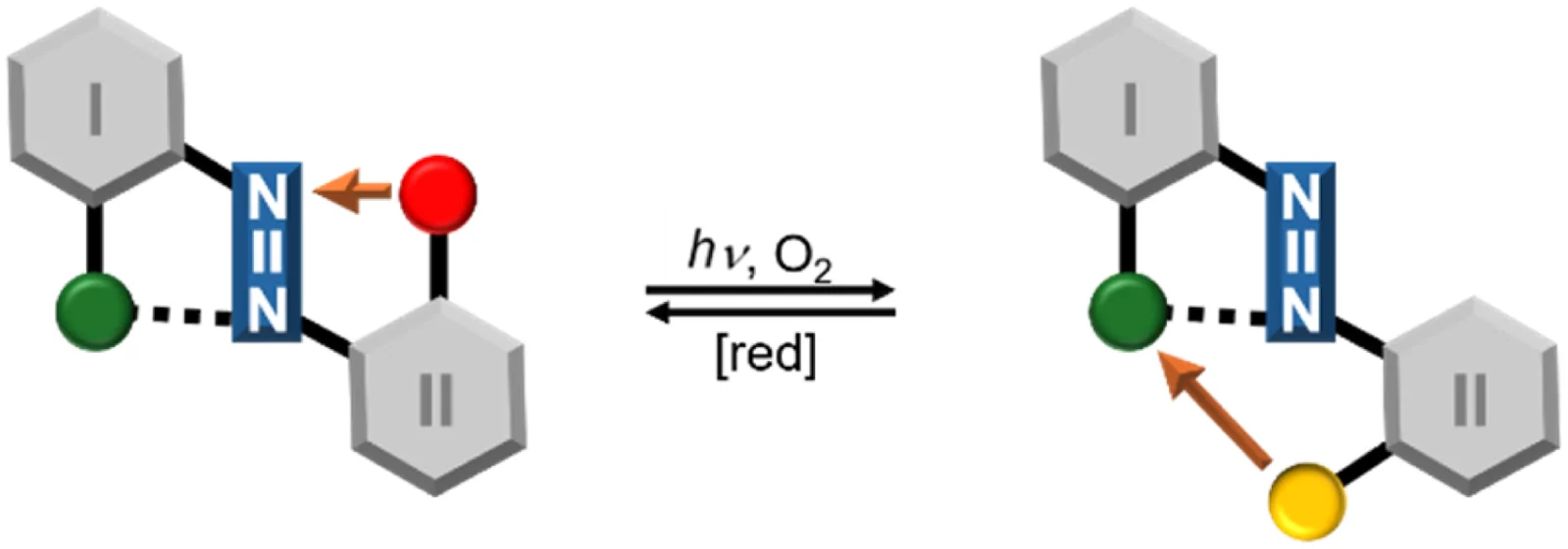

## 1 Introduction

Molecular motion shapes biological function and inspires the design of artificial dynamic systems.^1–3^ The development of molecules that can move or rearrange in a controlled manner in response to external stimuli offers unique opportunities for transferring energy and information at the molecular level.^4–6^ Introducing new classes of compounds capable of such motions expands the chemical toolbox for constructing responsive molecular systems.

**Fig. 1:**
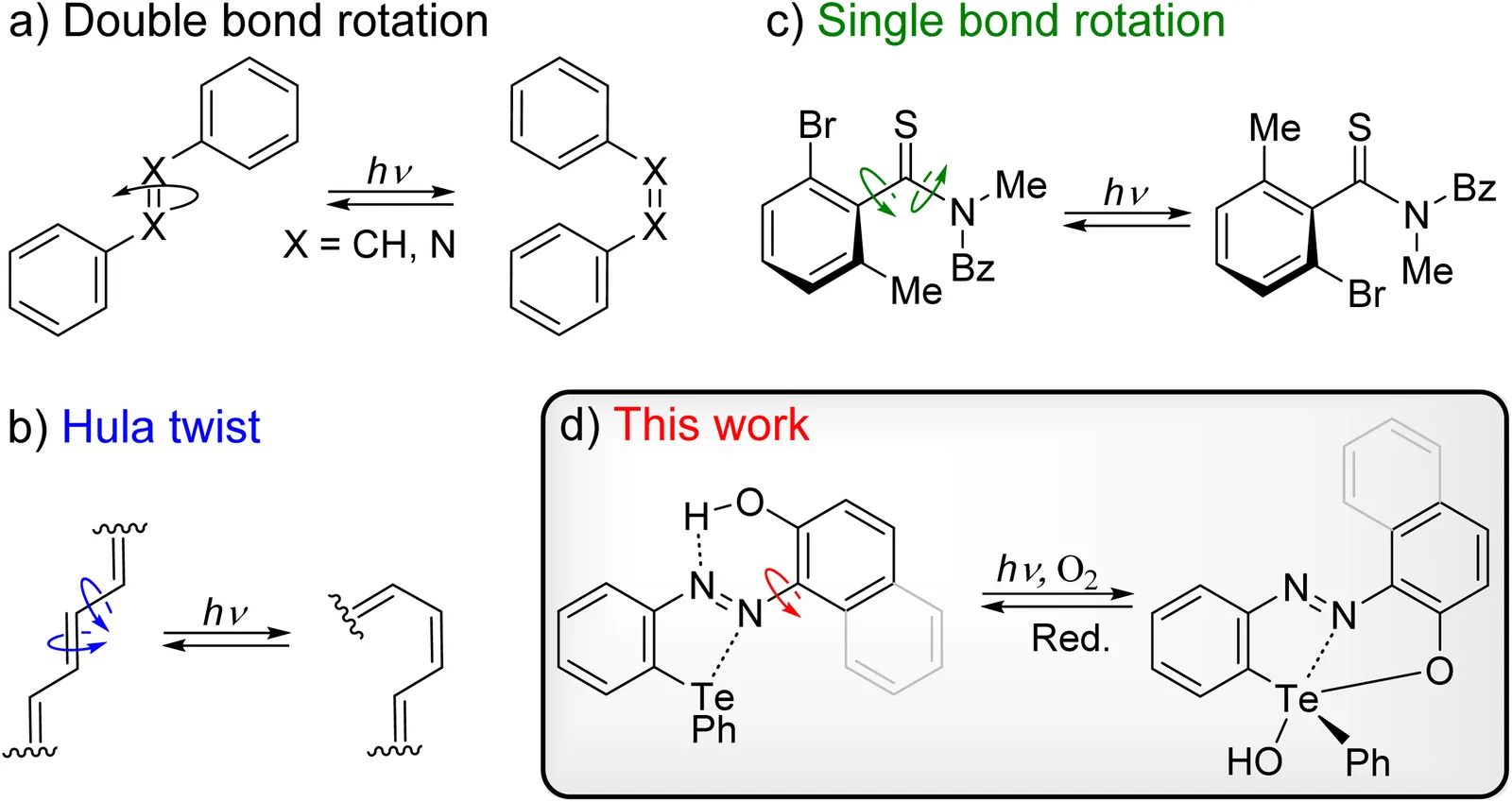
Selected examples of light-induced molecular motion involving double- (a and b) and single bond rotation (c and b) and comparison with this work (d).

A widely employed strategy to induce motion at the molecular scale is light-induced double bond isomerization.^3,7,8^ In most cases, an electron is promoted from a π to a π* orbital generating two unpaired electrons retaining the initial spin state. The π → π* excitation reduces the bond order of the π bond, thereby facilitating rotation around the bond. Such molecular motions are well established for the isomerization of C=C, C=N, and N=N double bonds (Fig. 1a).^8–13^ By contrast, the generation of a metastable state through rotation around a σ bond is generally not achieved *via* direct σ → σ* excitation, as this typically leads to bond cleavage and requires substantially higher energy. Nevertheless, light-induced rotation around a σ bond can occur when an adjacent π system is excited. However, this rotation is typically accompanied by the isomerization of the double bond. For example, N=N double bond systems have been reported in which rotation about the double bond triggers an additional single bond rotation.^1,14–17^ Such examples usually involve cyclic compounds where steric strain enforces geometric adjustments of remote single bonds. In contrast, a recently reported azoimidazolium molecular motor combines light-induced *trans*/*cis* photoisomerization with a subsequent thermally driven rotation about the adjacent C–N single bond, thereby achieving directional molecular rotation through a multistep reaction cycle.^18^ Furthermore, C=C double bonds can undergo a hula-twist motion,^19,20^ in which both the double bond and a directly adjacent single bond rotate simultaneously (Fig. 1b).^2,21^

More recently, single bond rotation has been observed in hemithioindigo (HTI) derivatives; however, these rotations are not selectively addressable and compete with rotation about the π bond as well as the hula twist.^22^ In a thioamide system (Fig. 1c), where any rotation about the C=S bond is not experimentally observable because the rotated structure is symmetry-equivalent to the initial state, concerted light-induced C–N/C–C rotation has been demonstrated.^23^

It is also worth noting that photoinduced motions around single bonds can arise through several distinct mechanisms. In some systems, a complete rotation around a σ bond occurs but does not lead to accumulation of a distinct photoproduct because symmetry renders the rotated state indistinguishable from the starting structure. For example, in TICT, PLICT, and PLATICT systems,^24–26^ excitation induces twisting and/or planarization toward charge-transfer states, which may involve either full bond rotation^27^ or merely increased torsion.^28^ These processes should be distinguished from photoswitches that generate metastable, isolable rotational isomers. A particularly illustrative example is a recently reported hemithioindigo photoswitch, in which four distinct photochemical pathways were identified within the same molecule, including single bond rotation, TICT-type twisting, hula-twist motion, and double bond isomerization.^29^ Controlled rotation about single bonds can also be achieved using stimuli other than light. In particular, redox-, acid/base-, and metal-coordination-responsive systems have established stimulus-controlled conformational switching and dynamic atropisomerism,^30–34^ while chemically fueled molecular motors and rotor systems exploit sequential reaction cycles to achieve directed molecular motion.^35–37^ Despite these advances, examples in which light directly generates an individually addressable, metastable rotational isomer through isolated single bond rotation remain exceedingly rare.

Here, we exploit the advantages of π → π* excitation of a double bond to design a molecular switch that operates *via* an isolated, light-induced single bond rotation without double bond isomerization (Fig. 1d). Azoarenes^7,9,11,38,39^ were chosen as photoresponsive systems due to their straightforward synthesis and functionalization, high switching reversibility, and pronounced photochemical stability, which allow numerous switching cycles. In addition, both the π → π* and n → π* transitions can be addressed, enabling visible-light-induced molecular motion.^7,40,41^ Our results show that irradiation triggers a self-sensitized photooxidation of the tellurium center, which in turn facilitates rotation about the N–C single bond directly adjacent to the N=N double bond. Evidence for this rotation is provided by UV and NMR experiments and supported by quantum chemical calculations. Moreover, we demonstrate that the quantitative light-induced switching is fully reversible upon reduction. The photooxidation-controlled bond rotation in chalcogen-substituted azoarenes represents a new concept for directing molecular motion.

## 2 Results and discussion

### 2.1 Concept

**Fig. 2:**
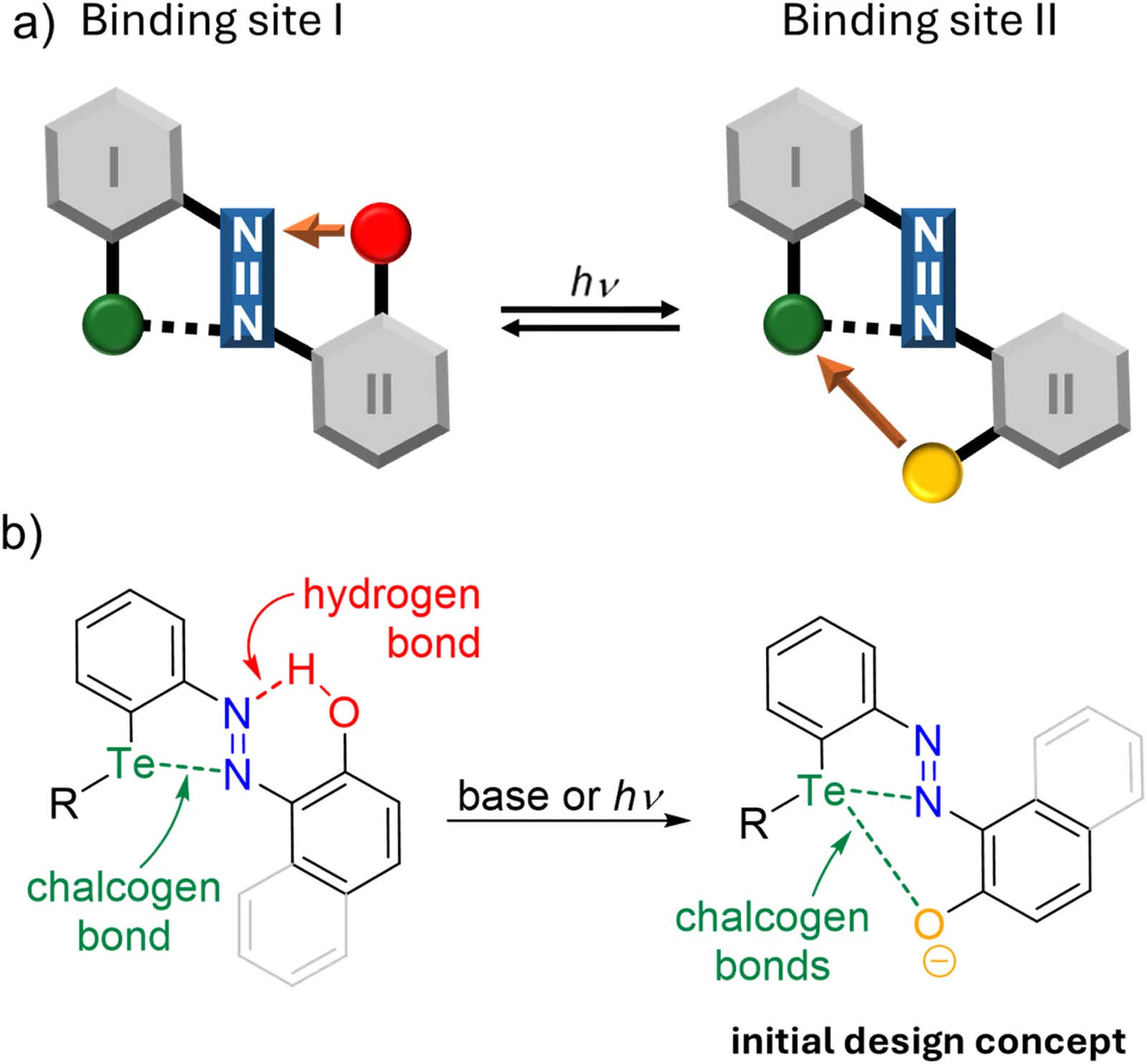
(a) Concept of single bond rotation about the N–C bond enabled by a light-induced change of the preferred binding site. (b) Initial design concept for single bond rotation of the N–C bond in β-hydroxyazoarene through (photo)deprotonation.

To induce light-triggered single bond rotation of the N–C bond without involving the azo unit, the system must meet specific requirements (Fig. 2). First, the *cis*/*trans* isomerization of the azo group must be suppressed, thereby preventing rotation about the N=N double bond. For this purpose, a substituent is introduced in the *ortho*-position of aryl ring I relative to the azo bridge, whose interaction with the azo unit fully suppresses photoisomerization (Fig. 2a, green). In addition, aryl ring II must bear a substituent that provides two potential binding sites within the molecule (Fig. 2a, red/yellow). The light-induced switching between these two binding sites is intended to trigger the rotation.

In the presented system, the two possible binding sites are, on the one hand, the azo unit itself and, on the other, a chalcogen center that can form a chalcogen bond with a Lewis base (Fig. 2b).^42–50^ In previous studies, we demonstrated that the photoisomerizability of azoarenes can be suppressed by introducing a tellurium substituent in the *ortho* position.^51–53^

Under irradiation, the chalcogen bond between the chalcogen atom and the azo nitrogen transforms to a three-electron two-center bond creating a minimum on the S_1_ energy profile from which no isomerization occurs.

As aryl unit II, we selected an *ortho*-hydroxy-substituted aromatic ring. Particularly in β-naphthol-based azoarenes, a hydrogen bond is formed with the azo nitrogen, promoting proton transfer across a six-membered ring and resulting in a tautomeric equilibrium between the azobenzene form (N=N; C–OH) and the hydrazone form (HN–N; C=O).^54,55^ Deprotonation of the compound is expected to shift the preferred binding site: the resulting phenolate can interact with the σ hole of the tellurium atom through the oxygen lone pair (Fig. 2b).^56–59^

This change in preferred binding site is accompanied by a rotation about the N–C single bond directly adjacent to the azo bridge, without direct involvement of the N=N bond. In the present case, we can exploit the photoacid properties of naphthols as an external stimulus. In such systems, the p*K*_a_ value decreases from 9.5 in the ground state to 0–2 in the excited state.^60,61^ As a result, the equilibrium between protonated and deprotonated forms shifts firmly toward the conjugate base in the excited state. Therefore, it should be possible to induce single bond rotation in the system shown in Fig. 2b through photodeprotonation.

### 2.2 Synthesis

**Fig. 3:**
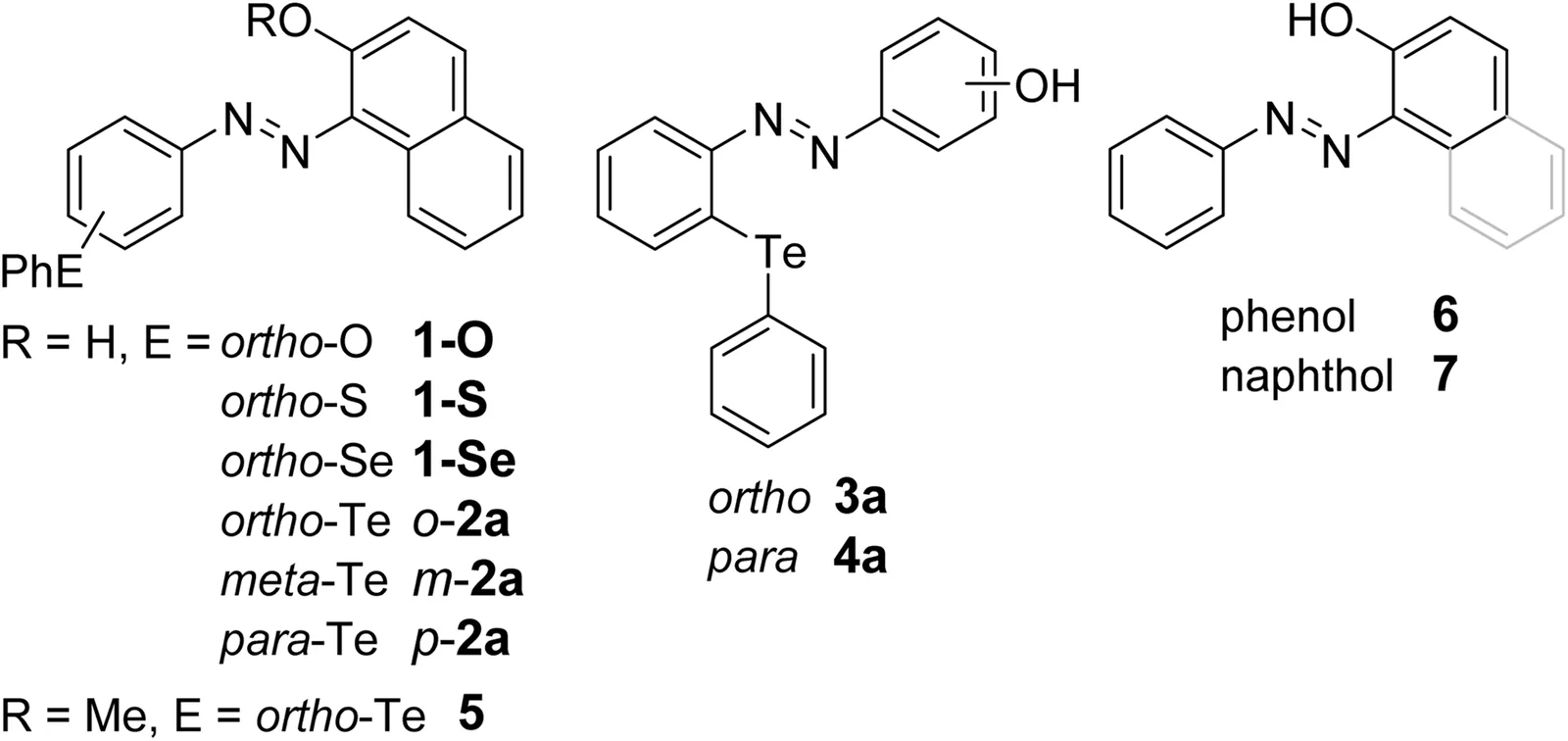
Overview of the investigated naphthol- and phenol-based azoarenes 1–7.

To explore the potential of an atypical azo switch operating through single bond rotation, we examined a series of chalcogen-containing naphthol- and phenol-based azoarenes (1–7). We selected systems that meet both criteria for single bond rotation (*o*-2a and 3a, Fig. 3). These compounds feature a tellurium center in the *ortho* position, which suppresses rotation around the N=N bond and provides an additional binding site. In addition, these compounds contain an *ortho*-hydroxy group, whose preferred binding site is expected to change with pH. We included both naphthol- and phenol-based systems because they differ in their tendency toward tautomerization. Furthermore, we included compounds that contain a tellurium center but contain either a *para*-hydroxy (4a) or a protected hydroxy group (5, Fig. 3). To assess the influence of the chalcogen on the photoswitching behavior, we also investigated systems bearing *ortho*-hydroxy substituents but featuring other chalcogens (E = O, S, Se; 1) or lacking a chalcogen entirely (6 and 7). Finally, we varied the position of the TePh group (*m*/*p*-2a) to probe positional effects on photoresponsiveness.

**Scheme 1:**
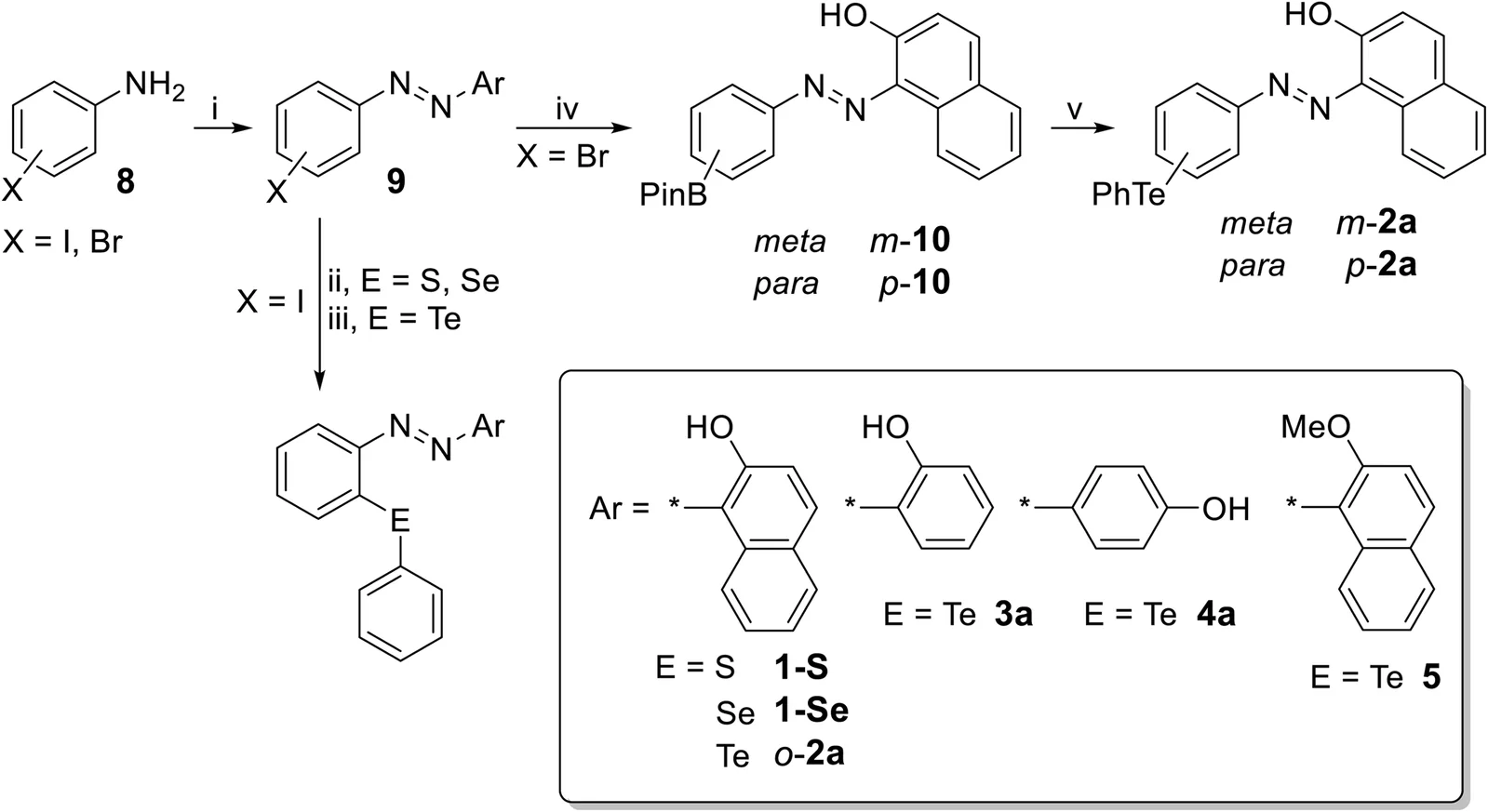
Synthesis of the azoarenes 1–5. Reaction conditions: (i) NaNO_2_, HCl, AcOH; (ii) KO*t*-Bu, Ph_2_E_2_ (E = S, Se), dimethyl sulfoxide; (iii) *n*-BuLi, Ph_2_Te_2_, diethyl ether; (iv) (BPin)_2_, Pd(dppf)Cl_2_, KOAc, dioxane; (v) Ph_2_Te_2_, CuI, 2,2′-bipyridine, dimethyl sulfoxide/water.

The *ortho*-chalcogen substituted azoarenes were prepared according to a literature-known sequence.^51,53^ The aniline 8 was reacted with the desired naphthol/phenol derivative to give the halogenated azoarene 9 (Scheme 1). In cases 3a and 5, an additional step of functional group interconversion was required (see SI). For the sulfur- and selenium-substituted azoarenes 1-S and 1-Se, compound 9 was reacted with the corresponding diphenyl dichalcogenide (E = S, Se) and potassium *tert*-butanolate.^62^ The *ortho*-tellurium-substituted compounds were prepared by lithium-halogen exchange with *n*-BuLi and addition of diphenyl ditelluride.^51^ The azoarenes *m*/*p*-2a were synthesized by reacting the corresponding bromoaniline with 2-naphthol. The product of the conversion of 9 in a Miyaura borylation *m*/*p*-10 is then used in a copper-catalyzed reaction with diphenyl ditelluride to form the *meta*- and *para*-substituted 2a. Compounds 1-O, 6, and 7 were synthesized according to procedures reported in the literature.^63,64^

### 2.3 Tautomeric equilibrium

Since the degree of azo-hydrazone tautomerism directly determines the bond order of the N–C bond adjacent to the azo unit, we first analyzed the tautomeric equilibrium of the investigated azoarenes. The carbonyl carbon chemical shift (*δ*_CO_) is well established as a sensitive probe for the azo-hydrazone equilibrium of β-hydroxy azoarenes and was therefore used to estimate the tautomeric composition of the naphthol derivatives (Equation S1, Table S1).^65,66^ Comparison of compounds 1 and *o*-2a reveals a pronounced dependence on the nature of the chalcogen atom. Whereas the oxygen derivative 1-O predominantly exists as the hydrazone tautomer (*K*_CDCl_3__ = 7.7), the hydrazone contribution decreases with increasing chalcogen size, reaching an azo preference for *o*-2a (*K*_CDCl_3__ = 0.7). The experimentally determined tautomer ratios correlate well with the crystallographic bond lengths obtained for 1-S and *o*-2a (Fig. S54), supporting the validity of the NMR-based analysis. Comparison of the three tellurium regioisomers 2a further demonstrates that the intramolecular Te⋯N chalcogen bond significantly shifts the equilibrium toward the azobenzene tautomer. While the *meta*- and *para*-substituted derivatives exhibit *K*_MeOD_ = 2.8 (*m*-2a) and *K*_MeOD_ = 3.0 (*p*-2a), respectively, the corresponding *ortho* derivative *o*-2a displays a substantially lower value of *K*_MeOD_ = 0.6. This observation indicates that the intramolecular Te⋯N interaction stabilizes the azobenzene tautomer in solution. As expected, the methoxy-protected derivative 5 exhibits no tautomerism and exists, as well as the phenol derivatives 3a and 4a, exclusively as the azobenzene tautomer. For the phenol compounds, formation of the corresponding hydrazone would require complete loss of the aromaticity of the phenyl ring and is therefore energetically highly unfavorable.^55^ Consequently, whereas *o*-2a occupies an intermediate regime in which the N–C bond exhibits partial double bond character owing to azo-hydrazone tautomerism, the corresponding phenol derivative 3a contains a genuine N–C single bond. As discussed below, both systems nevertheless display the same oxygen-dependent photooxidation-induced conformational switching despite their different tautomeric preferences.

### 2.4 Irradiation experiments

**Fig. 4:**
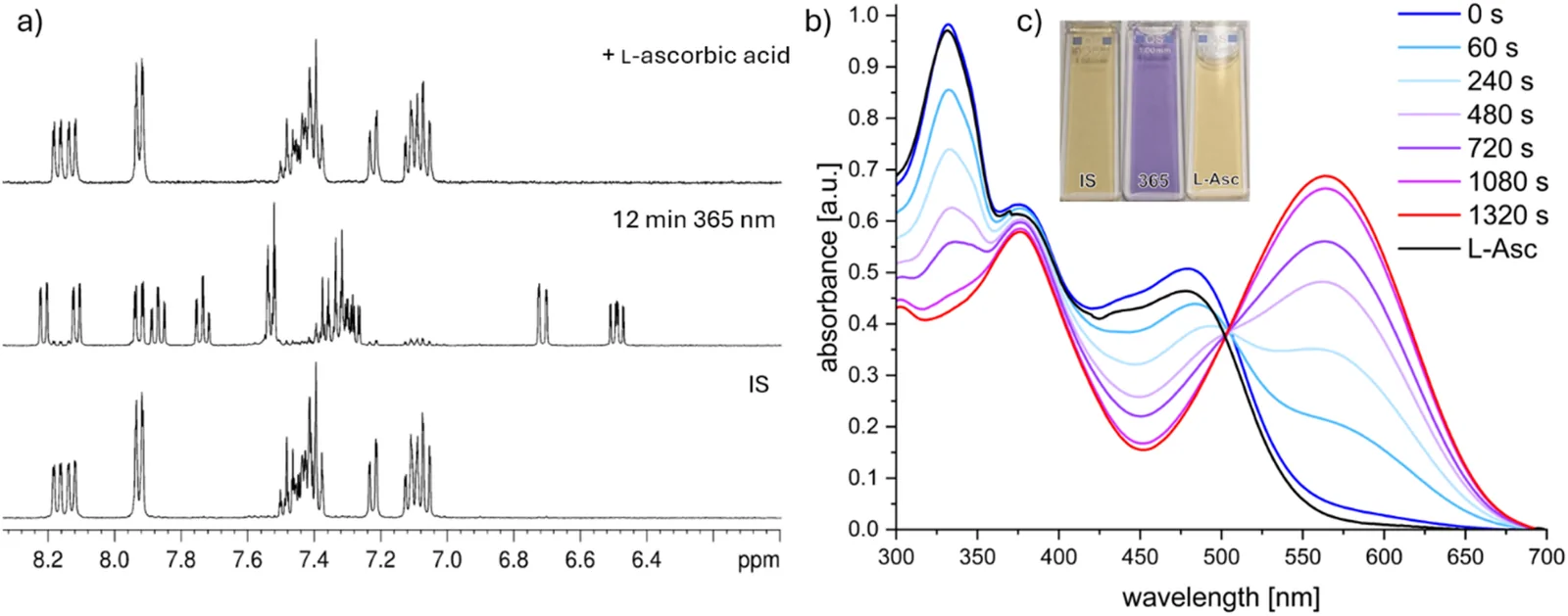
(a) Section of the ^1^H NMR spectra of the azobenzene 3a after synthesis (IS, initial state), after 12 minutes of UV light irradiation with *λ* = 365 nm and after addition of l-ascorbic acid to the irradiated sample (MeOD, 400 MHz, *c* = 5 mm). (b) Normalized UV/Vis spectra of the azobenzene 3a: after synthesis (0 s, blue), after UV irradiation with *λ* = 365 nm after different irradiation periods and after addition of l-ascorbic acid to the irradiated sample (MeOH, *c* = 1.0 mm). (c) Picture of the UV/Vis cuvettes (*d* = 1 mm) before and after irradiation with *λ* = 365 nm as well as after addition of l-ascorbic acid to the irradiated sample.

Below, we studied the photoresponsiveness of compounds 1–7. The azoarenes were dissolved in methanol and analyzed by UV/Vis spectroscopy (*c* = 1 mm, Fig. S1–S11) and NMR spectroscopy (*c* = 5 mm, Fig. S17–S22). An initial spectrum of each compound was recorded, followed by irradiation at *λ* = 365 nm for the specified period (see SI), and then another spectrum was recorded. Irradiation was continued until no further spectral changes were observed. Notably, naphthol *o*-2a and the phenol derivative 3a exhibit particularly characteristic switching behavior upon irradiation. Fig. 4a shows the NMR experiment of compound 3a as an example. With increasing irradiation time, a selective conversion of the starting material into a new species is observed. This is evident from the decrease of the signals from 7.0 to 7.2 ppm and the appearance of new signals at 6.5 and 6.7 ppm. Complete conversion is achieved after 12 minutes of UV irradiation. Fig. 4b shows the UV/Vis spectrum of compound 3a. With increasing irradiation time, the absorption maximum shifts from 470 to 570 nm, accompanied by a visible color change from yellowish to violet (Fig. 4c). The presence of a well-defined isosbestic point is consistent with a selective conversion between two dominant species. In this experimental setup, the complete conversion (Fig. 4b, red) is observed after approximately 20 minutes. An analogous behavior under UV irradiation is detected for compound *o*-2a (Fig. S4 and S24a–c). However, prolonged irradiation results in a decrease in UV absorbance (Fig. S4), indicating slight product decomposition, as evidenced by NMR signals at 7.4–6.5 ppm (Fig. S24c). All assignments of the observed species throughout this study are based on the combined analysis of UV/Vis, ^1^H and ^125^Te NMR, HRMS, and complementary control experiments, including chemical reduction and thermal relaxation, allowing photooxidation products to be distinguished from irreversible degradation products. The stepwise conversion of *o*-2a and 3a cannot be explained by light-induced deprotonation, as shown by a control experiment. When *o*-2a is deprotonated by the addition of two equivalents of KO*t*-Bu, the resulting ^1^H NMR spectrum (Fig. S33b) does not match the spectrum obtained after UV irradiation of *o*-2a (Fig. S24c). Therefore, the observed process is not a simple photodeprotonation of the phenol (3a) or naphthol unit (*o*-2a). Notably, irradiation of the deprotonated form of *o*-2a yields the same product as irradiation of the protonated species (Fig. S24c and S33c). Subsequent irradiation of *o*-2a with *λ* = 405 nm and *λ* = 530 nm shows no regeneration of the initial compound (Fig. S23). Furthermore, since no back-isomerization occurs even after several days of storage, the photoproduct *o*-2b does not correspond to the *cis* form of this azobenzene.

**Scheme 2:**
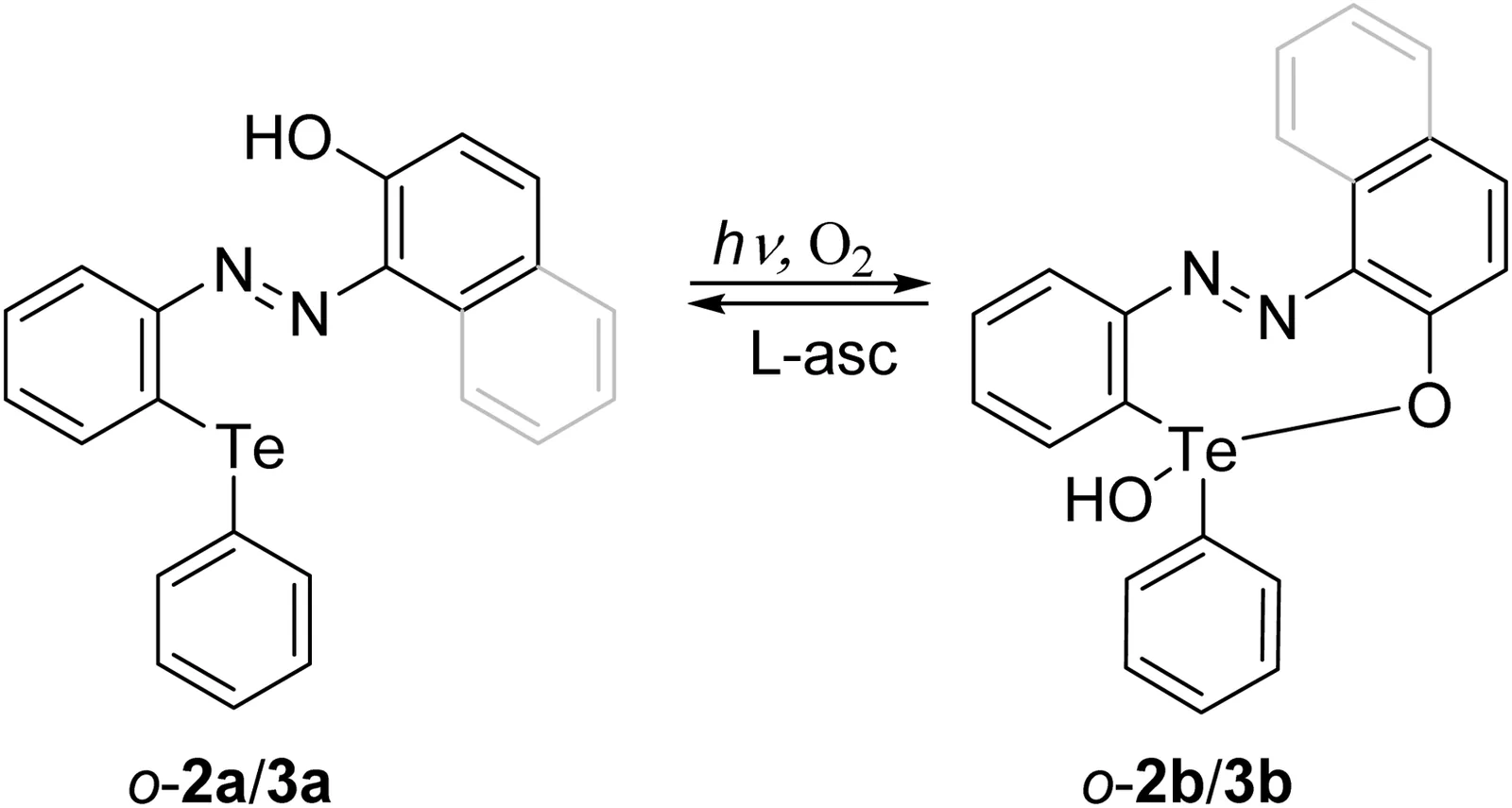
Photooxidation-induced conformational switching of *o*-2a/3a to *o*-2b/3b and regeneration of the starting compounds by reduction with l-ascorbic acid. The grey structural fragment is present in the naphthol derivatives *o*-2a and *o*-2b and absent in the phenol derivatives 3a and 3b. The photoproducts are shown in the structural formulation most consistent with the combined spectroscopic and computational evidence (*vide infra*).

To elucidate the origin of the long-lived photoproduct, the irradiation experiment was repeated under oxygen-free conditions. Under these conditions, neither photooxidation nor detectable *trans* → *cis* photoisomerization was observed (Fig. S25). The only additional signals observed (≈10%) are attributable to a degradation product, as evidenced by the benzene resonance at 7.33 ppm, arising from cleavage of the Te–Ph bond. Although an ultrafast *trans* → *cis* → *trans* cycle cannot be completely excluded, molecular oxygen is clearly indispensable for generating the long-lived switched state. These findings strongly indicate that the observed transformation is initiated by self-sensitized photooxidation of the tellurium center, a mechanism previously described for several tellurium-containing cationic dyes such as pyrylium and tellurorhodamines.^67–70^ The proposed photooxidation-induced transformations of *o*-2a and 3a and the corresponding reductive regeneration processes are summarized in Scheme 2.

To obtain further insight into the nature of the photoproduct, the irradiated solutions were characterized by ^1^H, ^13^C, and ^125^Te NMR spectroscopy as well as HRMS (see SI). Irradiation experiments were additionally performed in other solvents (Fig. S41–S44), revealing a pronounced solvent dependence of the photooxidation. While considerably less selective transformations were observed in DMSO and MeCN, irradiation of *o*-2a in CDCl_3_ represents an exception and proceeds with comparatively high selectivity. However, pronounced spectral differences between the products observed in different solvents indicate solvent-dependent solution-state speciation. Since the photoproduct formed in CDCl_3_ therefore cannot be assumed to represent the same solution-state species as that generated in methanol, the detailed structural characterization discussed below focuses on the photoproduct generated in methanol. HRMS reveals two molecular-ion peaks corresponding to the molecular formulas [C_22_H_15_N_2_OTe]^+^ ([*o*-2a − H]^+^) and [C_22_H_17_N_2_O_2_Te]^+^ ([*o*-2a + OH]^+^), confirming the incorporation of an additional oxygen atom. In addition, the comparison of the ^125^Te NMR chemical shifts of *o*-2a (661 ppm), the photoproduct *o*-2b (1214 ppm), and the deprotonated species (674 ppm, Fig. S26) shows a pronounced deshielding of the tellurium center in *o*-2b. This NMR shift aligns with literature-reported values for Te(iv) derivatives bearing strongly electron-withdrawing substituents, such as oxygen.^71^ Together, the HRMS and ^125^Te NMR data establish the formation of an oxygen-containing Te(iv) photoproduct. However, these data do not independently establish the exact protonation and bonding state of the oxygen-containing Te(iv) species.

Additional experiments further demonstrate that the photoproduct *o*-2b can be fully and essentially instantaneously reconverted to the starting material *o*-2a by the addition of the reducing agent l-ascorbic acid (Fig. S24d). The sensitivity of Te(iv) derivatives to reduction by l-ascorbic acid is documented in the literature.^51^ To investigate whether the reverse transformation upon addition of l-ascorbic acid could be attributed to acidification rather than reduction, citric acid was used as an acidic non-ascorbate control (Fig. S34). Although the addition of citric acid induces pronounced spectral changes, no regeneration of the starting material was observed. This control therefore indicates that acidification alone is insufficient to induce the reverse transformation and supports the assignment of reduction as the relevant function of l-ascorbic acid under these conditions. These findings support the conclusion that the photoreaction proceeds *via* a self-sensitized photooxidation.

In addition to the naphthol *o*-2a and phenol derivative 3a, the compounds *m*-2a, *p*-2a and 4a (Fig. 3) also lack *cis*/*trans* isomerization upon irradiation with light of the wavelength *λ* = 365 nm (Fig. S5–S7 and S18–S20), but they similarly undergo photooxidation. For β-naphthols such as *m*-2a and *p*-2a, where an *ortho*-tellurium substituent does not suppress photoswitching, the existing tautomeric equilibrium between the azobenzene and hydrazone forms (Table S1) enables rapid thermal back-isomerization through rotation around the N–N single bond. As a result, a change of the *cis*/*trans* ratio in comparable systems is only observed at temperatures below −80 °C.^54,55^ The photooxidation observed in *m*-2a, *p*-2a and 4a is considerably less selective than for the *ortho*-substituted compounds *o*-2a and 3a, resulting in substantially greater decomposition and the formation of byproducts (Fig. S5 and S6). Additionally, the characteristic color change (Fig. 4c and S4) is absent for *m*-2a and *p*-2a, indicating that the *ortho*-tellurium center induces this effect.

The chalcogen-free compounds 6 and 7, as well as the naphthol derivatives 1 with lighter homologs (O, S, Se) in the *ortho* position, show no photochemical oxidation (Fig. S1–S3, S10, S11 and S17). Please note: the absence of detectable oxidation products for 1 does not necessarily imply that these compounds are unable to generate reactive oxygen species (ROS) upon irradiation. The observed differences may alternatively arise from the different susceptibility of the chalcogen centers toward oxidation. In this context, oxidation of organotellurium compounds is generally more facile than that of the corresponding lighter chalcogen homologues.^72,73^ Interestingly, compounds 1-O, 1-S, and 1-Se exhibit weak solid-state fluorescence with absolute photoluminescence quantum yields of 1–4% (Fig. S45). While azobenzene derivatives are generally weakly emissive in solution due to efficient non-radiative decay pathways,^74–76^ suppression of molecular motion in the solid or aggregated state can enable detectable emission.^77,78^ The methoxy-protected derivative 5 also lacks oxidation under irradiation, but a minor photoisomerization to the *cis* isomer (<10%) is observed (Fig. S9 and S22). This behavior is consistent with the destabilization of the three-electron two-center bond between the chalcogen center and the azo nitrogen in the excited-state when electron-rich aryl groups are used, which leads to partial recovery of photoswitchability.^53^ A hydroxy group in combination with a tellurium center is therefore essential for enabling self-sensitized photooxidation, irrespective of the substitution pattern. This explains the absence of photooxidation in previously studied *ortho*-Te-substituted azoarenes.^51–53^

Performing the photooxidation in the presence of the radical scavenger TEMPO ((2,2,6,6-tetramethylpiperidin-1-yl)oxyl) significantly slows the process, suggesting the involvement of a radical species (Fig. S32). Based on the oxygen dependence of the reaction and the inhibition observed in the presence of TEMPO, the transformation most likely proceeds *via* reactive oxygen species (ROS) generated during photoirradiation. Although the present data do not allow differentiation between individual ROS, a plausible mechanism involves the formation of a naphthoxyl radical, which can activate oxygen to the superoxide radical anion.^79^

### 2.5 Evidence of single bond rotation

**Fig. 5:**
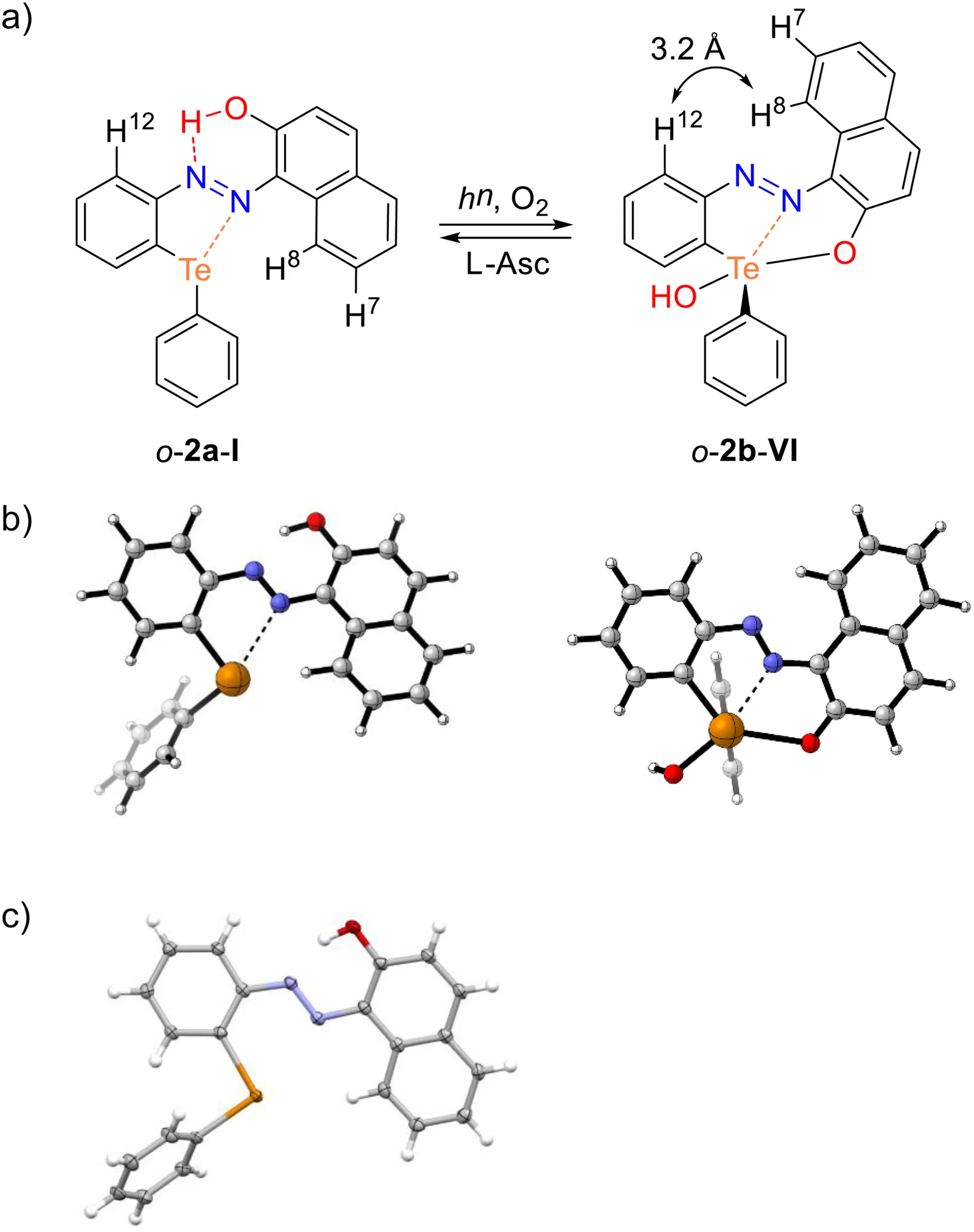
(a) Photochemical-induced single bond rotation through self-sensitized photooxidation. (b) Molecular structures of *o*-2a-I and *o*-2b-VI are calculated by means of PBE0-D3(SMD, MeOH)/def2-TZVP. (c) Molecular structure of *o*-2a in the solid state with thermal ellipsoids at 50% probability level.

Although all azoarenes 2–4 are capable of self-sensitized photooxidation, only for the *ortho*-tellurium substituted derivatives is isomerization of the N=N double bond clearly suppressed, enabling the desired isolated rotation around a σ bond. However, only the *ortho*, *ortho*-substituted compounds *o*-2a and 3a can form an intramolecular Te⋯O interaction, which provides the necessary driving force (Fig. 2). We therefore investigated whether the self-sensitized photooxidation of these azoarenes could induce rotation around the single bond adjacent to the N=N double bond. This involves an alteration in the preferred binding site of the oxygen atom before and after irradiation, driven by the strengthening of the chalcogen bond upon introduction of electron-withdrawing substituents at the tellurium center (Fig. 2). In a first step, the possible conformers of the starting materials *o*-2a and 3a (Fig. S46) were calculated using quantum chemical methods (PBE0-D3(SMD, MeOH)/def2-TZVP, Table S4). In principle, the tellurium center can form either a four- or five-membered ring with the azo nitrogen, and the OH group can adopt a *syn* or *anti* orientation relative to the tellurium atom. Previous studies have shown that the formation of the five-membered ring is strongly favored in tellurium-containing systems,^51–53^ so only these geometries were considered. For both derivatives, the *anti* conformers emerged as the most stable isomers (Fig. 5b and S46). Accordingly, the ground state structures correspond to *o*-2a-I and 3a-I, in which the PhTe and OH groups adopt an *anti* conformation relative to each other (Fig. S48 and S49).

If light-induced rotation around the N–C single bond occurs, the Te(iv) center and the oxygen atom should adopt a *syn* conformation. Consequently, in the photoproduct *o*-2b, the H^8^ proton on the naphthyl ring must approach the H^12^ proton on the phenyl ring (Fig. 5a). 1D-NOESY experiments on *o*-2b indeed show that the distance between H^8^ and H^12^ is approximately 3.2 Å (Fig. S28), with the H^7^–H^8^ distance of 2.4 Å used as a reference. In contrast, for *o*-2a (Fig. S27) and the deprotonated form (Fig. S29), a significant signal is detectable only for the H^7^–H^8^ interaction. While very weak cross peaks are also present in the spectra, including the H^8^–H^12^ region, comparable low-intensity features are observed for several other resonances and do not allow an unambiguous assignment of a minor *syn* conformational population. Thus, the NOESY data are consistent with an *anti*-dominated ground-state conformation. These 1D-NOESY experiments provide direct evidence for light-induced single bond rotation in the azoarene *o*-2a. Unlike previously reported systems exhibiting such molecular motion, this represents an individually addressable single bond rotation that leads to a stable state whose back-rotation occurs only upon addition of a reducing agent, such as l-ascorbic acid.

To further evaluate the experimentally observed *syn* conformation of the photoproducts *o*-2b and 3b, quantum-chemical calculations were performed (PBE0-D3(SMD, MeOH)/def2-TZVP, Table S4 and Fig. S47). Comparison of the calculated conformers and tautomers reveals a pronounced dependence of the conformational preference on the structural description of the oxidized tellurium species. For the hydroxy tellurium(iv)/phenolate formulation, the *syn* isomers *o*-2b-VI and 3b-VI, in which the Te(iv) center formally coordinates two anionic ligands, are by far the most stable conformers (Fig. S47 and S51). In contrast, the alternative telluroxide-types favor the *anti*-conformations *o*-2b-IV and 3b-IV (Fig. S47). This distinction is particularly relevant in view of the experimental 1D NOESY data: upon photooxidation of *o*-2a, a pronounced H^8^–H^12^ interaction between the naphthol and phenyl rings is observed, consistent with their close spatial proximity in the *syn* conformer, whereas no significant H^8^–H^12^ interaction is observed for the starting material. The calculated conformational preference of the hydroxy tellurium(iv)/phenolate structure VI is therefore consistent with the experimentally observed photooxidation-induced conformational change, whereas the calculated preferences of the alternative telluroxide formulations are not. The calculated structure therefore represents the formulation that is most consistent with the combined spectroscopic and computational evidence. Taken together, these results suggest that photooxidation of the tellurium center is accompanied by deprotonation of the phenol, which then acts as an anionic ligand for the Te(iv) center in the proposed structure VI. These findings also explain why prior deprotonation of *o*-2a with KO*t*-Bu yields the same photoproduct (Fig. S33). To further support the structures of *o*-2a and *o*-2b, the NMR chemical shifts of the most stable conformers *o*-2a-I, *o*-2a-III and *o*-2b-VI were simulated in methanol (Fig. 5b and S46). The calculated ^1^H and ^13^C NMR shifts agree well with the experimental values (Table S2). For example, the calculations predict a 1 ppm downfield shift for the H^8^ proton of *o*-2a upon oxidation. Likewise, the experimentally determined ^13^C NMR shifts of the quaternary Te and O carbons (C_quart_–Te: 119 ppm; C_quart_–O: 176 ppm) match closely with the calculated values (120 ppm and 176 ppm). The calculated NMR shifts for the phenol derivative 3a and its photoproduct 3b are likewise consistent with the experimental trends (Table S3).

As mentioned above, quantitative conversion of the photoproducts *o*-2b and 3b back to the Te(ii) species *o*-2a and 3a can be achieved by the addition of l-ascorbic acid (Fig. S21 and S24). To directly demonstrate the reversibility of the switching process, discrete cycling experiments consisting of alternating photooxidation and reduction steps were performed. Particularly promising behavior was observed for phenol derivative 3a, which undergoes five consecutive switching cycles with less than 5% degradation (Fig. S39). In contrast, *o*-2a shows substantially more pronounced decomposition under discrete cycling conditions (Fig. S38). Continuous irradiation experiments in the presence of l-ascorbic acid reveal a more complex, compound-dependent behavior. During irradiation, l-ascorbic acid is gradually converted to dehydroascorbic acid (Fig. S35 and S36), while control experiments with reference compounds 6 and 7 and l-ascorbic acid alone show no corresponding oxidation (Fig. S37). For *o*-2a, the presence of l-ascorbic acid suppresses degradation compared with discrete cycling, suggesting that the reductant additionally interferes with competing oxidative degradation pathways. Interestingly, the opposite trend is observed for 3a, for which continuous irradiation in the presence of l-ascorbic acid results in lower selectivity than the discrete switching experiment. The continuous-irradiation experiments therefore cannot be used to quantify the number of switching events from reductant consumption alone but instead illustrate the compound-dependent interplay between photooxidation, reduction, and competing degradation pathways.

### 2.6 Long wavelength photooxidation

In the preceding sections, we demonstrated that *o*-2a and 3a undergo self-sensitized photooxidation upon UV irradiation (*λ* = 365 nm), inducing an isolated rotation around the N–C single bond adjacent to the azo bridge. Because the accompanying color change was also observed upon prolonged exposure of the samples to daylight, we repeated the above experiments using different light sources. Irradiation of a 1 mm solution of *o*-2a in methanol with light at 530 nm over 20 minutes resulted in only minimal changes in the UV/Vis absorption spectrum (Fig. S12). Except for 4a, which shows selective conversion (Fig. S16 and S31), the other compounds also exhibited only very slow photooxidation under green light. Although *o*-2a absorbs at longer wavelengths (Fig. S12), the markedly lower conversion observed upon irradiation at 530 nm demonstrates that light absorption alone does not determine the efficiency of the transformation. Instead, excitation at different wavelengths may populate excited-state pathways with different efficiencies, resulting in different photooxidation yields. Changing the solvent from methanol to ethyl acetate markedly enhanced the self-sensitized photooxidation of the naphthol derivative *o*-2a, not only under 530 nm light but also under UV irradiation (Fig. S13 and S14). The origin of this solvent dependence remains unclear. Differences in hydrogen bonding and/or stabilization of reactive intermediates may contribute to the observed behavior; however, the available data do not permit a more detailed mechanistic interpretation. In the case of 3a, photooxidation in methanol can also be achieved using visible light (530 nm) when small amounts of the photosensitizer TPP (tetraphenylporphyrin) are added (Fig. S40).

Subsequently, the photooxidation of *o*-2a was studied under sunlight in both UV/Vis (*c* = 1 mm, Fig. S15) and NMR (*c* = 5 mm, Fig. S30) experiments in ethyl acetate. For the NMR experiments, a mixture of 90% non-deuterated ethyl acetate and 10% MeOD was used. Complete photooxidation was observed in the UV/Vis experiment after approximately 40 minutes. The NMR experiment confirmed the selectivity observed in previous experiments, showing a strong predominance of the photoproduct *o*-2b (9 : 1) after just 5 hours.

These results demonstrate that the system undergoes self-sensitized photooxidation and associated single bond rotation upon irradiation with sunlight.

## 3 Conclusion

We report the first example of light-induced N–C single bond rotation adjacent to the azo bridge in β-hydroxy-substituted azoarenes. This unprecedented motion is initiated by self-sensitized photooxidation of a tellurium center, which alters the preferred binding site of the hydroxy group. Remarkably, the transformation can be triggered not only by UV light but also by sunlight, yielding a stable oxidized state that is fully reversible upon treatment with a reducing agent. Beyond the specific molecular system investigated here, coupling photoexcitation to reversible redox chemistry may provide a design principle for controlling conformational changes in other redox-active molecular systems. In contrast to conventional photoswitches controlled primarily by the irradiation conditions, the present system integrates an additional chemical level of control: the switched state is generated under aerobic, non-reducing conditions and can be reset by reduction. The pronounced color change accompanying this process further illustrates the potential of such systems as environment-responsive molecular switches. Future studies will show to what extent this concept can be transferred to other molecular scaffolds and whether the requirements identified here can be relaxed through further molecular design.

## Author contributions

Zoe Nonie Scheller: conceptualization, data curation, formal analysis, investigation, methodology, visualization, writing – original draft, review and editing; Jan Schulte: formal analysis, investigation; Christoph Wölper: data curation; Gebhard Haberhauer: conceptualization, formal analysis, investigation, resources, supervision, writing – original draft, review and editing.

## Conflicts of interest

There is no conflicts of interest to declare.

## Supplementary Material

SC-OLF-D6SC06305E-s001

SC-OLF-D6SC06305E-s002

## Data Availability

CCDC 2504630 (1-S) and 2504631 (*o*-2a) contain the supplementary crystallographic data for this paper.^80*a*,*b*^ The data that support the findings of this study have been included as part of the supplementary information (SI). Raw data are available from the corresponding authors upon reasonable request. Supplementary information: figures and tables, NMR, UV, and fluorescence spectra, synthesis of new compounds, computational details, cartesian coordinates, and absolute energies for all calculated compounds and ^1^H NMR, ^13^C NMR, ^77^Se NMR, and ^125^Te NMR spectra of the new compounds are provided in the supplementary information file. See DOI: https://doi.org/10.1039/d6sc06305e.
