# Supplementary material for: Visible light-induced single bond rotation in azoarenes triggered by self-sensitized oxidation
Source: Chem Sci. 2026 Sep 15. Online ahead of print. doi: 10.1039/d6sc06305e (PMC13595109; doi:10.1039/d6sc06305e)
Supplement: Supplementary file 1 [file SC-OLF-D6SC06305E-s001.pdf]

Supplementary Information (SI) for Chemical Science.
This journal is © The Royal Society of Chemistry 2026

Supporting Information

Visible Light-Induced Single Bond Rotation in Azoarenes Triggered by Self-Sensitized Oxidation

Zoe Nonie Scheller*†, Jan Schulte†, Christoph Wölper† and Gebhard Haberhauer*†

† Institut für Organische Chemie, Universität Duisburg-Essen, Universitätsstr. 7, D-45117 Essen, Germany

1. **UV and NMR Photoirradiation Experiments** S2
2. **Fluorescence Spectra** S39
3. **Synthesis of New Compounds** S40
4. **Computational Details** S62
5. **Cartesian Coordinates and Absolute Energies for All Calculated Compounds** S67
6. **Crystal Structure Data** S81
7. **^{1}H NMR, ^{13}C NMR ^{77}Se NMR and ^{125}Te NMR Spectra of the New Compounds** S86
8. **Supporting Information References** S120

1. UV and NMR Photoirradiation Experiments

General procedure for the irradiation of the samples. As radiation sources, LEDs from Sahlmann Photochemical Solutions were used. The exact specifications are as follows: λ = 365 nm, LED type: 3× Nichia NC4U133A, total optical power: 3300 mW; λ = 405 nm, LED type: 3× Roithner APG2C1-405, total optical power: 315 mW; λ = 530 nm, LED type: 3× Luxeon LXML-PM01, total optical power: 763 mW. For the investigation of photooxidation, the sample was placed 1 cm from the light source and irradiated with the corresponding LED for the specified period. Then, a UV/Vis absorption spectrum was obtained with a Jasco V-550 spectrophotometer in a 1 mm quartz glass cuvette. This process was repeated until the starting material was consumed completely. Irradiation with sunlight was carried out in August 2025 in Essen, NRW, Germany (51° 27′ N, 7° 1′ E) on an SSE windowsill with an open window. Measurements of the irradiation strength with an SSL SPEKTRI S80.Ee light measuring tool showed a peak irradiance of 40 mW/cm^{-2}.

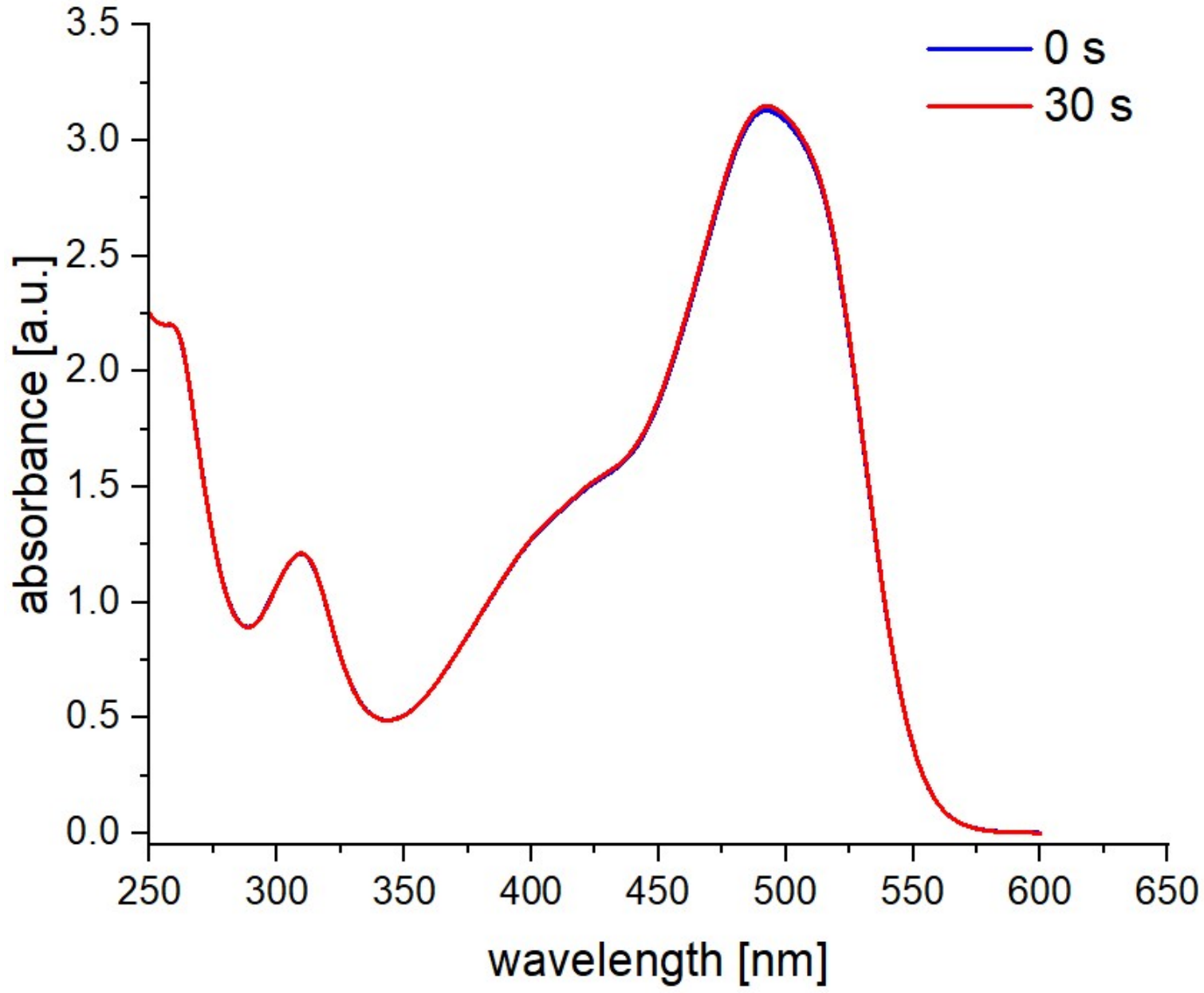

Figure S1. UV/Vis spectra of the azoarene **1-O**: after synthesis (0 s, blue) and after 30 s UV irradiation with λ = 365 nm (red, MeOH, c = 1.0 mM, d = 1 mm).

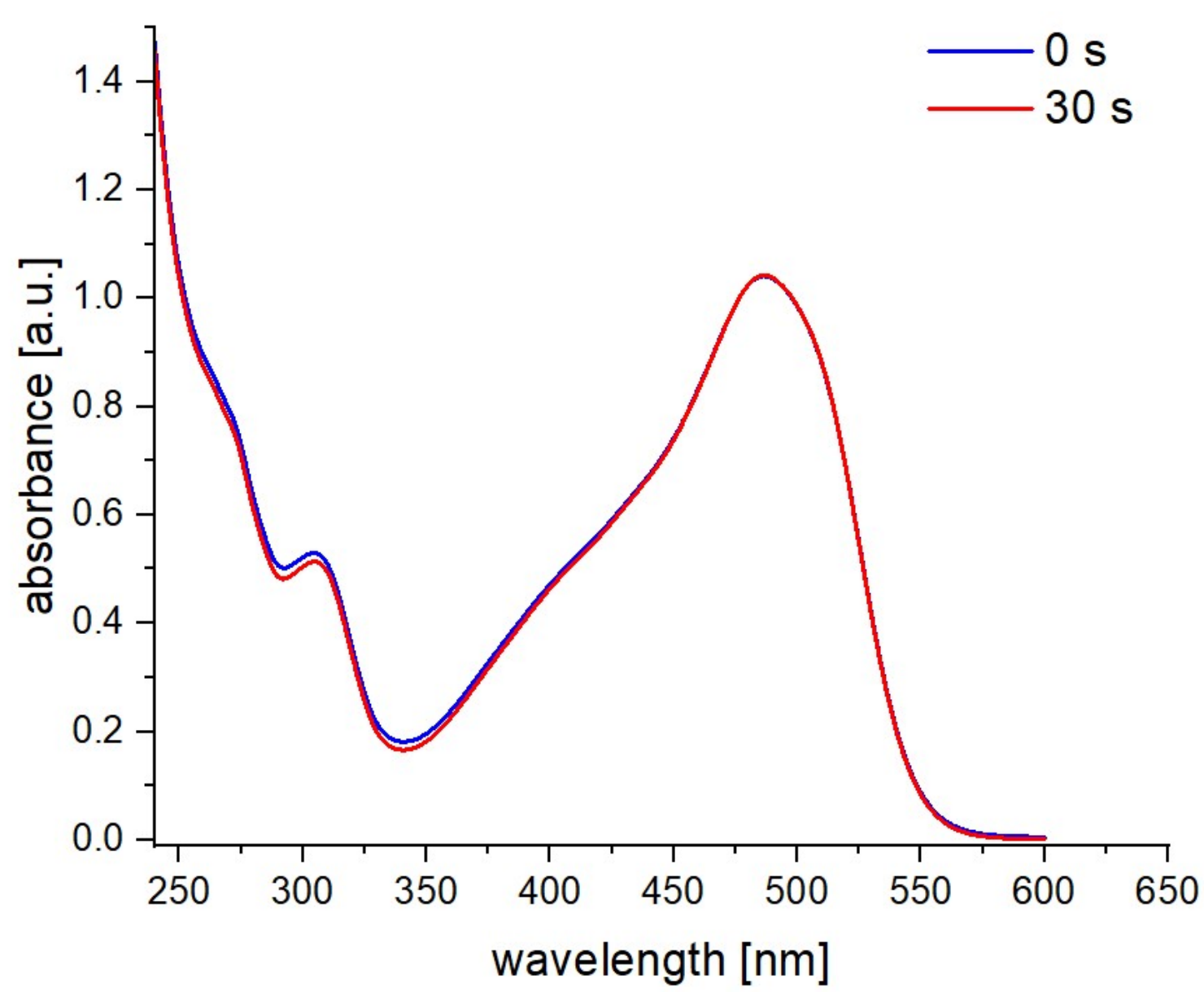

Figure S2. UV/Vis spectra of the azoarene **1-S**: after synthesis (0 s, blue) and after 30 s UV irradiation with λ = 365 nm (red, MeOH, c = 1.0 mM, d = 1 mm).

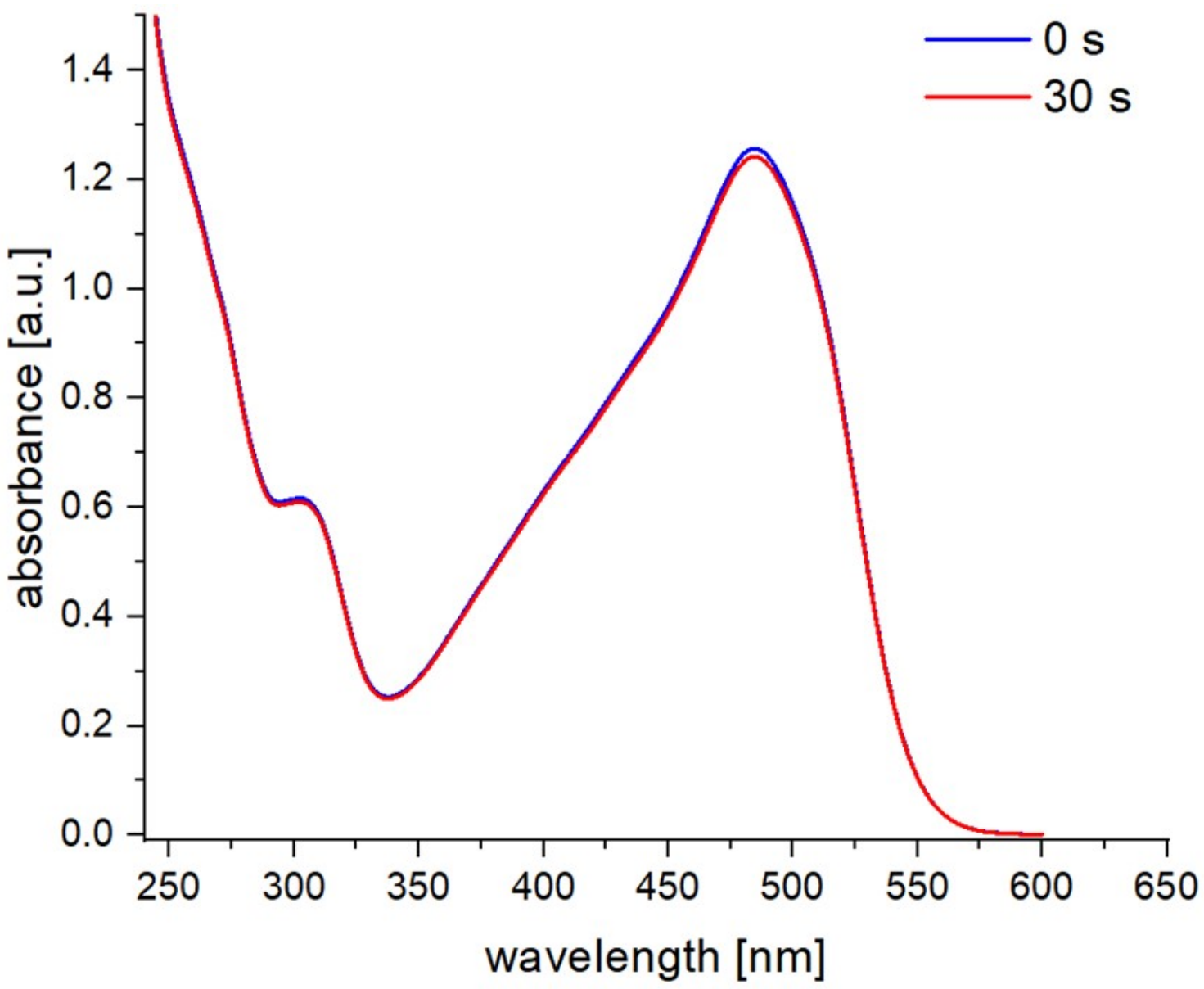

Figure S3. UV/Vis spectra of the azoarene **1-Se**: after synthesis (0 s, blue) and after 30 s UV irradiation with λ = 365 nm (red, MeOH, c = 1.0 mM, d = 1 mm).

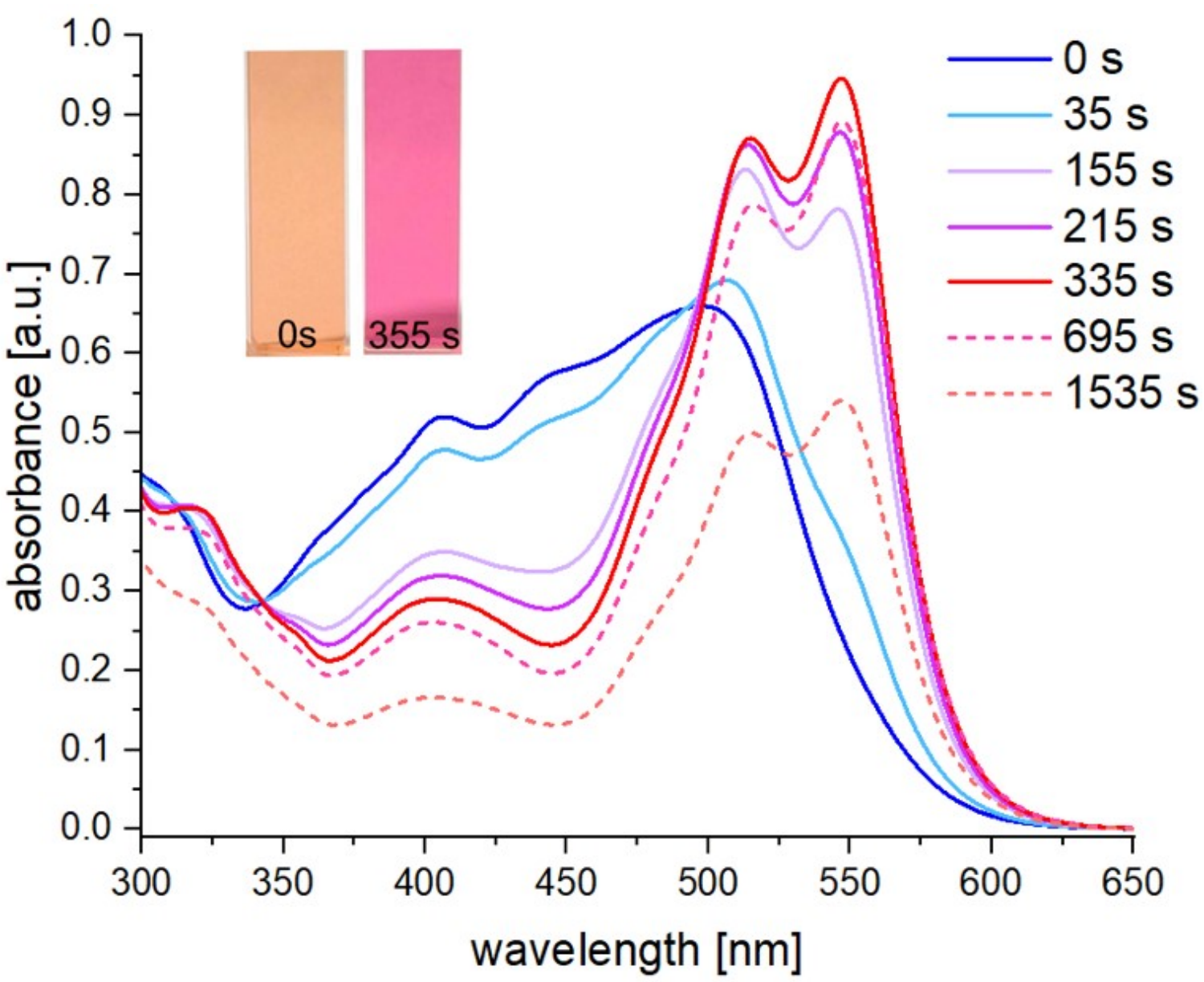

Figure S4. UV/Vis spectra of the azoarene *o*-**2a**: after synthesis (0 s, blue) and after UV irradiation with λ = 365 nm after different irradiation periods (MeOH, c = 1.0 mM, d = 1 mm). Picture of the UV/Vis cuvette (d = 1 mm) before (0 s) and after (355 s) of irradiation with λ = 365 nm.

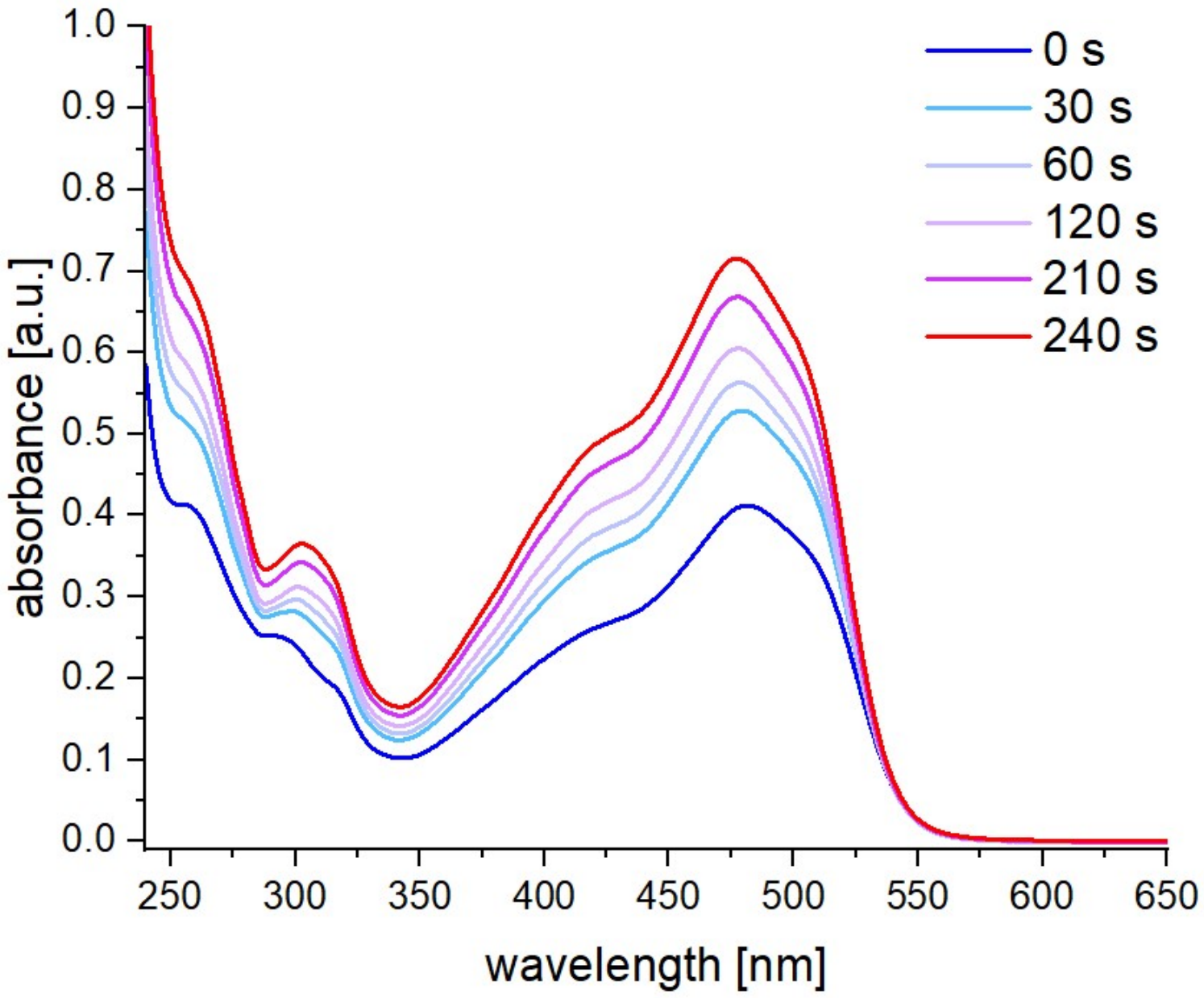

Figure S5. UV/Vis spectra of the azoarene *m*-**2a**: after synthesis (0 s, blue) and after UV irradiation with λ = 365 nm after different irradiation periods (MeOH, c = 0.5 mM, d = 1 mm).

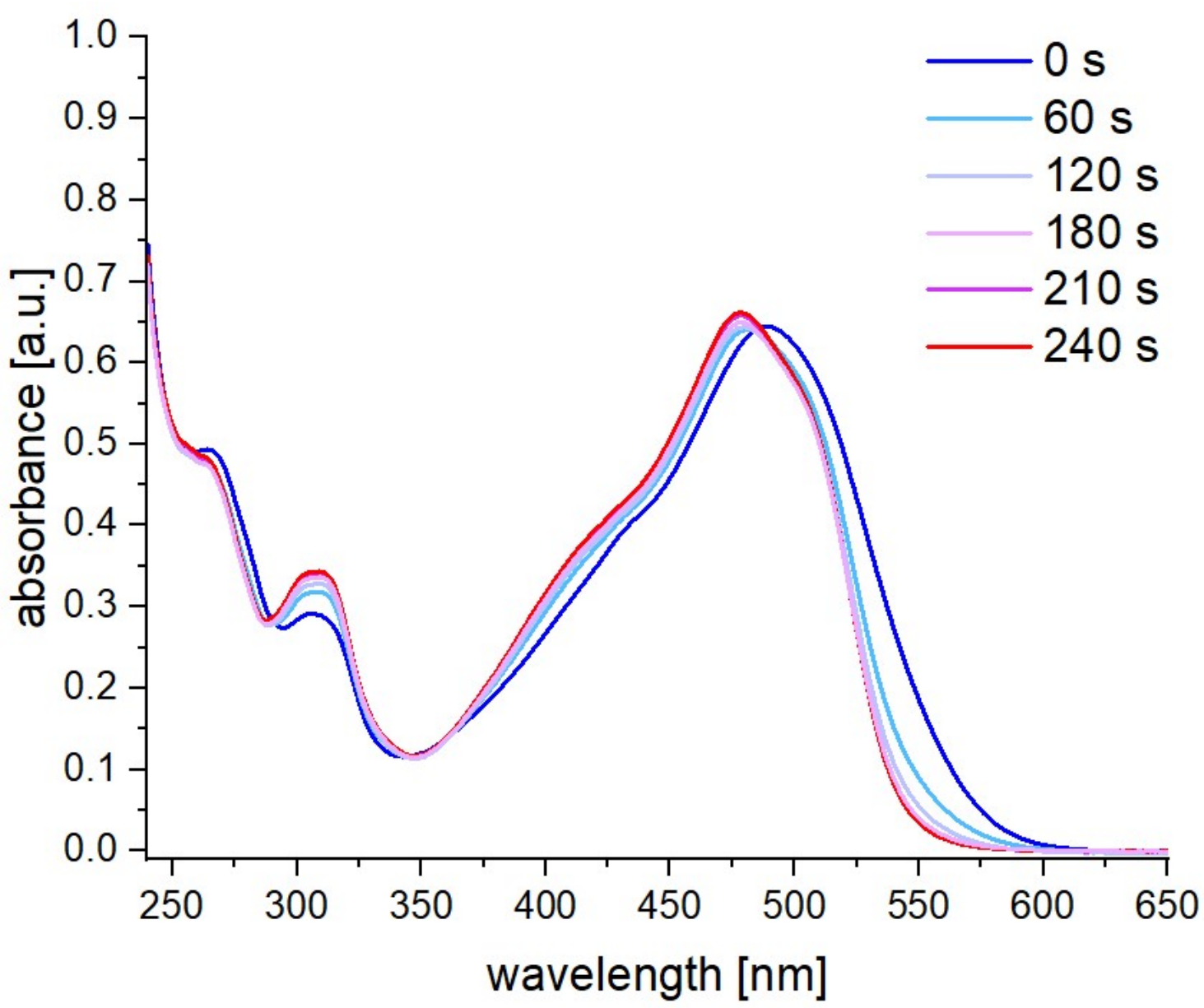

Figure S6. UV/Vis spectra of the azoarene *p*-**2a**: after synthesis (0 s, blue) and after UV irradiation with λ = 365 nm after different irradiation periods (MeOH, c = 0.5 mM, d = 1 mm).

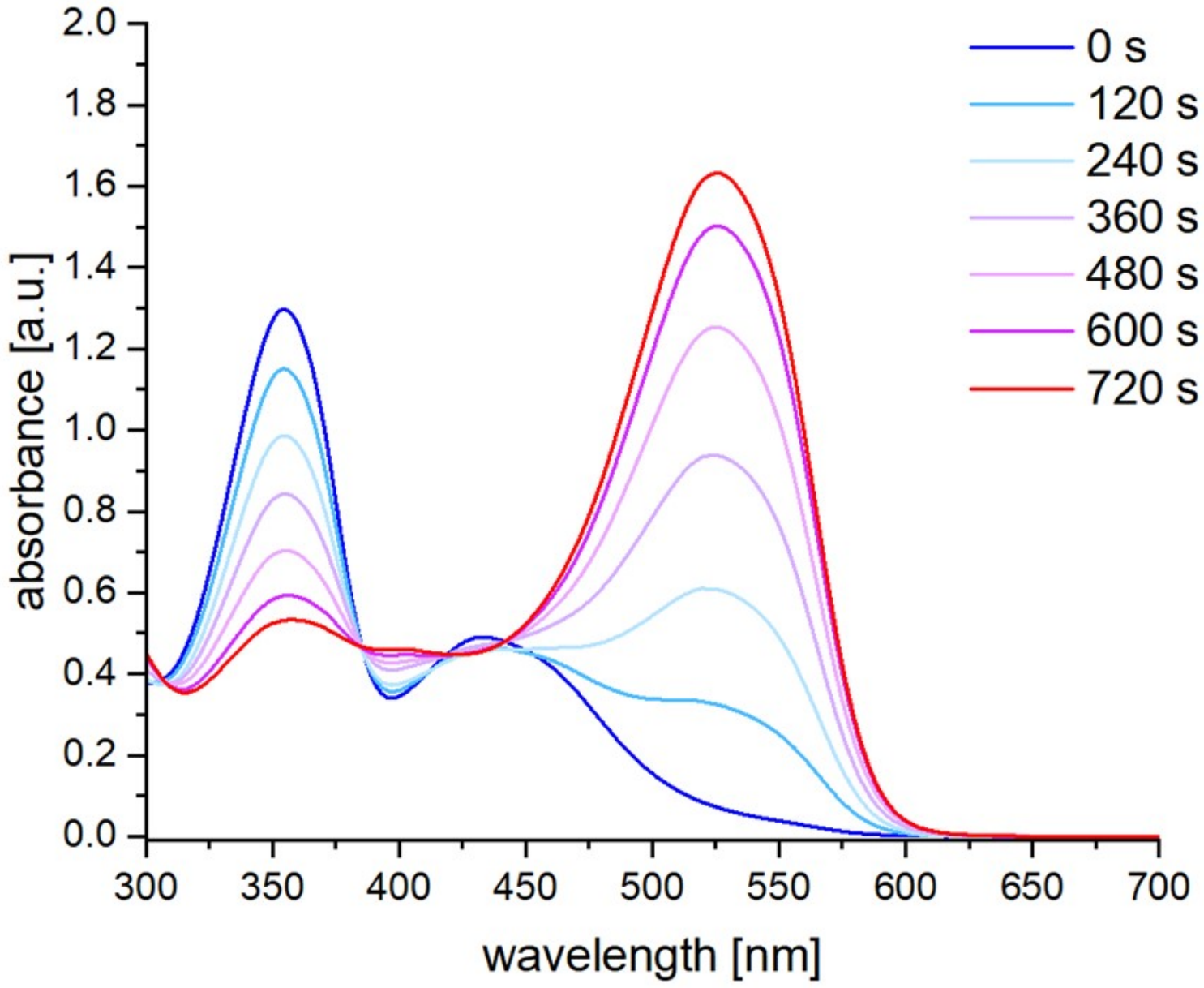

Figure S7. UV/Vis spectra of the azoarene **4a**: after synthesis (0 s, blue) and after UV irradiation with λ = 365 nm after different irradiation periods (MeOH, c = 1.0 mM, d = 1 mm).

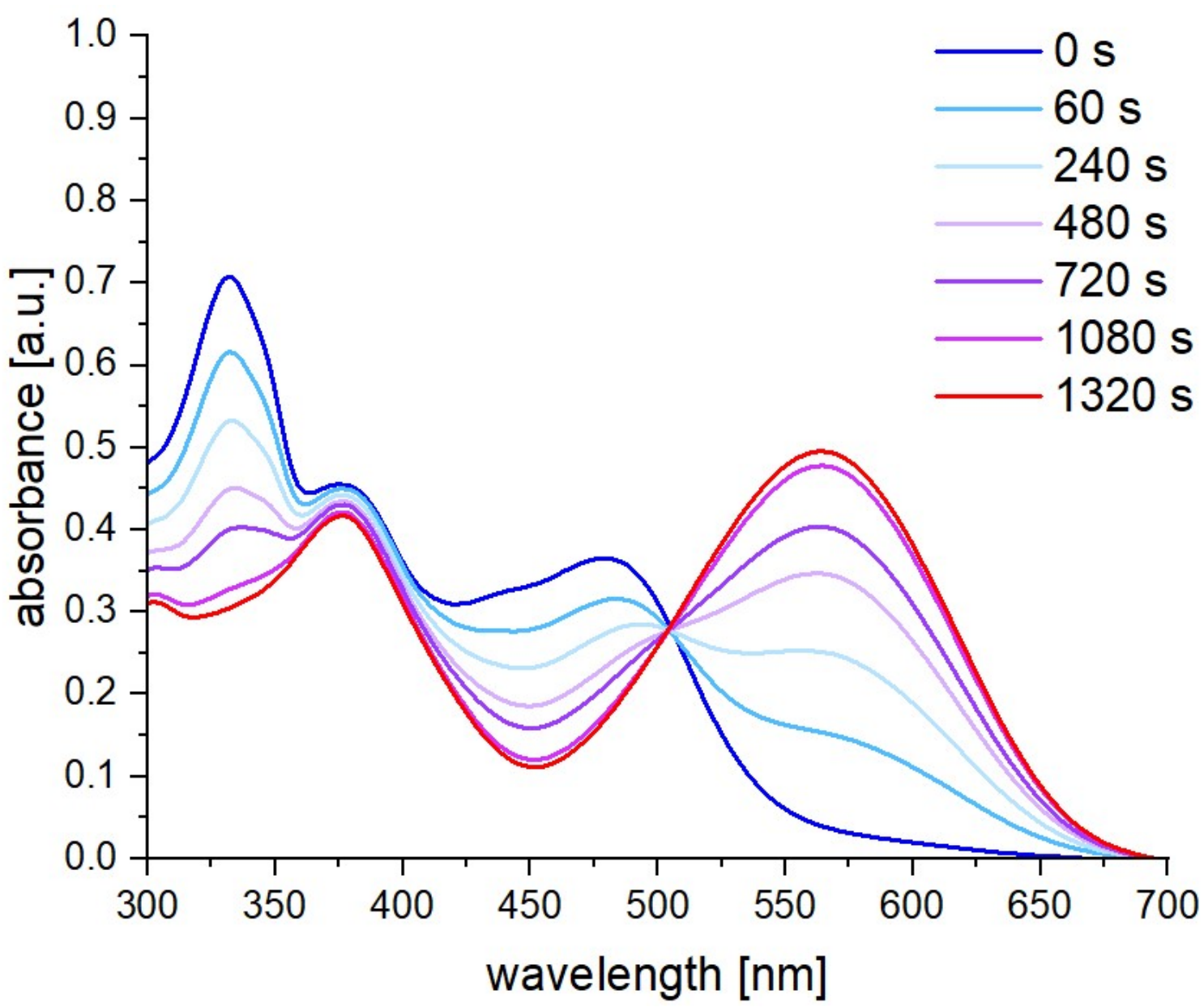

Figure S8. UV/Vis spectra of the azoarene **3a**: after synthesis (0 s, blue) and after UV irradiation with λ = 365 nm after different irradiation periods (MeOH, c = 1.0 mM, d = 1 mm).

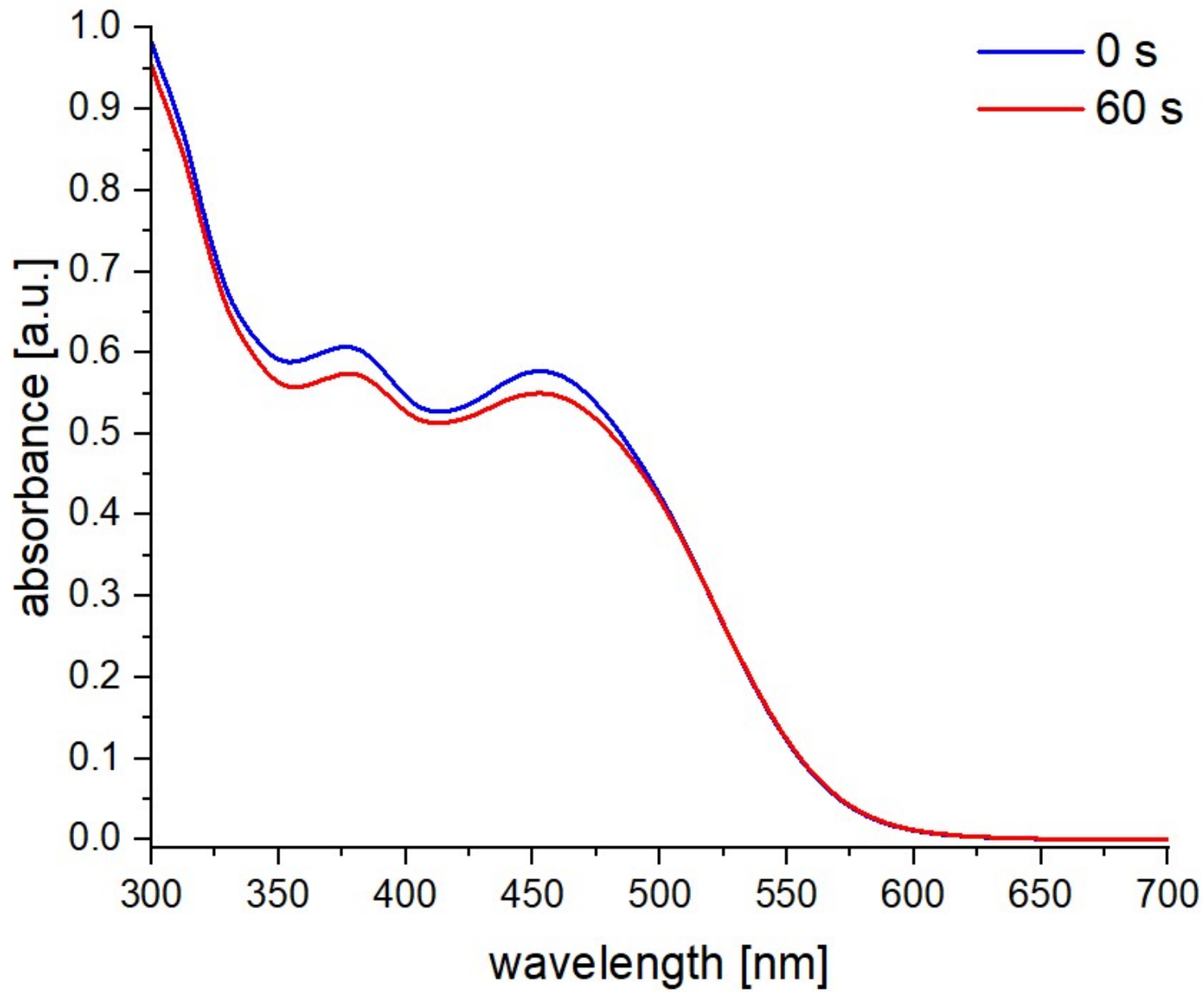

Figure S9. UV/Vis spectra of the azoarene **5**: after synthesis (0 s, blue) and after 60 s UV irradiation with λ = 365 nm (red, MeOH, c = 1.0 mM, d = 1 mm).

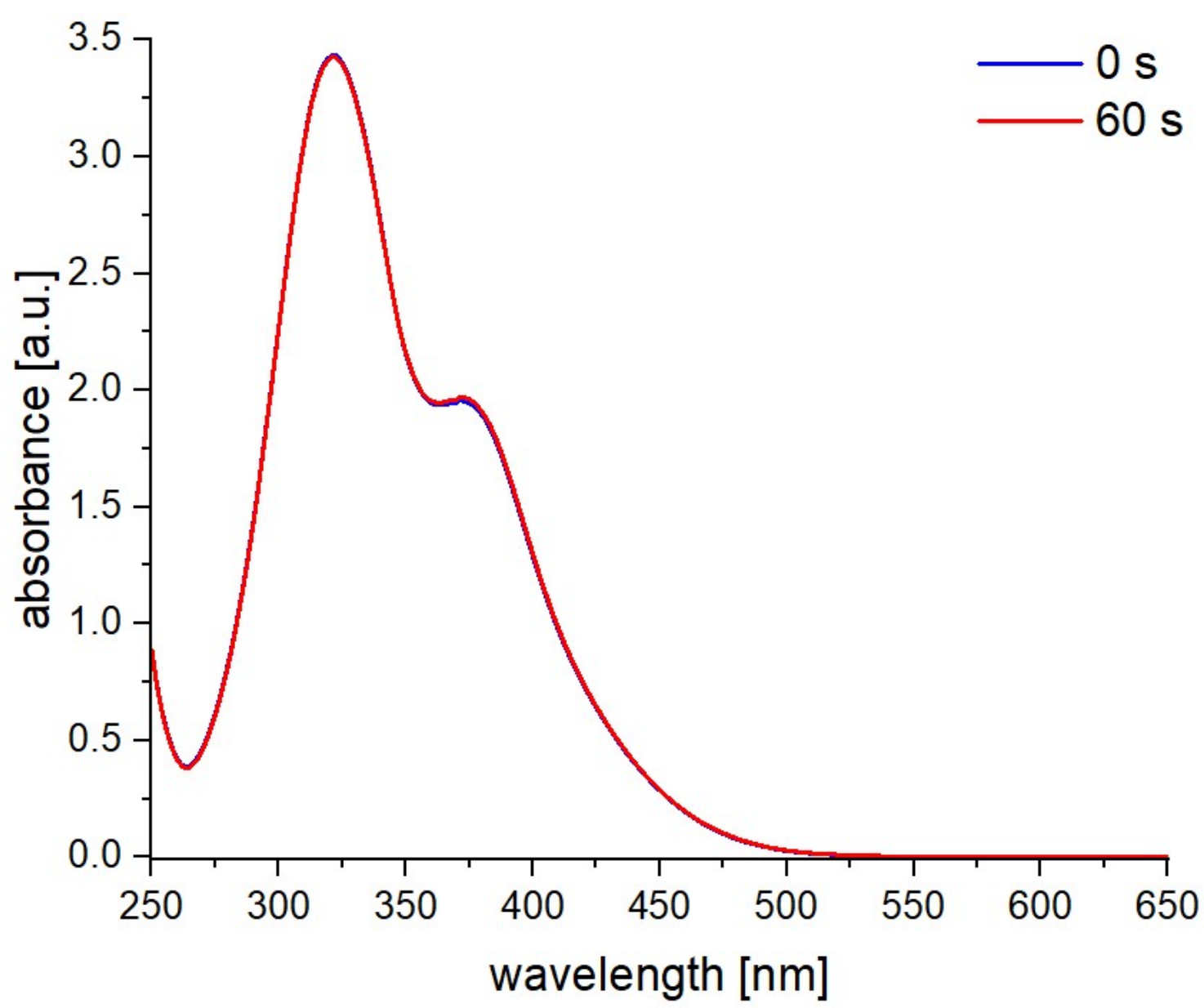

Figure S10. UV/Vis spectra of the azoarene **6**: after synthesis (0 s, blue) and after 60 s UV irradiation with λ = 365 nm (red, MeOH, c = 1.0 mM, d = 1 mm).

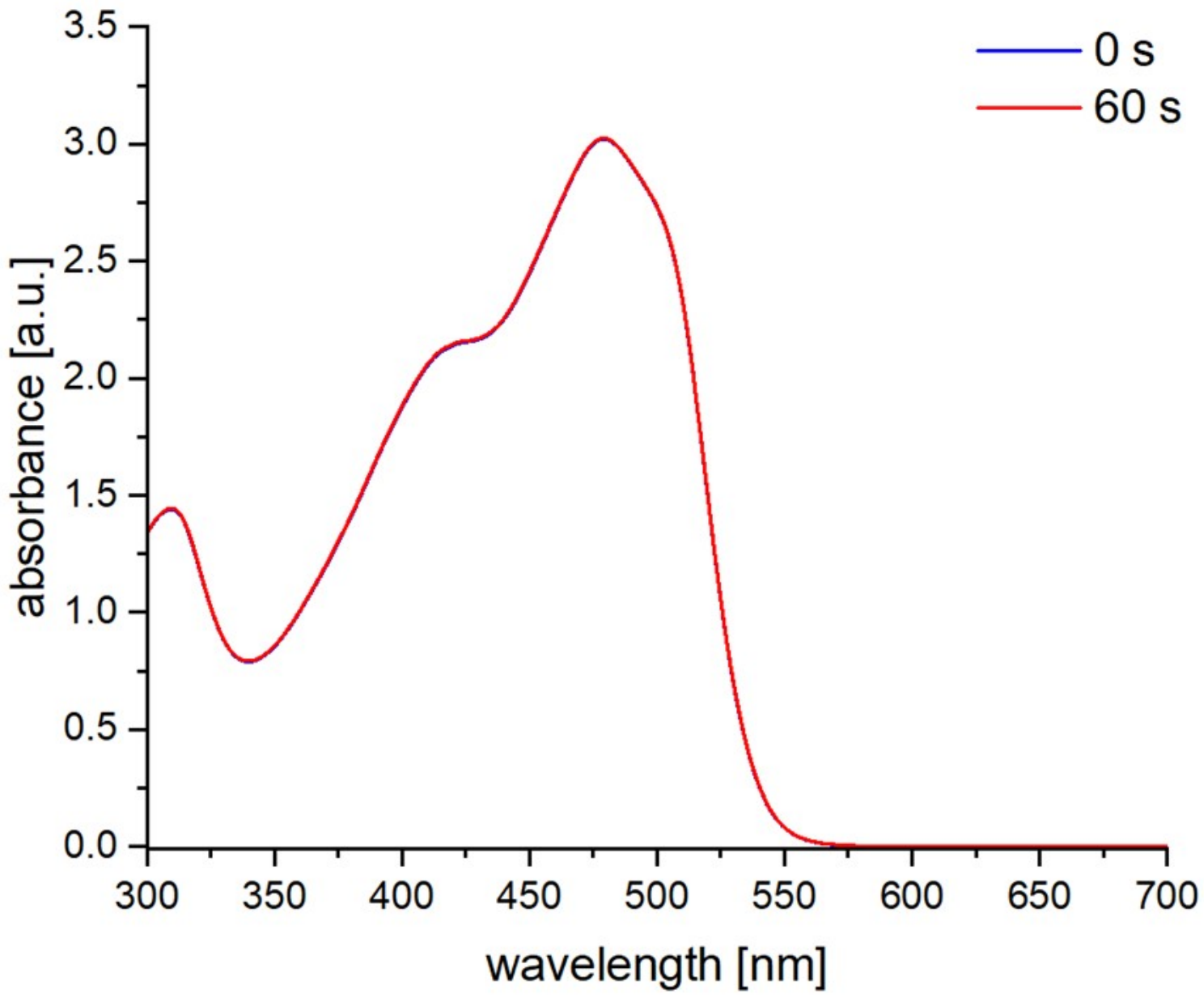

Figure S11. UV/Vis spectra of the azoarene **7**: after synthesis (0 s, blue) and after 60 s UV irradiation with λ = 365 nm (red, MeOH, c = 1.0 mM, d = 1 mm).

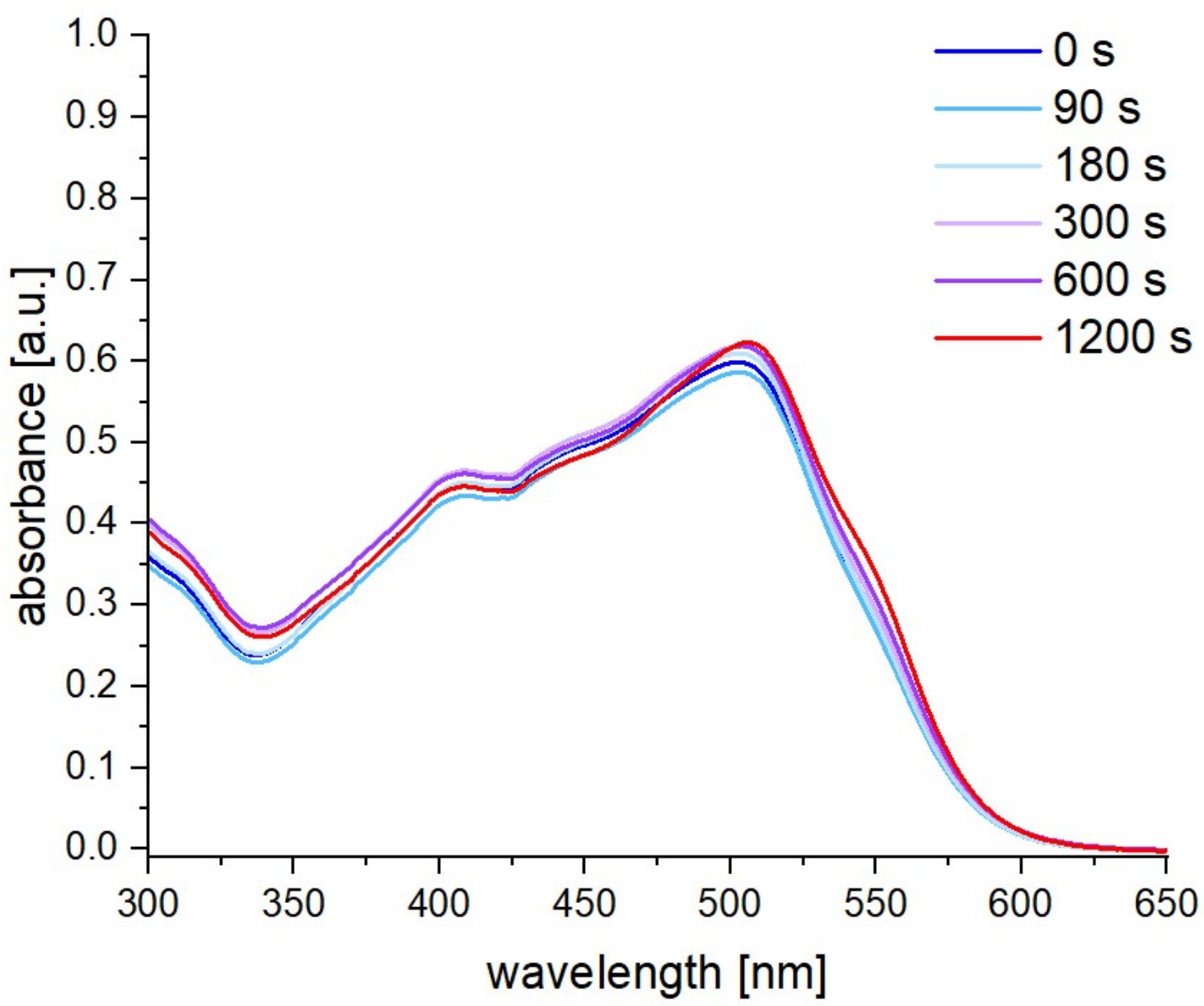

Figure S12. UV/Vis spectra of the azoarene *o*-**2a**: after synthesis (0 s, blue) and after irradiation with λ = 530 nm after different irradiation periods (MeOH, c = 1.0 mM, d = 1 mm).

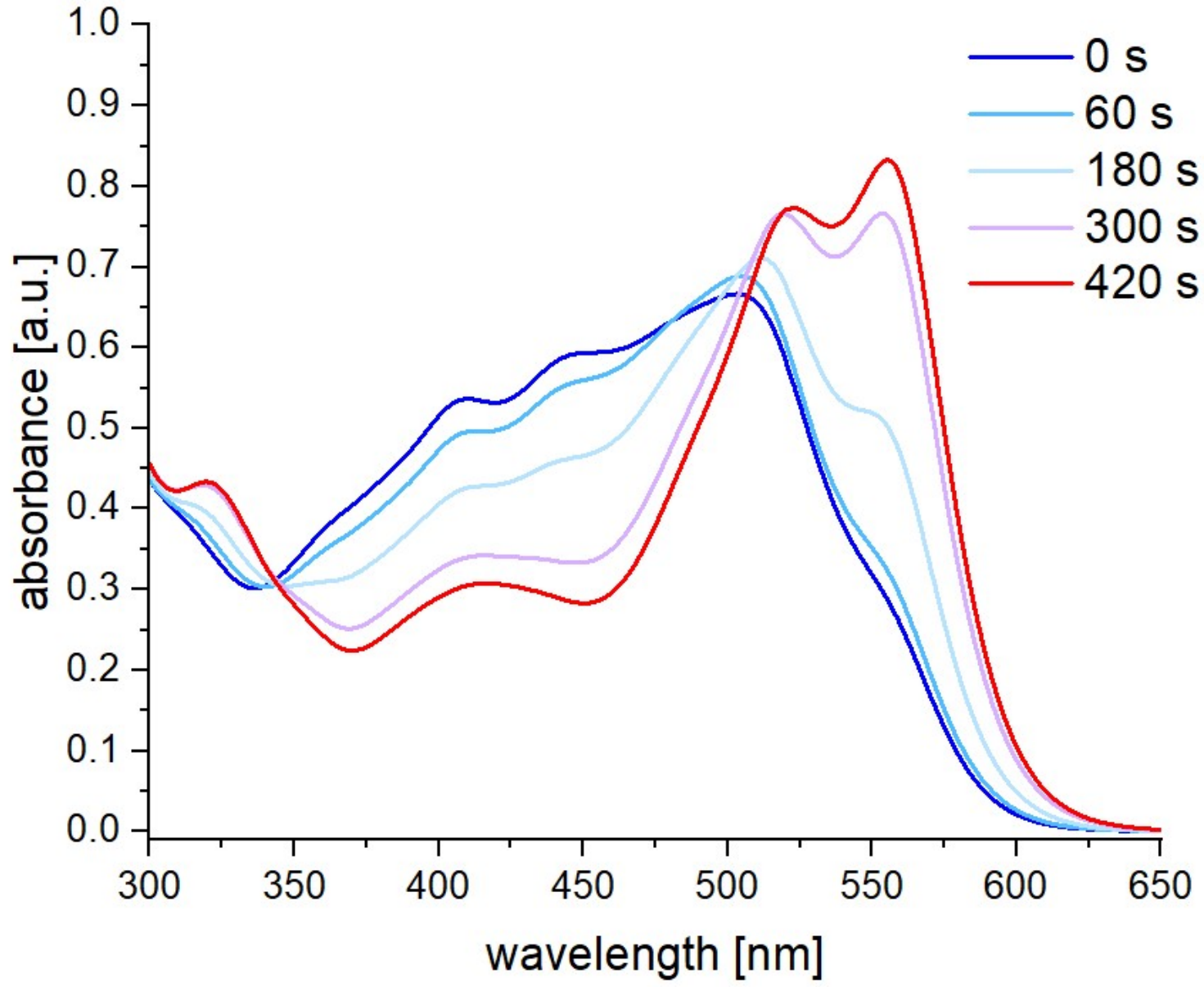

Figure S13. UV/Vis spectra of the azoarene *o*-**2a**: after synthesis (0 s, blue) and after UV irradiation with λ = 365 nm after different irradiation periods (ethyl acetate, c = 1.0 mM, d = 1 mm).

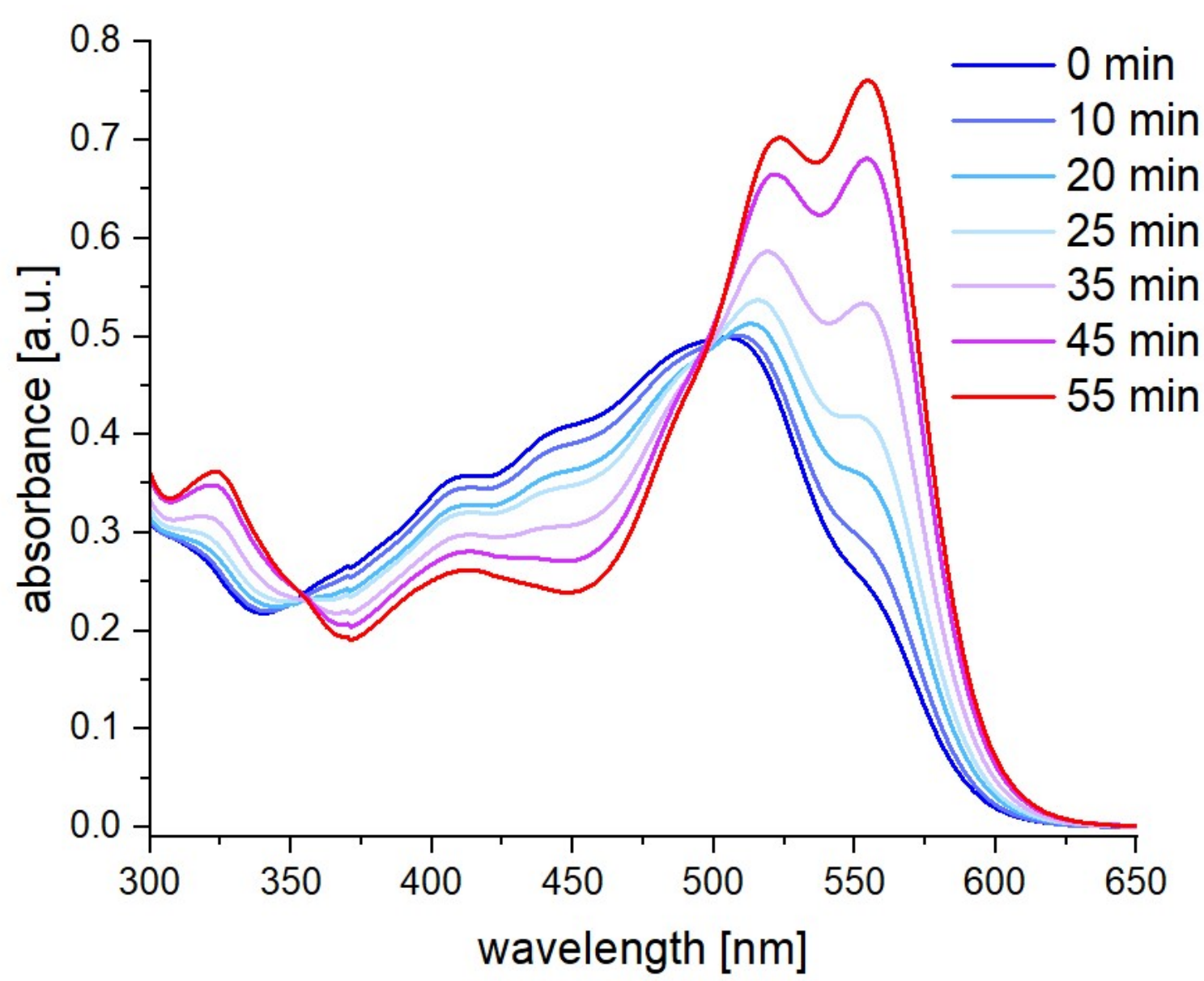

Figure S14. UV/Vis spectra of the azoarene *o*-**2a**: after synthesis (0 min, blue) and after irradiation with λ = 530 nm after different irradiation periods (ethyl acetate, c = 1.0 mM, d = 1 mm).

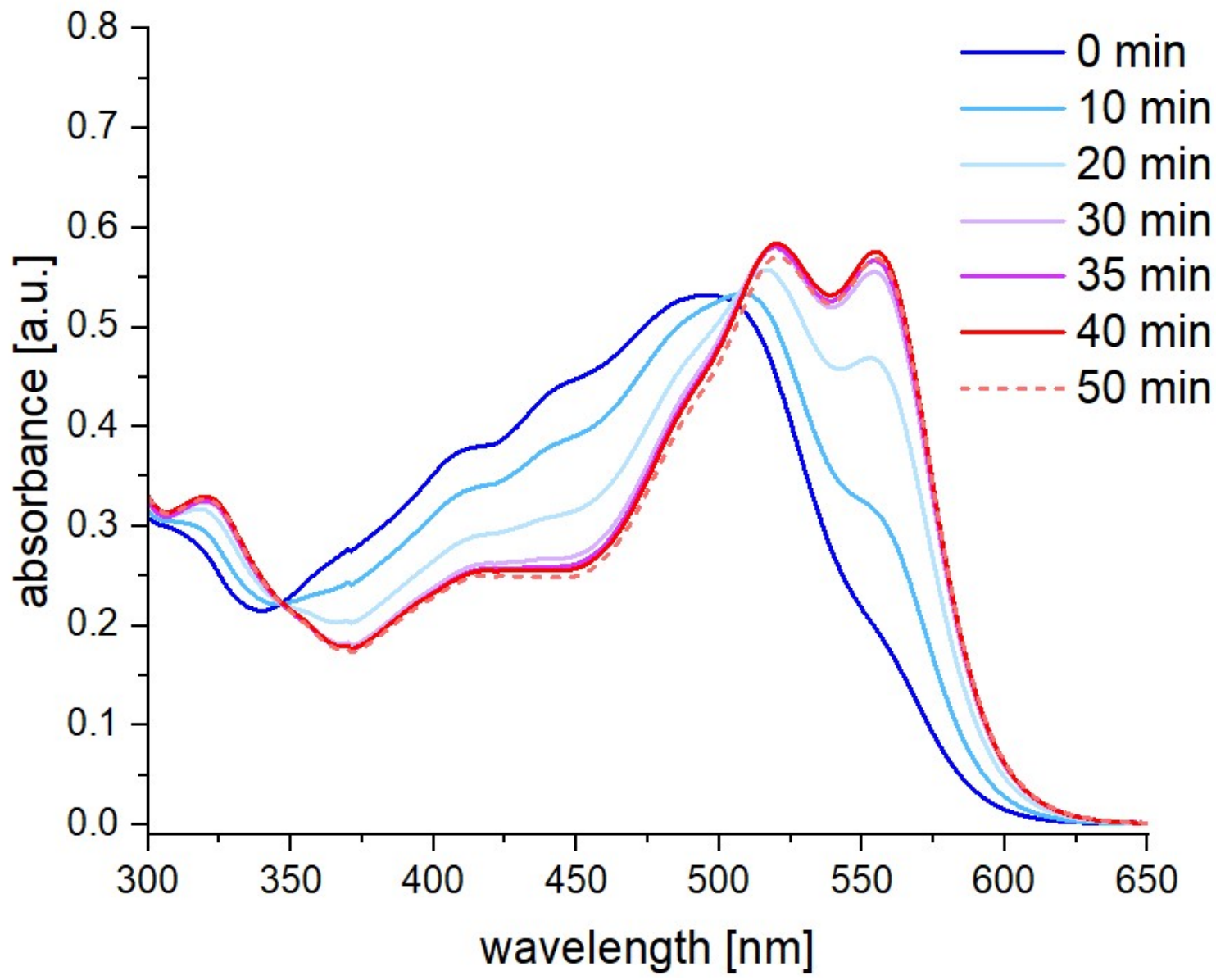

Figure S15. UV/Vis spectra of the azoarene *o*-**2a**: after synthesis (0 min, blue) and after irradiation with sunlight after different irradiation periods (ethyl acetate, c = 1.0 mM, d = 1 mm).

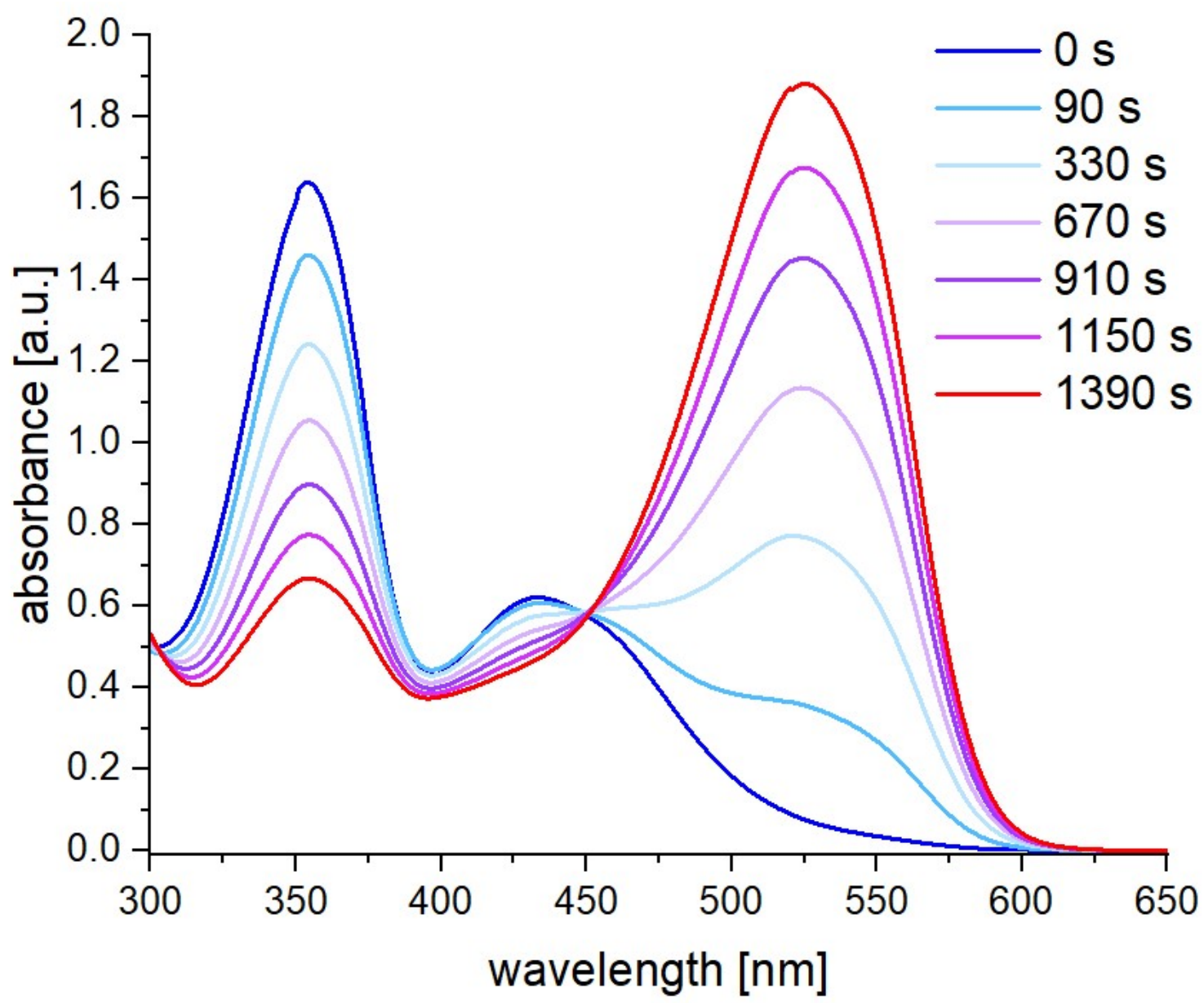

Figure S16. UV/Vis spectra of the azoarene **4a**: after synthesis (0 s, blue) and after UV irradiation with λ = 530 nm after different irradiation periods (MeOH, c = 1.0 mM, d = 1 mm).

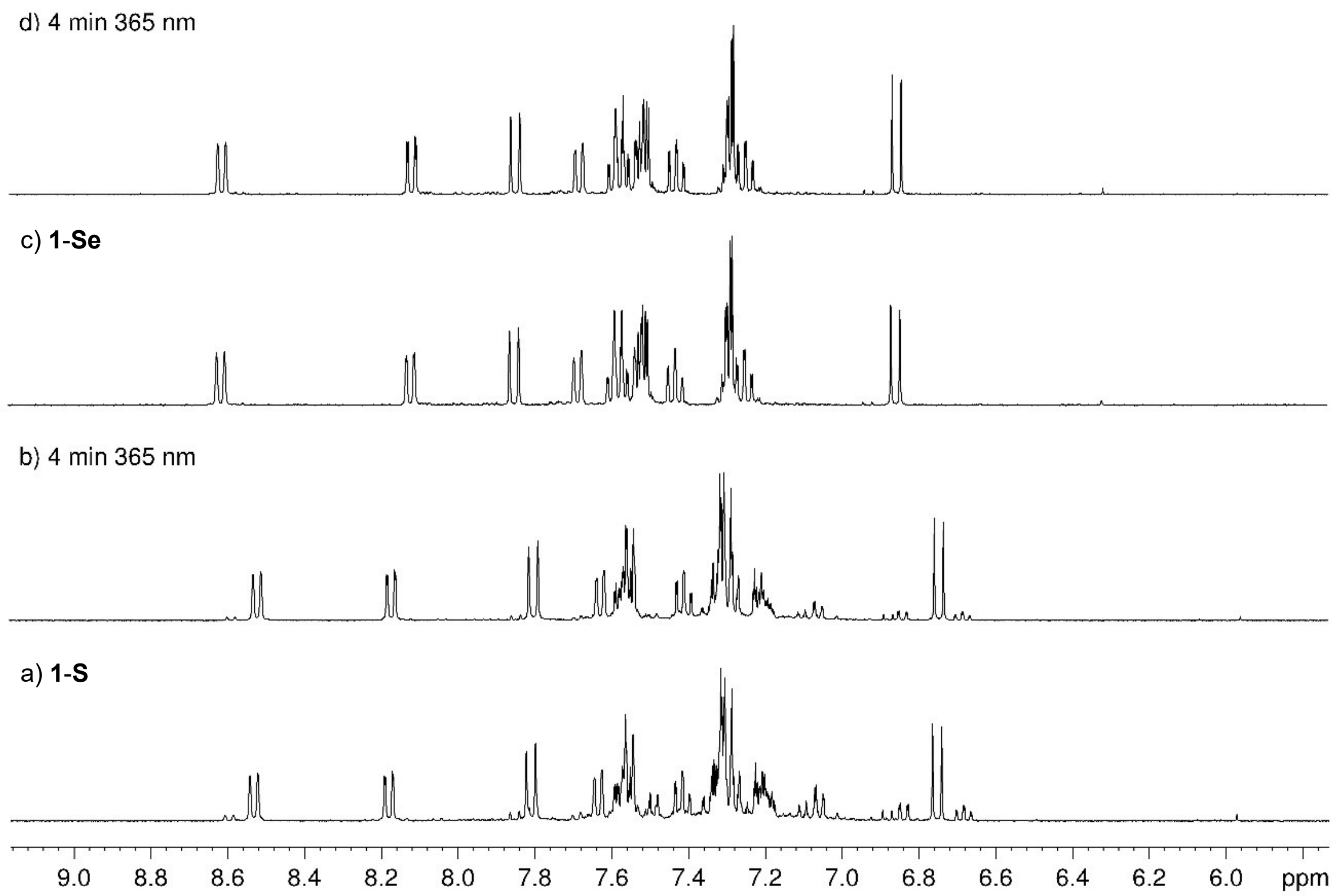

Figure S17. Section from the ^{1}H NMR spectra of the azoarene **1-S**: a) after synthesis and b) after 4 minutes UV irradiation with λ = 365 nm. Section from the ^{1}H NMR spectra of the azoarene **1-Se**: c) after synthesis and d) after 4 minutes UV irradiation with λ = 365 nm. (MeOD, 400 MHz, c = 5 mM).

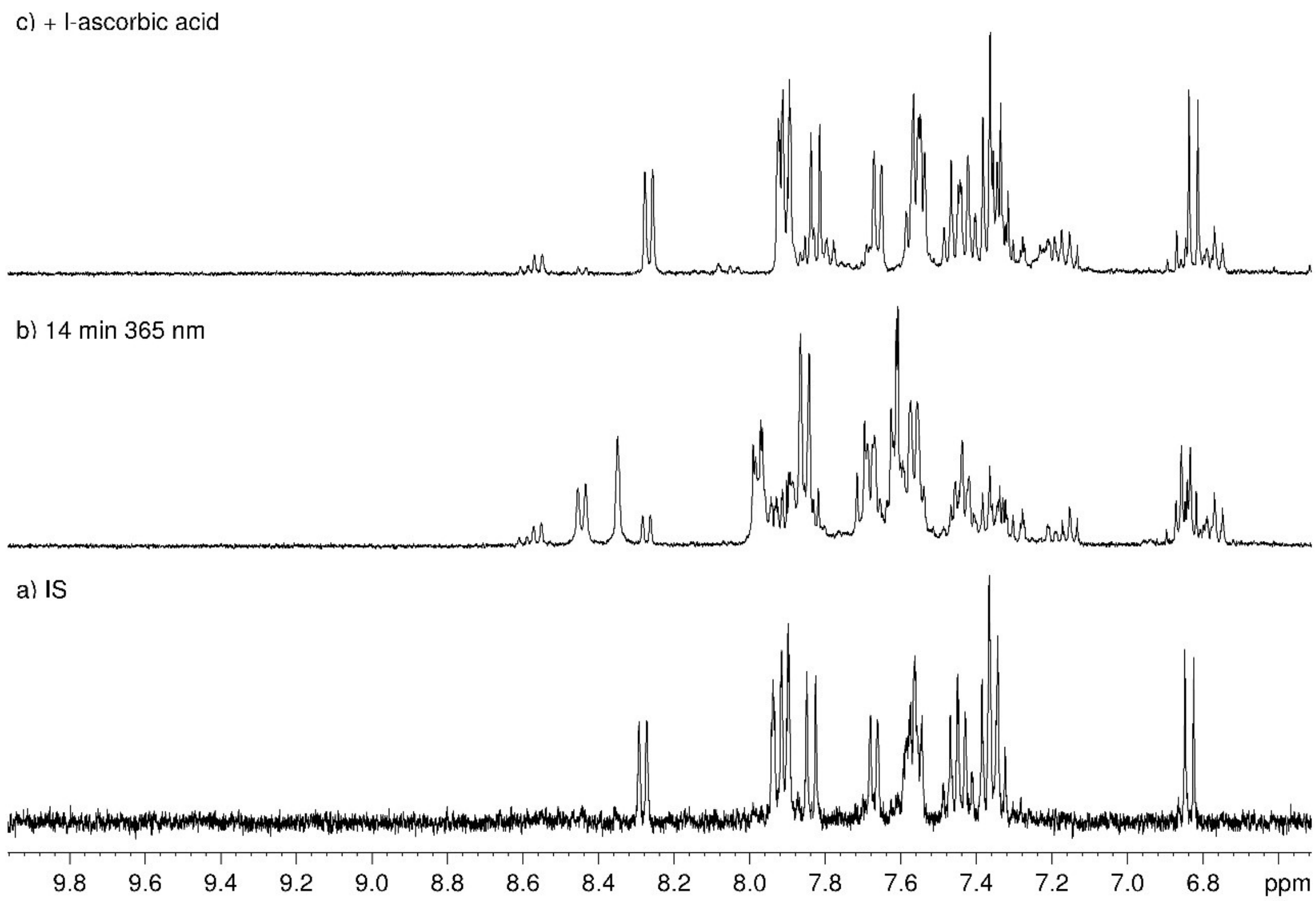

Figure S18. Section from the ^{1}H NMR spectra of the azoarene *m*-**2a**: a) after synthesis (initial state), b) after 14 minutes UV irradiation with λ = 365 nm, and c) after addition of L-ascorbic acid to the irradiated sample (MeOD, 400 MHz, c = 2.5 mM).

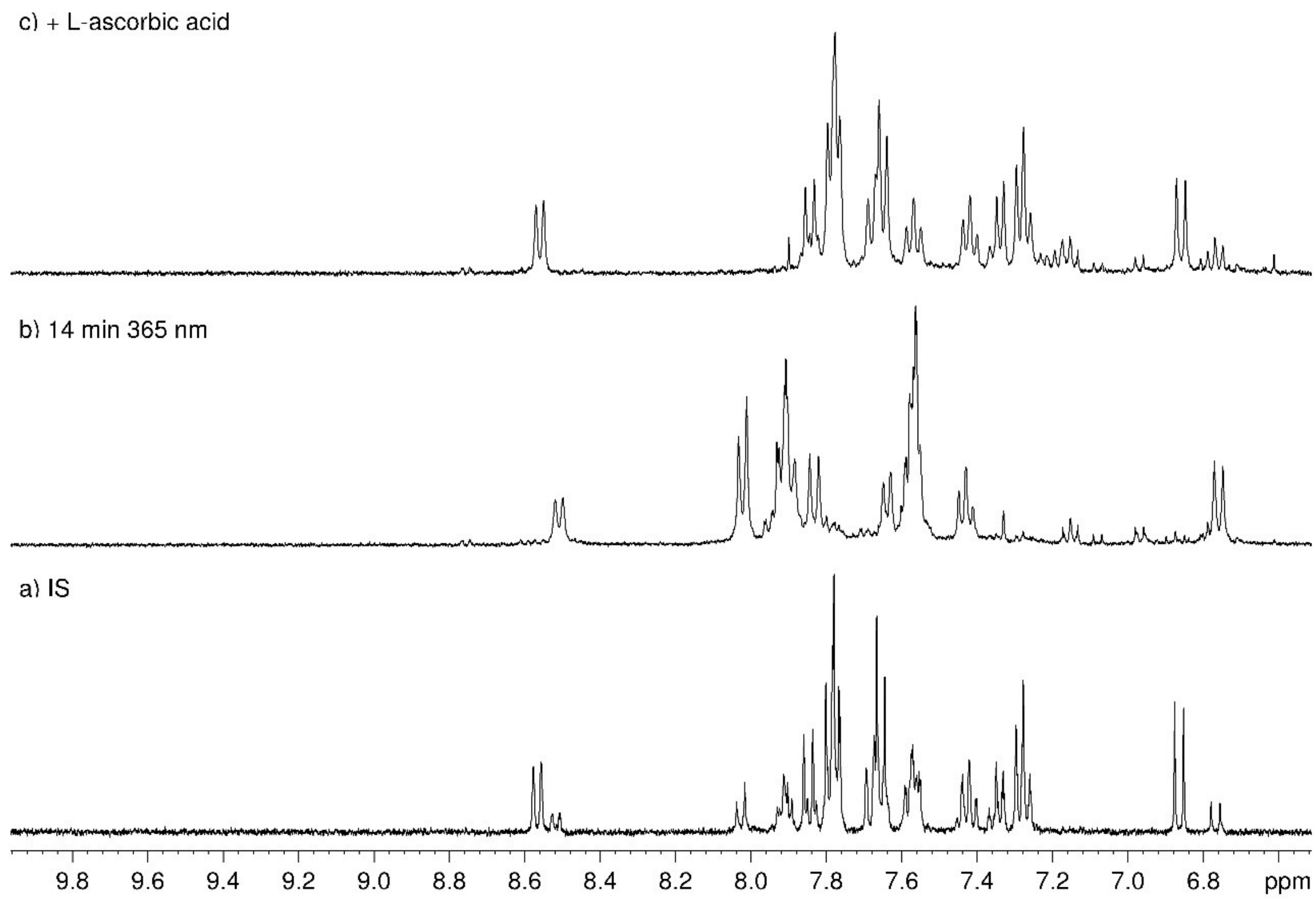

Figure S19. Section from the ^{1}H NMR spectra of the azoarene *p*-**2a**: a) after synthesis (initial state), b) after 14 minutes UV irradiation with λ = 365 nm, and c) after addition of L-ascorbic acid to the irradiated sample (MeOD, 400 MHz, c = 2.5 mM).

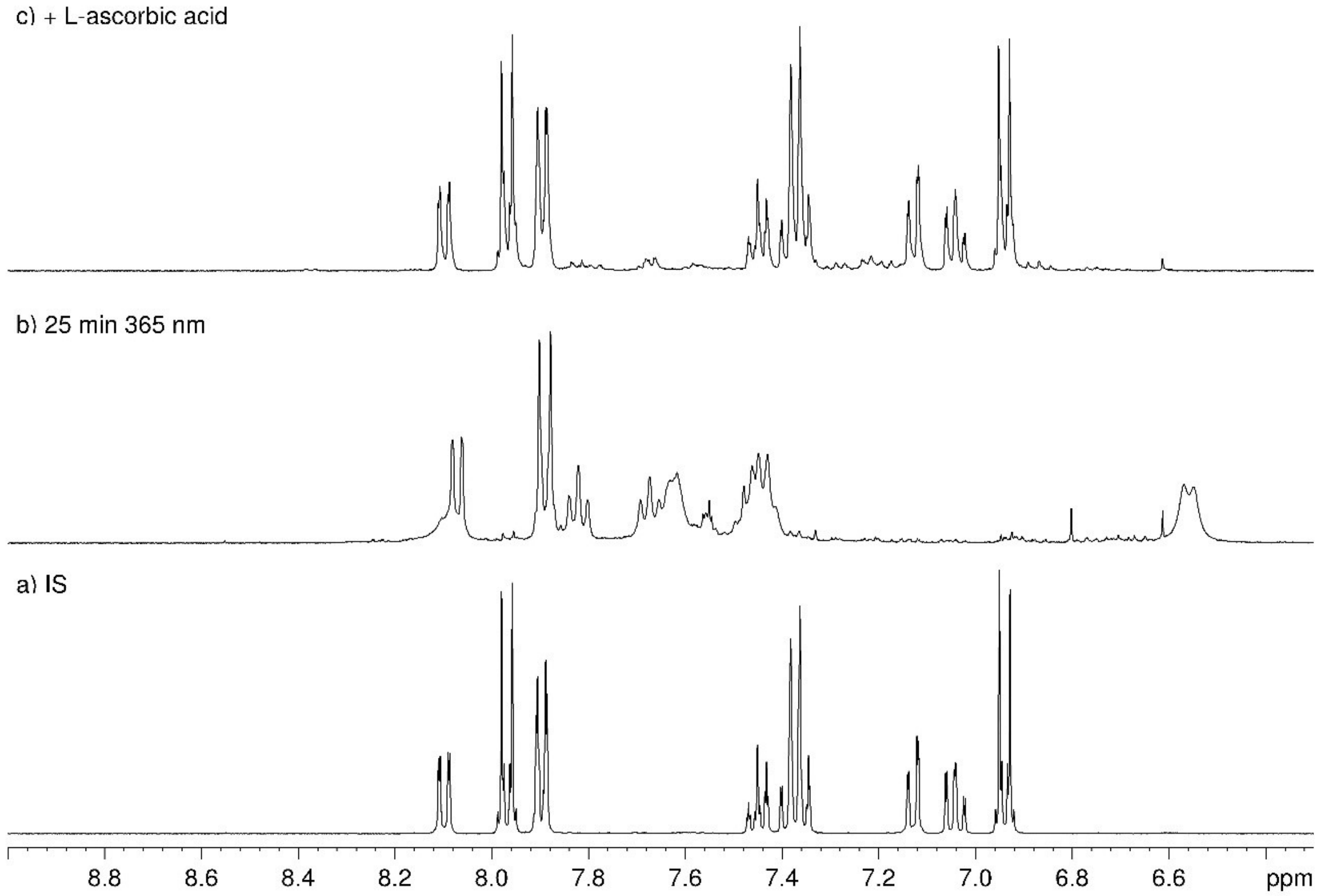

Figure S20. Section from the ^{1}H NMR spectra of the azoarene **4a**: a) after synthesis (initial state), b) after 25 minutes UV irradiation with λ = 365 nm, and c) after addition of L-ascorbic acid to the irradiated sample (MeOD, 400 MHz, c = 5 mM).

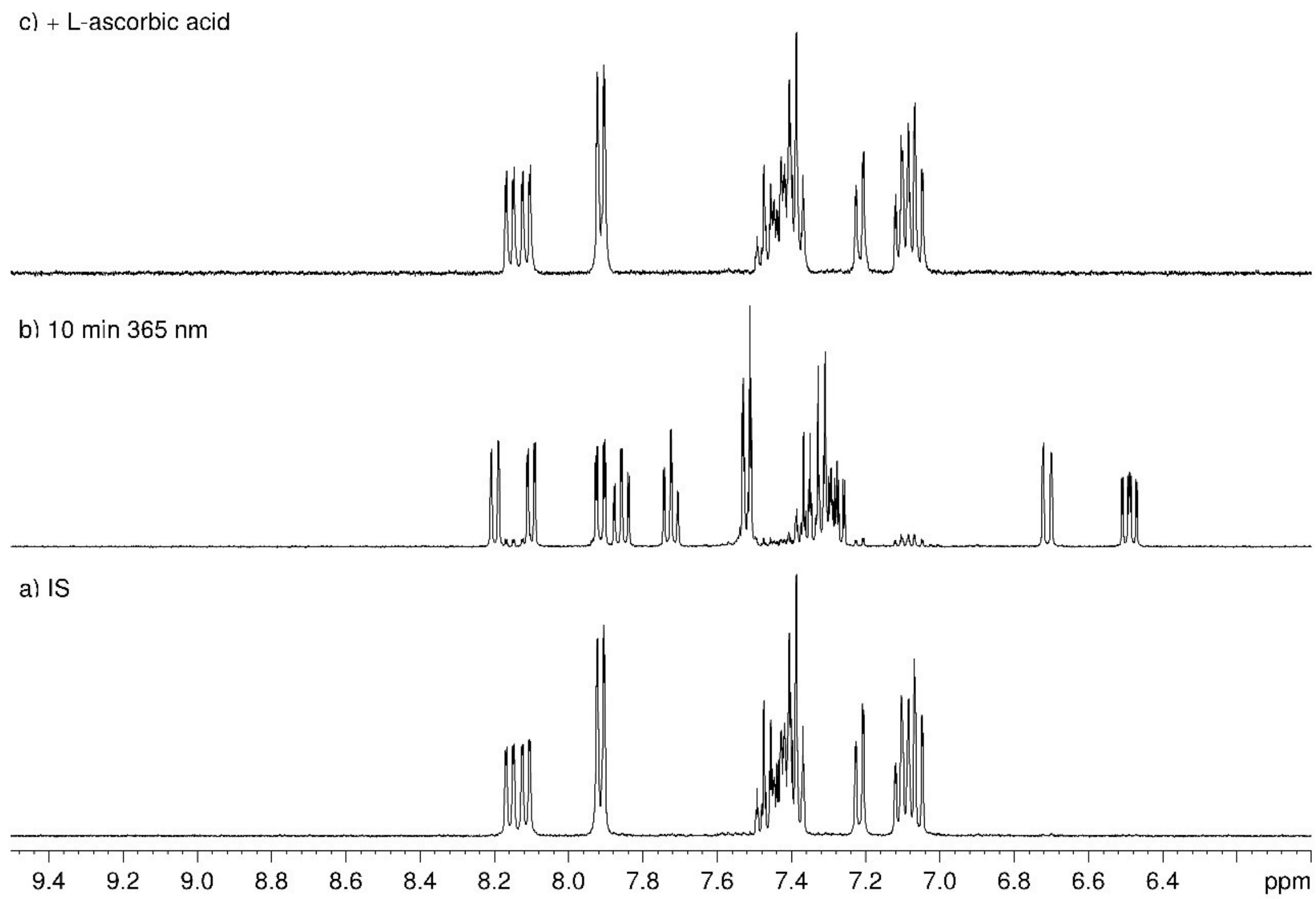

Figure S21. Section from the ^{1}H NMR spectra of the azoarene **3a**: a) after synthesis (initial state), b) after 10 minutes UV irradiation with λ = 365 nm, and c) after the addition of L-ascorbic acid to the irradiated sample (MeOD, 400 MHz, c = 5 mM).

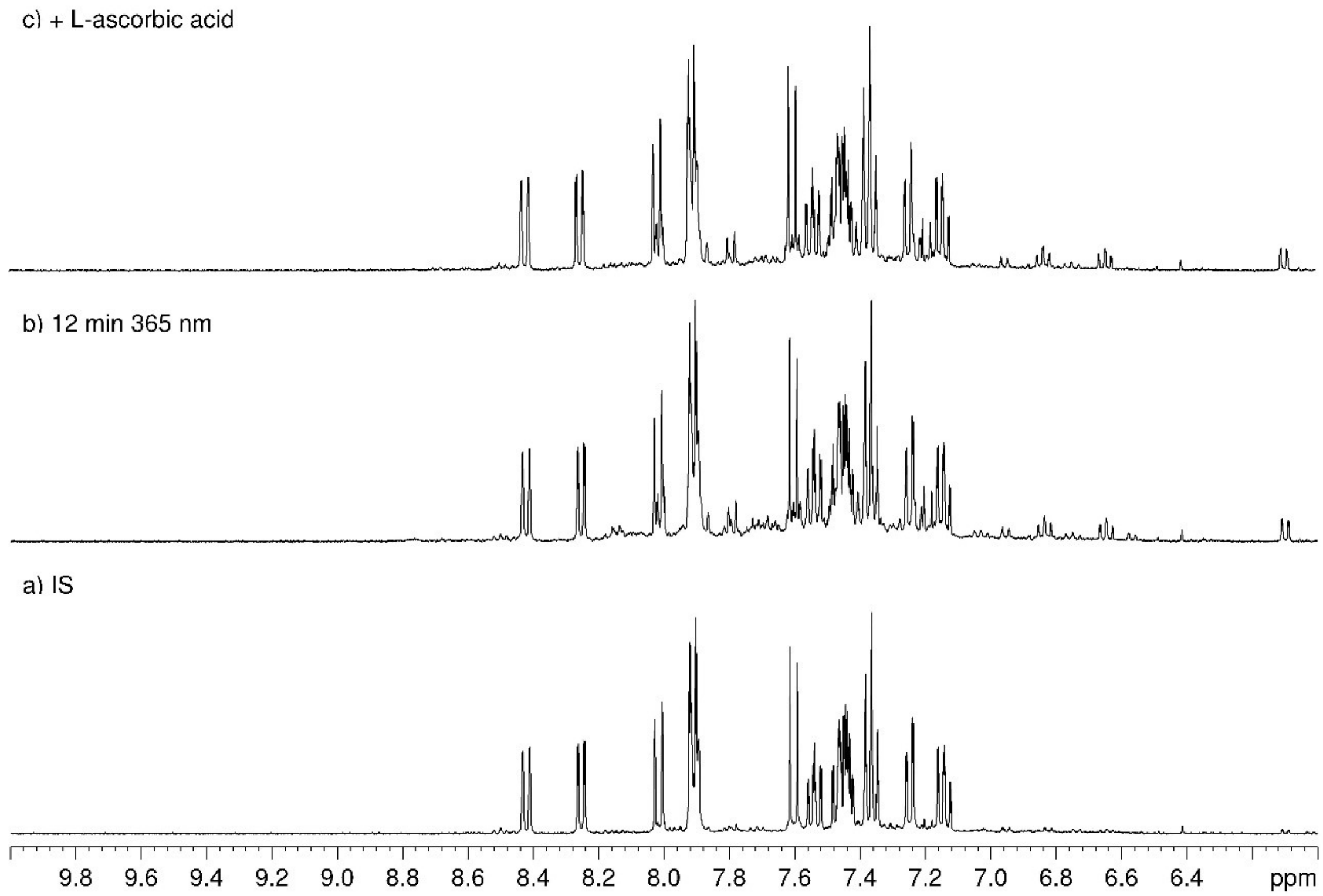

Figure S22. Section from the ^{1}H NMR spectra of the azoarene **5**: a) after synthesis (initial state) and b) after 12 minutes UV irradiation with λ = 365 nm and c) after the addition of L-ascorbic acid to the irradiated sample (MeOD, 400 MHz, c = 5 mM).

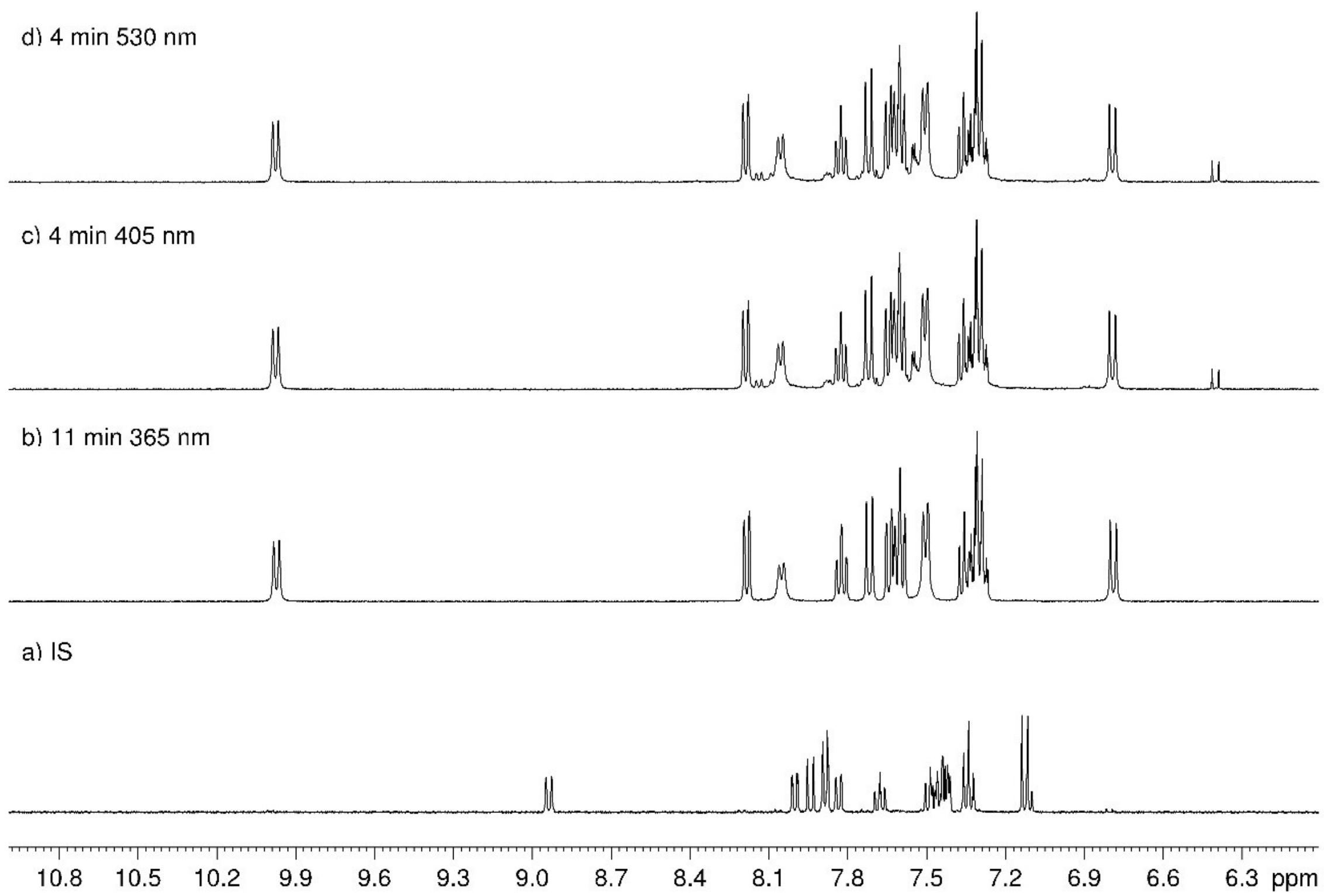

Figure S23. Section from the ^{1}H NMR spectra of the azoarene *o*-**2a**: a) after synthesis (initial state), b) after 11 minutes irradiation with λ = 365 nm, c) after additional 4 minutes irradiation with λ = 405 nm, and d) after additional 4 minutes irradiation with λ = 530 nm (MeOD, 400 MHz, c = 5 mM).

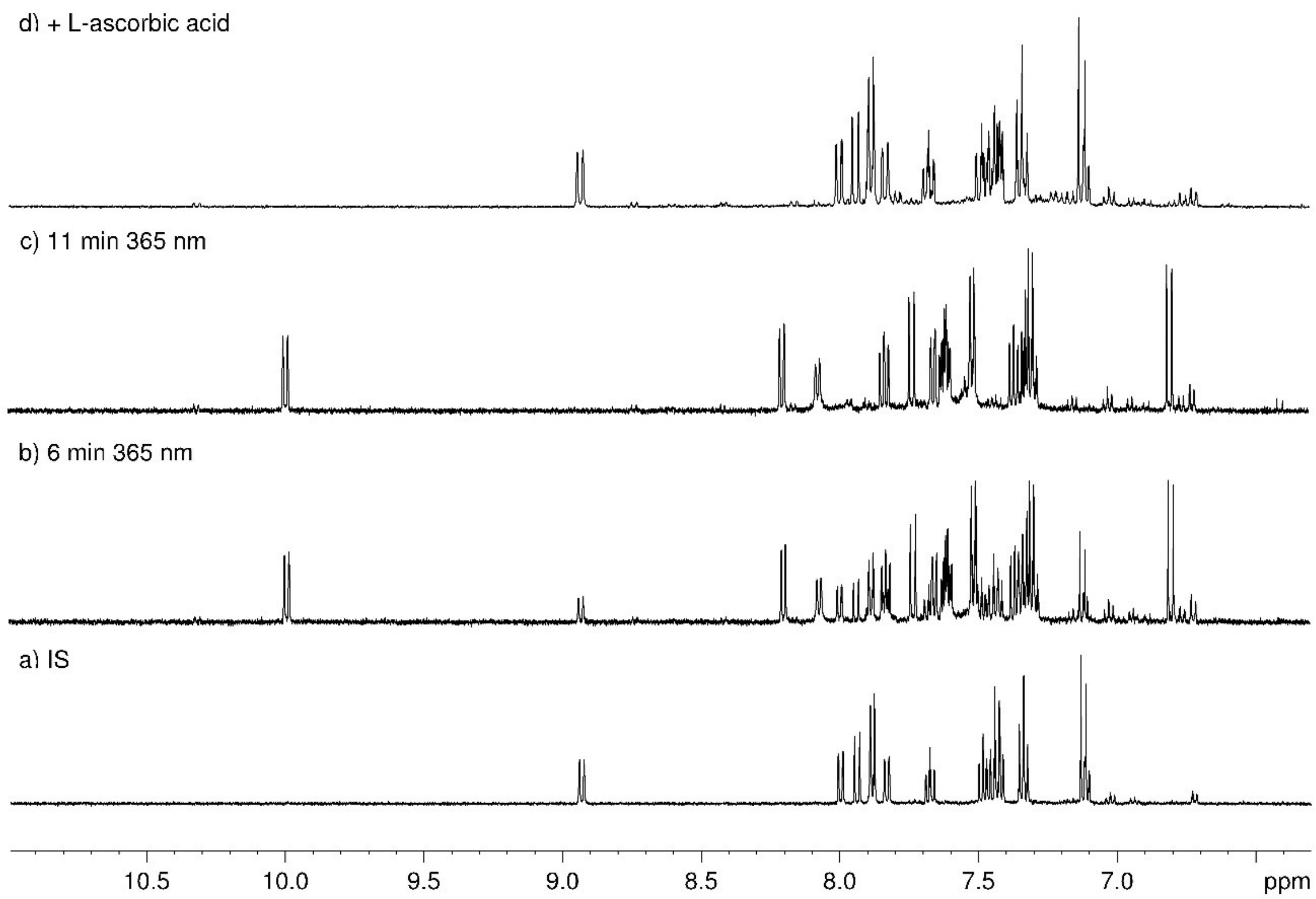

Figure S24. Section from the ^{1}H NMR spectra of the azoarene *o*-**2a**: a) after synthesis (initial state), b) after 6 and c) 11 minutes UV irradiation with λ = 365 nm, and d) after addition of L-ascorbic acid to the irradiated sample (MeOD, 400 MHz, c = 2.5 mM).

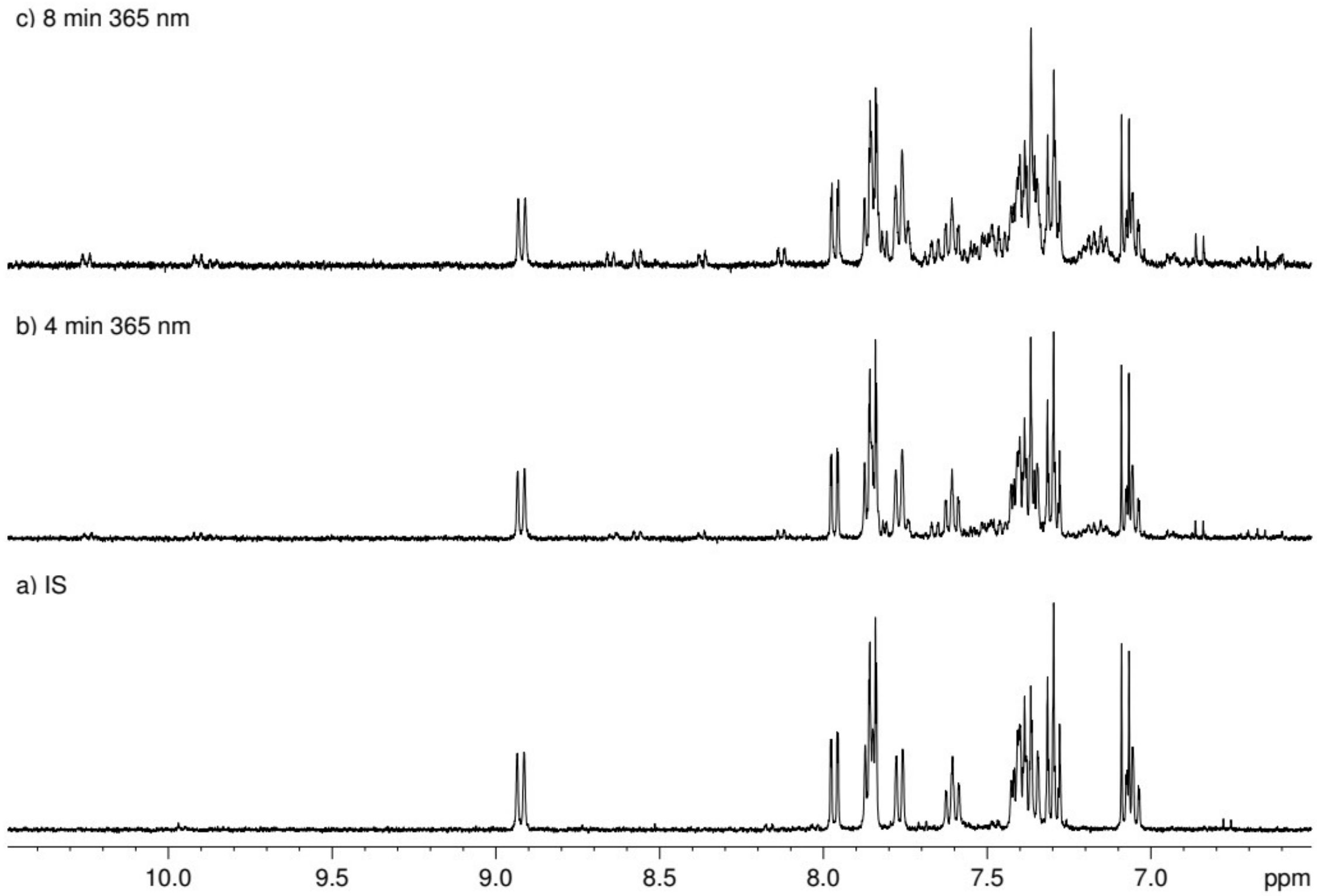

Figure S25. Section from the ^{1}H NMR spectra of the azoarene *o*-**2a** under oxygen exclusion: a) after synthesis (initial state), b) after 4, and c) after 8 minutes UV irradiation with λ = 365 nm (MeOD, 400 MHz, c = 5 mM).

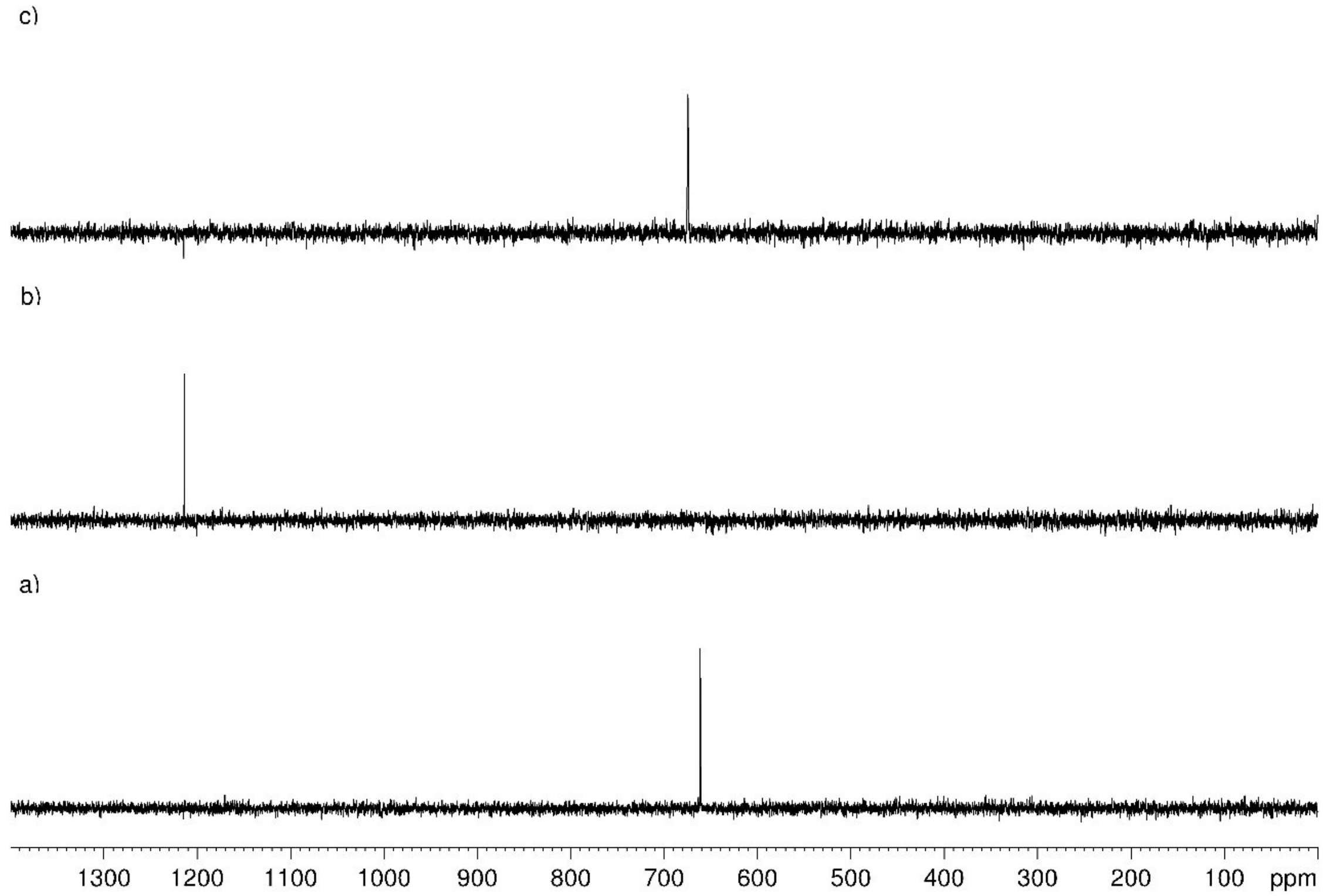

Figure S26. Section from the ^{125}Te NMR spectra of the azoarene *o*-**2a**: a) after synthesis, b) after 11 minutes irradiation with λ = 365 nm, and c) after deprotonation with 2 equivalents KO*t*-Bu (MeOD, 189 MHz, c = 5 mM).

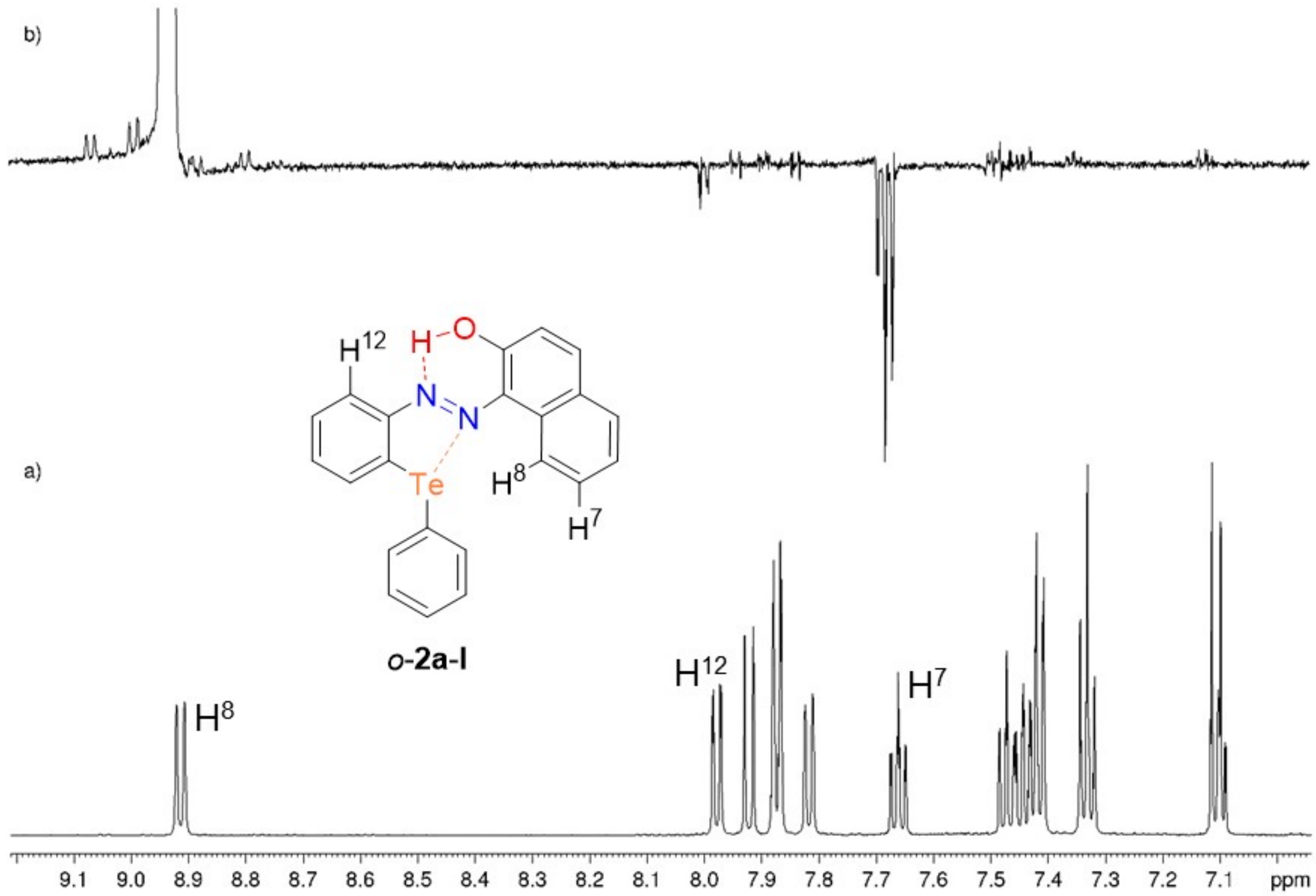

Figure S27. Section from the ^{1}H NMR spectrum (b) of the azoarene *o*-**2a** and 1D NOESY spectrum (a) with excitation of the proton H^{8} with a chemical shift of 8.9 ppm (MeOD, 600 MHz, c = 5 mM).

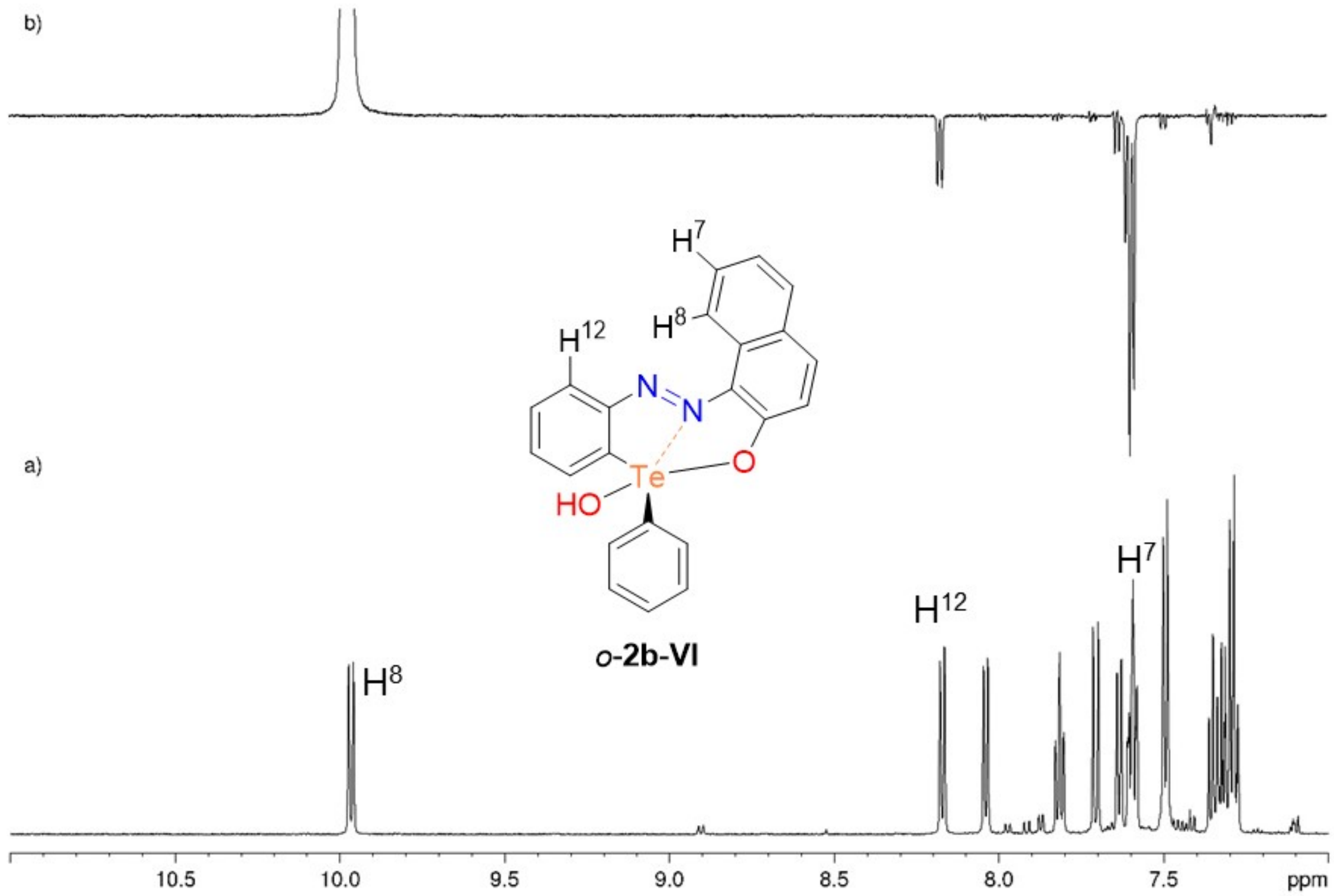

Figure S28. Section from the ^{1}H NMR spectrum (b) of the azoarene *o*-**2b** and 1D NOESY spectrum (a) with excitation of the proton H^8 with a chemical shift of 10.0 ppm (MeOD, 600 MHz, c = 5 mM).

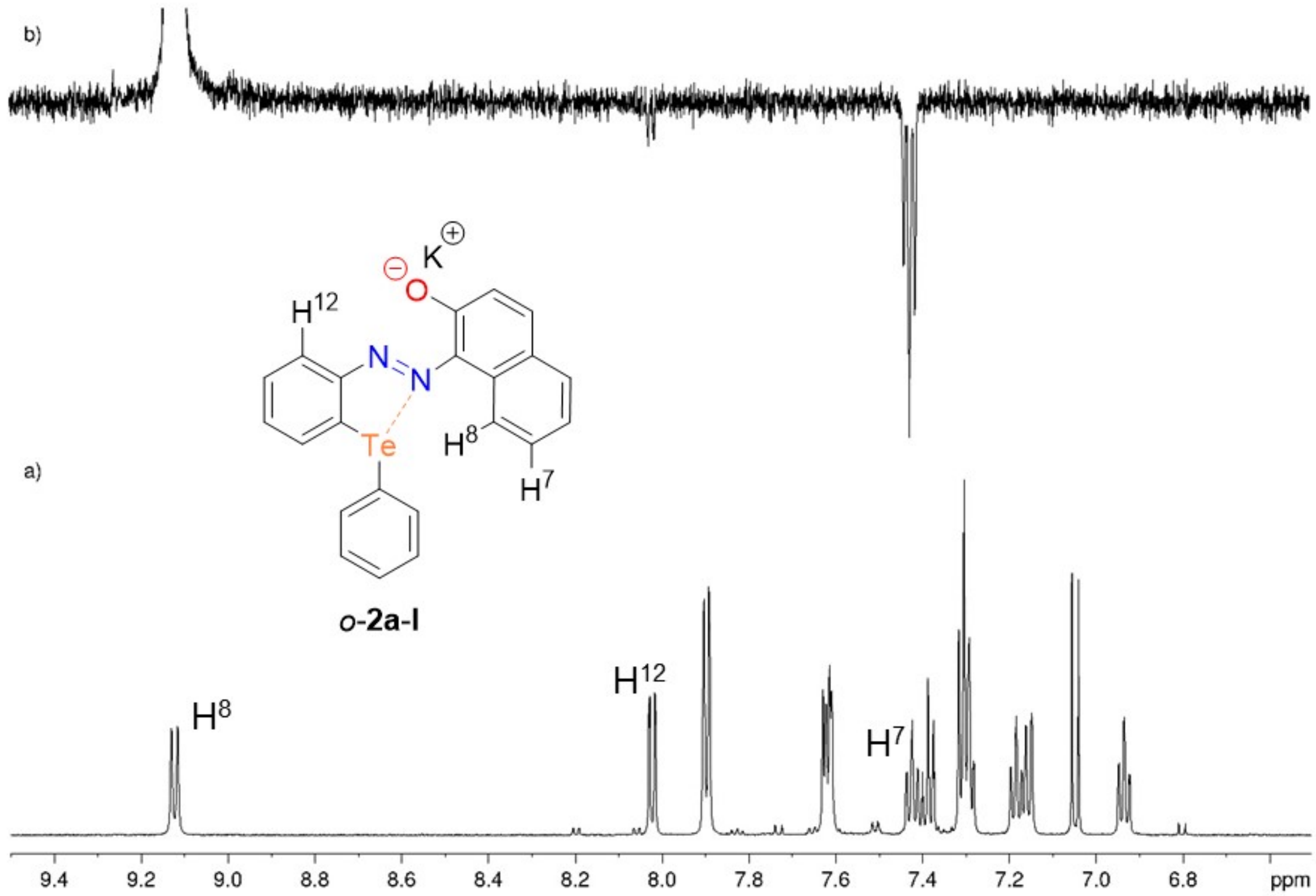

Figure S29. Section from the ^{1}H NMR spectrum of the azoarene *o*-**2a** with 2 equivalents KO*t*-Bu (a) and 1D NOESY spectrum (b) with excitation of the proton H^{8} with a chemical shift of 9.1 ppm. (MeOD, 600 MHz, c = 5 mM).

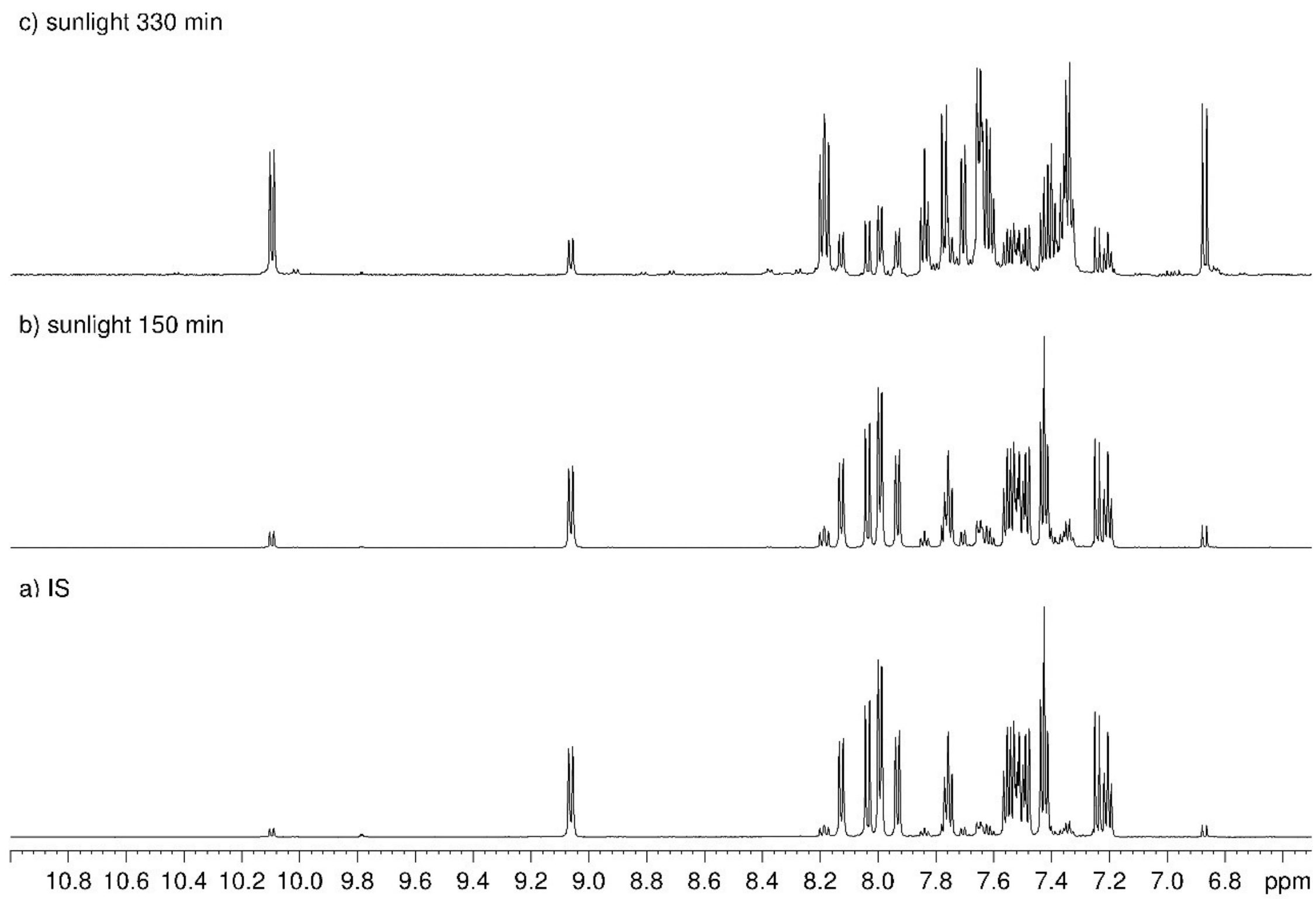

Figure S30. Section from the ^{1}H NMR spectra of the azoarene *o*-**2a**: a) after synthesis (initial state), b) after 2.5 h, and c) 5.5 h irradiation with sunlight (10% MeOD + 90 % ethyl acetate, 600 MHz, c = 5 mM).

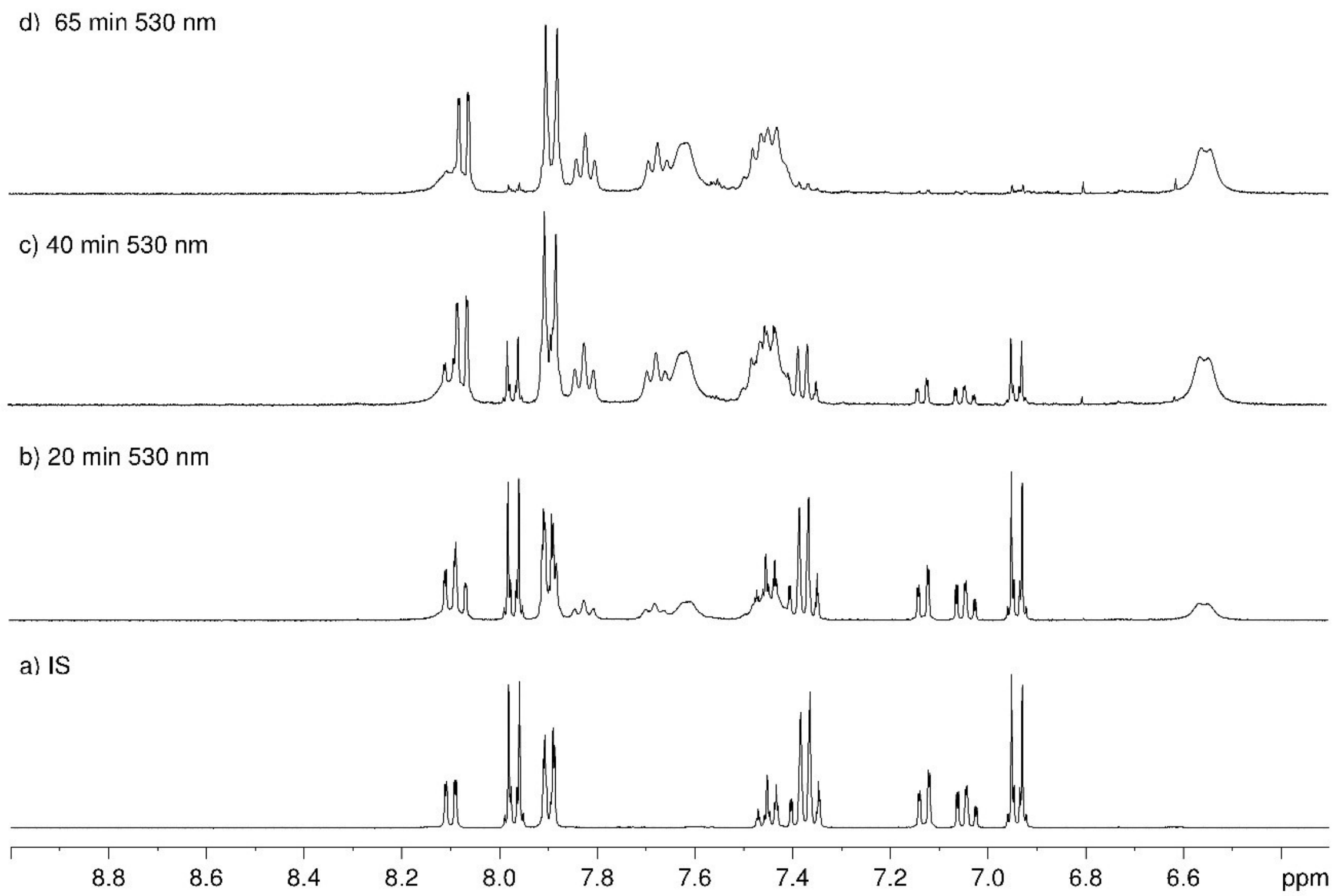

Figure S31. Section from the ^{1}H NMR spectra of the azoarene **4a**: a) after synthesis (initial state), b) after 20, c) 40, and d) 65 minutes irradiation with λ = 530 nm (MeOD, 400 MHz, c = 5 mM).

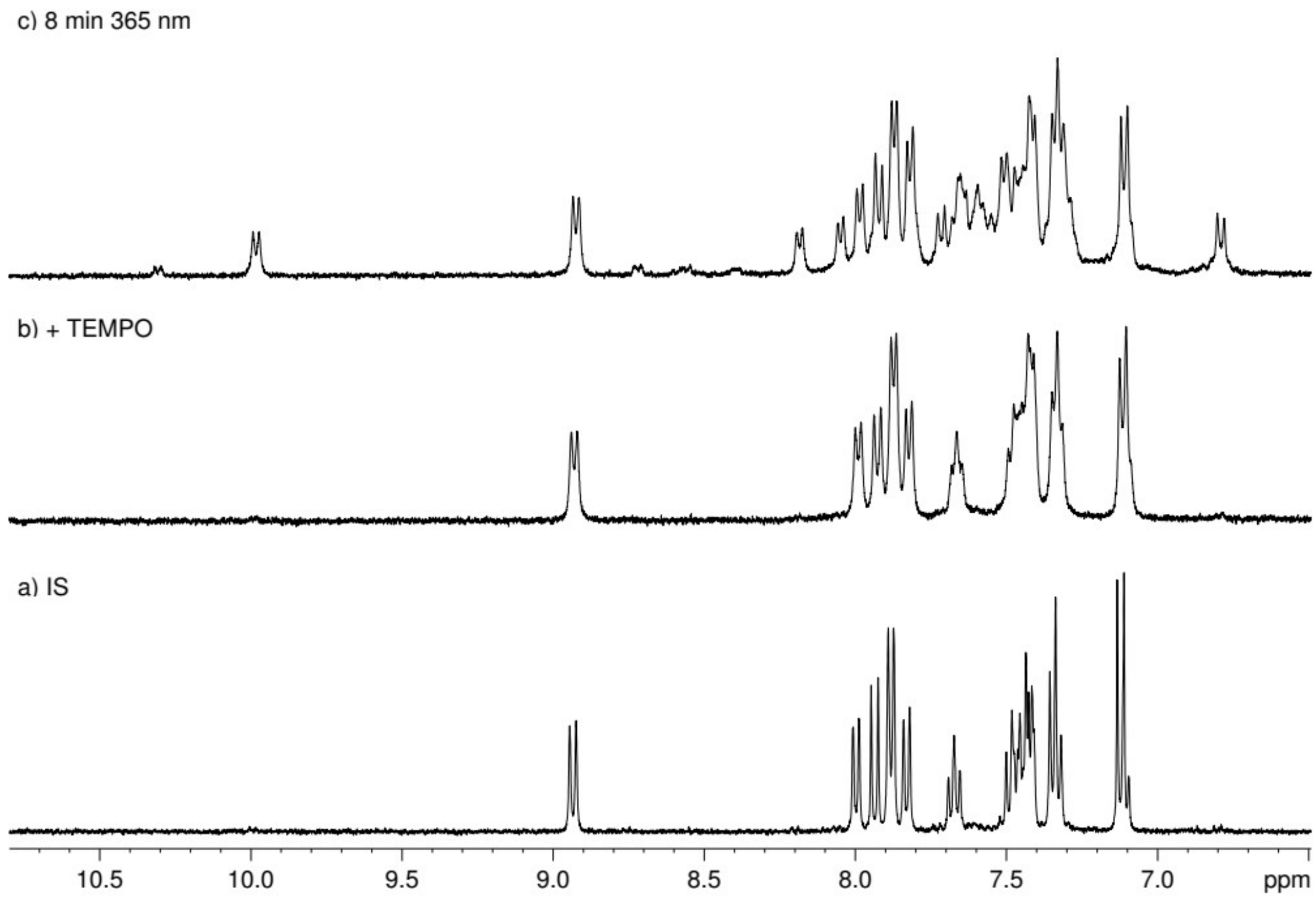

Figure S32. Section from the ^{1}H NMR spectra (MeOD, 400 MHz, c = 5 mM) of the azoarene *o*-**2a**: a) after synthesis (initial state), b) after addition of 2 eq TEMPO, and c) 8 minutes UV irradiation of b) with λ = 365 nm. The photoproduct to starting material ratio is 1:2 after 8 minutes of irradiation.

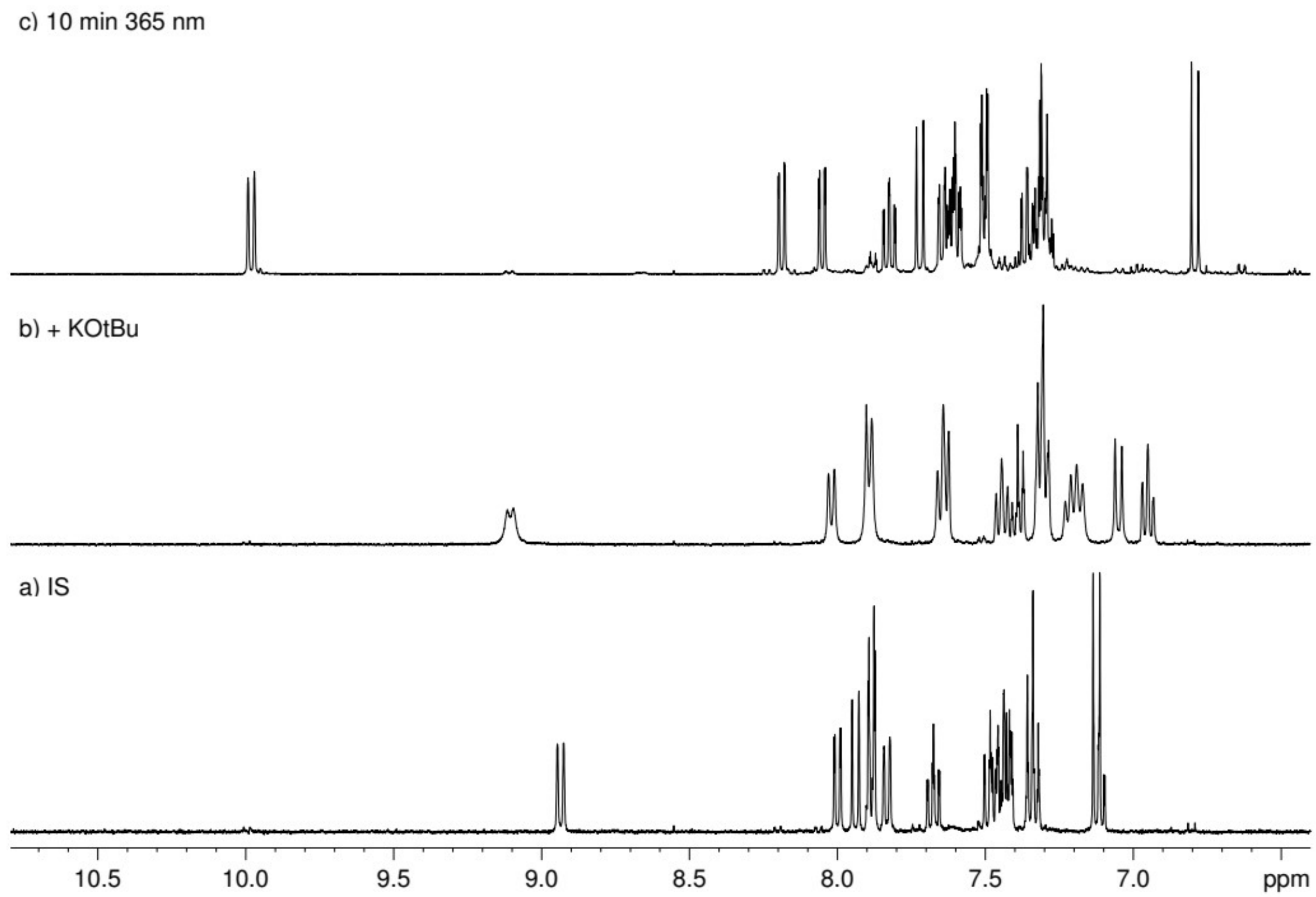

Figure S33. Section from the ^{1}H NMR spectra of the azoarene *o*-**2a**: a) after synthesis (initial state), b) after addition of 2 eq potassium KO*t*-Bu, and c) after 10 minutes UV irradiation of b) with λ = 365 nm (MeOD, 400 MHz, c = 5 mM).

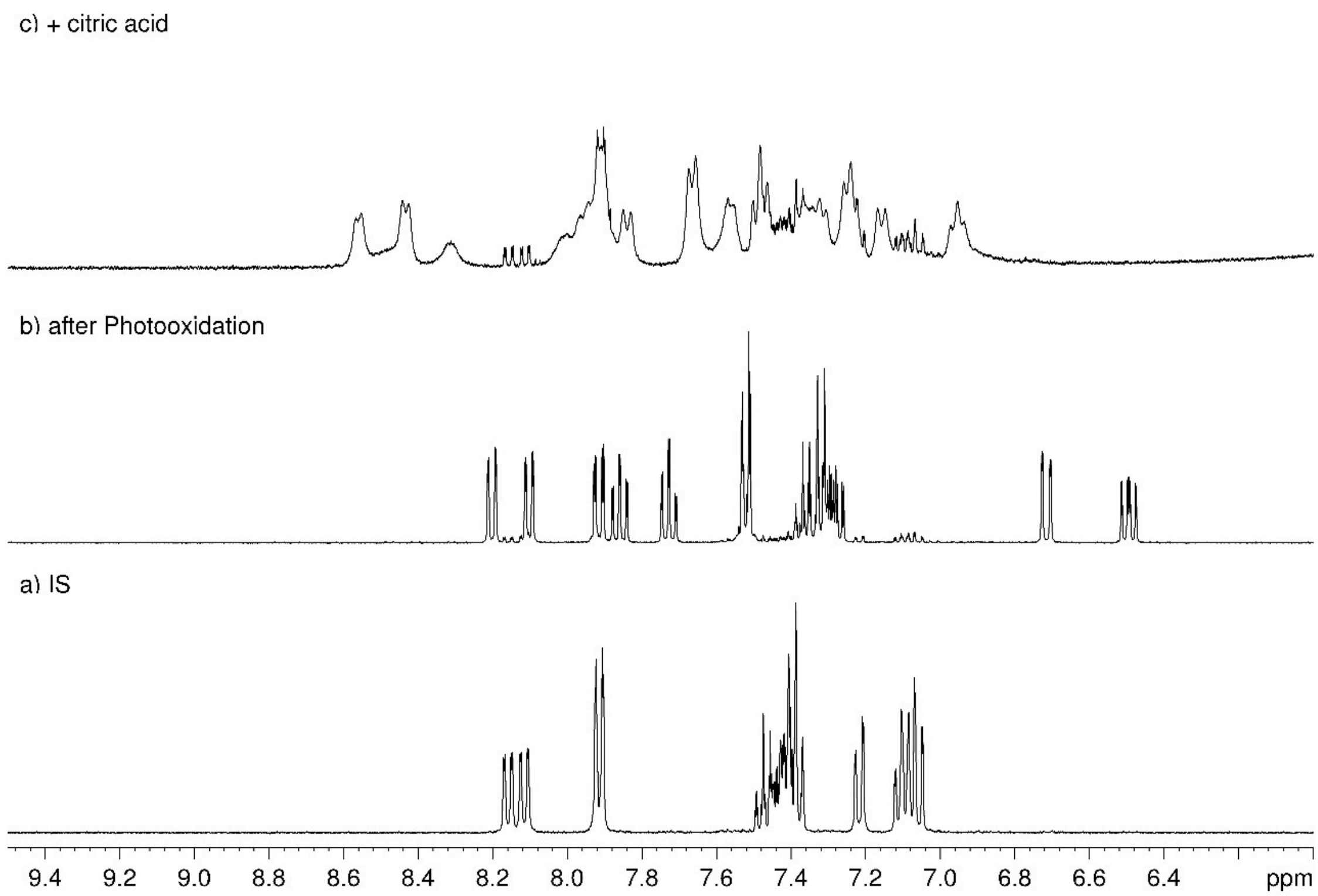

Figure S34. Section from the ^{1}H NMR spectra of the azoarene **3a**: a) after synthesis (initial state), b) after UV irradiation with λ = 365 nm, and c) after addition of citric acid to the irradiated sample (MeOD, 400 MHz, c = 5 mM).

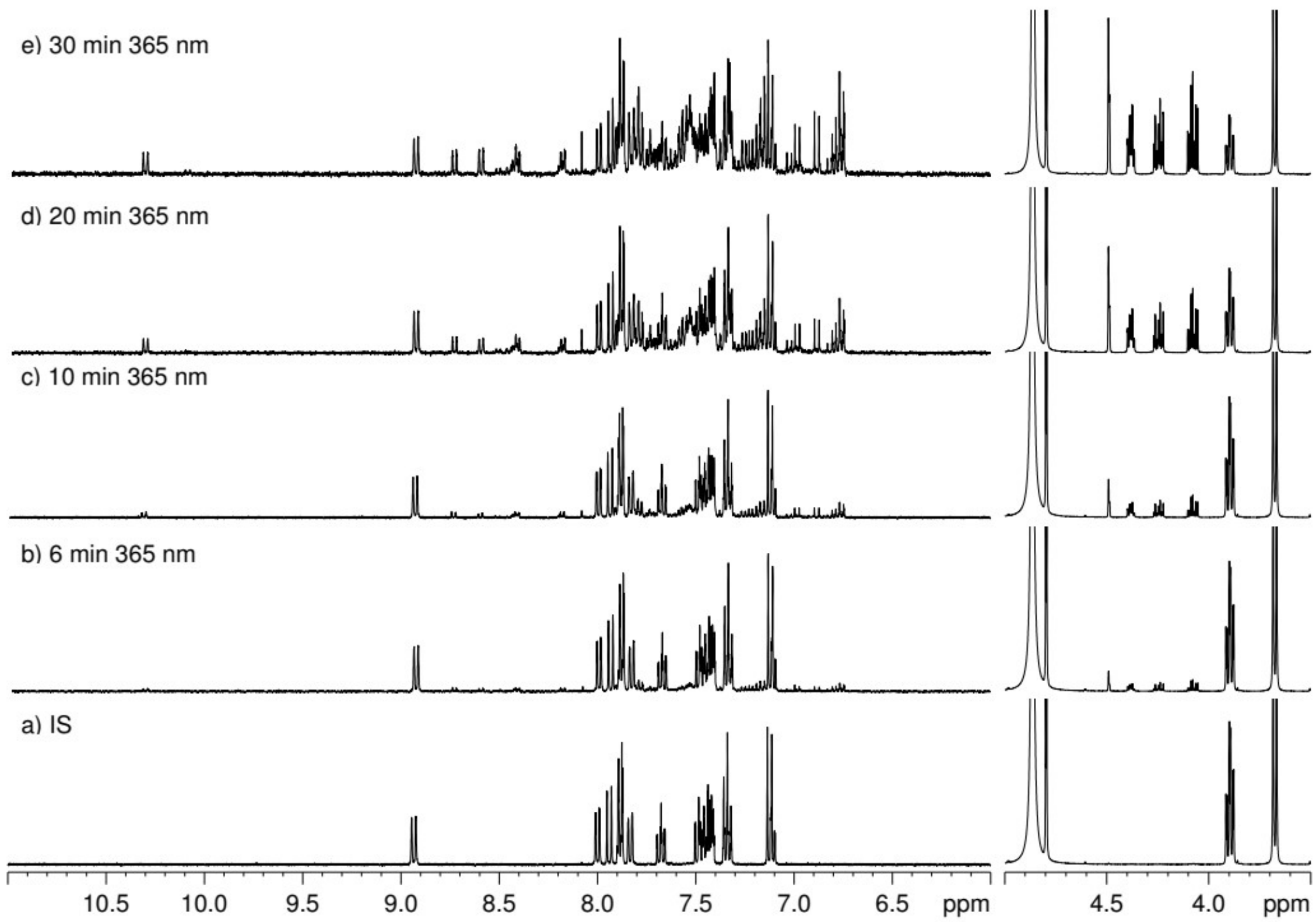

Figure S35. Section from the ^{1}H NMR spectra of the azoarene *o*-**2a** and 10 eq L-ascorbic acid: a) after synthesis (initial state), b) after 6, c) 10, d) 20, and e) 30 minutes UV irradiation with λ = 365 nm (MeOD, 400 MHz, c = 2.5 mM).

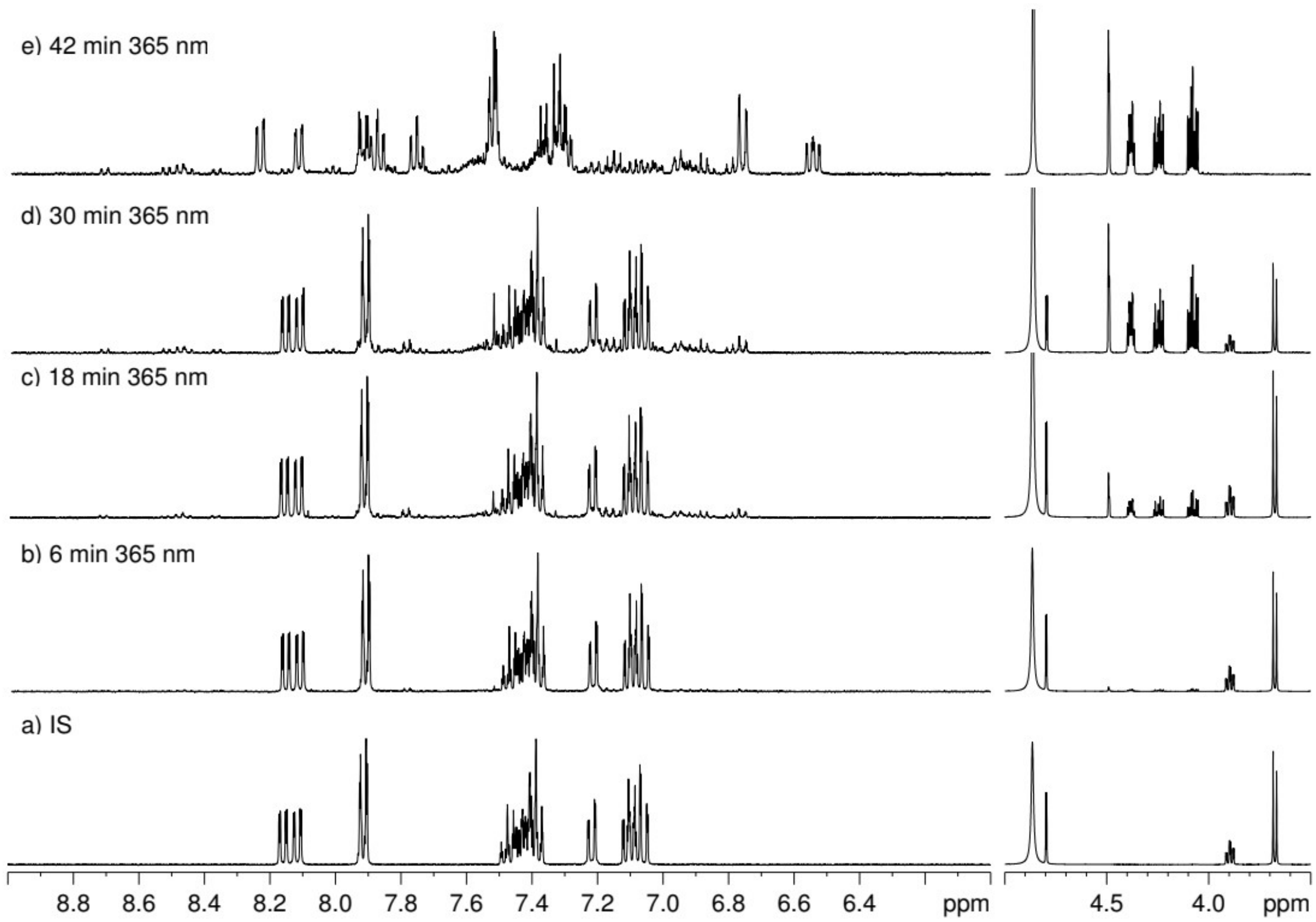

Figure S36. Section from the ^{1}H NMR spectra of the azoarene **3a** and 10 eq L-ascorbic acid: a) after synthesis (initial state), b) after 6, c) 18, d) 30, and e) 42 minutes UV irradiation with λ = 365 nm (MeOD, 400 MHz, c = 2.5 mM).

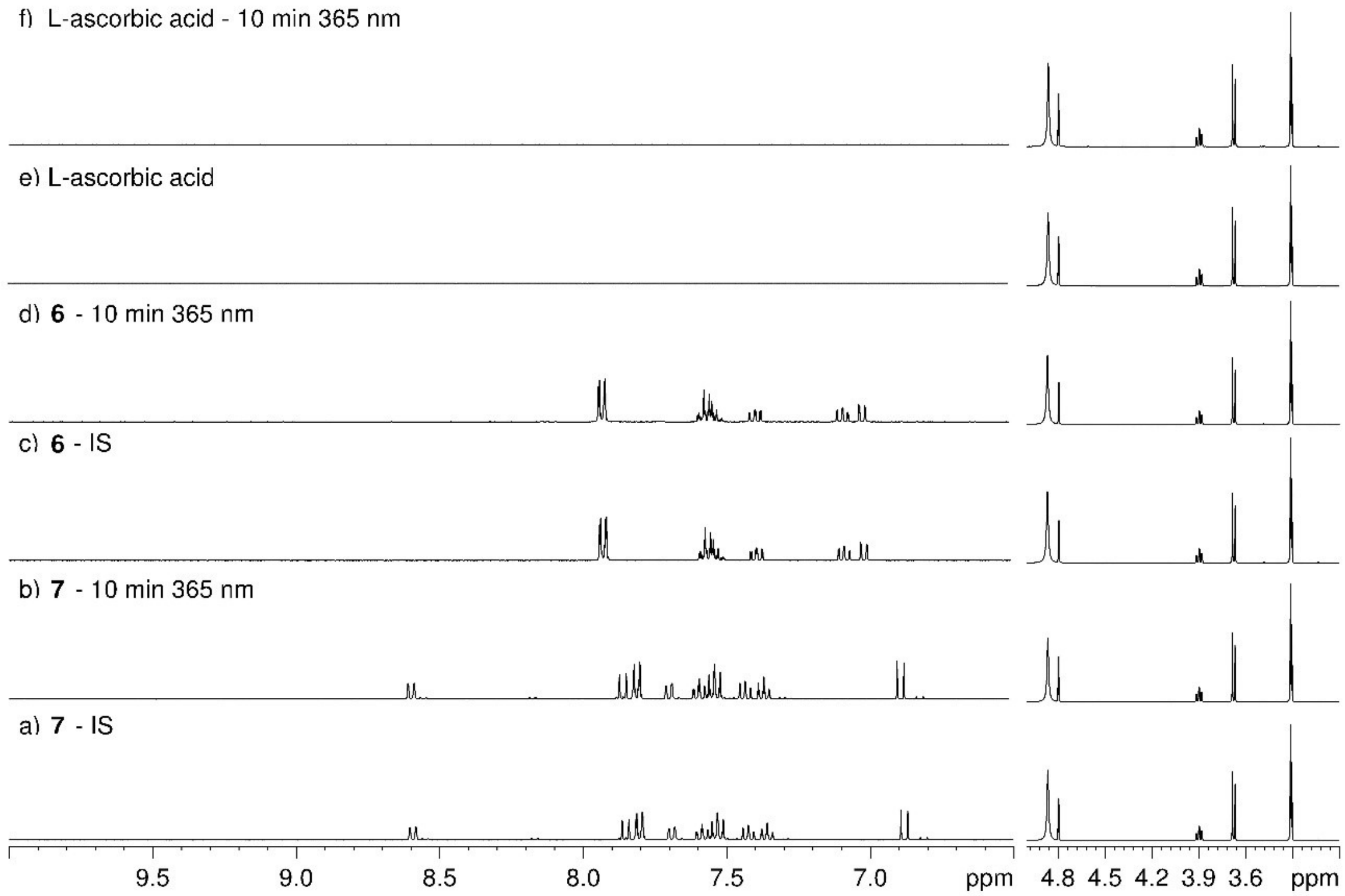

Figure S37. Section from the ^1H NMR spectra of the azoarene **7** and L-ascorbic acid: a) before and b) after irradiation with light of the wavelength λ = 365 nm. Section from the ^1H NMR spectra of the azoarene **6** and L-ascorbic acid: c) before and d) after irradiation with light of the wavelength λ = 365 nm. Section from the ^1H NMR spectra of L-ascorbic acid: e) before and f) after irradiation with light of the wavelength λ = 365 nm (MeOD, 400 MHz, c = 5 mM).

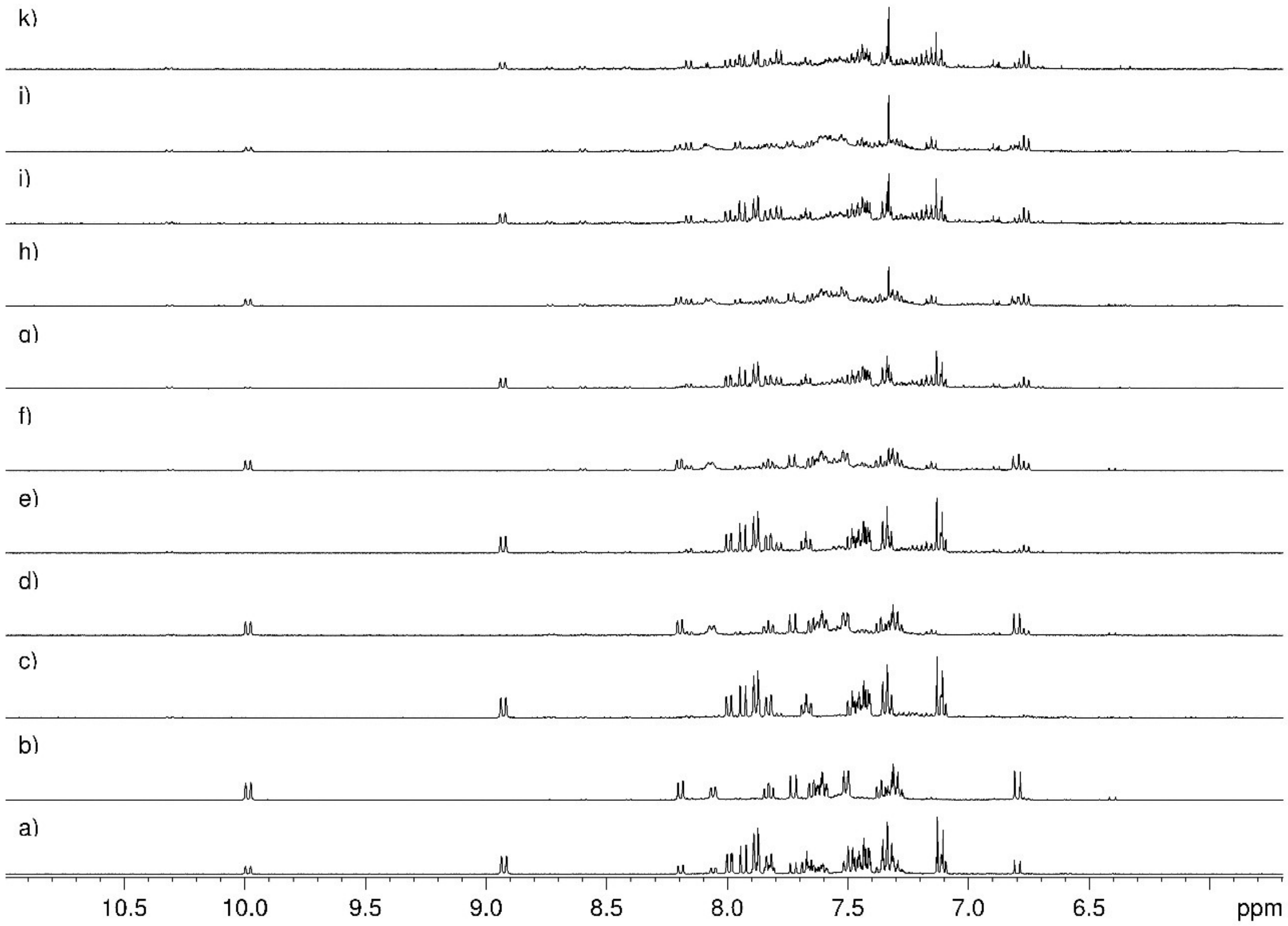

Figure S38. Section from the ^{1}H NMR spectra of the azoarene *o*-**2a** after multiple irradiation/reduction cycles: a) after synthesis (initial state), b)/d)/f)/h)/j) after UV irradiation with λ = 365 nm, c)/e)/g)/i)/k) after addition of L-ascorbic acid (MeOD, 400 MHz, c = 2.5 mM).

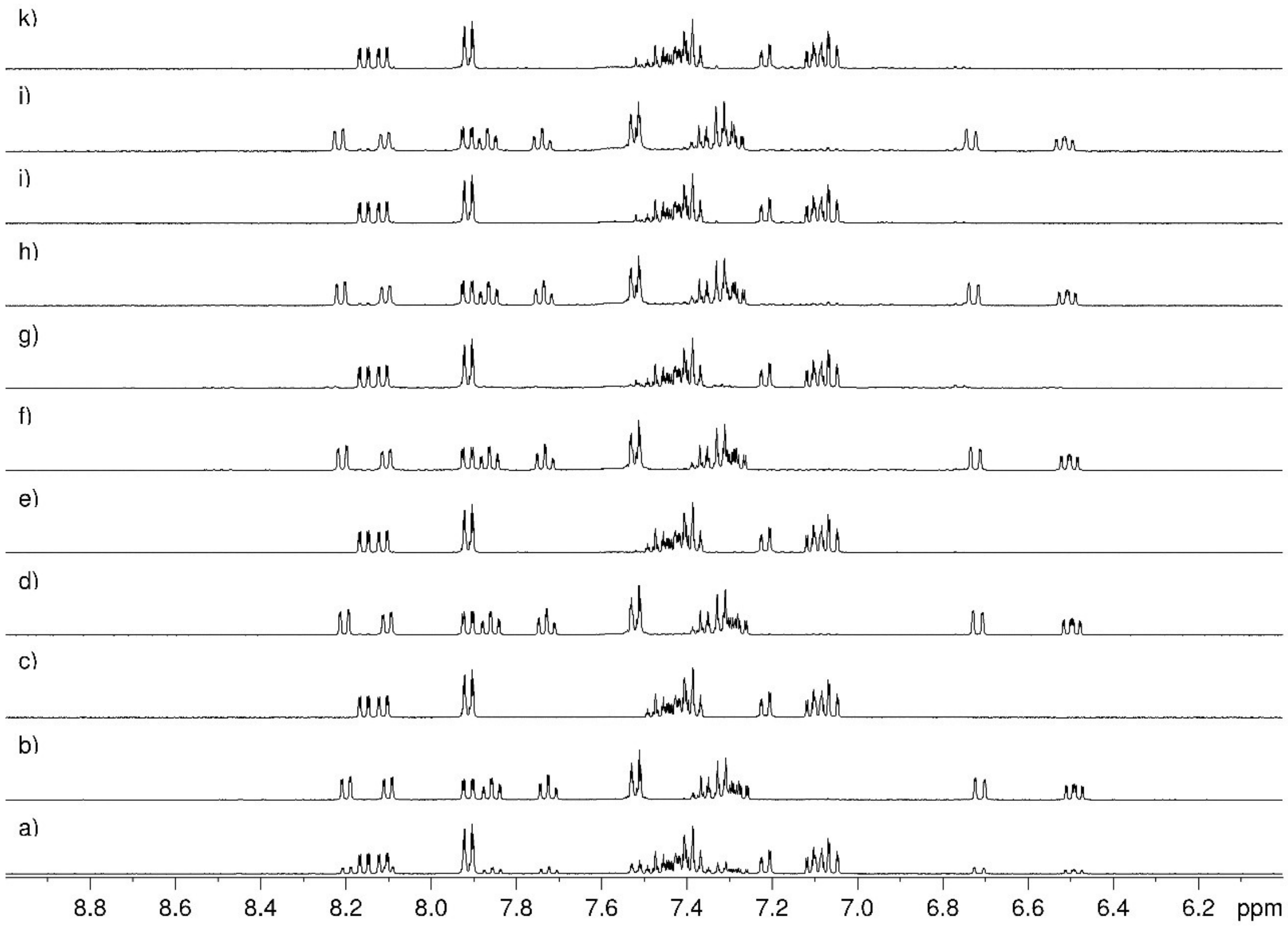

Figure S39. Section from the ^{1}H NMR spectra of the azoarene **3a** after multiple irradiation/reduction cycles: a) after synthesis (initial state), b)/d)/f)/h)/j) after UV irradiation with λ = 365 nm, c)/e)/g)/i)/k) after addition of L-ascorbic acid (MeOD, 400 MHz, c = 2.5 mM).

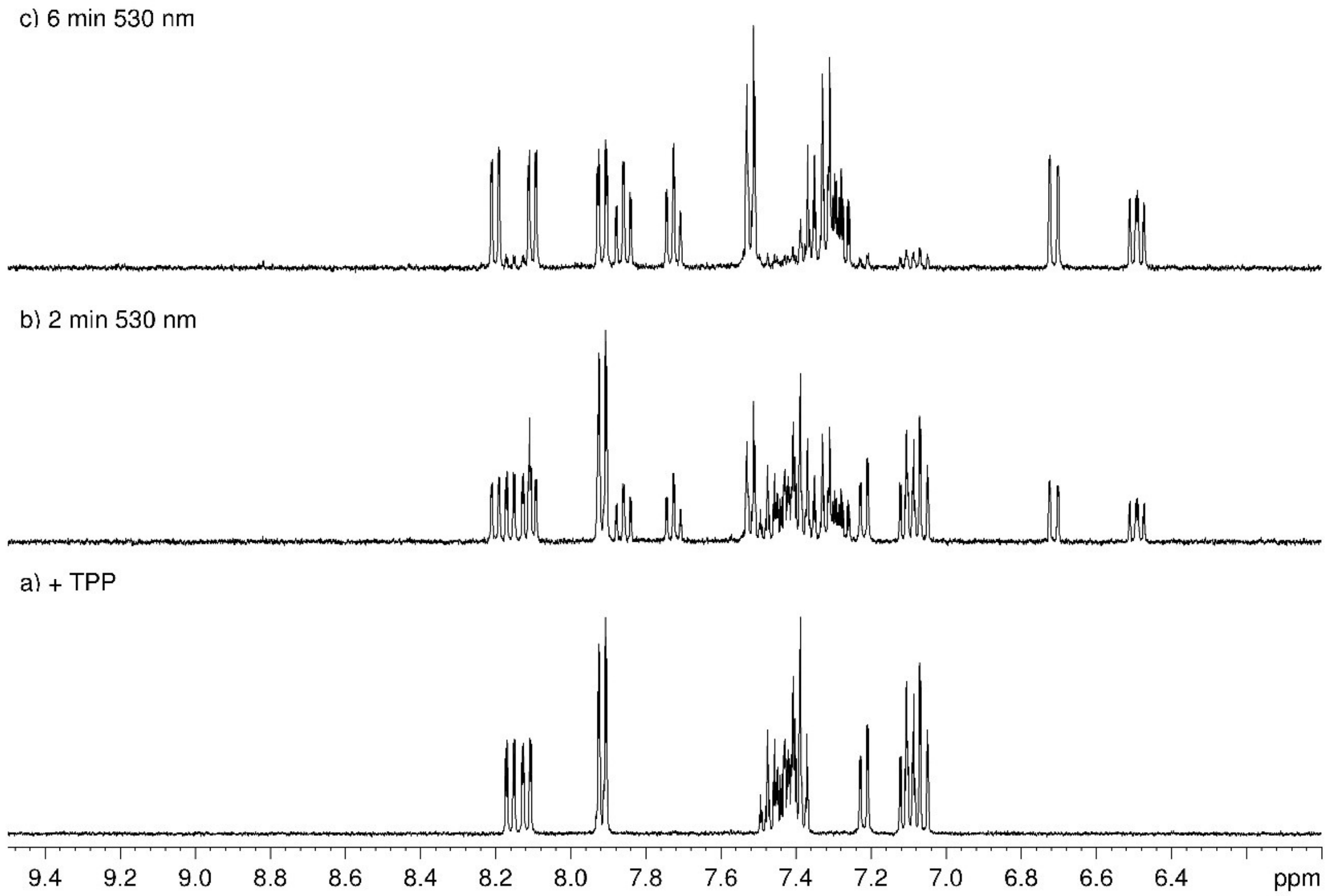

Figure S40. Section from the ^{1}H NMR spectra of the azoarene **3a** and 1 mol% TPP (tetraphenylporphyrin): a) initial state, b) after 2, and c) 6 minutes irradiation with λ = 530 nm (MeOD, 400 MHz, c = 2.5 mM).

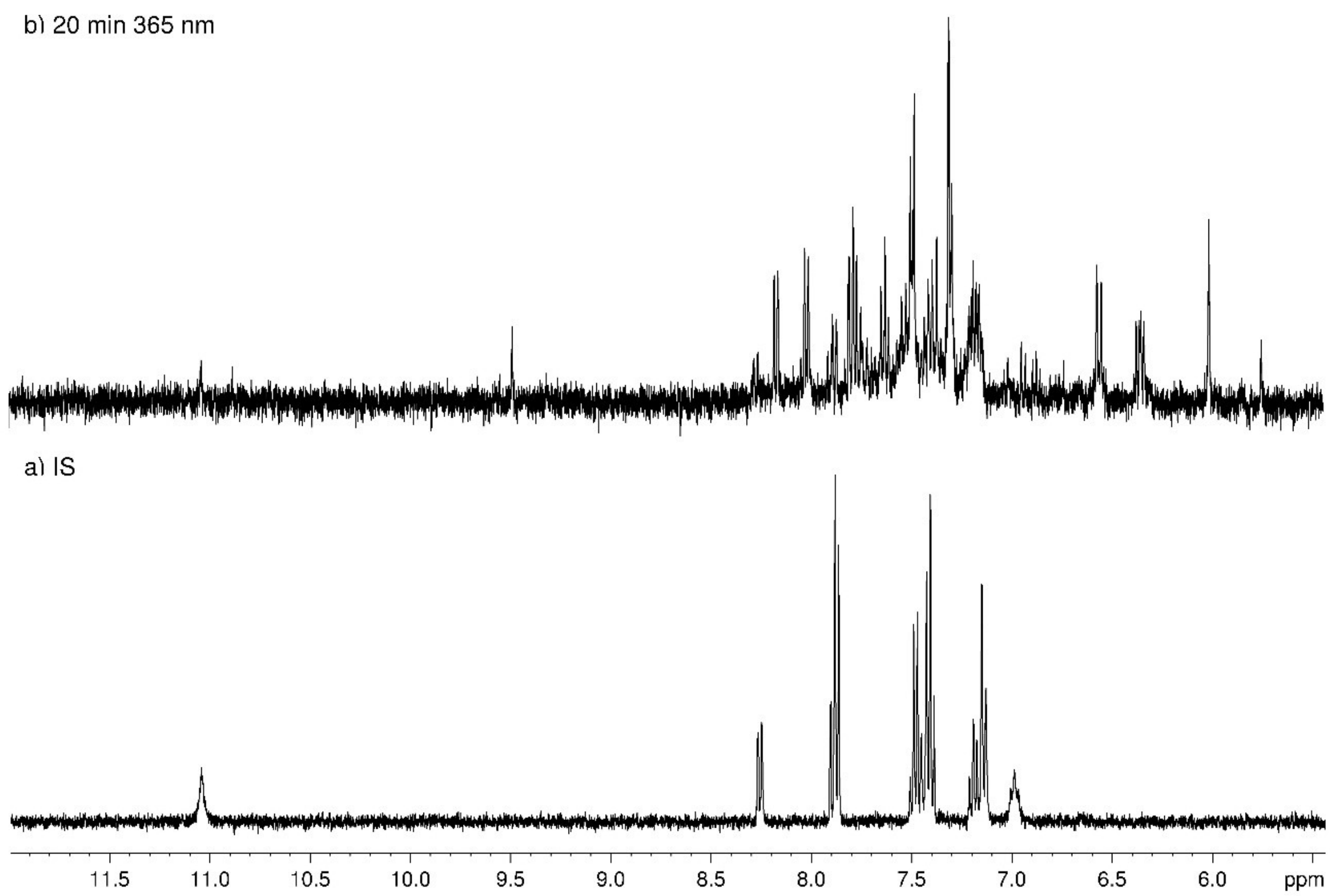

Figure S41. Section from the ^{1}H NMR spectra of the azoarene **3a**: a) after synthesis (initial state) and b) after 20 minutes UV irradiation with λ = 365 nm (DMSO-*d6*, 400 MHz, c = 1.5 mM).

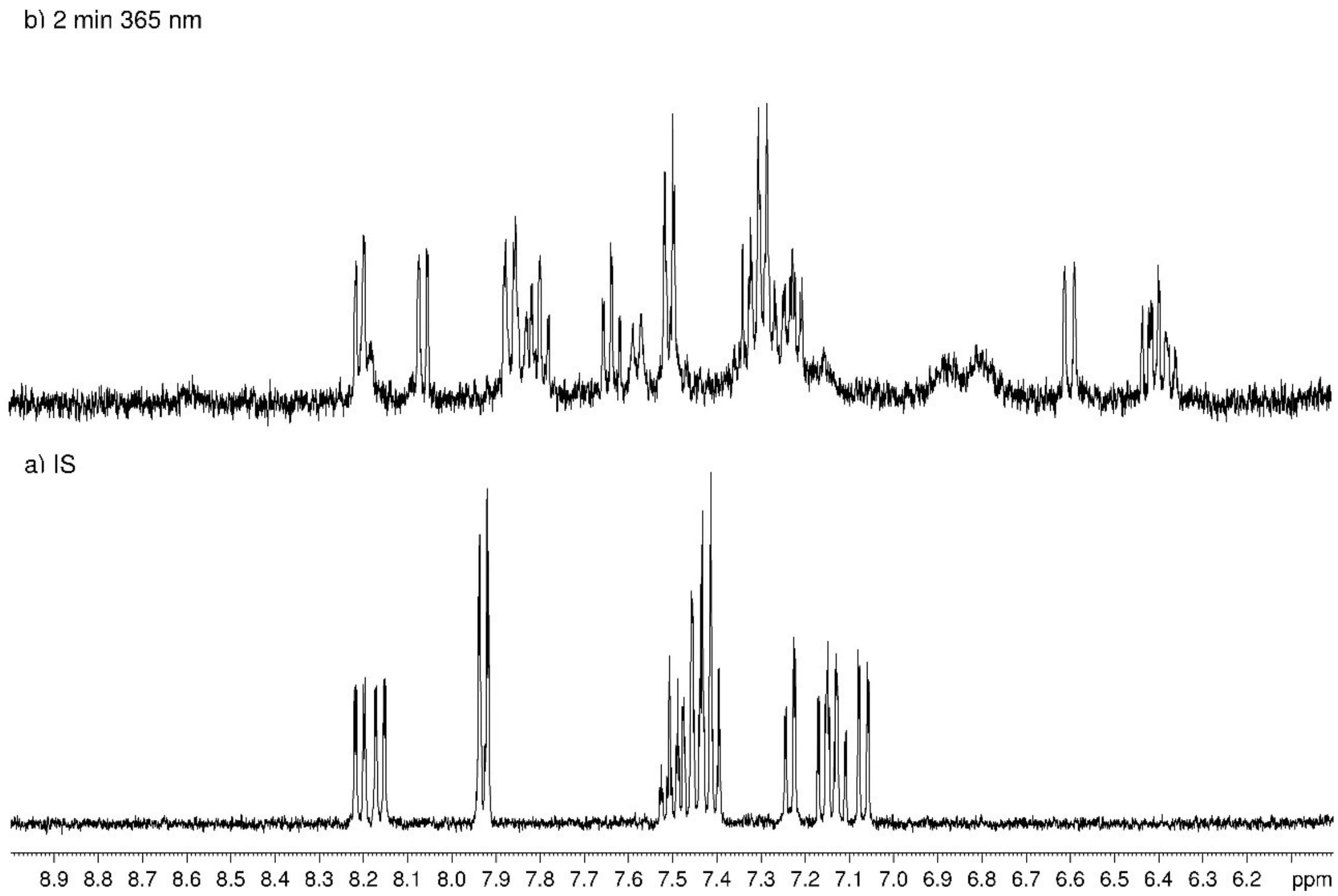

Figure S42. Section from the ^{1}H NMR spectra of the azoarene **3a**: a) after synthesis (initial state) and b) after 2 minutes UV irradiation with λ = 365 nm (MeCN-*d3*, 400 MHz, c = 1.5 mM).

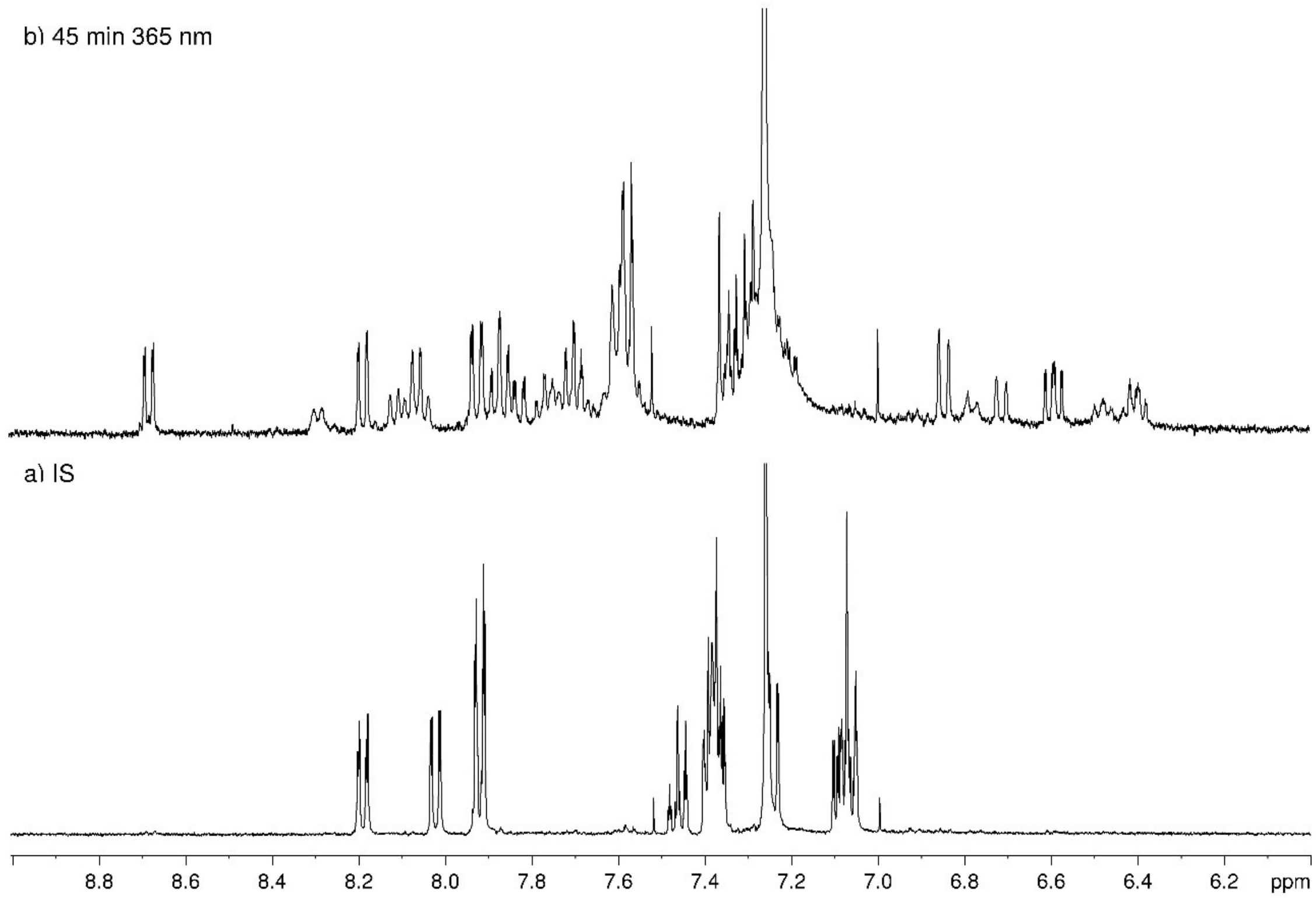

Figure S43. Section from the ^{1}H NMR spectra of the azoarene **3a**: a) after synthesis (initial state) and b) after 45 minutes UV irradiation with λ = 365 nm ($CDCl_3$, 400 MHz, c = 5 mM).

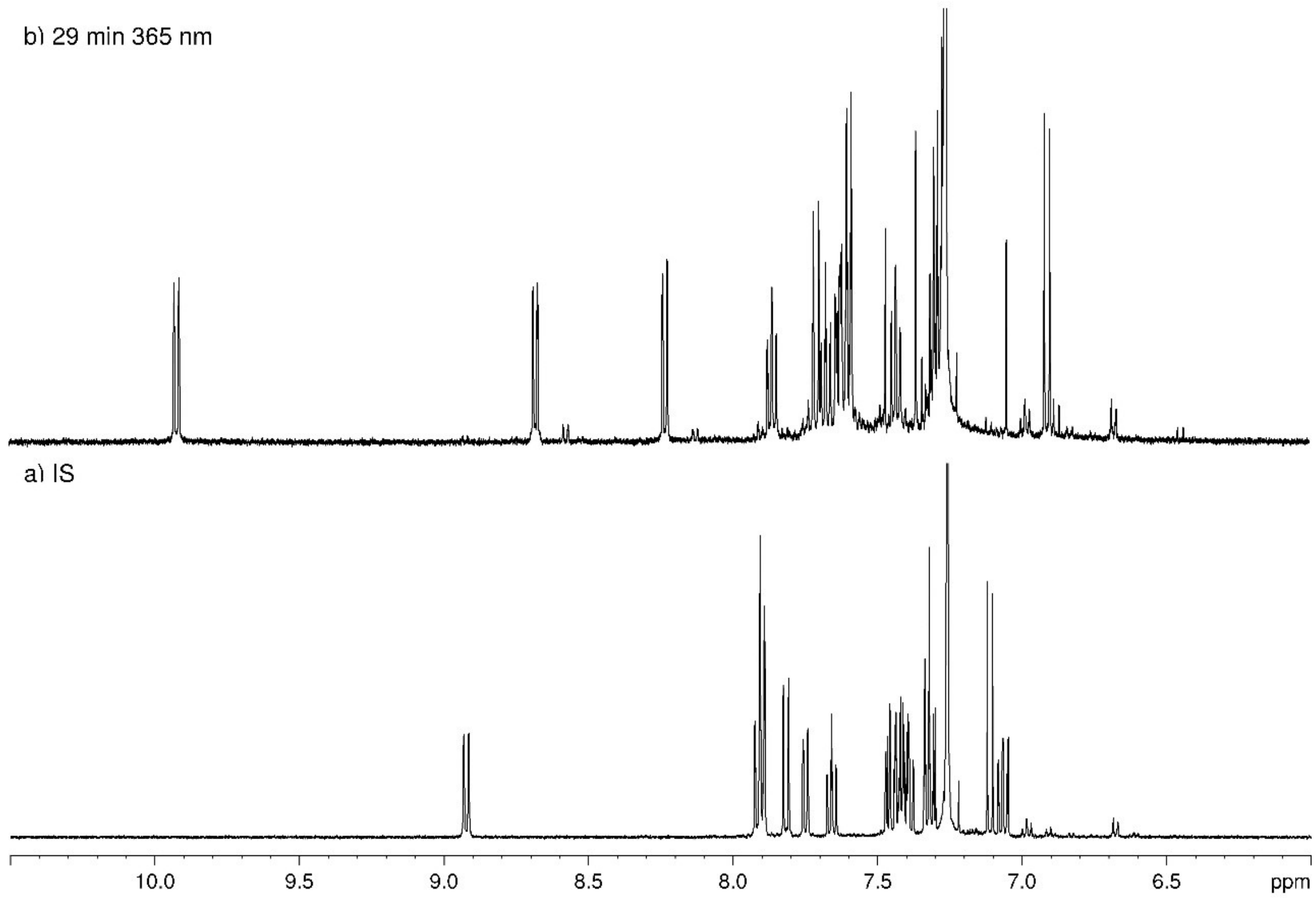

Figure S44. Section from the ^{1}H NMR spectra of the azoarene *o*-**2a**: a) after synthesis (initial state) and b) after 29 minutes UV irradiation with λ = 365 nm ($CDCl_3$, 400 MHz, c = 5 mM).

2. Fluorescence Spectra

Absorption spectra of the compounds were obtained with a Jasco V-550 spectrophotometer in methanol at a concentration of 1 mM using a 1 mm quartz glass cuvette. Emission spectra were recorded on an RF-6000 spectrometer from Shimadzu Corporation using 490 nm as excitation wavelength. The solid samples were placed between two quartz glass slides at a 45° angle to the excitation and emission slits. All measurements were conducted at room temperature. Absolute photoluminescence quantum yields were measured with the stand-alone absolute PL quantum yield measurement system C11347-11 by Quantaurus.

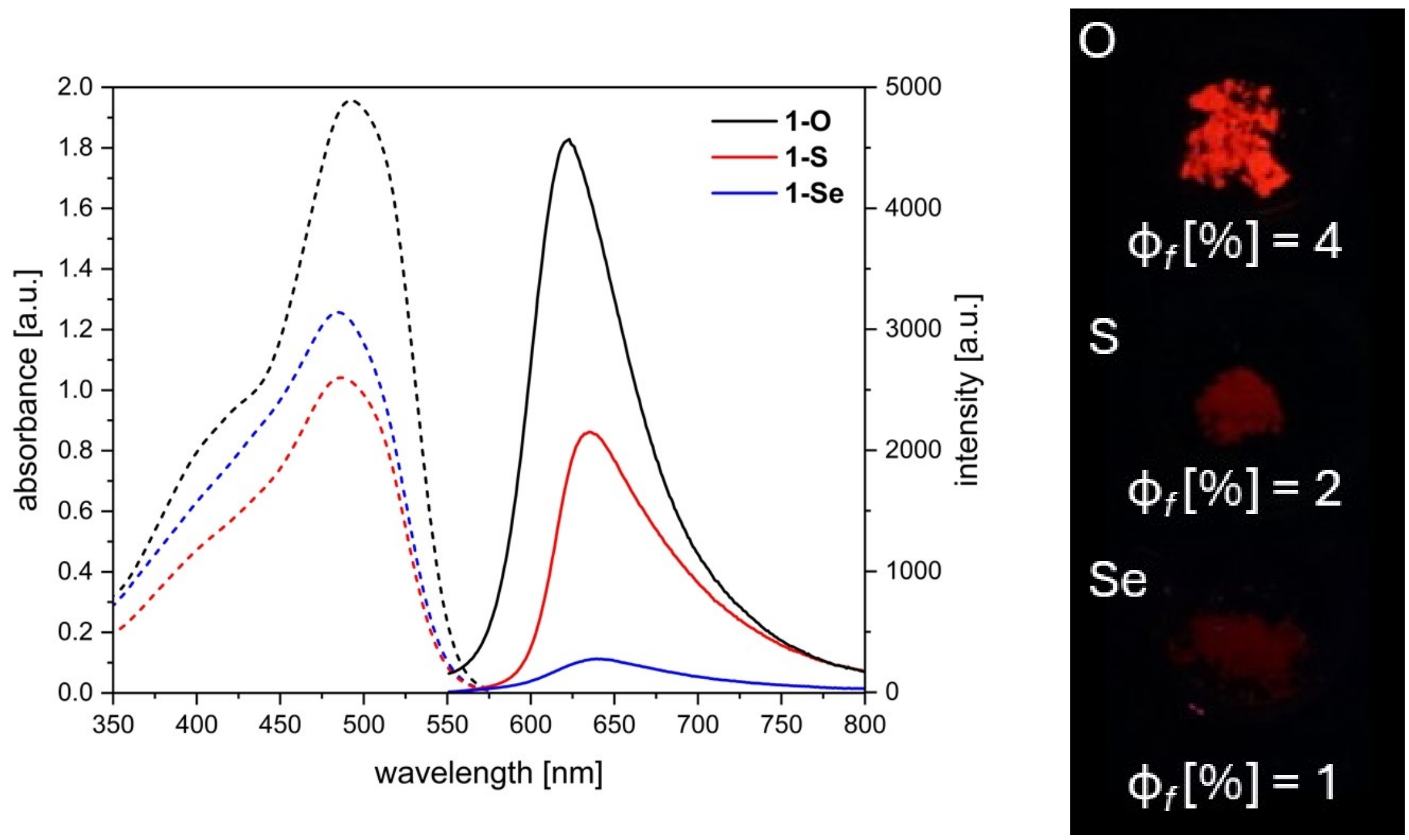

Figure S45. Absorption spectra in methanol, c = 1 mM (dashed) and solid-state emission spectra (solid) of *ortho*-chalcogen-substituted azoarenes **1**. Black: oxygen (**1-O**); red: sulfur (**1-S**); blue: selenium (**1-Se**). Pictures of the compounds under irradiation with light of the wavelength λ = 365 nm. Absolute photoluminescence quantum yields in the solid state Φ_f are given in percent with an uncertainty of ±2%.

3. Synthesis of New Compounds

General remarks. All chemicals were reagent grade and used as received from ABCR, Alfa Aesar, Acros Organics, Fisher, TCI, or Sigma-Aldrich. Reactions were monitored by TLC analysis with silica gel 60 F254 thin-layer plates. Flash chromatography was performed on silica 60 (40–63 µm, 230–400 mesh). 1H, 13C, 77Se, and 125Te NMR spectra were recorded using Bruker Avance NEO 400 and Avance HD 600 spectrometers. All chemical shifts (δ) are reported in ppm. The spectra were referenced to the peak of the protium impurity in the deuterated solvents listed in brackets in the analytical data ($CDCl_3$, ^1H: 7.26 ppm, ^{13}C: 77.16 ppm; MeOD, ^1H: 3.31 ppm, ^{13}C: 49.00 ppm; DMSO-d_6, ^1H: 2.50 ppm, ^{13}C: 39.52 ppm). Signal multiplicity for ^1H NMR was designated as s (singlet), d (doublet), t (triplet), dd (doublet of doublets), dt (doublet of triplets), and m (multiplet). ^{13}C NMR spectra were recorded with ^1H decoupling. The ^{13}C signals were categorized as p (primary), s (secondary), t (tertiary), and q (quaternary) carbon atoms. HRMS spectra were obtained using a Bruker BioTOF III mass spectrometer with electrospray ionization (ESI). UV/Vis absorption spectra were measured with a Jasco V-550 spectrophotometer. IR absorption spectra were recorded with a Shimadzu IR Tracer 100 IR spectrometer. Melting points were determined with a Büchi melting point apparatus (Model B-540) using an open capillary and are uncorrected. Compounds **6**, **7**, *p*-**9b**, and **9f** were synthesized according to procedures reported in the literature.[1-4]

General procedure (*A*) for the synthesis of the *β*-naphthol azoarenes.

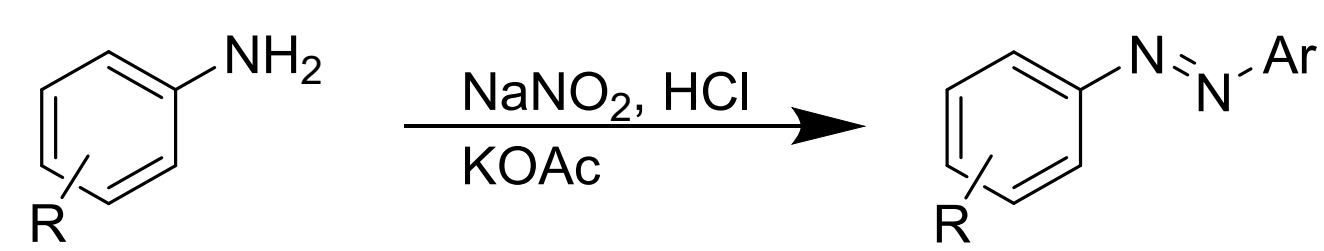

The corresponding aniline (8.85 mmol, 1 eq) was dissolved in a mixture of glacial acetic acid (12.5 mL) and hydrochloric acid (2.5 mL). The reaction mixture was cooled in an ice bath, and sodium nitrite (0.760 g, 11.01 mmol, 1.24 eq) in water (3 mL) was added, and the mixture was stirred for an additional 30 minutes. The desired aryl (11.47 mmol, 1.3 eq) and potassium acetate (2.789 g, 28.2 mmol, 3.18 eq) were dissolved in 10 mL ethanol/water (1:1) and cooled to 0 °C. The diazonium salt solution was added dropwise to this solution, and the resulting mixture was stirred for 30 minutes to 18 hours. The crude product was filtered off and washed with cold water. Purification depended on the derivative.

General procedure (*B*) for the synthesis of *ortho*-tellurated azoarenes.

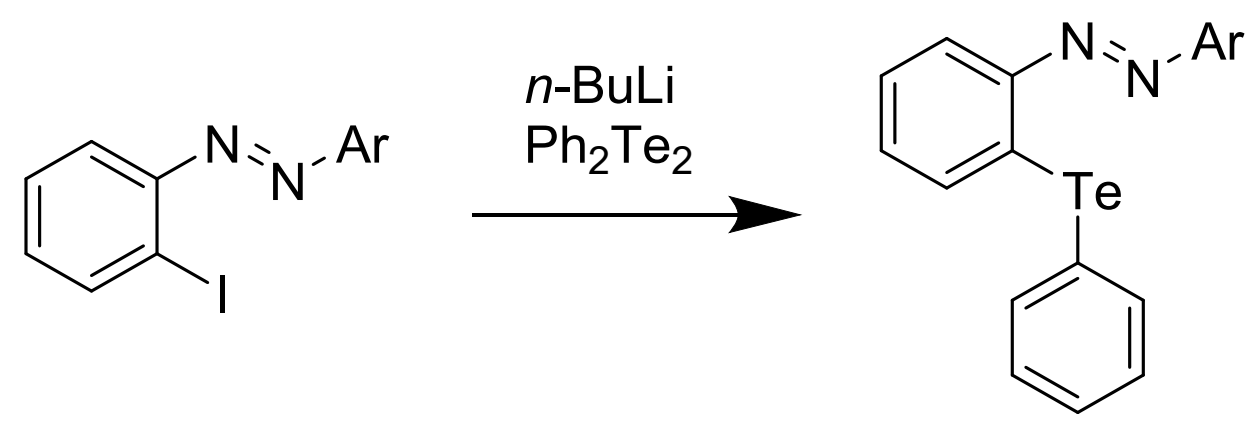

To a solution of aryl iodide (0.15 mmol, 1 eq) in 8 mL dry diethyl ether, *n*-butyllithium in hexane (2.5 M, 0.2 mL) was added at −78 °C under argon atmosphere and the mixture was stirred at this temperature for 10 minutes. This step was followed by dropwise addition of a solution of diphenyl ditelluride (0.15 mmol, 1 eq) in dry diethyl ether (8 mL). The resulting mixture was stirred at −78 °C for 1-2 h. The reaction mixture was allowed to reach room temperature and was then washed with saturated $NaHCO_3$. The organic layer was dried over $MgSO_4$ and concentrated in vacuo. The residue was purified by flash column chromatography with silica gel to provide the desired azoarene.

General procedure (*C*) for the synthesis of azoarenes 1-S and 1-Se.

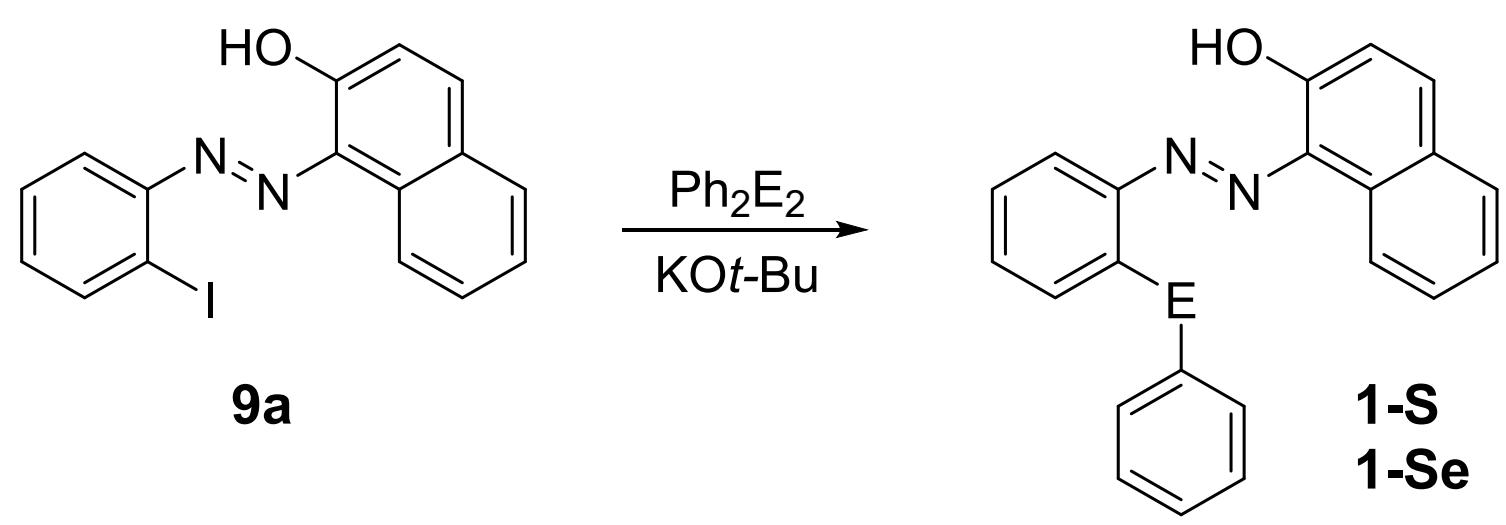

Azo compound **9a** (374 mg, 1.0 mmol, 1.0 eq) and the corresponding diphenyl dichalcogenide (1.00 mmol, 1.0 eq) were dissolved in 2 mL DMSO and heated to 45 °C. Potassium *tert*-butanolate (140 mg, 1.2 mmol, 1.2 eq) was added in several portions and then stirred at 45 °C for a further 15 minutes. The mixture was then heated to 80 °C and stirred overnight. After cooling to room temperature, the organic phase was added to water, extracted with ethyl acetate, and dried over magnesium sulfate. The solvent was removed under reduced pressure in a rotary evaporator, and the crude product was purified by column chromatography.

General procedure (*D*) for the Miyaura borylation of 9b.

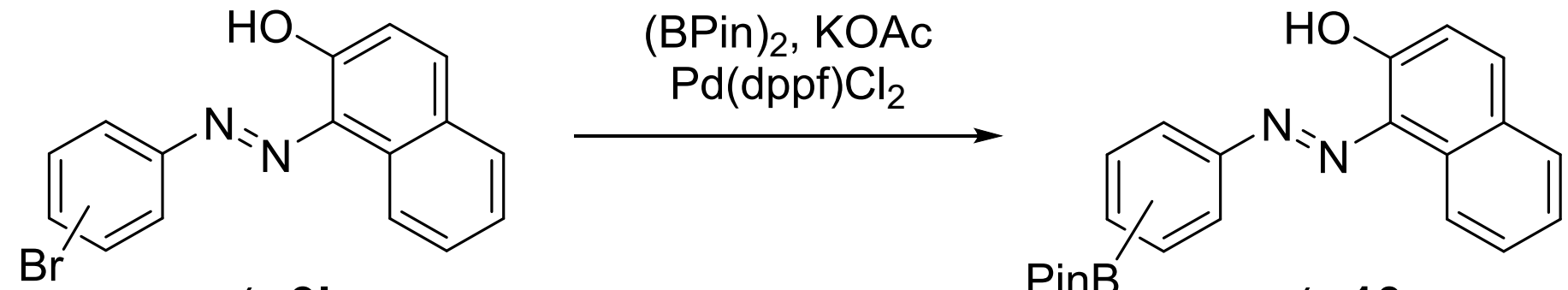

m/p-**9b** *m/p*-**10**

After heating the Schlenk flask to remove moisture, bis(pinacolato)diboron ($(BPin)_2$, 304 mg, 1.2 mmol, 1.2 eq), potassium acetate (294 mg, 3.0 mmol, 3.0 eq), and aryl bromide **9b** (327 mg, 1.0 mmol, 1.0 eq) were added in 6 mL of dry dioxane. The reaction mixture was degassed with argon for 30 minutes. The palladium catalyst ($Pd(dppf)Cl_2$, 40 mg, 0.05 mmol, 0.05 eq) was added, and the reaction mixture was stirred overnight at 110 °C. After cooling to room temperature, the solvent was removed under reduced pressure. The residue was dissolved in ethyl acetate and washed with saturated aqueous NaCl solution. The organic phase was dried over magnesium sulfate, and the solvent was removed using a rotary evaporator. The crude product was purified by column chromatography.

General procedure (*E*) for the synthesis of *meta/para*-tellurated azoarenes.

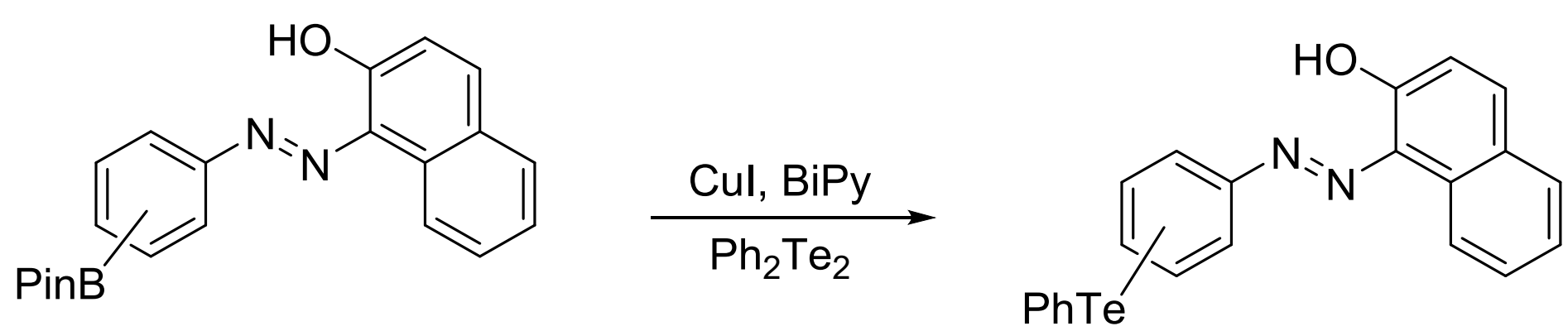

m/p-**10** **2a**

The pinacol boronic acid ester **10** (94 mg, 0.25 mmol, 1.0 eq), diphenyl ditelluride (102 mg, 0.25 mmol, 1.0 eq), copper(I) iodide (1 mg, 0.005 mmol, 0.005 eq), and bipyridine (BiPy, 0.7 mg, 0.005 mmol, 0.005 eq) were suspended in a 2:1 mixture of DMSO/water (0.3 mL) and stirred overnight at 100 °C in air. The reaction mixture was then treated with diethyl ether and successively washed with water and saturated aqueous NaCl solution. The organic phase was dried over magnesium sulfate, and the excess solvent was removed under reduced pressure. The crude product is purified by column chromatography.

Synthesis of 1-O:

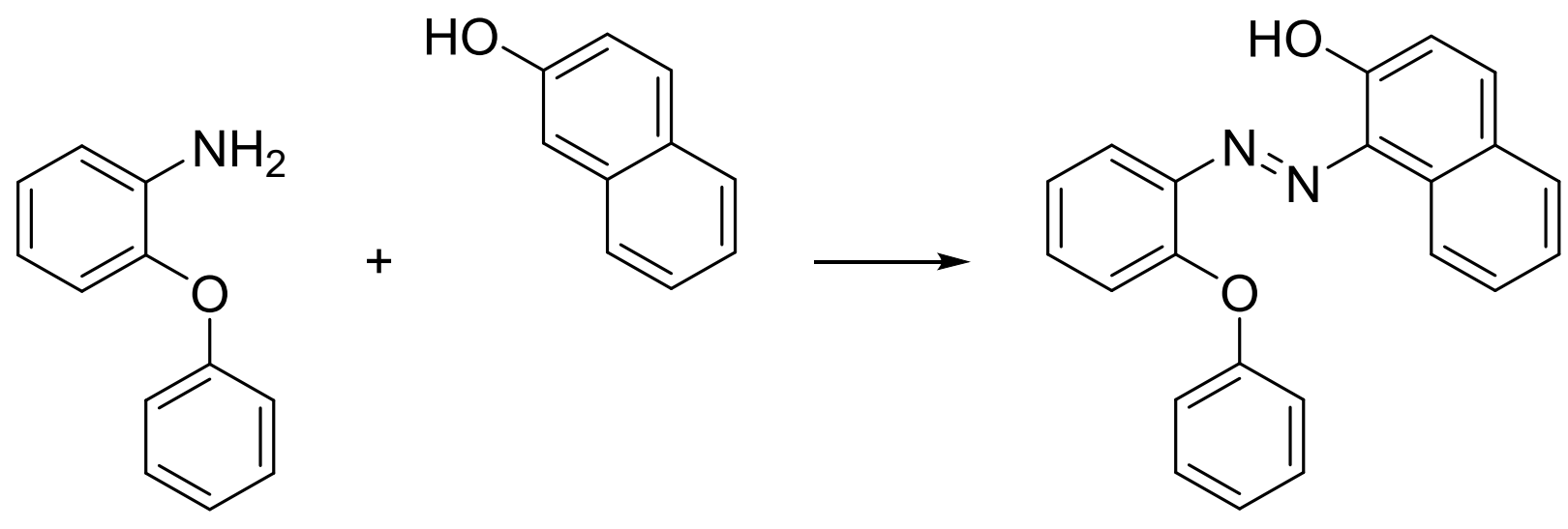

8a **11** **1-O**

Azoarene **1-O** was synthesized according to the general procedure (*A*) using 2-phenoxyaniline (**8a**) and 2-naphthol (**11**). The crude product was purified via column chromatography.

Yield: 1.43 g, 4.20 mmol, 95%

R_f (DCM) = 0.4

M.p.: 153 °C

^{1}H NMR (400 MHz, $CDCl_3$): δ = 16.49 (s, 1 H, OH), 8.52 (d, $^3J_{H,H}$ = 7.8 Hz, 1 H, $C_{ar}H$), 8.15 (dd, $^3J_{H,H}$ = 8.1 Hz, $^4J_{H,H}$ = 1.7 Hz, 1 H, $C_{ar}H$), 7.65 (d, $^3J_{H,H}$ = 9.5 Hz, 1 H, $C_{ar}H$), 7.57–7.51 (m, 2 H, $C_{ar}H$), 7.41–7.34 (m, 3 H, $C_{ar}H$), 7.25 (dt, $^3J_{H,H}$ = 8.0 Hz, $^4J_{H,H}$ = 1.3 Hz, 1 H, $C_{ar}H$), 7.17 (dt, $^3J_{H,H}$ = 8.0 Hz, $^4J_{H,H}$ = 1.7 Hz, 1 H, $C_{ar}H$), 7.16–7.11 (m, 3 H, $C_{ar}H$), 6.99 (dd, $^3J_{H,H}$ = 8.2, $^4J_{H,H}$ = 1.4 Hz, 1 H, $C_{ar}H$), 6.76 ppm (d, $^3J_{H,H}$ = 9.5 Hz, 1 H, $C_{ar}H$).

^{13}C NMR (101 MHz, $CDCl_3$): δ = 176.2 (q, C_{ar}), 156.4 (q, C_{ar}), 147.3 (q, C_{ar}), 140.9 (t, $C_{ar}H$), 134.9 (q, C_{ar}), 133.6 (q, C_{ar}), 130.9 (q, C_{ar}), 129.9 (t, $C_{ar}H$), 129.0 (t, $C_{ar}H$), 128.7 (t, $C_{ar}H$), 128.0 (q, C_{ar}), 126.7 (t, $C_{ar}H$), 126.1 (t, $C_{ar}H$), 126.0 (t, $C_{ar}H$), 124.4 (t, $C_{ar}H$), 123.9 (t, $C_{ar}H$), 121.8 (t, $C_{ar}H$), 118.8 (t, $C_{ar}H$), 118.8 (t, $C_{ar}H$), 116.7 ppm (t, $C_{ar}H$).

IR (ATR): $\tilde{\nu}$ = 3055, 3038, 1620, 1582, 1557, 1549, 1497, 1487, 1476, 1460, 1447, 1383, 1290, 1242, 1221, 1207, 1144, 1134, 1099, 1069, 1032, 1022, 988, 970, 868, 831, 797, 762, 748, 719, 691, 625 cm^{-1}.

UV/Vis (MeOH): λ_{max} (log ε) = 309 (3.09), 493 nm (3.50).

HRMS (ESI): [$C_{22}H_{16}N_2O_2+H^+$]: calculated: 341.1285; observed: 341.1287.

Synthesis of 9a:

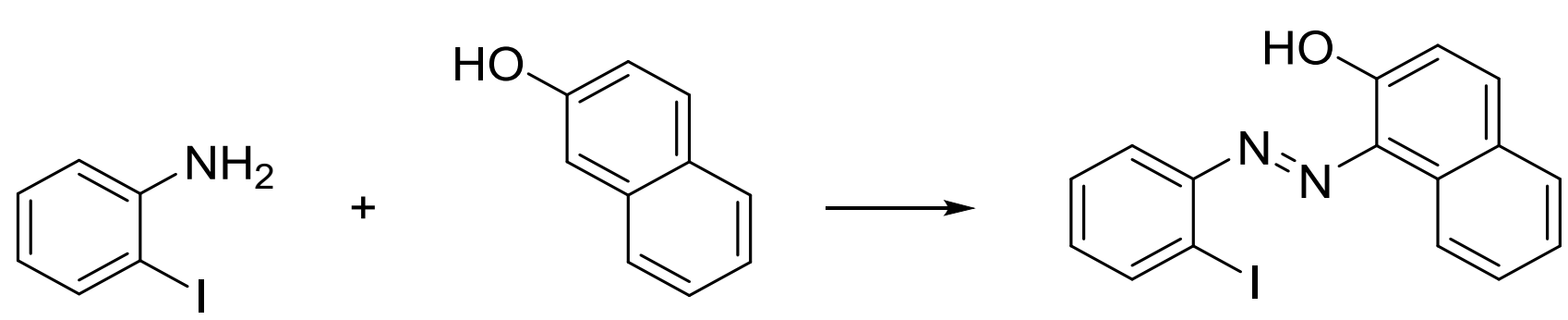

8b **11** **9a**

Azoarene **9a** was synthesized according to the general procedure (*A*) using 2-iodoaniline (**8b**) and 2-naphthol (**11**). The crude product was used without further purification.

Yield: 2.89 g, 7.11 mmol, 88%

M.p.: 175 °C

^{1}H NMR (600 MHz, $CDCl_3$): δ = 15.85 (s, 1 H, OH), 8.58 (d, $^{3}J_{H,H}$ = 8.2 Hz, 1 H, C_{ar}H), 8.04 (dd, $^{3}J_{H,H}$ = 8.2 Hz, $^{4}J_{H,H}$ = 1.5 Hz, 1 H, C_{ar}H), 7.92 (dd, $^{3}J_{H,H}$ = 8.0 Hz, $^{4}J_{H,H}$ = 1.2 Hz, 1 H, C_{ar}H), 7.75 (d, $^{3}J_{H,H}$ = 9.5 Hz, 1 H, C_{ar}H), 7.62 (d, $^{3}J_{H,H}$ = 7.9 Hz, 1 H, C_{ar}H), 7.56 (dt, $^{3}J_{H,H}$ = 7.7 Hz, $^{4}J_{H,H}$ = 1.3 Hz, 1 H, C_{ar}H), 7.48 (dt, $^{3}J_{H,H}$ = 7.7 Hz, $^{4}J_{H,H}$ = 1.3 Hz, 1 H, C_{ar}H), 7.42 (dt, $^{3}J_{H,H}$ = 7.3 Hz, $^{4}J_{H,H}$ = 1.2 Hz, 1 H, C_{ar}H), 7.02 (dt, $^{3}J_{H,H}$ = 7.1 Hz, $^{4}J_{H,H}$ = 1.5 Hz, 1 H, C_{ar}H), 6.92 ppm (d, $^{3}J_{H,H}$ = 9.5 Hz, 1 H, C_{ar}H).

^{13}C NMR (151 MHz, $CDCl_3$): δ = 171.0 (q, C_{ar}), 146.0 (q, C_{ar}), 140.7 (t, C_{ar}H), 139.9 (t, C_{ar}H), 135.6 (q, C_{ar}), 130.7 (q, C_{ar}), 129.5 (t, C_{ar}H), 129.2 (t, C_{ar}H), 128.9 (t, C_{ar}H), 128.6 (q, C_{ar}), 128.5 (t, C_{ar}H), 126.3 (t, C_{ar}H), 124.7 (t, C_{ar}H), 122.1 (t, C_{ar}H), 117.2 (t, C_{ar}H), 91.0 ppm (q, C_{ar}).

IR (ATR): $\tilde{\nu}$ = 3061, 1618, 1553, 1489, 1437, 1281, 1242, 1202, 1175, 1107, 1092, 1040, 1013, 986, 945, 835, 777, 745, 683, 638 cm^{-1}.

UV/Vis ($CHCl_3$): λ_{max} (log ε) = 309 (2.73), 482 nm (3.03).

HRMS (ESI): [$C_{16}H_{11}N_2OI+H^+$]: calculated: 374.9989; observed: 374.9988.

Synthesis of 9c:

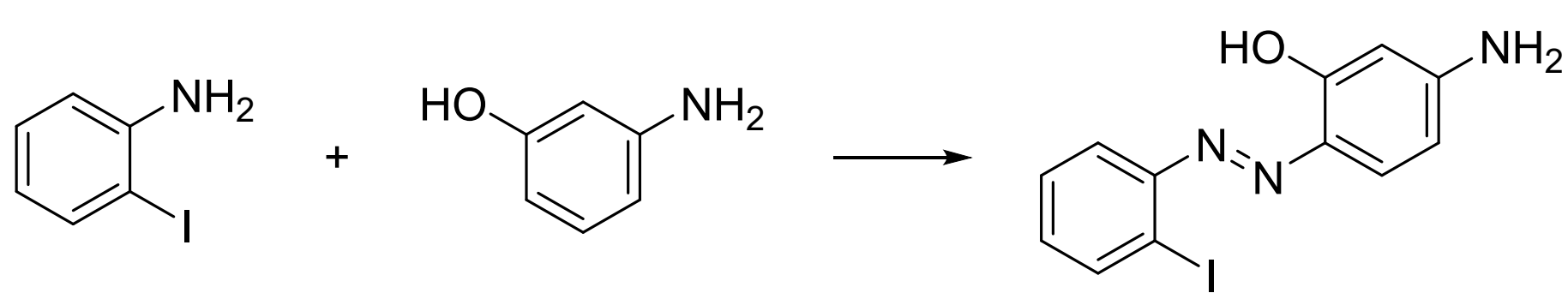

8b **12** **9c**

Azoarene **9c** was synthesized according to the general procedure (*A*) using 2-iodoaniline (**8b**) and 3-aminophenol (**12**). The crude product was purified via column chromatography.

Yield: 4.51 g, 13.31 mmol, 76%

R_f (DCM/ethyl acetate 4:1) = 0.6

M.p.: 149 °C

^{1}H NMR (400 MHz, DMSO-d_6): δ = 13.94 (s, 1 H, OH), 7.97 (dd, $^{3}J_{H,H}$ = 7.9 Hz, $^{4}J_{H,H}$ = 1.3 Hz, 1 H, C_{ar}H), 7.70 (dd, $^{3}J_{H,H}$ = 8.3 Hz, $^{4}J_{H,H}$ = 1.5 Hz, 1 H, C_{ar}H), 7.50–7.43 (m, 2 H, C_{ar}H), 7.10 (dt, $^{3}J_{H,H}$ = 7.5 Hz, $^{4}J_{H,H}$ = 1.6 Hz, 1 H, C_{ar}H), 6.83 (s, 2 H, NH_2), 6.38 (dd, $^{3}J_{H,H}$ = 9.0 Hz, $^{4}J_{H,H}$ = 2.3 Hz, 1 H, C_{ar}H), 6.01 ppm (d, $^{4}J_{H,H}$ = 2.3 Hz, 1 H, C_{ar}H).

^{13}C NMR (101 MHz, DMSO-d$_6$): δ = 158.1 (q, C_{ar}), 156.2 (q, C_{ar}), 149.4 (q, C_{ar}), 139.4 (t, $C_{ar}H$), 134.9 (t, $C_{ar}H$), 130.8 (q, C_{ar}), 129.7 (t, $C_{ar}H$), 129.3 (t, $C_{ar}H$), 116.6 (t, $C_{ar}H$), 109.4 (t, $C_{ar}H$), 98.4 (t, $C_{ar}H$), 97.5 ppm (q, C_{ar}).

IR (ATR): $\tilde{\nu}$ = 3424, 3318, 3200, 1626, 1612, 1560, 1474, 1456, 1373, 1314, 1254, 1188, 1128, 1111, 1016, 814, 764, 604 cm^{-1}.

UV/Vis ($CHCl_3$): λ_{max} (log ε) = 413 nm (3.06).

HRMS (ESI): [$C_{12}H_{10}N_3OI+H^+$]: calculated: 339.9941; observed: 339.9943.

Synthesis of *m*-9b:

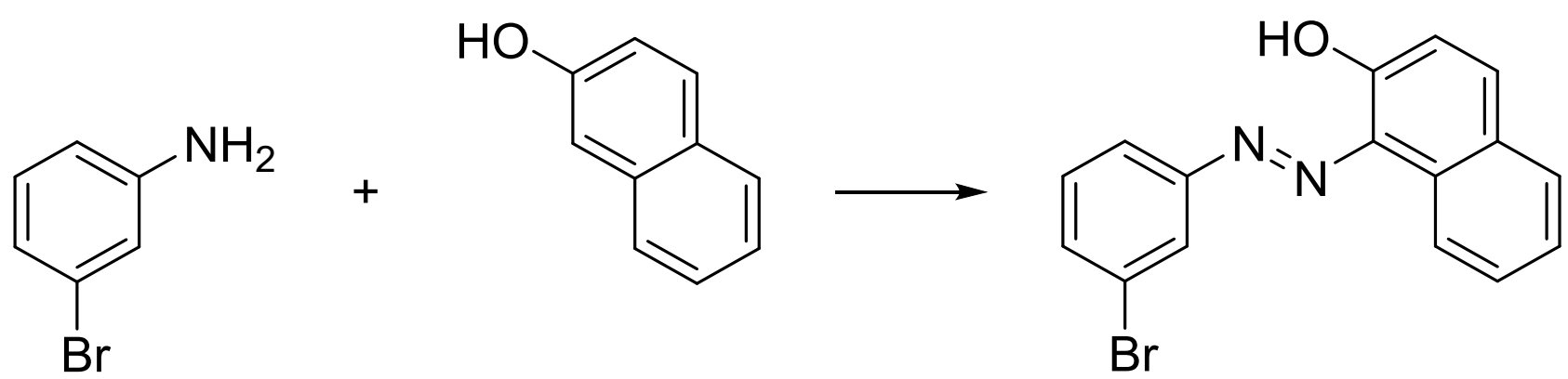

8c **11** ***m*-9b**

Azoarene *m*-**9b** was synthesized according to the general procedure (*A*) using 3-bromonaniline (**8c**) and 2-naphthol (**11**). The crude product was purified via column chromatography.

Yield: 2.65 g, 8.11 mmol, 92%

R_f (DCM) = 0.6

M.p.: 179 °C

^1H NMR (600 MHz, $CDCl_3$): δ = 16.05 (s, 1 H, OH), 8.53 (d, $^3J_{H,H}$ = 8.2 Hz, 1 H, $C_{ar}H$), 7.93 (t, $^4J_{H,H}$ = 1.8 Hz, 1 H, $C_{ar}H$), 7.72 (d, $^3J_{H,H}$ = 9.4 Hz, 1 H, $C_{ar}H$), 7.62–7.55 (m, 3 H, $C_{ar}H$), 7.44–7.38 (m, 2 H, $C_{ar}H$), 7.33 (t, $^3J_{H,H}$ = 8.0 Hz, 1 H, $C_{ar}H$), 6.83 ppm (d, $^3J_{H,H}$ = 9.4 Hz, 1 H, $C_{ar}H$).

^{13}C NMR (151 MHz, $CDCl_3$): δ = 173.3 (q, C_{ar}), 146.0 (q, C_{ar}), 141.1 (t, $C_{ar}H$), 133.5 (q, C_{ar}), 131.0 (t, $C_{ar}H$), 130.6 (q, C_{ar}), 130.0 (t, $C_{ar}H$), 129.3 (t, $C_{ar}H$), 128.9 (t, $C_{ar}H$), 128.4 (q, C_{ar}), 126.4 (t, $C_{ar}H$), 125.1 (t, $C_{ar}H$), 123.7 (q, C_{ar}), 122.1 (t, $C_{ar}H$), 120.9 (t, $C_{ar}H$), 117.6 ppm (t, $C_{ar}H$).

IR (ATR): $\tilde{\nu}$ = 3078, 3067, 1616, 1591, 1497, 1472, 1450, 1437, 1387, 1327, 1319, 1302, 1250, 1223, 1204, 1161, 1148, 1096, 1061, 1034, 991, 982, 970, 951, 889, 860, 833, 779, 752, 689, 671, 646 cm^{-1}.

UV/Vis (MeOH): λ_{max} (log ε) = 303 (3.03), 433 (3.20), 474 nm (3.33).

HRMS (ESI): [$C_{16}H_{11}{}^{79}BrN_2O+H^+$]: calculated: 327.0128; observed: 327.0129.

Synthesis of 9d:

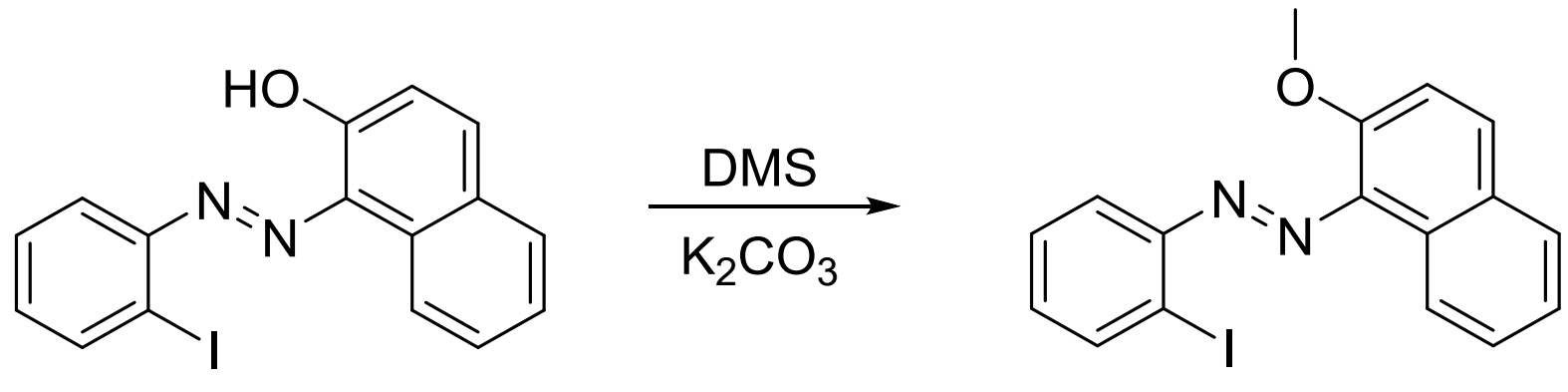

Azoarene **9a** (750 mg, 2.0 mmol, 1.0 eq), dimethyl sulfate (DMS, 252 mg, 2.0 mmol, 1.0 eq), and potassium carbonate (555 mg, 4.0 mmol, 2.0 eq) were suspended in 7 mL acetonitrile. After stirring the suspension at 100 °C overnight, the reaction mixture was quenched with water and extracted with diethyl ether. The combined organic phases were dried over magnesium sulfate, and the solvent was removed in vacuo. The crude product was purified via column chromatography.

Yield: 560 mg, 1.44 mmol, 72%

R_f (*n*-hexane/DCM 2:1) = 0.2

M.p.: 95 °C

^{1}H NMR (400 MHz, $CDCl_3$): δ = 8.94 (dd, $^3J_{H,H}$ = 8.7 Hz, $^4J_{H,H}$ = 0.9 Hz, 1 H, $C_{ar}H$), 8.07 (dd, $^3J_{H,H}$ = 7.1 Hz, $^4J_{H,H}$ = 1.3 Hz, 1 H, $C_{ar}H$), 7.94 (d, $^3J_{H,H}$ = 9.1 Hz, 1 H, $C_{ar}H$), 7.82 (d, $^3J_{H,H}$ = 8.1 Hz, 1 H, $C_{ar}H$), 7.75 (dd, $^3J_{H,H}$ = 8.0 Hz, $^4J_{H,H}$ = 1.5 Hz, 1 H, $C_{ar}H$), 7.61 (dt, $^3J_{H,H}$ = 7.9 Hz, $^4J_{H,H}$ = 1.4 Hz, 1 H, $C_{ar}H$), 7.50–7.40 (m, 3 H, $C_{ar}H$), 7.16 (dt, $^3J_{H,H}$ = 7.6 Hz, $^4J_{H,H}$ = 1.6 Hz, 1 H, $C_{ar}H$), 4.10 ppm (s, 3 H, O-CH_3).

^{13}C NMR (151 MHz, $CDCl_3$): δ = 153.6 (q, C_{ar}), 152.1 (q, C_{ar}), 140.0 (t, $C_{ar}H$), 135.0 (q, C_{ar}), 133.3 (t, $C_{ar}H$), 131.8 (t, $C_{ar}H$), 129.3 (q, C_{ar}), 129.0 (t, $C_{ar}H$), 128.9 (t, $C_{ar}H$), 128.1 (t, $C_{ar}H$), 128.0 (q, C_{ar}), 124.8 (t, $C_{ar}H$), 124.5 (t, $C_{ar}H$), 117.5 (t, $C_{ar}H$), 114.6 (t, $C_{ar}H$), 102.1 (q, C_{ar}), 57.8 ppm (s, O-CH_3).

IR (ATR): $\tilde{\nu}$ = 3062, 3055, 2980, 2971, 2933, 2903, 2889, 2835, 1894, 1858, 1830, 1748, 1719, 1700, 1686, 1664, 1621, 1589, 1561, 1505, 1472, 1451, 1418, 1395, 1378, 1343, 1294, 1270, 1249, 1198, 1181, 1142, 1087, 1039, 1014, 956, 921, 873, 860, 803, 765, 746, 704, 670, 658, 633 cm^{-1}.

UV/Vis (MeCN): λ_{max} (log ε) = 404 (2.97), 445 nm (2.52).

HRMS (ESI): [$C_{17}H_{13}N_2OI+H^+$]: calculated: 389.0145; observed: 389.0145.

Synthesis of 9e:

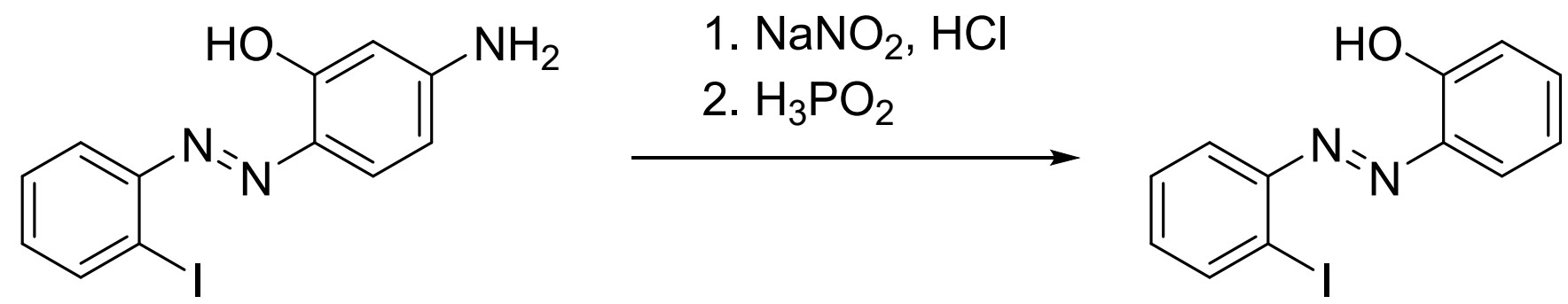

9c **9e**

Amino azoarene **9c** (1.70 g, 5.0 mmol, 1.0 eq) was dissolved in a mixture of 10 mL hydrochloric acid and 40 mL glacial acetic acid and cooled to 0 °C. Sodium nitrite (0.80 g, 11.6 mmol, 2.3 eq) was dissolved in 10 mL of water and slowly added to the azoarene solution. The reaction mixture was stirred at 0 °C for an additional hour. Afterwards, 50 mL of phosphinic acid (50wt%) cooled to 0 °C was added. The reaction mixture was stirred at room temperature overnight, neutralized with sodium hydroxide solution, and extracted several times with ethyl acetate. The combined organic phases were dried over magnesium sulfate, and the solvent was removed in vacuo. The crude product was purified via column chromatography.

Yield: 360 mg, 1.11 mmol, 22%

R_f (DCM) = 0.8

M.p.: 147 °C

^{1}H NMR (400 MHz, $CDCl_3$): δ = 12.63 (s, 1 H, OH), 8.04 (dd, $^{3}J_{H,H}$ = 8.0 Hz, $^{4}J_{H,H}$ = 1.3 Hz, 1 H, C_{ar}H), 7.97 (dd, $^{3}J_{H,H}$ = 7.9 Hz, $^{4}J_{H,H}$ = 1.7 Hz, 1 H, C_{ar}H), 7.85 (dd, $^{3}J_{H,H}$ = 8.1 Hz, $^{4}J_{H,H}$ = 1.6 Hz, 1 H, C_{ar}H), 7.47 (dt, $^{3}J_{H,H}$ = 7.6 Hz, $^{4}J_{H,H}$ = 1.3 Hz, 1 H, C_{ar}H), 7.40 (dt, $^{3}J_{H,H}$ = 7.8 Hz, $^{4}J_{H,H}$ = 1.7 Hz, 1 H, C_{ar}H), 7.19 (dt, $^{3}J_{H,H}$ = 7.6 Hz, $^{4}J_{H,H}$ = 1.6 Hz, 1 H, C_{ar}H), 7.13–7.06 ppm (m, 2 H, C_{ar}H).

^{13}C NMR (101 MHz, $CDCl_3$): δ = 152.3 (q, C_{ar}), 150.3 (q, C_{ar}), 140.0 (t, C_{ar}H), 137.5 (q, C_{ar}), 134.2 (t, C_{ar}H), 134.0 (t, C_{ar}H), 132.5 (t, C_{ar}H), 129.3 (t, C_{ar}H), 120.2 (t, C_{ar}H), 118.6 (t, C_{ar}H), 117.9 (t, C_{ar}H), 101.2 ppm (q, C_{ar}).

IR (ATR): $\tilde{\nu}$ = 3048, 1582, 1481, 1452, 1356, 1271, 1258, 1148, 1063, 997, 858, 762, 752, 733, 704, 691 cm^{-1}.

UV/Vis ($CHCl_3$): λ_{max} (log ε) = 328 (3.04), 390 nm (2.83).

HRMS (ESI): [$C_{12}H_9N_2OI+H^+$]: calculated: 324.9832; observed: 324.9833.

Synthesis of *o*-2a:

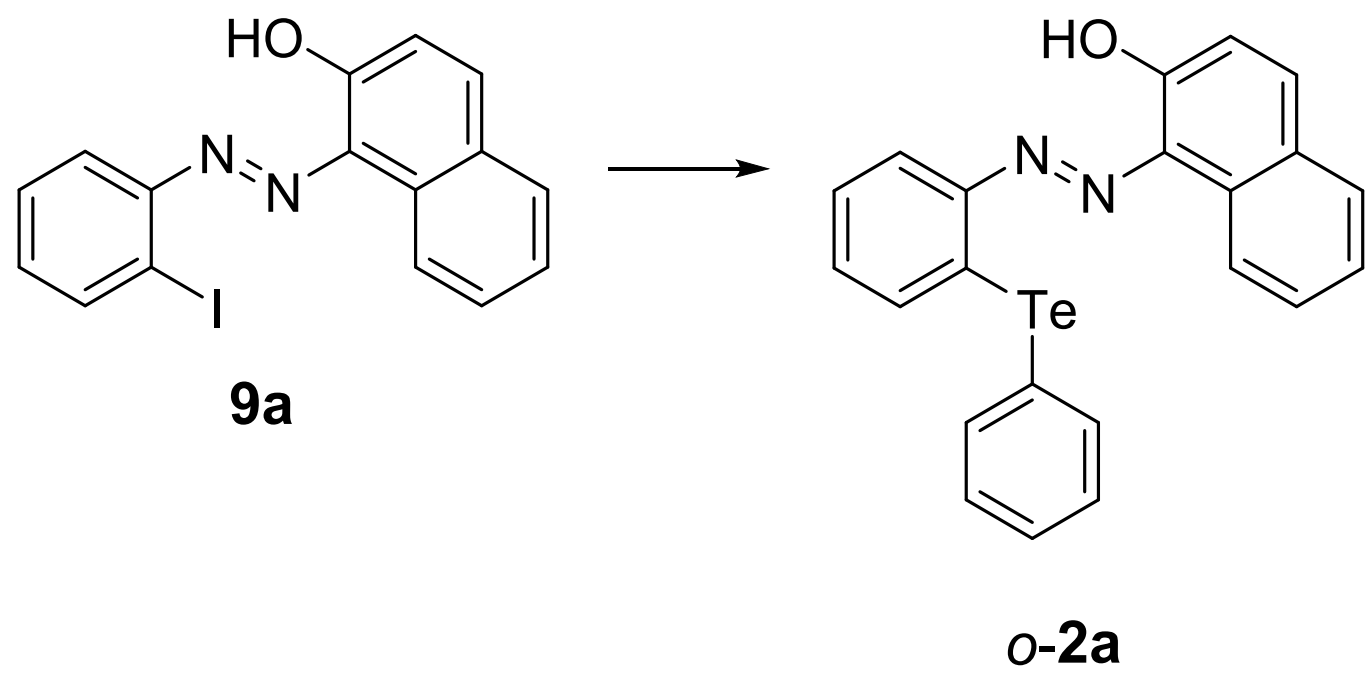

Azoarene *o*-**2a** was synthesized according to the general procedure (*B*). The crude product was purified via column chromatography.

Yield: 80 mg, 0.18 mmol, 11%

R_f (*n*-hexane/DCM 3:2) = 0.4

M.p.: 117 °C

^{1}H NMR (600 MHz, $CDCl_3$): δ = 15.45 (s, 1 H, OH), 8.92 (d, $^{3}J_{H,H}$ = 8.4 Hz, 1 H, C_{ar}H), 7.91 (m, 3 H, C_{ar}H), 7.82 (d, $^{3}J_{H,H}$ = 9.2 Hz, 1 H, C_{ar}H), 7.75 (d, $^{3}J_{H,H}$ = 7.8 Hz, 1 H, C_{ar}H), 7.66 (dt, $^{3}J_{H,H}$ = 7.7 Hz, $^{4}J_{H,H}$ = 1.1 Hz, 1 H, C_{ar}H), 7.46 (dt, $^{3}J_{H,H}$ = 7.4 Hz, $^{4}J_{H,H}$ = 1.0 Hz, 1 H, C_{ar}H), 7.41 (m, 3 H, C_{ar}H), 7.31 (t, $^{3}J_{H,H}$ = 7.6 Hz, 2 H, C_{ar}H), 7.11 (d, $^{3}J_{H,H}$ = 9.2 Hz, 1 H, C_{ar}H), 7.07 ppm (dt, $^{3}J_{H,H}$ = 7.1 Hz, $^{4}J_{H,H}$ = 1.3 Hz, 1 H, C_{ar}H).

^{13}C NMR (151 MHz, $CDCl_3$): δ = 160.1 (q, C_{ar}), 148.4 (q, C_{ar}), 140.1 (t, C_{ar}H), 137.0 (t, C_{ar}H), 136.1 (t, C_{ar}H), 132.6 (q, C_{ar}), 130.5 (q, C_{ar}), 129.6 (t, C_{ar}H), 129.0 (t, C_{ar}H), 128.8 (t, C_{ar}H), 128.6 (t, C_{ar}H), 128.5 (t, C_{ar}H), 128.4 (q, C_{ar}), 127.6 (t, C_{ar}H), 125.7 (t, C_{ar}H), 125.1 (t, C_{ar}H), 122.5 (t, C_{ar}H), 121.9 (t, C_{ar}H), 117.6 (q, C_{ar}), 112.7 ppm (q, C_{ar}).

^{125}Te NMR (189 MHz, $CDCl_3$): δ = 667 ppm.

^{125}Te NMR (189 MHz, MeOD): δ = 661 ppm.

IR (ATR): $\tilde{\nu}$ = 3381, 2961, 2924, 1622, 1258, 1084, 1015, 864, 793, 720 cm^{-1}.

UV/Vis (MeOH): λ_{max} (log ε) = 408 (2.72), 503 nm (2.81).

HRMS (ESI): [$C_{22}H_{16}N_2O^{130}Te+H^+$]: calculated: 455.0399; observed: 455.0399.

Synthesis of 3a:

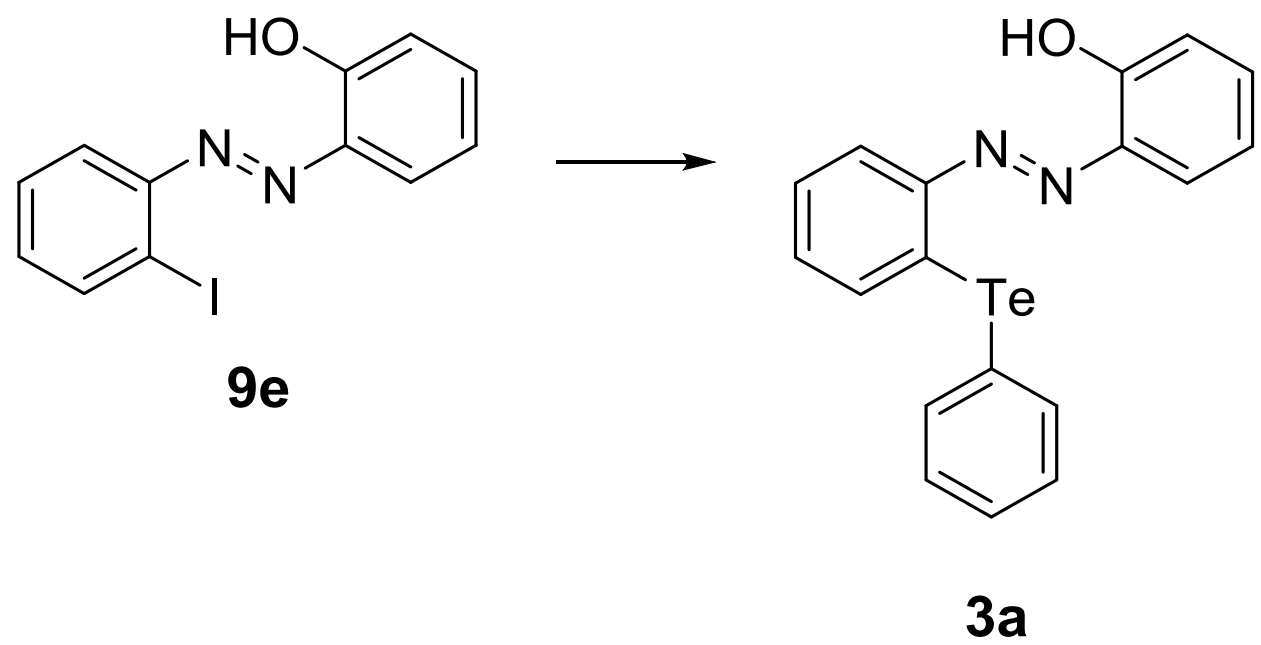

Azoarene **3a** was synthesized according to the general procedure (*B*). The crude product was purified via column chromatography.

Yield: 25 mg, 0.06 mmol, 8%

R_f (*n*-hexane/DCM 9:1) = 0.4

M.p.: 124 °C

^{1}H NMR (400 MHz, $CDCl_3$): δ = 12.49 (s, 1 H, OH), 8.19 (d, $^3J_{H,H}$ = 7.8 Hz, 1 H, $C_{ar}H$), 8.02 (dd, $^3J_{H,H}$ = 7.9 Hz, $^4J_{H,H}$ = 1.4 Hz, 1 H, $C_{ar}H$), 7.92 (dd, $^3J_{H,H}$ = 8.2 Hz, $^4J_{H,H}$ = 1.4 Hz, 2 H, $C_{ar}H$), 7.66 (tt, $^3J_{H,H}$ = 7.4 Hz, $^4J_{H,H}$ = 1.3 Hz, 1 H, $C_{ar}H$), 7.41–7.35 (m, 4 H, $C_{ar}H$), 7.24 (dd, $^3J_{H,H}$ = 8.1 Hz, $^4J_{H,H}$ = 1.2 Hz, 1 H, $C_{ar}H$), 7.11–7.04 ppm (m, 3 H, $C_{ar}H$).

^{13}C NMR (101 MHz, $CDCl_3$): δ = 152.3 (q, C_{ar}), 150.0 (q, C_{ar}), 140.6 (t, $C_{ar}H$), 136.2 (q, C_{ar}), 134.5 (t, $C_{ar}H$), 133.5 (t, $C_{ar}H$), 132.1 (t, $C_{ar}H$), 131.5 (t, $C_{ar}H$), 130.6 (t, $C_{ar}H$), 129.8 (t, $C_{ar}H$), 129.1 (t, $C_{ar}H$), 126.8 (t, $C_{ar}H$), 121.5 (q, C_{ar}), 120.4 (t, $C_{ar}H$), 118.8 (t, $C_{ar}H$), 115.9 ppm (q, C_{ar}).

^{125}Te NMR (189 MHz, $CDCl_3$): δ = 738 ppm.

^{125}Te NMR (189 MHz, MeOD): δ = 738 ppm.

IR (ATR): $\tilde{\nu}$ = 3061, 3048, 1582, 1481, 1356, 1271, 1258, 1148, 1109, 1032, 941, 858, 762, 752, 733, 704, 691 cm^{-1}.

UV/Vis (MeOH): λ_{max} (log ε) = 332 (2.88), 376 (2.69), 478 nm (2.57).

HRMS (ESI): [$C_{18}H_{14}N_2O^{130}Te+H^+$]: calculated: 405.0242; observed: 405.0243.

Synthesis of 4a:

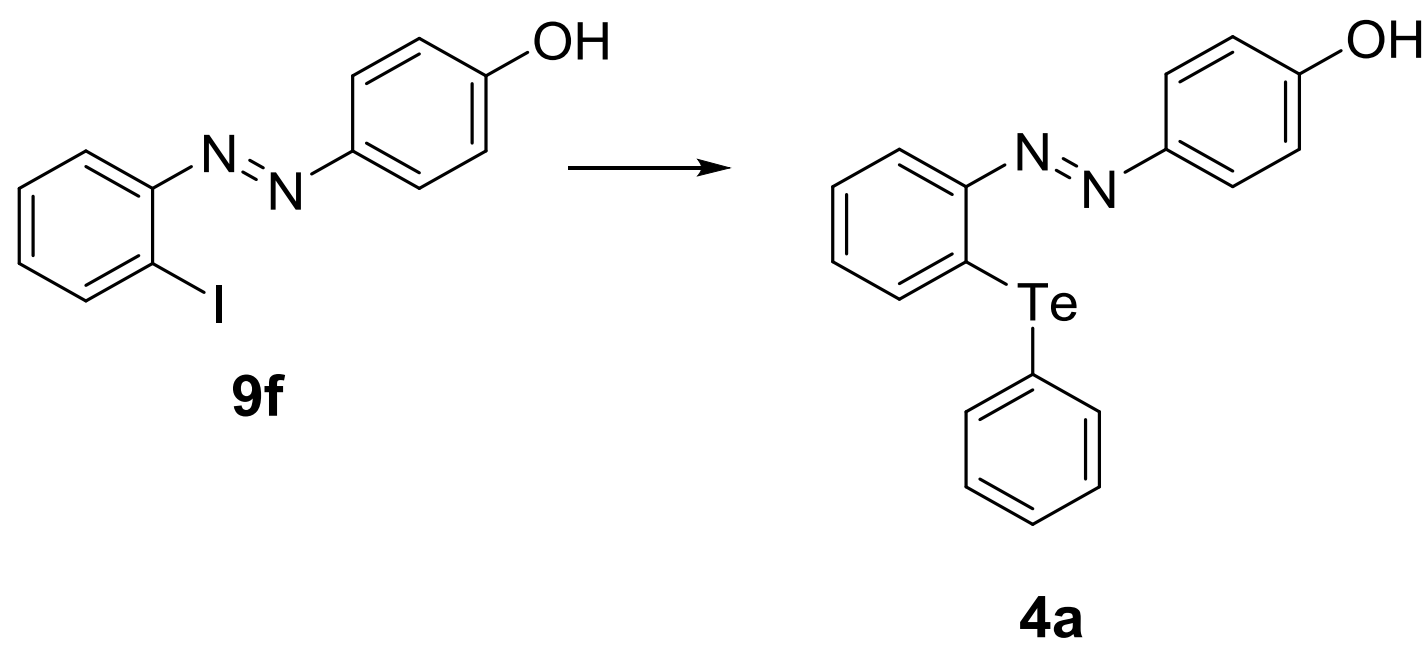

Azoarene **4a** was synthesized according to the general procedure (*B*). The crude product was purified via column chromatography.

Yield: 250 mg, 0.62 mmol, 39%

R_f (DCM/toluene 99:1) = 0.3

M.p.: 136 °C

^{1}H NMR (400 MHz, $CDCl_3$): δ = 8.15 (dd, $^3J_{H,H}$ = 7.9 Hz, 4*J*H,H = 1.4 Hz, 1 H, $C_{ar}H$), 8.01 (d, $^3J_{H,H}$ = 8.9 Hz, 2 H, $C_{ar}H$), 7.92 (dd, $^3J_{H,H}$ = 8.2 Hz, $^4J_{H,H}$ = 1.4 Hz, 2 H, $C_{ar}H$), 7.44 (tt, $^3J_{H,H}$ = 7.4 Hz, $^4J_{H,H}$ = 1.4 Hz, 1 H, $C_{ar}H$), 7.41–7.33 (m, 3 H, $C_{ar}H$), 7.18 (dd, $^3J_{H,H}$ = 8.0 Hz, $^4J_{H,H}$ = 1.3 Hz, 1 H, $C_{ar}H$), 7.06 (dt, $^3J_{H,H}$ = 7.4 Hz, $^4J_{H,H}$ = 1.5 Hz, 1 H, $C_{ar}H$), 6.96 ppm (d, $^3J_{H,H}$ = 8.9 Hz, 2 H, $C_{ar}H$).

^{13}C NMR (101 MHz, $CDCl_3$): δ = 158.5 (q, C_{ar}), 151.9 (q, C_{ar}), 145.6 (q, C_{ar}), 140.6 (t, $C_{ar}H$), 133.9 (t, $C_{ar}H$), 132.8 (t, $C_{ar}H$), 130.2 (t, $C_{ar}H$), 129.7 (t, $C_{ar}H$), 128.8 (t, $C_{ar}H$), 126.6 (t, $C_{ar}H$), 124.6 (t, $C_{ar}H$), 122.2 (q, C_{ar}), 116.2 (t, $C_{ar}H$), 116.2 ppm (q, C_{ar}).

^{125}Te NMR (126 MHz, $CDCl_3$): δ = 724 ppm.

IR (ATR): $\tilde{\nu}$ = 3327, 3059, 2922, 1587, 1501, 1447, 1395, 1277, 1225, 1148, 1103, 1016, 999, 837, 768, 756, 733,723, 692 cm^{-1}.

UV/Vis (MeOH): λ_{max} (log ε) = 354 (3.11), 443 nm (2.67).

HRMS (ESI): [$C_{18}H_{14}N_2O^{130}Te+H^+$]: calculated: 405.0242; observed: 405.0243.

Synthesis of 5:

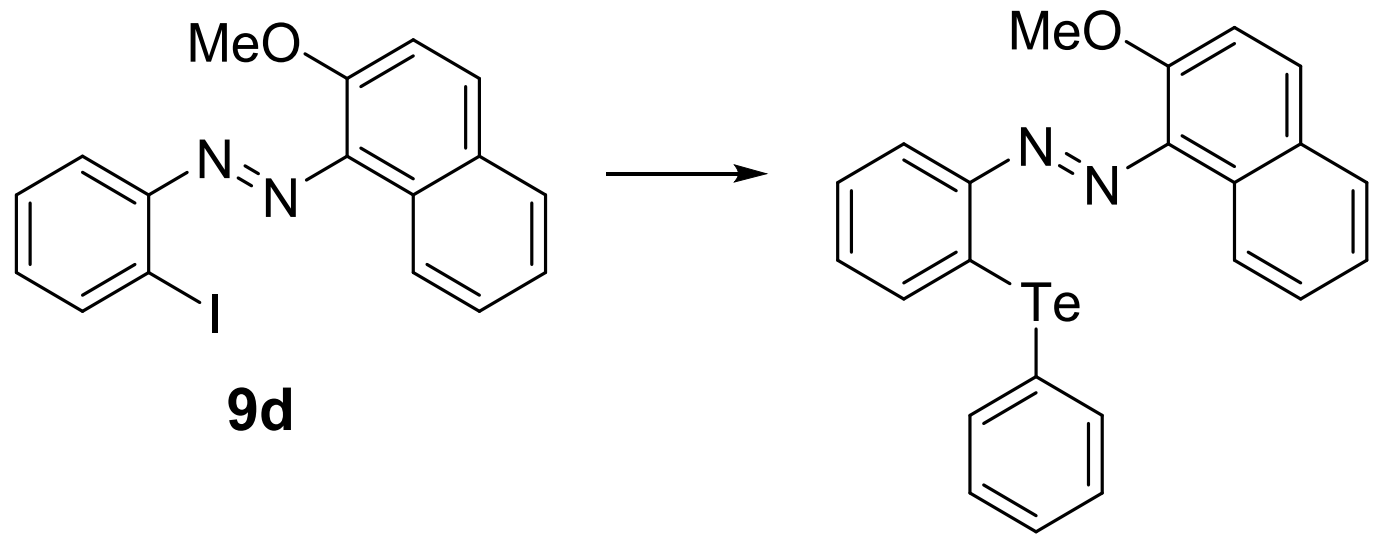

Azoarene **5** was synthesized according to the general procedure (*B*). The crude product was purified via column chromatography.

Yield: 21 mg, 0.05 mmol, 5%

R_f (*n*-hexane/ethyl acetate 9:1) = 0.2

M.p.: 102 °C

^{1}H NMR (600 MHz, $CDCl_3$): δ = 8.49 (d, $^3J_{H,H}$ = 8.7 Hz, 1 H, $C_{ar}H$), 8.32 (dd, $^3J_{H,H}$ = 7.9 Hz, $^4J_{H,H}$ = 1.4 Hz, 1 H, $C_{ar}H$), 7.97–7.89 (m, 3 H, $C_{ar}H$), 7.84 (d, $^3J_{H,H}$ = 8.1 Hz, 1 H, $C_{ar}H$), 7.54 (dt, $^3J_{H,H}$ = 7.8 Hz, $^4J_{H,H}$ = 1.3 Hz, 1 H, $C_{ar}H$), 7.48–7.40 (m, 4 H, $C_{ar}H$), 7.35 (t, $^3J_{H,H}$ = 7.4 Hz, 2 H, $C_{ar}H$7.29 (dd, $^3J_{H,H}$ = 7.9 Hz, $^4J_{H,H}$ = 1.2 Hz, 1 H, $C_{ar}H$), 7.13 (dt, $^3J_{H,H}$ = 7.6 Hz, $^4J_{H,H}$ = 1.5 Hz, 1 H, $C_{ar}H$), 4.05 ppm (s, 3 H, O-CH_3*)*.

^{13}C NMR (151 MHz, $CDCl_3$): δ = 152.5 (q, C_{ar}), 150.0 (q, C_{ar}), 140.7 (t, $C_{ar}H$), 134.9 (q, C_{ar}), 134.1 (t, $C_{ar}H$), 133.4 (t, $C_{ar}H$), 131.4 (t, $C_{ar}H$), 130.5 (t, $C_{ar}H$), 129.6 (t, $C_{ar}H$), 129.4 (q, C_{ar}), 128.7 (t, $C_{ar}H$), 128.3 (t, $C_{ar}H$), 128.0 (t, $C_{ar}H$), 126.4 (t, $C_{ar}H$), 124.7 (t, $C_{ar}H$), 123.7 (t, $C_{ar}H$), 122.1 (q, C_{ar}), 116.5 (q, C_{ar}), 114.5 (t, $C_{ar}H$), 57.4 ppm (p, O-CH_3).

^{125}Te NMR (189 MHz, $CDCl_3$): δ = 746 ppm.

IR (ATR): $\tilde{\nu}$ = 3053, 2959, 2920, 2851, 1620, 1591, 1508, 1433, 1339, 1254, 1150, 1082, 1065, 1016, 806, 762, 735 cm^{-1}.

UV/Vis (MeOH): λ_{max} (log ε) = 376 (2.79), 452 nm (2.76).

HRMS (ESI): [$C_{23}H_{18}N_2O^{130}Te+H^+$]: calculated: 469.0555; observed: 469.0556.

Synthesis of 1-S:

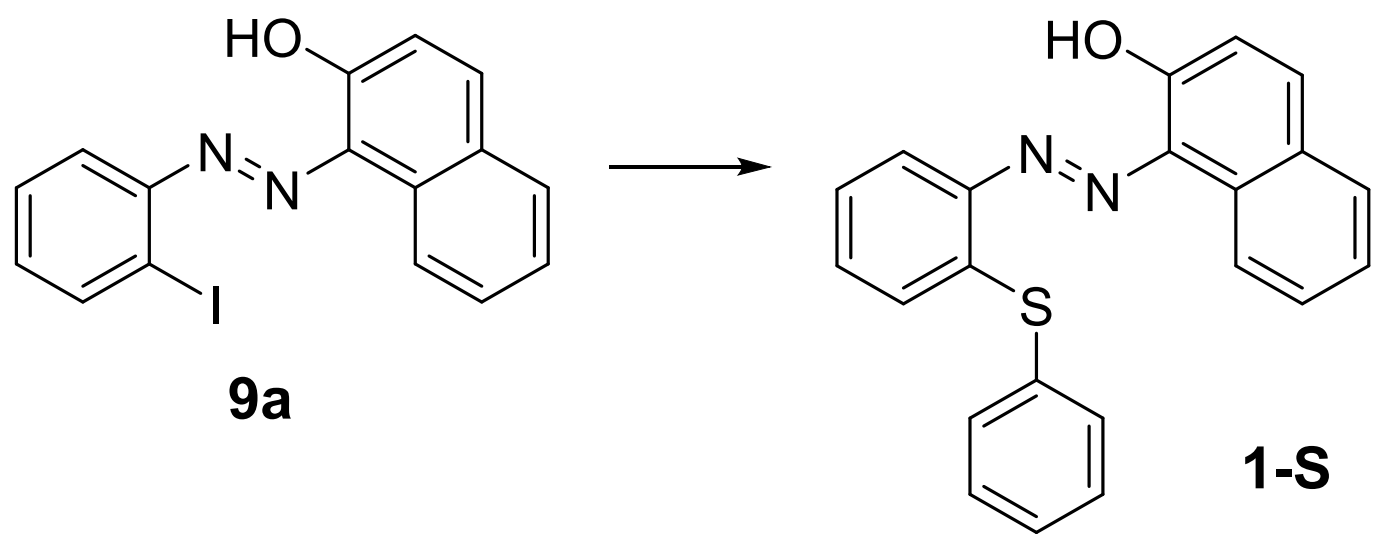

Azoarene **1-S** was synthesized according to the general procedure (*C*). The crude product was purified via column chromatography.

Yield: 180 mg, 0.49 mmol, 49%

R_f (DCM) = 0.4

M.p.: 134 °C

^{1}H NMR (600 MHz, $CDCl_3$): δ = 16.24 (s, 1 H, OH), 8.53 (d, $^3J_{H,H}$ = 8.0 Hz, 1 H, C_{ar}H), 8.15 (dd, $^3J_{H,H}$ = 8.1 Hz, $^4J_{H,H}$ = 1.4 Hz, 1 H, C_{ar}H), 7.67 (d, $^3J_{H,H}$ = 9.6 Hz, 1 H, C_{ar}H), 7.58–7.53 (m, 2 H, C_{ar}H), 7.52–7.45 (m, 2 H, C_{ar}H), 7.39 (dt, $^3J_{H,H}$ = 7.5 Hz, $^4J_{H,H}$ = 1.5 Hz, 1 H, C_{ar}H), 7.36 (dd, $^3J_{H,H}$ = 8.2 Hz, $^4J_{H,H}$ = 1.4 Hz, 2 H, C_{ar}H), 7.27 (t, $^3J_{H,H}$ = 7.2 Hz, 2 H, C_{ar}H), 7.24–7.17 (m, 2 H, C_{ar}H), 6.80 ppm (d, $^3J_{H,H}$ = 9.5 Hz, 1 H, C_{ar}H).

^{13}C NMR (151 MHz, $CDCl_3$): δ = 174.0 (q, C_{ar}), 145.0 (q, C_{ar}), 140.7 (q, C_{ar}), 135.2 (t, C_{ar}H), 134.9 (q, C_{ar}), 133.6 (q, C_{ar}), 130.8 (q, C_{ar}), 129.9 (t, C_{ar}H), 129.8 (t, C_{ar}H), 129.2 (t, C_{ar}H), 129.0 (t, C_{ar}H), 128.7 (t, C_{ar}H), 128.3 (q, C_{ar}), 126.9 (t, C_{ar}H), 126.9 (t, C_{ar}H), 126.1 (t, C_{ar}H), 125.5 (t, C_{ar}H), 125.1 (q, C_{ar}), 122.0 (t, C_{ar}H), 116.7 ppm (t, C_{ar}H).

IR (ATR): $\tilde{\nu}$ = 3055, 2988, 2901, 1618, 1582, 1557, 1487, 1476, 1460, 1447, 1381, 1290, 1242, 1221, 1207, 1146, 1134, 1067, 1024, 986, 868, 831, 746, 721, 691 cm^{-1}.

UV/Vis (MeOH): λ_{max} (log ε) = 304 (2.74), 487 nm (3.02).

HRMS (ESI): [$C_{22}H_{16}N_2OS+H^+$]: calculated: 357.1056; observed: 357.1056.

Synthesis of 1-Se:

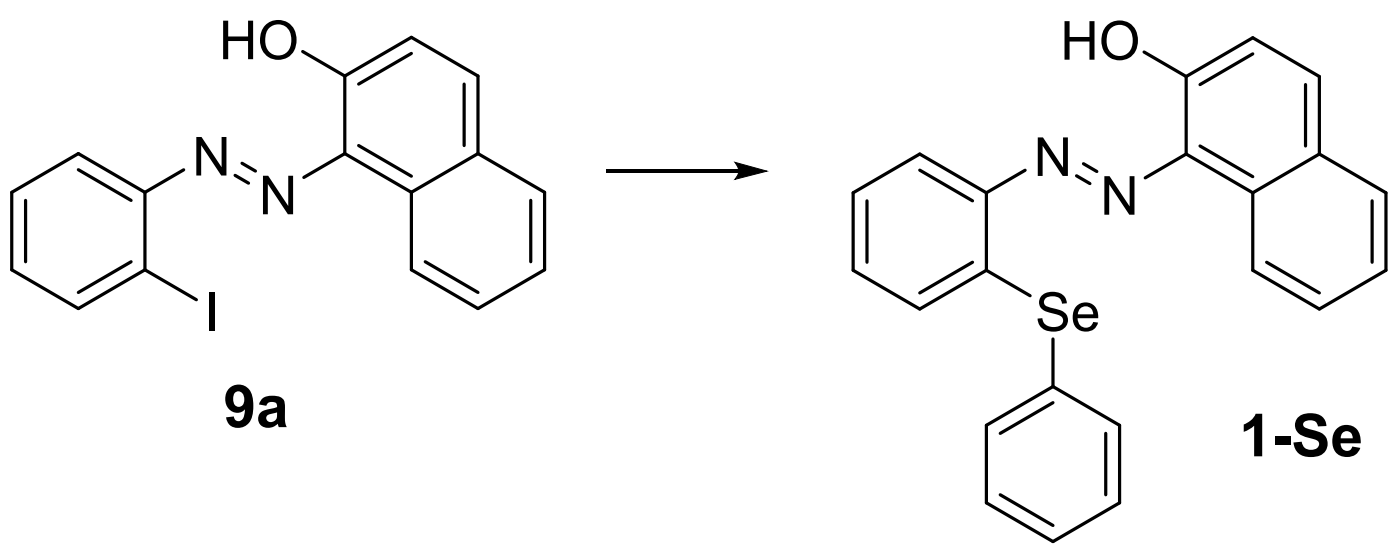

Azoarene **1-Se** was synthesized according to the general procedure (*C*). The crude product was purified via column chromatography.

Yield: 125 mg, 0.31 mmol, 31%

R_f (DCM) = 0.4

M.p.: 125 °C

^{1}H NMR (600 MHz, $CDCl_3$): δ = 16.00 (s, 1 H, OH), 8.61 (d, $^3J_{H,H}$ = 8.3 Hz, 1 H, C_{ar}H), 8.08 (dd, $^3J_{H,H}$ = 8.2 Hz, $^4J_{H,H}$ = 1.4 Hz, 1 H, C_{ar}H), 7.71 (d, $^3J_{H,H}$ = 9.5 Hz, 1 H, C_{ar}H), 7.60 (d, $^3J_{H,H}$ =7.9 Hz, 1 H, C_{ar}H), 7.58–7.51 (m, 4 H, C_{ar}H), 7.45 (dt, $^3J_{H,H}$ = 7.8 Hz, $^4J_{H,H}$ = 1.4 Hz, 1 H, C_{ar}H), 7.40 (dt, $^3J_{H,H}$ = 7.5 Hz, 4*J*H,H = 1.3 Hz, 1 H, C_{ar}H), 7.30–7.25 (m, 3 H, C_{ar}H), 7.17 (dt, $^3J_{H,H}$ =7.6, $^4J_{H,H}$ = 1.4 Hz, 1 H, C_{ar}H), 6.89 ppm (d, $^3J_{H,H}$ = 9.5 Hz, 1 H, C_{ar}H).

^{13}C NMR (151 MHz, $CDCl_3$): δ = 170.6 (q, C_{ar}), 145.8 (q, C_{ar}), 140.0 (t, C_{ar}H), 135.8 (t, C_{ar}H), 133.7 (q, C_{ar}), 133.3 (t, C_{ar}H), 130.8 (q, C_{ar}), 130.3 (q, C_{ar}), 129.6 (t, C_{ar}H), 129.4 (t, C_{ar}H), 129.0 (t, C_{ar}H), 128.8 (t, C_{ar}H), 128.4 (q, C_{ar}), 127.9 (t, C_{ar}H), 127.8 (t, C_{ar}H), 126.0 (t, C_{ar}H), 124.8 (t, C_{ar}H), 124.0 (q, C_{ar}), 122.2 (t, C_{ar}H), 118.3 ppm (t, C_{ar}H).

^{77}Se NMR (114 MHz, $CDCl_3$): δ = 352 ppm.

IR (ATR): $\tilde{\nu}$ = 3055, 2988, 2972, 2901, 1616, 1591, 1582, 1574, 1562, 1557, 1495, 1487, 1476, 1449, 1437, 1379, 1292, 1242, 1221, 1207, 1146, 1022, 986, 870, 862, 816, 746, 689 cm^{-1}.

UV/Vis (MeOH): λ_{max} (log ε) = 302 (2.80), 485 nm (3.10).

HRMS (ESI): [$C_{22}H_{16}N_2O^{80}Se+H^+$]: calculated: 405.0502; observed: 405.0500.

Synthesis of *m*-10:

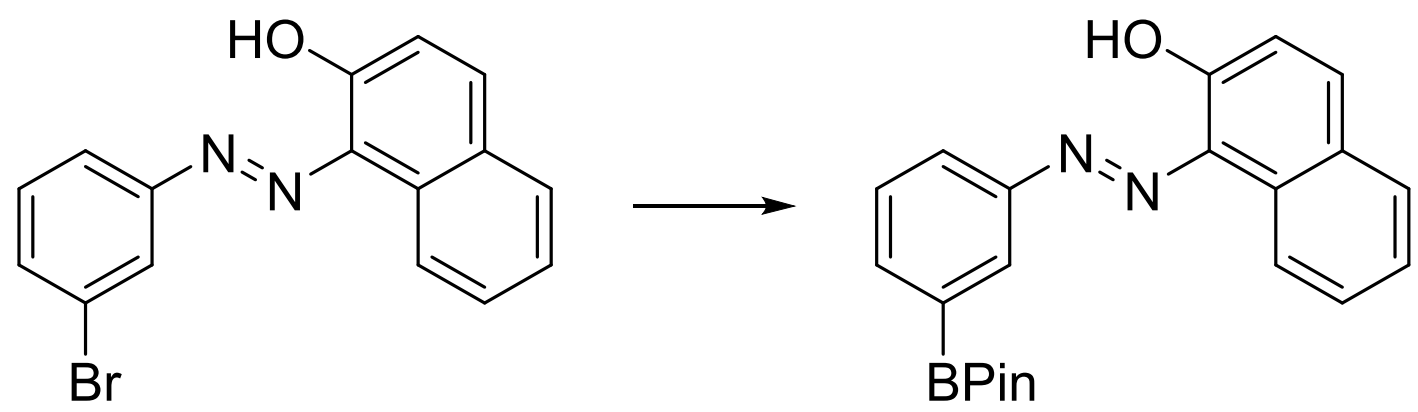

m-**9b** *m*-**10**

Azoarene *m*-**10** was synthesized according to the general procedure (*D*). The crude product was purified via column chromatography.

Yield: 117 mg, 0.31 mmol, 31%

R_f (*n*-hexane/ethyl acetate 9:1) = 0.4

M.p.: 128 °C

^{1}H NMR (600 MHz, $CDCl_3$): δ = 16.27 (s, 1 H, OH), 8.59 (d, $^3J_{H,H}$ = 8.2 Hz, 1 H, C_{ar}H), 8.07 (s, 1 H, C_{ar}H), 7.93 (dd, $^3J_{H,H}$ = 8.1 Hz, $^4J_{H,H}$ = 1.1 Hz, 1 H, C_{ar}H), 7.74 (d, $^3J_{H,H}$ = 7.3 Hz, 1 H, C_{ar}H), 7.71 (d, $^3J_{H,H}$ = 9.5 Hz, 1 H, C_{ar}H), 7.62–7.53 (m, 2 H, C_{ar}H), 7.50 (t, $^3J_{H,H}$ = 7.7 Hz, 1 H, C_{ar}H), 7.39 (dt, $^3J_{H,H}$ = 7.6 Hz, $^4J_{H,H}$ = 1.1 Hz, 1 H, C_{ar}H), 6.88 (d, $^3J_{H,H}$ = 9.4 Hz, 1 H, C_{ar}H), 1.39 ppm (s, 12 H, CH_3).

^{13}C NMR (151 MHz, $CDCl_3$): δ = 171.6 (q, C_{ar}), 144.3 (q, C_{ar}), 139.9 (t, C_{ar}H), 133.8 (t, C_{ar}H), 133.6 (q, C_{ar}), 130.1 (q, C_{ar}), 129.1 (t, C_{ar}H), 128.8 (t, C_{ar}H), 128.6 (t, C_{ar}H), 128.0 (q, C_{ar}), 125.6 (t, C_{ar}H), 125.6 (t, C_{ar}H), 124.8 (t, C_{ar}H), 121.8 (t, C_{ar}H), 120.5 (t, C_{ar}H), 84.2 (q, C_{ar}), 24.9 ppm (p, CH_3).

IR (ATR): $\tilde{\nu}$ = 3063, 2976, 1622, 1601, 1582, 1566, 1557, 1501, 1487, 1466, 1450, 1379, 1352, 1319, 1294, 1248, 1223, 1207, 1167, 1136, 1096, 1067, 986, 962, 868, 843, 827, 797, 781, 754, 710, 700, 692, 673, 660 cm^{-1}.

UV/Vis (MeOH): λ_{max} (log ε) = 307 (2.85), 423 (3.04), 481 nm (3.16).

HRMS (ESI): [$C_{22}H_{23}BN_2O_3$+H^+]: calculated: 375.1878; observed: 375.1878.

Synthesis of *p*-10:

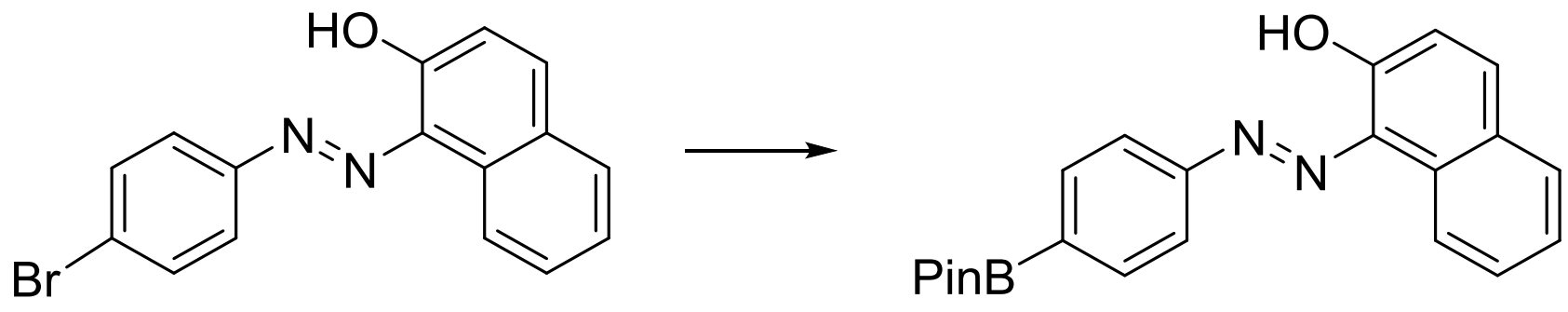

p-**9b** *p*-**10**

Azoarene *p*-**10** was synthesized according to the general procedure (*D*). The crude product was purified via column chromatography.

Yield: 130 mg, 0.35 mmol, 35%

R_f (*n*-hexane/ethyl acetate 9:1) = 0.4

M.p.: 158 °C

^{1}H NMR (600 MHz, $CDCl_3$): δ = 16.36 (s, 1 H, OH), 8.52 (d, $^3J_{H,H}$ = 8.3 Hz, 1 H, C_{ar}H), 7.89 (d, $^3J_{H,H}$ = 8.3 Hz, 2 H, C_{ar}H), 7.69 (d, $^3J_{H,H}$ = 9.5 Hz, 1 H, C_{ar}H), 7.66 (d, $^3J_{H,H}$ = 8.3 Hz, 2 H, C_{ar}H), 7.58–7.53 (m, 2 H, C_{ar}H), 7.39 (t, $^3J_{H,H}$ = 7.6 Hz, 1 H, C_{ar}H), 6.79 (d, $^3J_{H,H}$ = 9.5 Hz, 1 H, C_{ar}H), 1.37 ppm (s, 12 H, CH_3).

^{13}C NMR (151 MHz, $CDCl_3$): δ = 175.9 (q, C_{ar}), 146.0 (q, C_{ar}), 141.4 (t, C_{ar}H), 136.4 (t, C_{ar}H), 133.7 (q, C_{ar}), 130.6 (q, C_{ar}), 129.3 (t, C_{ar}H), 128.9 (t, C_{ar}H), 128.3 (q, C_{ar}), 126.3 (t, C_{ar}H), 125.9 (t, C_{ar}H), 122.1 (t, C_{ar}H), 117.1 (t, C_{ar}H), 84.1 (q, C_{ar}), 25.0 ppm (p, CH_3).

IR (ATR): $\tilde{\nu}$ = 3055, 2988, 2913, 1616, 1591, 1582, 1557, 1497, 1487, 1476, 1449, 1439, 1381, 1312, 1290, 1242, 1221, 1207, 1146, 1134, 1032, 1022, 986, 870, 862, 829, 818, 799, 689 cm^{-1}.

UV/Vis (MeOH): λ_{max} (log ε) = 310 (2.78), 426 (2.89), 484 nm (3.16).

HRMS (ESI): [$C_{22}H_{23}BN_2O_3$+H^+]: calculated: 375.1878; observed: 375.1880.

Synthesis of *m*-2a:

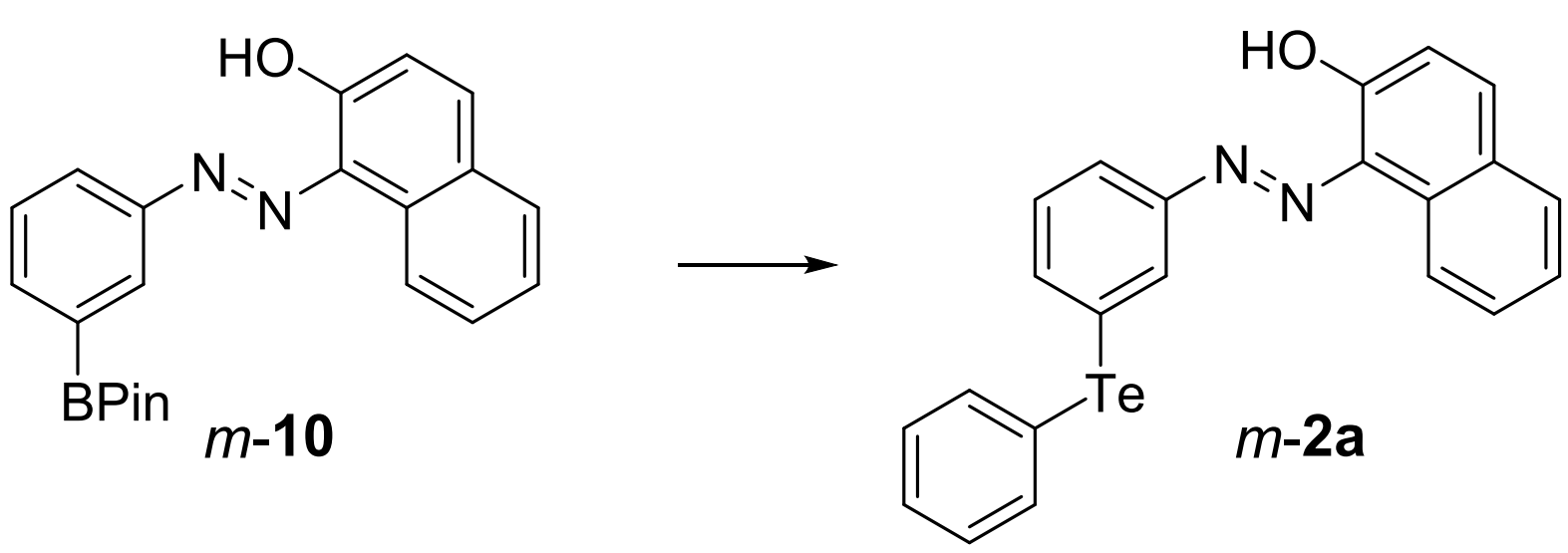

m-**10** *m*-**2a**

Azoarene *m*-**2a** was synthesized according to the general procedure (*E*). The crude product was purified via column chromatography.

Yield: 78 mg, 0.17 mmol, 68%

R_f (DCM/ethyl acetate 9:1) = 0.5

M.p.: 137 °C

^{1}H NMR (600 MHz, CDCl$_3$): δ = 16.08 (s, 1 H, OH), 8.36 (d, $^{3}J_{H,H}$ = 8.3 Hz, 1 H, C$_{ar}$H), 7.97 (s, 1 H, C$_{ar}$H), 7.85 (d, $^{3}J_{H,H}$ = 7.6 Hz, 2 H, C$_{ar}$H), 7.70 (d, $^{3}J_{H,H}$ = 9.5 Hz, 1 H, C$_{ar}$H), 7.58 (d, $^{3}J_{H,H}$ = 8.0 Hz, 1 H, C$_{ar}$H), 7.56–7.50 (m, 3 H, C$_{ar}$H), 7.42–7.36 (m, 2 H, C$_{ar}$H), 7.33–7.26 (m, 3 H, C$_{ar}$H), 6.83 ppm (d, $^{3}J_{H,H}$ = 9.4 Hz, 1 H, C$_{ar}$H).

^{13}C NMR (151 MHz, CDCl$_3$): δ = 172.8 (q, C$_{ar}$), 145.4 (q, C$_{ar}$), 140.7 (t, C$_{ar}$H), 139.3 (t, C$_{ar}$H), 135.5 (t, C$_{ar}$H), 133.6 (q, C$_{ar}$), 130.4 (t, C$_{ar}$H), 130.4 (q, C$_{ar}$), 129.9 (t, C$_{ar}$H), 129.1 (t, C$_{ar}$H), 128.8 (t, C$_{ar}$H), 128.6 (t, C$_{ar}$H), 128.2 (q, C$_{ar}$), 126.1 (t, C$_{ar}$H), 125.9 (t, C$_{ar}$H), 125.1 (t, C$_{ar}$H), 122.0 (t, C$_{ar}$H), 118.3 (t, C$_{ar}$H), 117.0 (q, C$_{ar}$), 113.9 ppm (q, C$_{ar}$).

^{125}Te NMR (189 MHz, CDCl$_3$): δ = 709 ppm.

IR (ATR): $\tilde{\nu}$ = 3042, 1616, 1558, 1551, 1497, 1489, 1476, 1447, 1435, 1256, 1223, 1207, 1165, 1144, 1134, 1088, 986, 862, 839, 777, 758, 737, 691 cm^{-1}.

UV/Vis (MeOH): λ_{max} (log ε) = 481 nm (2.92).

HRMS (ESI): [C$_{22}$H$_{16}$N$_2$O^{130}Te+H$^+$]: calculated: 455.0399; observed: 455.0398.

Synthesis of *p*-2a:

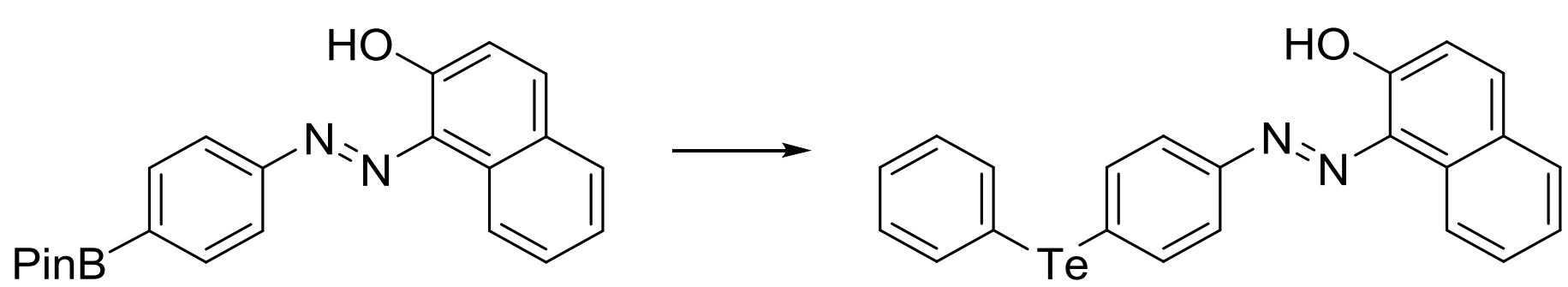

p-**10** *p*-**2a**

Azoarene *p*-**2a** was synthesized according to the general procedure (*E*). The crude product was purified via column chromatography.

Yield: 50 mg, 0.11 mmol, 44%

R$_f$ (DCM/ethyl acetate 9:1) = 0.5

M.p.: 123 °C

^{1}H NMR (600 MHz, CDCl$_3$): δ = 16.19 (s, 1 H, OH), 8.51 (d, $^{3}J_{H,H}$ = 8.3 Hz, 1 H, C$_{ar}$H), 7.77–7.73 (m, 4 H, C$_{ar}$H), 7.59 (d, $^{3}J_{H,H}$ = 7.8 Hz, 1 H, C$_{ar}$H), 7.57 (d, $^{3}J_{H,H}$ = 8.5 Hz, 2 H, C$_{ar}$H), 7.71 (d, $^{3}J_{H,H}$ = 9.3 Hz, 1 H, C$_{ar}$H), 7.54 (dt, $^{3}J_{H,H}$ = 7.8 Hz, $^{4}J_{H,H}$ = 1.3 Hz, 1 H, C$_{ar}$H), 7.39 (dt, $^{3}J_{H,H}$ = 7.5 Hz, $^{4}J_{H,H}$ = 1.2 Hz, 1 H, C$_{ar}$H), 7.33 (dt, $^{3}J_{H,H}$ = 7.4 Hz, $^{4}J_{H,H}$ = 1.3 Hz, 1 H, C$_{ar}$H), 7.25 (t, $^{3}J_{H,H}$ = 7.4 Hz, 2 H, C$_{ar}$H), 6.85 ppm (d, $^{3}J_{H,H}$ = 9.5 Hz, 1 H, C$_{ar}$H).

^{13}C NMR (151 MHz, CDCl$_3$): δ = 172.5 (q, C$_{ar}$), 144.6 (q, C$_{ar}$), 140.5 (t, C$_{ar}$H), 139.0 (t, C$_{ar}$H), 138.4 (t, C$_{ar}$H), 133.6 (q, C$_{ar}$), 130.4 (q, C$_{ar}$), 129.8 (t, C$_{ar}$H), 129.1 (t, C$_{ar}$H), 128.8 (t, C$_{ar}$H), 128.3

(t, $C_{ar}H$), 128.3 (q, C_{ar}), 126.1 (t, $C_{ar}H$), 125.0 (t, $C_{ar}H$), 121.9 (t, $C_{ar}H$), 119.5 (t, $C_{ar}H$), 114.7 (q, C_{ar}), 114.0 ppm (q, C_{ar}).

^{125}Te NMR (189 MHz, $CDCl_3$): δ = 695 ppm.

IR (ATR): $\tilde{\nu}$ = 3061, 3053, 1614, 1603, 1582, 1572, 1557, 1551, 1489, 1476, 1449, 1431, 1387, 1315, 1300, 1254, 1227, 1204, 1146, 1132, 1097, 1018, 986, 964, 947, 862, 843, 820, 781, 750, 727, 689 cm^{-1}.

UV/Vis (MeOH): λ_{max} (log ε) = 307 (2.79), 488 nm (3.15).

HRMS (ESI): [$C_{22}H_{16}N_2O^{130}Te+H^+$]: calculated: 455.0399; observed: 455.0399.

Photoproducts

The photoproducts were generated in situ by irradiation of solutions of the corresponding starting compounds with λ = 365 nm under aerobic conditions until complete consumption of the starting material. The products were not isolated and therefore no yields are given. Concentrations and irradiation times are specified in Figures S4–S8 (UV/vis) and Figures S18–S21 and S24 (NMR).

o-**2b:**

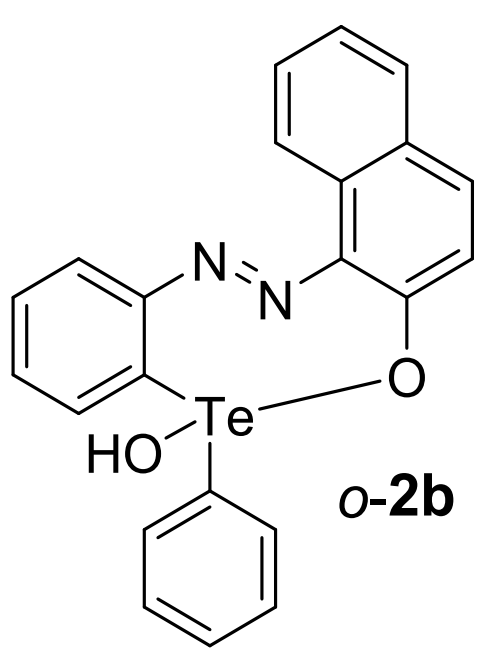

o-**2b**

^{1}H NMR (600 MHz, MeOD): δ = 9.97 (d, $^3J_{H,H}$ = 8.4 Hz, 1 H, $C_{ar}H$), 8.18 (d, $^3J_{H,H}$ = 8.2 Hz, 1 H, $C_{ar}H$), 8.05 (d, $^3J_{H,H}$ = 8.1 Hz, 1 H, $C_{ar}H$), 7.82 (dt, $^3J_{H,H}$ = 7.6 Hz, $^4J_{H,H}$ = 1.2 Hz, 1 H, $C_{ar}H$), 7.72 (d, $^3J_{H,H}$ = 9.4 Hz, 1 H, $C_{ar}H$), 7.64 (d, $^3J_{H,H}$ = 7.5 Hz, 1 H, $C_{ar}H$), 7.62–7.57 (m, 2 H, $C_{ar}H$), 7.50 (d, $^3J_{H,H}$ = 7.5 Hz, 2 H, $C_{ar}H$), 7.36 (t, $^3J_{H,H}$ = 7.3 Hz, 1 H, $C_{ar}H$),7.33 (t, $^3J_{H,H}$ = 7.0 Hz, 1 H, $C_{ar}H$), 7.29 (t, $^3J_{H,H}$ = 8.0 Hz, 2 H, $C_{ar}H$), 6.78 ppm (d, $^3J_{H,H}$ = 9.4 Hz, 1 H, $C_{ar}H$).

^{13}C NMR (151 MHz, MeOD): δ = 176.3 (q, C_{ar}), 154.7 (q, C_{ar}), 141.2 (t, $C_{ar}H$), 139.5 (q, C_{ar}), 134.0 (t, $C_{ar}H$), 132.2 (t, $C_{ar}H$), 131.9 (t, $C_{ar}H$), 131.6 (t, $C_{ar}H$), 131.3 (t, $C_{ar}H$), 130.7 (t, $C_{ar}H$), 130.6 (t, $C_{ar}H$), 130.2 (t, $C_{ar}H$), 129.5 (q, C_{ar}), 129.4 (q, C_{ar}), 129.3 (q, C_{ar}), 128.8 (t, $C_{ar}H$), 126.8 (t, $C_{ar}H$), 126.5 (t, $C_{ar}H$), 126.5 (t, $C_{ar}H$), 119.0 ppm (q, C_{ar}).

^{125}Te NMR (189 MHz, MeOD): δ = 1214 ppm.

UV/Vis (MeOH): λ_{max} = 403, 515, 547 nm.

HRMS (ESI): [$C_{22}H_{16}N_2O_2{}^{130}Te+H^+$]: calculated: 471.0348; observed: 471.0347.
[$C_{22}H_{15}N_2O^{130}Te^+$]: calculated: 453.0241; observed: 453.0242.

m-**2b:**

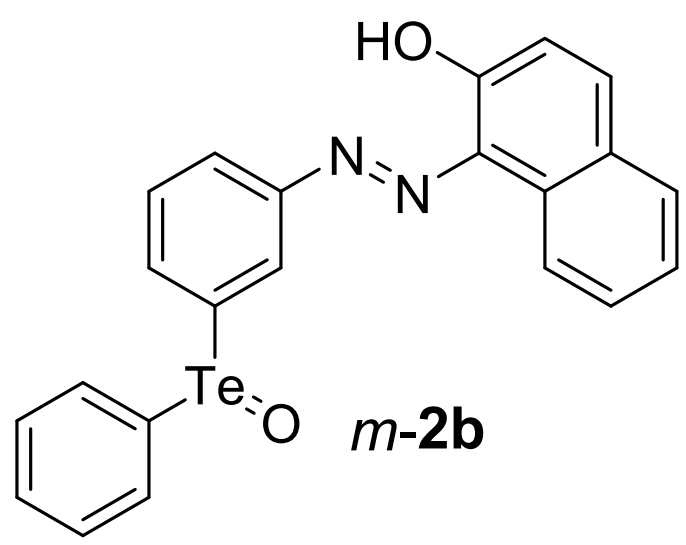

m-**2b**

^{1}H NMR (600 MHz, MeOD): δ = 8.43 (d, $^3J_{H,H}$ = 8.2 Hz, 1 H, C_{ar}H), 8.34 (t, $^4J_{H,H}$ = 1.5 Hz, 1 H, C_{ar}H), 8.00–7.94 (m, 2 H, C_{ar}H), 7.90–7.81 (m, 3 H, C_{ar}H), 7.69 (t, $^3J_{H,H}$ = 8.0 Hz, 1 H, C_{ar}H), 7.66 (d, $^3J_{H,H}$ = 7.7 Hz, 1 H, C_{ar}H), 7.65–7.58 (m, 3 H, C_{ar}H), 7.57 (t, $^3J_{H,H}$ = 7.8 Hz, 1 H, C_{ar}H), 7.43 (dt, $^3J_{H,H}$ = 7.4 Hz, $^4J_{H,H}$ = 1.0 Hz, 1 H, C_{ar}H), 6.84 ppm (d, $^3J_{H,H}$ = 9.4 Hz, 1 H, C_{ar}H).

^{13}C NMR (151 MHz, MeOD): δ = 171.1 (q, C_{ar}), 146.1 (q, C_{ar}), 140.7 (t, C_{ar}H), 137.1 (q, C_{ar}), 134.6 (q, C_{ar}), 133.2 (q, C_{ar}), 132.4 (t, C_{ar}H), 131.1 (t, C_{ar}H), 130.7 (t, C_{ar}H), 130.4 (t, C_{ar}H), 130.0 (q, C_{ar}), 129.5 (t, C_{ar}H), 128.8 (t, C_{ar}H), 128.5 (t, C_{ar}H), 128.4 (q, C_{ar}), 125.9 (t, C_{ar}H), 123.8 (t, C_{ar}H), 121.5 (t, C_{ar}H), 121.3 (t, C_{ar}H), 121.2 ppm (t, C_{ar}H).

^{125}Te NMR (189 MHz, MeOD): δ = 1090 ppm.

UV/Vis (MeOH): λ_{max} = 309, 478 nm.

HRMS (ESI): [$C_{22}H_{16}N_2O_2{}^{130}Te+H^+$]: calculated: 471.0348; observed: 471.0344.

p-**2b:**

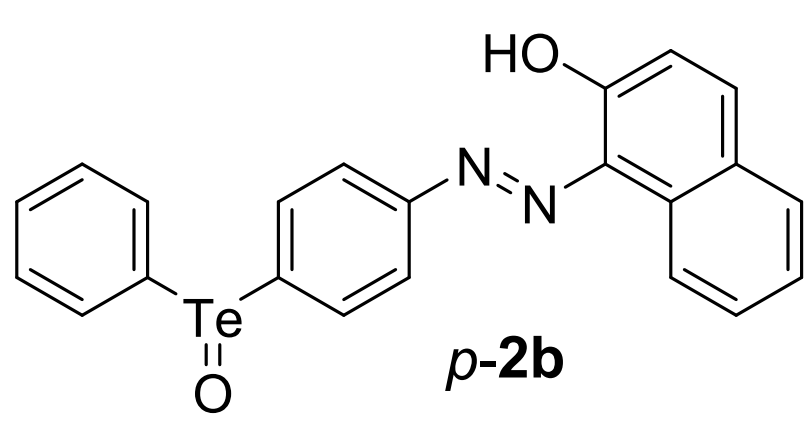

p-**2b**

^{1}H NMR (600 MHz, MeOD): δ = 8.48 (d, $^3J_{H,H}$ = 8.0 Hz, 1 H, C_{ar}H), 8.01 (d, $^3J_{H,H}$ = 8.2 Hz, 2 H, C_{ar}H), 7.92 (dd, $^3J_{H,H}$ = 8.2 Hz, $^4J_{H,H}$ = 1.7 Hz, 2 H, C_{ar}H), 7.87 (d, $^3J_{H,H}$ = 8.0 Hz, 2 H, C_{ar}H), 7.81 (d, $^3J_{H,H}$ = 9.9 Hz, 1 H, C_{ar}H), 7.62 (d, $^3J_{H,H}$ = 7.5 Hz, 1 H, C_{ar}H), 7.60–7.53 (m, 4 H, C_{ar}H), 7.42 (t, $^3J_{H,H}$ = 7.7 Hz, 1 H, C_{ar}H), 6.74 ppm (d, $^3J_{H,H}$ = 9.4 Hz, 1 H, C_{ar}H).

^{13}C NMR (151 MHz, MeOD): δ = 175.8 (q, C_{ar}), 146.1 (q, C_{ar}), 142.0 (t, $C_{ar}H$), 135.1 (q, C_{ar}), 133.9 (t, $C_{ar}H$), 133.2 (q, C_{ar}), 132.2 (t, $C_{ar}H$), 131.8 (q, C_{ar}), 131.0 (t, $C_{ar}H$), 130.5 (q, C_{ar}), 129.4 (t, $C_{ar}H$), 129.1 (t, $C_{ar}H$), 128.7 (t, $C_{ar}H$), 128.4 (q, C_{ar}), 126.4 (t, $C_{ar}H$), 124.9 (t, $C_{ar}H$), 121.7 (t, $C_{ar}H$), 118.0 ppm (t, $C_{ar}H$).

^{125}Te NMR (189 MHz, MeOD): δ = 1094 ppm.

UV/Vis (MeOH): λ_{max} = 303, 477 nm.

HRMS (ESI): [$C_{22}H_{16}N_2O_2{}^{130}Te+H^+$]: calculated: 471.0348; observed: 471.0345.

3b:

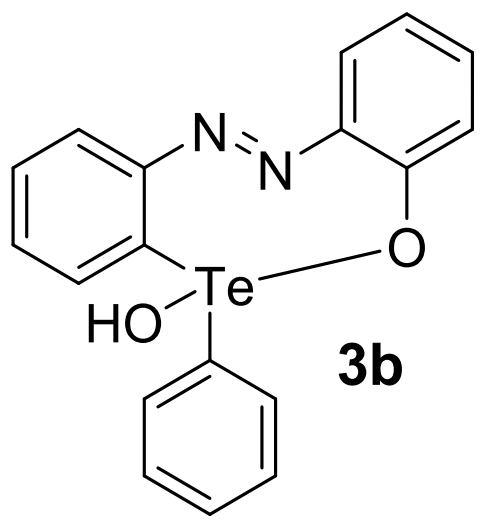

3b

^{1}H NMR (600 MHz, MeOD): δ = 8.20 (dd, $^3J_{H,H}$ = 7.9 Hz, $^4J_{H,H}$ = 1.0 Hz, 1 H, $C_{ar}H$), 8.10 (dd, $^3J_{H,H}$ = 7.8 Hz, $^4J_{H,H}$ = 1.2 Hz, 1 H, $C_{ar}H$), 7.91 (dd, $^3J_{H,H}$ = 8.8 Hz, $^4J_{H,H}$ = 1.6 Hz, 1 H, $C_{ar}H$), 7.86 (dt, $^3J_{H,H}$ = 7.5 Hz, $^4J_{H,H}$ = 1.4 Hz, 1 H, $C_{ar}H$), 7.72 (dt, $^3J_{H,H}$ = 7.4 Hz, $^4J_{H,H}$ = 1.2 Hz, 1 H, $C_{ar}H$), 7.52 (d, $^3J_{H,H}$ = 8.4 Hz, 2 H, $C_{ar}H$), 7.37 (dt, $^3J_{H,H}$ = 7.3 Hz, $^4J_{H,H}$ = 1.2 Hz, 1 H, $C_{ar}H$), 7.31 (t, $^3J_{H,H}$ = 7.7 Hz, 2 H, $C_{ar}H$), 7.28 (dt, $^3J_{H,H}$ = 7.7 Hz, $^4J_{H,H}$ = 1.7 Hz, 1 H, $C_{ar}H$), 6.71 (dd, $^3J_{H,H}$ = 8.8 Hz, $^4J_{H,H}$ = 1.0 Hz, 1 H, $C_{ar}H$), 6.49 ppm (dt, $^3J_{H,H}$ = 7.6 Hz, $^4J_{H,H}$ = 1.2 Hz, 1 H, $C_{ar}H$).

^{13}C NMR (151 MHz, MeOD): δ = 173.0 (q, C_{ar}), 153.5 (q, C_{ar}), 138.4 (t, $C_{ar}H$), 138.3 (q, C_{ar}), 137.0 (q, C_{ar}), 134.2 (t, $C_{ar}H$), 132.5 (t, $C_{ar}H$), 132.3 (t, $C_{ar}H$), 132.2 (t, $C_{ar}H$), 130.9 (t, $C_{ar}H$), 130.5 (t, $C_{ar}H$), 125.8 (t, $C_{ar}H$), 122.7 (q, C_{ar}), 120.5 (t, $C_{ar}H$), 117.7 ppm (t, $C_{ar}H$).

^{125}Te NMR (189 MHz, MeOD): δ = 1199 ppm.

UV/Vis (MeOH): λ_{max} = 376, 564 nm.

HRMS (ESI): [$C_{18}H_{14}N_2O_2{}^{130}Te+H^+$]: calculated: 421.0190; observed: 421.0194.

4b:

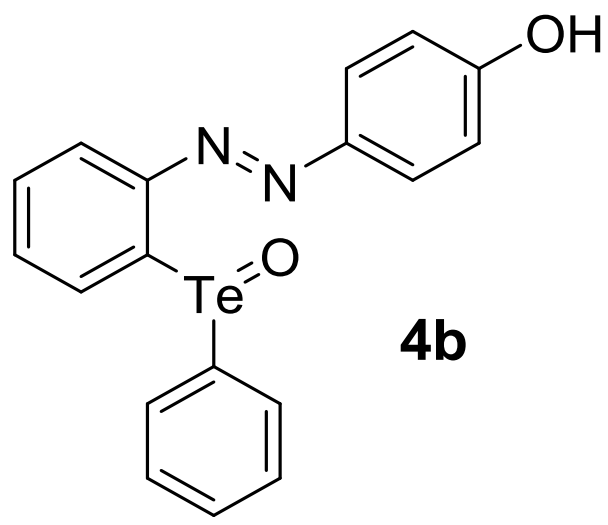

4b

^{1}H NMR (600 MHz, MeOD): δ = 8.10 (bs, 1 H, C_{ar}H), 8.07 (dd, $^{3}J_{H,H}$ = 8.1 Hz, $^{4}J_{H,H}$ = 1.0 Hz, 1 H, C_{ar}H), 7.89 (d, $^{3}J_{H,H}$ = 9.3Hz, 2 H, C_{ar}H), 7.82 (t, $^{3}J_{H,H}$ = 7.4 Hz, 1 H, C_{ar}H), 7.68 (t, $^{3}J_{H,H}$ = 6.9 Hz, 1 H, C_{ar}H), 7.61 (bs, 2 H, C_{ar}H), 7.50–7.38 (m, 3 H, C_{ar}H), 6.55 ppm (bs, 2 H, C_{ar}H).

^{13}C NMR (151 MHz, MeOD): δ = 155.7 (q, C_{ar}), 141.9 (t, C_{ar}H), 135.0 (t, C_{ar}H), 133.5 (t, C_{ar}H), 133.3 (t, C_{ar}H), 133.2 (t, C_{ar}H), 132.7 (q, C_{ar}), 131.6 (t, C_{ar}H), 130.9 (t, C_{ar}H), 130.7, 130.0 128.8, 125.8 (q, C_{ar}), 123.2, 121.6 (q, C_{ar}), 117.4 ppm.

^{125}Te NMR (189 MHz, MeOD): δ = 1194 ppm.

UV/Vis (MeOH): λ_{max} = 357, 525 nm.

HRMS (ESI): [$C_{18}H_{14}N_2O_2{}^{130}Te+H^+$]: calculated: 421.0190; observed: 421.0191.

$$K = \frac{[hydrazone]}{[azobenzene]} = \frac{\delta_{CO} - 147\ ppm}{180\ ppm - \delta_{CO}}$$ Eq. S1[5, 6]

Table S1: Tautomeric equilibrium for the investigated compounds.

Compound	Solvent	δ_{CO} [ppm]	K [-][a]
**1-O**	$CDCl_3$	176.2	7.7
**1-S**	$CDCl_3$	174.0	4.5
**1-Se**	$CDCl_3$	170.5	2.5
o-**2a**	MeOD	159.8	0.6
	$CDCl_3$	160.1	0.7
m-**2a**	MeOD	171.4	2.8
	$CDCl_3$	172.9	3.6
p-***2a***	MeOD	171.7	3.0
	$CDCl_3$	172.5	3.4
**3a**	MeOD	143.7	-0.1
	$CDCl_3$	152.4	0.2
**4a**	$CDCl_3$	158.5	0.5
**5**	$CDCl_3$	152.5	0.3

[a] For the phenol and methoxy derivatives, the formally calculated K values are listed for comparison only and should not be interpreted as physical azo–hydrazone equilibrium constants, as the underlying correlation was established for β-hydroxy azoarenes.

4. Computational Details

All calculations were performed using the program Gaussian 16[7]. The geometrical parameters of the investigated azoarenes *o*-**2** and **3** were optimized by means of PBE0[8] with additional dispersion correction with D3[9] and the basis sets def2-TZVP[10]. Furthermore, implicit methanol (MeOH) solvation using the solvation model based on density (SMD[11]) was employed. For all stationary points, no symmetry restriction was applied. Frequency calculations were carried out at each of the structures to verify the nature of the stationary point. It turned out that all structures have no imaginary frequencies. The NMR spectra of compounds *o*-**2** and **3** were computed by means of the Gauge-Independent Atomic Orbital (GIAO)[12] method (GIAO-PBE0-D3(SMD/def2-TZVP)). The ^{1}H and ^{13}C NMR shifts are referenced to TMS calculated using GIAO-B3LYP/6-311+G(2d,p).

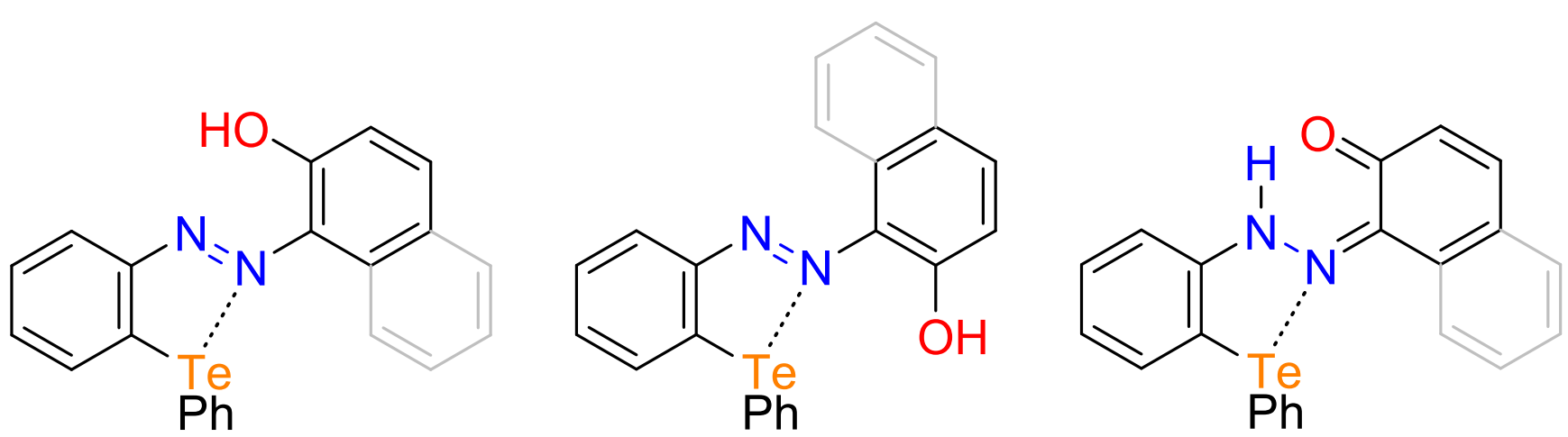

	I	II	III	IV
ΔE *o*-**2a**	0.8	5.8	**0.0**	8.0
ΔE **3a**	**0.0**	3.9	3.6	10.1

Figure S46. Tautomers and conformers of azoarenes *o*-**2a** and **3a** calculated by means of PBE0-D3(SMD,MeOH)/def2-TZVP. Energies are given in kcal/mol.

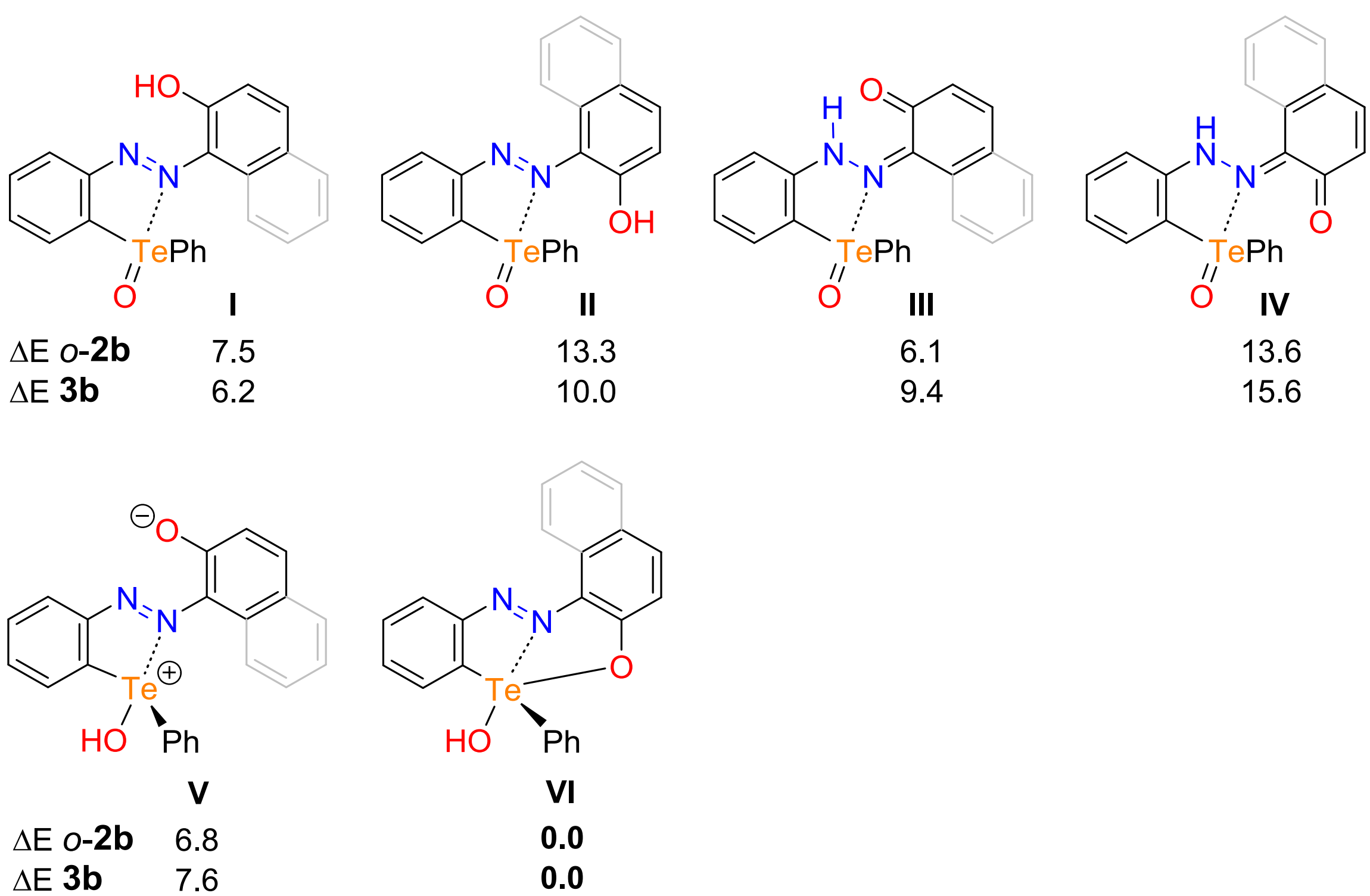

Figure S47. Tautomers and conformers of azoarenes *o*-**2b** and **3b** calculated by means of PBE0-D3(SMD,MeOH)/def2-TZVP. Energies are given in kcal/mol.

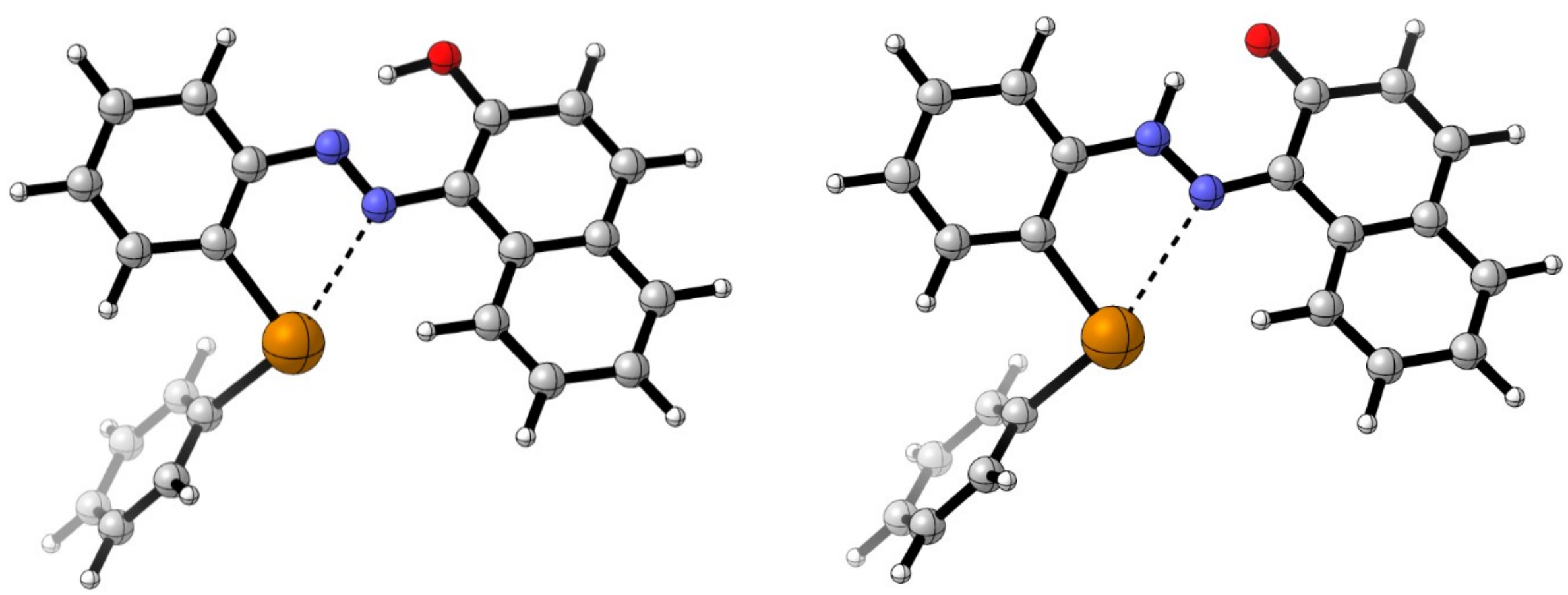

Figure S48. Molecular structures of *o*-**2a**-**I** (left) and *o*-**2a**-**III** (right) calculated by means of PBE0-D3(SMD,MeOH)/def2-TZVP.

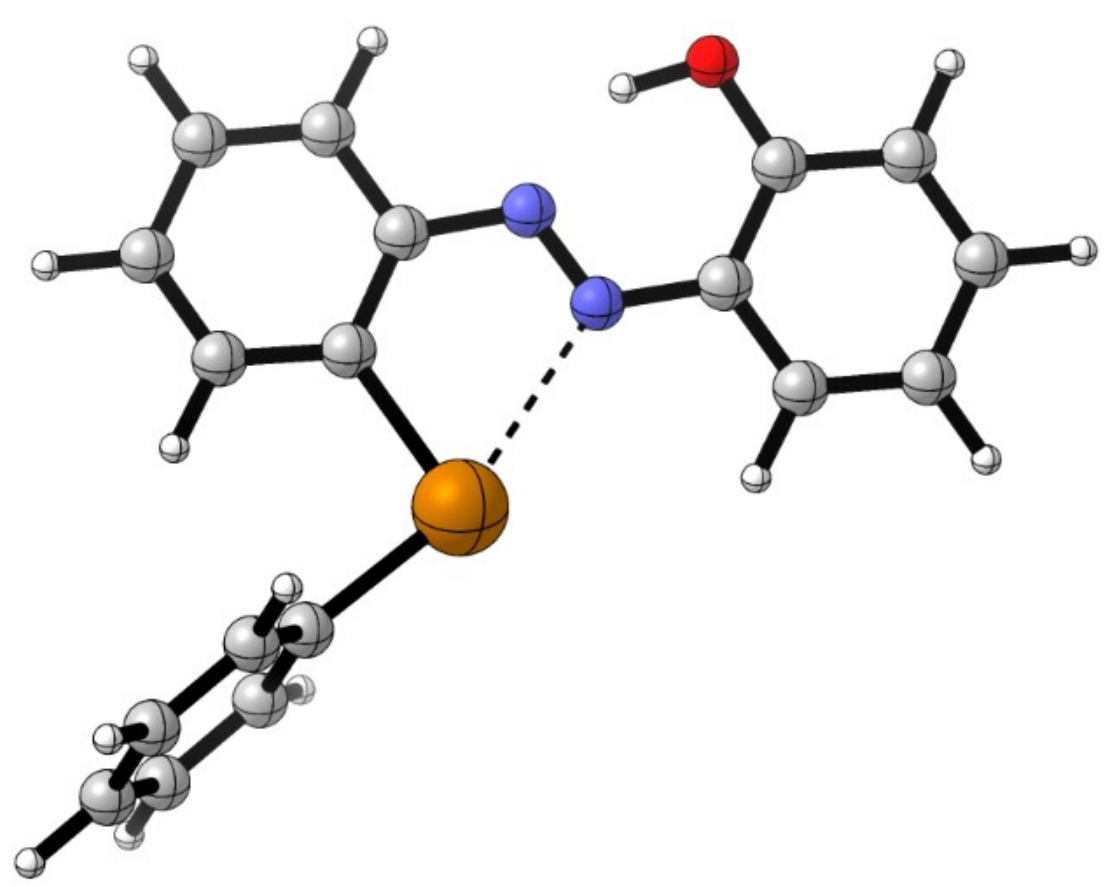

Figure S49. Molecular structures of **3a-I** (left) and **3a-III** (right) calculated by means of PBE0-D3(SMD,MeOH)/def2-TZVP.

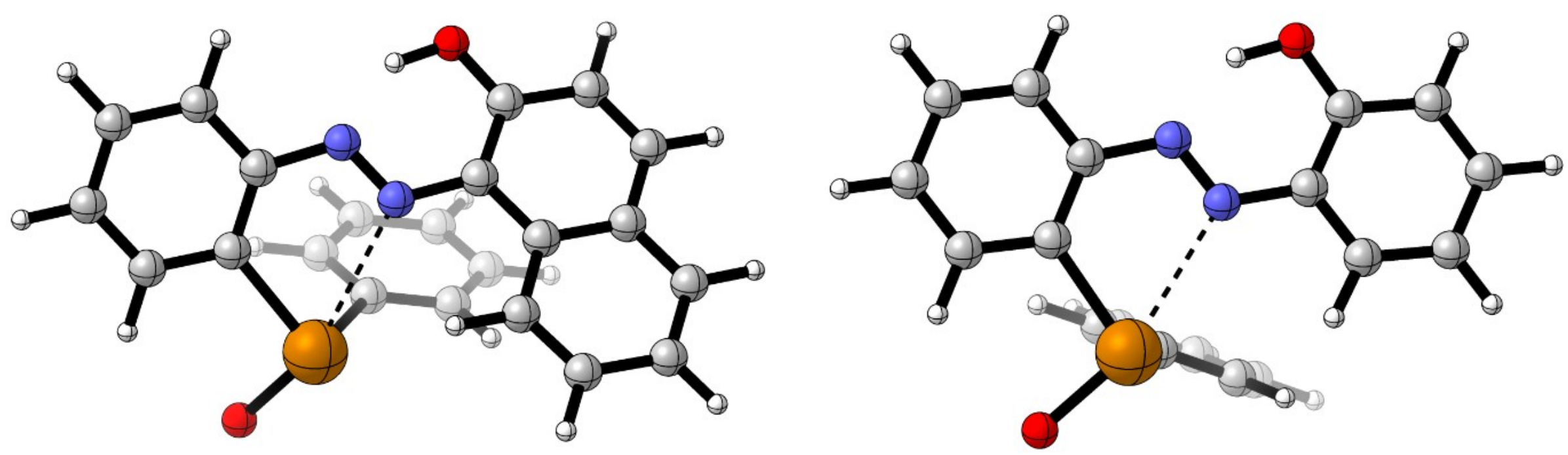

Figure S50. Molecular structures of *o*-**2b-I** (left) and **3b-I** (right) calculated by means of PBE0-D3(SMD,MeOH)/def2-TZVP.

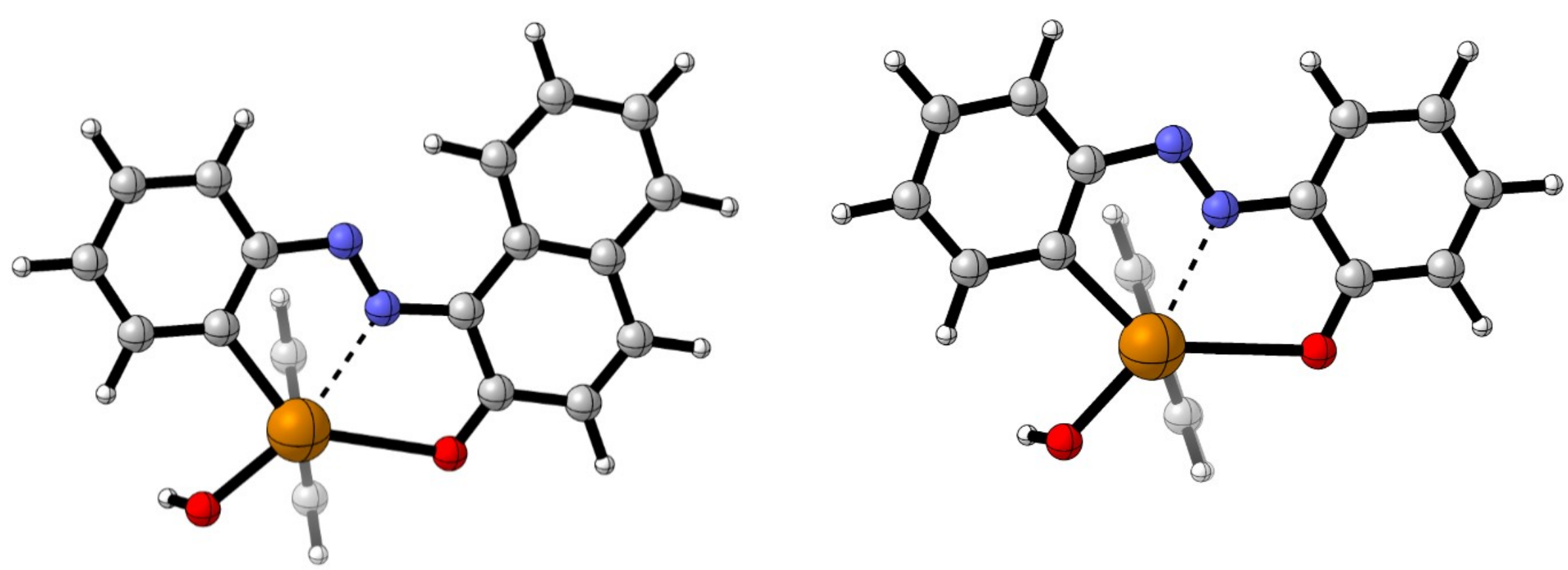

Figure S51. Molecular structures of *o*-**2b-VI** (left) and **3b-VI** (right) calculated by means of PBE0-D3(SMD,MeOH)/def2-TZVP.

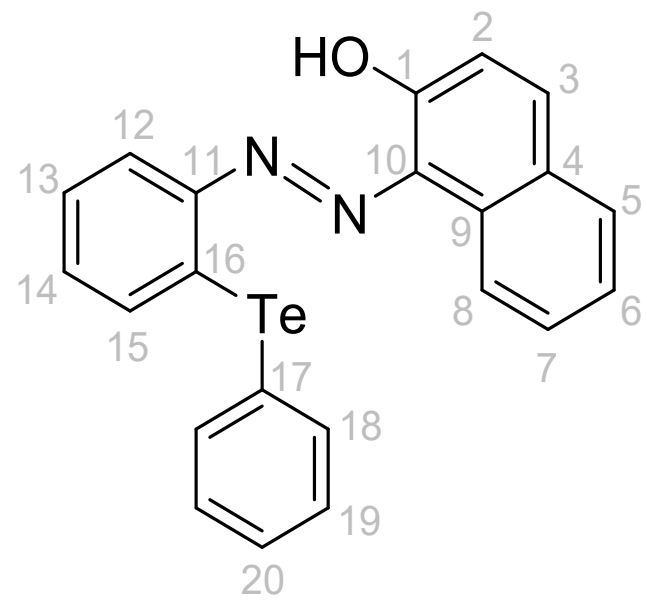

Figure S52. Molecular structure of motif *o*-**2** with assigned atomic numbers.

Table S2. Experimental and calculated (PBE0-D3(SMD,MeOH)/def2-TZVP) ^{1}H and ^{13}C chemical shifts of compounds *o*-**2a** and *o*-**2b**.

Atom	*o*-**2a**						*o*-**2b**			
	experimental		calculated *o*-**2a-I**		calculated *o*-**2a-III**		experimental		calculated o-**2b-VI**	
	^{1}H	^{13}C	^{1}H	^{13}C	^{1}H	^{13}C	^{1}H	^{13}C	^{1}H	^{13}C
1	-	159.8	-	154.0		178.5	-	176.4	-	176.1
2	7.1	122.6	8.0	118.7	7.5	126.0	6.8	126.5	7.4	124.9
3	7.9	138.5	8.8	138.6	8.7	144.9	7.7	141.3	8.5	142.9
4	-	130.2	-	127.6	-	126.9	-	129.4	-	126.7
5	7.8	130.0	8.8	130.0	8.5	130.5	7.7	130.7	8.5	130.7
6	7.5	126.5	8.3	124.1	8.2	125.3	7.4	126.5	8.1	124.3
7	7.7	129.9	8.6	129.5	8.5	130.3	7.6	131.3	8.5	130.9
8	8.9	123.7	10.1	121.8	9.7	121.2	10.0	126.8	11.1	123.7
9	-	134.1	-	132.1	-	133.4	-	129.5	-	127.4
10	-	131.6	-	128.1	-	128.6	-	129.3	-	123.7
11	-	150.8	-	149.1	-	140.1	-	154.8	-	151.3
12	8.0	127.2	8.8	132.1	8.2	122.2	8.2	128.9	9.0	127.7
13	7.5	129.3/137.6	8.1	126.2	8.0	126.6	7.8	134.0	8.6	133.3
14	7.1	130.8	7.8	128.5	7.6	125.4	7.6	130.3	8.3	128.3
15	7.5	129.3/137.6	7.6	134.1	7.5	134.5	8.0	131.6	8.8	129.1
16	-	113.9	-	138.7	-	136.2	-	119.0	-	119.6
17	-	118.8	-	142.8	-	141.0	-	139.6	-	149.3
18	7.9	141.3	8.3/8.8	139.5	8.5	140.2	7.5	132.3	7.0/9.0	126.0/134.3
19	7.3	131.0	8.2	129.4	8.3	129.7	7.3	130.7	7.6/8.2	128.9
20	7.5	130.1	8.3	129.6	8.2	130.0	7.3	131.9	8.0	130.6

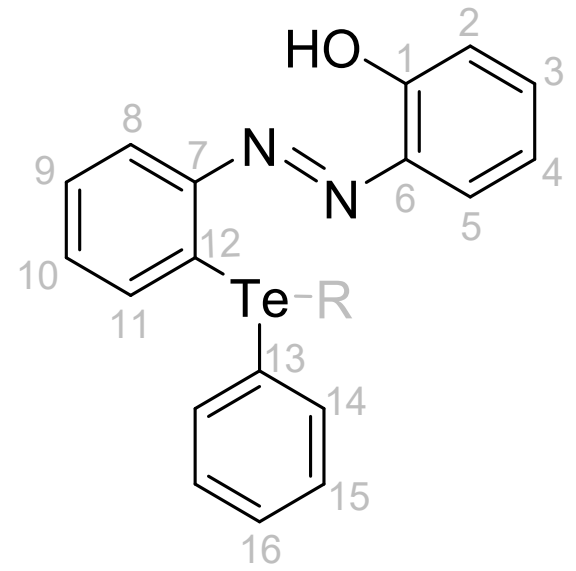

Figure S53. Molecular structure of motif **3** with assigned atomic numbers.

Table S3. Experimental and calculated (PBE0-D3(SMD,MeOH)/def2-TZVP) ^{1}H and ^{13}C chemical shifts of compounds **3a** and **3b.**

Atom	**3a**				**3b**			
	experimental		calculated (**3a-I**)		experimental		calculated (**3b-VI**)	
	^{1}H	^{13}C	^{1}H	^{13}C	^{1}H	^{13}C	^{1}H	^{13}C
1	-	153.9	-	153.8	-	172.9	-	173.4
2	8.1	133.2	7.8	117.1	6.7	125.9	7.2	123.4
3	7.4	134.7	8.2	135.4	7.2	138.4	8.0	138.7
4	7.1	131.6	7.8	118.6	6.5	117.8	7.1	113.9
5	8.2	130.4	9.3	131.8	7.9	120.6	8.7	119.2
6	-	143.7	-	134.4	-	136.9	-	132.2
7	-	152.0	-	147.4	-	153.7	-	150.4
8	7.1	119.7/121.5	8.8	134.4	8.2	130.5	8.9	129.5
9	7.4	124.7	8.1	125.8	7.8	134.3	8.6	133.4
10	7.1	119.7/121.5	7.8	129.4	7.7	132.3	8.4	130.7
11	7.2	135.6	7.4	132.5	8.1	132.3	8.7	129.9
12	-	119.7	-	140.8	-	122.7	-	123.9
13	-	122.9	-	142.4	-	138.3	-	147.6
14	7.9	141.8	8.5	139.9	7.5	132.5	6.8/8.9	126.0/135.0
15	7.4	130.9	8.3	129.4	7.3	131.0	7.7/8.2	129.0
16	7.5	130.2	8.3	129.8	7.4	132.3	8.0	131.0

5. Cartesian Coordinates and Absolute Energies for All Calculated Compounds

Table S4. Absolute energies [au] of all compounds calculated by means of PBE0-D3(SMD,MeOH)/def2-TZVP.

Compound	*E*	*G*
o-**2a-I**	-1299.922166	-1299.652930
o-**2a-II**	-1299.914104	-1299.645130
o-**2a-III**	-1299.923376	-1299.654071
o-**2a-IV**	-1299.910635	-1299.640879
o-**2b-I**	-1375.078263	-1374.806300
o-**2b-II**	-1375.068913	-1374.797197
o-**2b-III**	-1375.080451	-1374.808244
o-**2b-IV**	-1375.068415	-1374.795748
o-**2b-V**	-1375.079374	-1374.808029
o-**2b-VI**	-1375.090148	-1374.817798
**3a-I**	-1146.401547	-1146.175593
**3a-II**	-1146.395410	-1146.169920
**3a-III**	-1146.395809	-1146.171988
**3a-IV**	-1146.385409	-1146.159642
**3b-I**	-1221.556319	-1221.327330
**3b-II**	-1221.550295	-1221.321286
**3b-III**	-1221.551302	-1221.322700
**3b-IV**	-1221.541432	-1221.312675
**3b-V**	-1221.554131	-1221.327555
**3b-VI**	-1221.566229	-1221.336825

Cartesian coordinates of the optimized geometry for *o*-**2a-I** at PBE0-D3(SMD,MeOH)/def2-TZVP level of theory (number of imaginary frequencies = 0):

C	4.31892900	-3.15955700	-0.79406000
C	5.01111200	-2.25993200	-0.02581000
C	4.46846900	-0.99244900	0.26738100
C	3.18316300	-0.64458700	-0.22491000
C	2.50681600	-1.57940400	-1.03334600
C	3.06057300	-2.80625900	-1.30645200
H	6.17098900	-0.33078600	1.41667900
H	4.74239900	-4.13263000	-1.01645000
H	5.99275400	-2.50557000	0.36680000
C	5.19233400	-0.05105200	1.04016700
C	2.63979500	0.64380800	0.09380000
H	1.54816700	-1.31897300	-1.46178500
H	2.51993200	-3.50721000	-1.93359600
C	3.41919200	1.55882600	0.81924600
C	4.69057700	1.18722700	1.30122200
H	5.24627400	1.91667100	1.87963700
O	2.99059800	2.78463500	1.10475700
N	1.33579000	0.90517700	-0.28754400
N	0.93487200	2.09522600	-0.15471600
C	-0.40327100	2.34612200	-0.42470900
C	-1.40303100	1.35931500	-0.47924900
C	-0.73172800	3.69610900	-0.59831000
C	-2.70922500	1.76429100	-0.75046100
C	-2.02900400	4.07490800	-0.86680200
H	0.06483200	4.42926800	-0.52730800
C	-3.01995600	3.09962100	-0.94651800
H	-3.50174000	1.02700400	-0.80446300
H	-2.27320500	5.12083000	-1.01257500
H	-4.04622100	3.38097000	-1.15673100
Te	-0.95906400	-0.66175500	-0.11047700
C	-2.97102400	-1.19156800	0.34113800
C	-3.56610900	-0.76722300	1.52774300
C	-3.68739900	-2.00540900	-0.53204300
C	-4.86811100	-1.14209600	1.82872800
H	-3.01503800	-0.13747200	2.21878600

C	-4.98776000	-2.38815000	-0.22351000
H	-3.23582400	-2.33886400	-1.46071200
C	-5.57980800	-1.95440300	0.95420000
H	-5.32644500	-0.80322700	2.75197000
H	-5.53973300	-3.02173200	-0.90990200
H	-6.59599300	-2.24968300	1.19269600
H	2.09356500	2.87393000	0.65487400

Cartesian coordinates of the optimized geometry for *o*-**2a-II** at PBE0-D3(SMD,MeOH)/def2-TZVP level of theory (number of imaginary frequencies = 0):

C	5.72423600	0.81531600	-1.96157200
C	5.81045800	-0.31771800	-1.19702800
C	4.73806800	-0.72178500	-0.37496700
C	3.55325100	0.06730000	-0.31529500
C	3.48938000	1.21884600	-1.13093700
C	4.54516800	1.57626000	-1.93320100
H	5.74015200	-2.49851900	0.32472100
H	6.55280900	1.11815100	-2.59201900
H	6.70433000	-0.93313700	-1.21739100
C	4.82442000	-1.91869800	0.37539300
C	2.47898300	-0.38869700	0.51726400
H	2.59954800	1.83057500	-1.12230600
H	4.46361500	2.46270000	-2.55323800
C	2.60252800	-1.59374800	1.20535400
C	3.78413400	-2.35168700	1.14046700
H	3.84047500	-3.26929300	1.71448700
O	1.62649700	-2.07506700	1.99273700
N	1.24411500	0.23062200	0.68960800
N	1.17947500	1.47859400	0.59903200
C	-0.10209000	2.01090800	0.70285700
C	-1.29116900	1.27113100	0.56471900
C	-0.15177900	3.39277900	0.91990800
C	-2.50328500	1.94700200	0.68974900
C	-1.36078600	4.04274600	1.04467200
H	0.78791100	3.92917200	0.99782400
C	-2.54003400	3.31098900	0.93095500
H	-3.43692900	1.40522700	0.59279800
H	-1.39156300	5.11094800	1.22588400
H	-3.50003400	3.80752600	1.02365800
Te	-1.23616600	-0.78567100	0.13282600
C	-3.26688900	-0.87726200	-0.50799700
C	-4.21807300	-1.51280500	0.28535300
C	-3.64490500	-0.34511600	-1.73908900
C	-5.53582500	-1.61124500	-0.14625000
H	-3.93597400	-1.92821300	1.24738200
C	-4.96314900	-0.43675600	-2.16420900
H	-2.91049000	0.14762300	-2.36816700
C	-5.90955300	-1.07130200	-1.36886600
H	-6.27165400	-2.10679200	0.47845700
H	-5.25106100	-0.01585100	-3.12193900
H	-6.93862200	-1.14533800	-1.70387100
H	0.87248000	-1.46258800	1.98070100

Cartesian coordinates of the optimized geometry for *o*-**2a-III** at PBE0-D3(SMD,MeOH)/def2-TZVP level of theory (number of imaginary frequencies = 0):

C	4.28932400	-3.27041800	-0.60741700
C	5.03143900	-2.30927300	0.04269700
C	4.51495900	-1.02270200	0.25082200
C	3.21383600	-0.70279200	-0.19650500
C	2.48774200	-1.68764500	-0.87859500
C	3.01390900	-2.94801600	-1.07582100
H	6.29651500	-0.29765200	1.23995300

H	4.69489600	-4.26344300	-0.76503200
H	6.03190500	-2.53196100	0.39984100
C	5.30296600	-0.01930900	0.90064200
C	2.69628400	0.63066500	0.04712500
H	1.50887700	-1.45299600	-1.27546500
H	2.42982000	-3.69118400	-1.60812500
C	3.54729300	1.64653000	0.64716700
C	4.85693100	1.24219000	1.08265800
H	5.46877800	1.99907900	1.56121200
O	3.15580400	2.83159700	0.80812200
N	1.42032900	0.86097200	-0.25981500
N	0.94582100	2.04832200	-0.12325000
C	-0.39029900	2.35260900	-0.33839900
C	-1.40210000	1.38606700	-0.41045700
C	-0.69224600	3.71301400	-0.45585300
C	-2.70435900	1.82595400	-0.64497900
C	-1.99054600	4.12339500	-0.67106700
H	0.11445000	4.43565400	-0.38257900
C	-3.00061700	3.17242200	-0.77460000
H	-3.50796000	1.10252100	-0.71736200
H	-2.21508900	5.17957000	-0.76516600
H	-4.02538000	3.47873500	-0.95327600
Te	-1.00823400	-0.66524400	-0.11542900
H	1.61517800	2.77211100	0.20583500
C	-3.02795500	-1.17508800	0.29230400
C	-3.60698400	-0.84444400	1.51584300
C	-3.76949500	-1.88041500	-0.65152400
C	-4.91983500	-1.20545600	1.78507900
H	-3.03528700	-0.29980800	2.26002500
C	-5.08084500	-2.24884000	-0.37489800
H	-3.32885700	-2.13895500	-1.60860600
C	-5.65733500	-1.90923600	0.84087500
H	-5.36638400	-0.94070700	2.73778600
H	-5.65310400	-2.79769000	-1.11541500
H	-6.68206300	-2.19367400	1.05460200

Cartesian coordinates of the optimized geometry for *o*-**2a-IV** at PBE0-D3(SMD,MeOH)/def2-TZVP level of theory (number of imaginary frequencies = 0):

C	6.37330200	0.97782500	-0.61832800
C	6.18470200	-0.25214700	-0.01694500
C	4.90319700	-0.77824400	0.15507400
C	3.76909500	-0.03320900	-0.24480600
C	3.98901600	1.18178300	-0.90699900
C	5.26938300	1.67810700	-1.08937000
H	5.63255900	-2.63880400	0.99115000
H	7.37287100	1.37679300	-0.74780500
H	7.03651000	-0.83848500	0.31240600
C	4.73828700	-2.11183100	0.67144400
C	2.44999700	-0.61117900	-0.00827400
H	3.16993700	1.73038500	-1.35463000
H	5.40306200	2.61773200	-1.61376300
C	2.32907600	-2.05312800	0.28400900
C	3.54289800	-2.72714500	0.70323200
H	3.43891400	-3.75450300	1.03465900
O	1.25155400	-2.65611400	0.23681400
N	1.30457900	0.02599500	0.08825500
N	1.21078800	1.31540500	0.09002600
C	0.00201700	1.96697600	0.29947900
C	-1.23405800	1.30742700	0.30277500
C	0.07673600	3.34865000	0.49744600
C	-2.37432200	2.07714300	0.53523000
C	-1.06755600	4.08680300	0.71315800
H	1.05087800	3.82842700	0.48550700
C	-2.30126300	3.44514700	0.73593200

H	-3.34547400	1.59733200	0.55510500
H	-0.99795700	5.15723800	0.86899400
H	-3.21066900	4.00933500	0.91064800
Te	-1.38297200	-0.77402100	-0.03881200
C	-3.50534100	-0.74873500	-0.22340900
C	-4.30436400	-1.14125900	0.84783000
C	-4.10666100	-0.38923600	-1.42829100
C	-5.68780600	-1.16841400	0.71773400
H	-3.84850400	-1.42247300	1.79159600
C	-5.48918500	-0.41141400	-1.55515500
H	-3.49511300	-0.08586000	-2.27195300
C	-6.28096500	-0.80162300	-0.48241500
H	-6.30220500	-1.47439500	1.55807900
H	-5.94896000	-0.12637300	-2.49574600
H	-7.36087400	-0.82121300	-0.58338300
H	2.04247500	1.89931000	0.09880000

Cartesian coordinates of the optimized geometry for *o*-**2b-I** at PBE0-D3(SMD,MeOH)/def2-TZVP level of theory (number of imaginary frequencies = 0):

C	4.48389900	-2.09479600	-1.13386500
C	4.82935500	-0.85751900	-0.65435600
C	3.87303800	0.17363200	-0.56743700
C	2.53510800	-0.07325400	-0.96683500
C	2.21441800	-1.34310100	-1.48279300
C	3.16687400	-2.33052100	-1.55788900
H	5.25137400	1.63613600	0.21879500
H	5.22403000	-2.88446100	-1.19851700
H	5.84649000	-0.64951300	-0.33812200
C	4.22630100	1.46193000	-0.09203600
C	1.57043500	0.98181500	-0.85049600
H	1.21105800	-1.53700000	-1.83815800
H	2.89768000	-3.30183700	-1.95883400
C	1.98386000	2.25755100	-0.43162700
C	3.31821300	2.47472800	-0.03369800
H	3.58936800	3.46380300	0.31736300
O	1.14171400	3.27907800	-0.34727500
N	0.24654400	0.66081000	-1.04346800
N	-0.57553000	1.61093200	-1.12217800
C	-1.92665000	1.26022300	-1.17214000
C	-2.43708300	0.01451400	-0.78491800
C	-2.79652900	2.26353100	-1.59631000
C	-3.79899300	-0.21585900	-0.86393000
C	-4.15646100	2.01929400	-1.68113200
H	-2.38313900	3.22964800	-1.86496500
C	-4.65963100	0.77814600	-1.31925100
H	-4.19601700	-1.17346600	-0.54301300
H	-4.82400400	2.80121800	-2.02483900
H	-5.72470400	0.58232700	-1.37398600
Te	-1.24904600	-1.56703700	0.00066900
C	-0.55222200	-0.42489900	1.64989600
C	-1.41266700	0.43171600	2.32486900
C	0.76871200	-0.57104700	2.05558900
C	-0.93693600	1.16214300	3.40482300
H	-2.44625000	0.53889600	2.01187400
C	1.23733600	0.16241200	3.13750600
H	1.43731100	-1.24275100	1.52722800
C	0.38671200	1.03131700	3.80729100
H	-1.60211400	1.83823900	3.93125400
H	2.26962900	0.05605100	3.45295100
H	0.75502600	1.60860200	4.64846900
H	0.25702400	2.94373700	-0.68442200
O	-2.61821400	-2.49128400	0.80904000

Cartesian coordinates of the optimized geometry for *o*-**2b-II** at PBE0-D3(SMD,MeOH)/def2-TZVP level of theory (number of imaginary frequencies = 0):

C	4.69701800	2.01494500	0.46354300
C	4.94390500	0.81348000	-0.14388000
C	3.89446600	0.05751700	-0.70844500
C	2.56296900	0.56147600	-0.67867700
C	2.33964200	1.79465000	-0.02639000
C	3.37844900	2.49452200	0.53331700
H	5.17053800	-1.57595900	-1.30096800
H	5.50744700	2.58749700	0.90058100
H	5.95083100	0.41083900	-0.18897800
C	4.14814500	-1.21405500	-1.27135800
C	1.51888200	-0.25425800	-1.22046300
H	1.33823400	2.19459800	0.04101500
H	3.17450500	3.43259600	1.03846400
C	1.81062300	-1.52091700	-1.71251400
C	3.13590800	-1.98881200	-1.75437400
H	3.32898500	-2.96970600	-2.17721400
O	0.79922500	-2.27019200	-2.18656500
N	0.16409600	0.06958200	-1.17923200
N	-0.16680700	1.25349500	-1.37748400
C	-1.53646200	1.50377300	-1.19565300
C	-2.42940700	0.59738100	-0.61151900
C	-1.98267100	2.75930100	-1.59860500
C	-3.75763600	0.95028900	-0.46389200
C	-3.31686600	3.10316100	-1.45279000
H	-1.26629800	3.45167100	-2.02732300
C	-4.20440900	2.19969100	-0.88697500
H	-4.44642600	0.25302400	0.00192600
H	-3.66187000	4.07886700	-1.77542700
H	-5.24821600	2.46597000	-0.76200900
Te	-1.84662800	-1.32568500	0.08483100
C	-0.54121400	-0.57466400	1.58483900
C	-0.91184900	0.52761600	2.34480500
C	0.66905400	-1.21819100	1.81066100
C	-0.05170200	0.99824800	3.32751800
H	-1.86183600	1.02356200	2.17281100
C	1.52361500	-0.74310500	2.79686100
H	0.95624800	-2.07837500	1.21444000
C	1.16571200	0.36663700	3.55013800
H	-0.33198100	1.86421800	3.91766800
H	2.47323400	-1.23780800	2.97022200
H	1.83801600	0.74192000	4.31407000
H	1.12753300	-3.14156100	-2.44285500
O	-3.34201900	-1.62973100	1.12062500

Cartesian coordinates of the optimized geometry for *o*-**2b-III** at PBE0-D3(SMD,MeOH)/def2-TZVP level of theory (number of imaginary frequencies = 0):

C	4.48352400	-1.86436000	-1.33768000
C	4.82343700	-0.70926800	-0.66609600
C	3.87821600	0.30438400	-0.46421000
C	2.55903300	0.13852200	-0.93384700
C	2.24363700	-1.02321000	-1.64594300
C	3.18883200	-2.01175500	-1.83720800
H	5.26420000	1.61412000	0.55865400
H	5.21972300	-2.64536200	-1.49055200
H	5.83109700	-0.56618800	-0.28919100
C	4.24300000	1.52247500	0.20033800
C	1.58799100	1.18796200	-0.67452900
H	1.25197300	-1.14370300	-2.06249500
H	2.92116100	-2.90585700	-2.38982600
C	2.01768600	2.45962100	-0.09609500
C	3.37873000	2.54569700	0.36540000

H	3.67487200	3.47046900	0.84807100
O	1.22612400	3.41940100	0.04112000
N	0.31409700	0.90441100	-0.88030200
N	-0.58521500	1.81678200	-0.73550300
C	-1.94114700	1.52453400	-0.80435000
C	-2.45008300	0.22576100	-0.72762800
C	-2.81008000	2.60835700	-0.92362200
C	-3.81939700	0.03076200	-0.81260600
C	-4.17480200	2.39744800	-0.99988200
H	-2.40014300	3.61235500	-0.96147700
C	-4.68528100	1.10746300	-0.95434100
H	-4.21193800	-0.97812400	-0.73771200
H	-4.84134000	3.24677900	-1.09817800
H	-5.75402000	0.93686700	-1.01527600
Te	-1.27946000	-1.52057400	-0.39329000
C	-0.55449900	-0.84100500	1.48196500
C	-1.39177900	-0.15365100	2.35181600
C	0.76051400	-1.12133600	1.83277900
C	-0.89932500	0.26995300	3.57835400
H	-2.42033300	0.05823500	2.07815700
C	1.24636300	-0.69348100	3.06110700
H	1.41074100	-1.66066800	1.15210800
C	0.41851800	0.00456100	3.93003900
H	-1.54611400	0.81285700	4.25919700
H	2.27406700	-0.90439500	3.33641800
H	0.80079500	0.34300700	4.88700200
O	-2.65245300	-2.60569000	0.16344900
H	-0.25290800	2.76479300	-0.49230200

Cartesian coordinates of the optimized geometry for *o*-**2b-IV** at PBE0-D3(SMD,MeOH)/def2-TZVP level of theory (number of imaginary frequencies = 0):

C	5.81406200	1.19935600	0.38304500
C	5.49429000	-0.14659400	0.36654400
C	4.20951700	-0.57247400	0.03132500
C	3.20051700	0.37561200	-0.25243000
C	3.56009500	1.72659400	-0.28624500
C	4.84881800	2.13133000	0.02730800
H	4.72904900	-2.67173000	0.16060700
H	6.81651100	1.51965600	0.64285700
H	6.24837300	-0.89275300	0.59468800
C	3.93102300	-1.98176800	-0.09732800
C	1.86025500	-0.12607100	-0.54755200
H	2.86371300	2.48318000	-0.62525700
H	5.09815700	3.18552100	-0.01701300
C	1.68470100	-1.54883000	-0.93367700
C	2.76662200	-2.44704600	-0.58109400
H	2.59286600	-3.50576800	-0.73747500
O	0.65716400	-1.96294000	-1.46946100
N	0.72450600	0.49815500	-0.39543900
N	0.61935300	1.69022700	0.10782700
C	-0.62796700	2.24545000	0.36924300
C	-1.82090100	1.63329000	-0.01771900
C	-0.65602600	3.46041900	1.05350500
C	-3.02636900	2.24789600	0.28583800
C	-1.86697100	4.05847400	1.34911200
H	0.27848800	3.92326600	1.35477900
C	-3.05921800	3.45498800	0.96911800
H	-3.94873300	1.76554700	-0.02353100
H	-1.87763900	5.00228200	1.88265200
H	-4.00959900	3.92195700	1.20087200
Te	-1.92703900	-0.16969800	-1.13989600
C	-1.65930800	-1.47452200	0.51899900
C	-1.18960300	-1.05720500	1.75661600
C	-2.00064500	-2.80592000	0.30831200

C	-1.05737800	-1.97991000	2.78661400
H	-0.93209500	-0.01977500	1.93676200
C	-1.86955500	-3.72259300	1.34235600
H	-2.36652800	-3.13572300	-0.65925200
C	-1.39473100	-3.31070500	2.58055600
H	-0.69273100	-1.65334100	3.75473100
H	-2.13443300	-4.76142300	1.17716000
H	-1.28875000	-4.02822000	3.38704900
O	-3.76348800	-0.28178800	-1.24435600
H	1.42562500	2.17215000	0.49413800

Cartesian coordinates of the optimized geometry for *o*-**2b-V** at PBE0-D3(SMD,MeOH)/def2-TZVP level of theory (number of imaginary frequencies = 0):

C	-4.23475000	-2.54243500	0.27788300
C	-4.55013500	-1.24683400	-0.06783500
C	-3.58723100	-0.39454300	-0.62672000
C	-2.26474000	-0.85816300	-0.82803300
C	-1.98281100	-2.20302300	-0.51964100
C	-2.94285300	-3.02089900	0.03606700
H	-4.92385000	1.29341000	-0.84712700
H	-4.98597300	-3.19374600	0.71041300
H	-5.55569900	-0.86607200	0.08289700
C	-3.91045900	0.94064300	-1.01678200
C	-1.27868700	0.02351500	-1.38685700
H	-1.00745600	-2.61354800	-0.75413700
H	-2.69663700	-4.05167400	0.26821500
C	-1.62985400	1.34504400	-1.85717100
C	-2.99780100	1.75021900	-1.59589900
H	-3.25592300	2.76152300	-1.89353800
O	-0.82479200	2.15676900	-2.36800900
N	0.02952400	-0.32050300	-1.22073100
N	0.91874000	0.10212600	-2.01491300
C	2.21805500	-0.11893800	-1.56612300
C	2.51344900	-0.55601800	-0.26975600
C	3.26375400	0.12598800	-2.45457100
C	3.82020900	-0.78994600	0.11954900
C	4.57231700	-0.09389100	-2.06013500
H	3.02780500	0.48421700	-3.45051900
C	4.85450200	-0.55393700	-0.77953900
H	4.04003000	-1.13999200	1.12126300
H	5.38086200	0.09490200	-2.75740100
H	5.88046000	-0.72141200	-0.47238300
Te	0.89042000	-0.92446500	0.99316200
C	0.22661100	1.05062000	1.30815700
C	0.99997800	2.14565800	0.94801600
C	-1.03850000	1.20822200	1.86191500
C	0.48887900	3.42127200	1.14171500
H	1.98393200	2.01648300	0.51091200
C	-1.53742600	2.48933300	2.04965300
H	-1.63747000	0.34641000	2.13565600
C	-0.77671400	3.59249300	1.68662900
H	1.08175800	4.28348600	0.85718200
H	-2.52625400	2.62218000	2.47424700
H	-1.17306800	4.59211600	1.82694200
O	2.01925100	-0.96321300	2.62490000
H	2.51734500	-0.14221100	2.73420100

Cartesian coordinates of the optimized geometry for *o*-**2b-VI** at PBE0-D3(SMD,MeOH)/def2-TZVP level of theory (number of imaginary frequencies = 0):

C	-5.75281600	1.10163300	0.70113200
C	-5.52053500	-0.12907900	0.12703100
C	-4.22401000	-0.54345700	-0.20078200

C	-3.10832600	0.30344800	0.04807300
C	-3.37821000	1.55334800	0.63954000
C	-4.66793800	1.93700400	0.95617600
H	-4.91092000	-2.44311400	-0.96994000
H	-6.76095200	1.41229200	0.95151700
H	-6.34474300	-0.80409100	-0.08171300
C	-4.03200000	-1.83068400	-0.79105000
C	-1.79072700	-0.17886200	-0.31779200
H	-2.56263200	2.22383600	0.85161500
H	-4.82916100	2.90835800	1.41209800
C	-1.62693500	-1.49963100	-0.87811600
C	-2.80643700	-2.28685100	-1.11628800
H	-2.65940600	-3.26655000	-1.55722400
O	-0.49680900	-1.98014500	-1.16248100
N	-0.63001400	0.51348700	-0.20626400
N	-0.58325200	1.69296700	0.26235500
C	0.68175800	2.26616000	0.24340600
C	1.79912700	1.61666100	-0.28691400
C	0.82110900	3.54743900	0.78177800
C	3.03507200	2.24166900	-0.29535500
C	2.06017800	4.16064300	0.78629500
H	-0.05328700	4.04245200	1.19007400
C	3.16848500	3.51295000	0.24881200
H	3.89649000	1.74424800	-0.72582500
H	2.16587700	5.15422000	1.20768200
H	4.13798400	3.99824600	0.25044100
Te	1.44544700	-0.27069800	-1.13414300
C	1.67381500	-1.39257300	0.64196000
C	1.39531500	-0.85278600	1.88822400
C	2.12705200	-2.69983900	0.51374900
C	1.57369800	-1.63541800	3.02212500
H	1.04586600	0.16751000	1.99569700
C	2.30456400	-3.47226800	1.65249200
H	2.34077500	-3.11891300	-0.46359000
C	2.02681200	-2.94170300	2.90561500
H	1.35867600	-1.21636700	3.99906900
H	2.65693900	-4.49355700	1.55751200
H	2.16444100	-3.54873100	3.79374500
O	3.39604100	-0.54588300	-1.54987800
H	3.93553100	-0.58733800	-0.75015000

Cartesian coordinates of the optimized geometry for **3a-I** at PBE0-D3(SMD,MeOH)/def2-TZVP level of theory (number of imaginary frequencies = 0):

C	4.23402400	-2.61139700	-0.09885700
C	3.06149800	-1.88983200	-0.13993100
H	6.37448700	-2.49437300	0.09085000
C	5.44516100	-1.93613200	0.05646600
C	3.06916400	-0.49442300	-0.03025800
C	4.30221000	0.18238100	0.12280500
C	5.48023800	-0.55892600	0.16621100
H	6.41791600	-0.02740500	0.28576800
O	4.37092800	1.51379100	0.22912100
N	1.83264100	0.13762700	-0.07746600
N	1.82732400	1.39285000	-0.01062900
C	0.57958800	2.00113900	-0.04441700
C	-0.64111400	1.30053600	-0.07653100
C	0.60005600	3.40027900	-0.03737500
C	-1.81657500	2.04867900	-0.11120000
C	-0.57488600	4.11896400	-0.07104300
H	1.56392900	3.89723500	-0.00801800
C	-1.78653600	3.43386500	-0.10935500
H	-2.77590100	1.54619900	-0.13926200
H	-0.55434700	5.20256600	-0.06799500
H	-2.72136300	3.98345900	-0.13687200

Te	-0.68224400	-0.80215800	-0.06000900
C	-2.81484500	-0.91922700	0.03852500
C	-3.46761100	-0.87958600	1.26902400
C	-3.56258300	-1.08824200	-1.12460700
C	-4.85008800	-0.99779900	1.33376500
H	-2.89737700	-0.75041800	2.18349000
C	-4.94539300	-1.20862500	-1.05894000
H	-3.06712300	-1.12111300	-2.08971700
C	-5.58997800	-1.16220000	0.16988900
H	-5.35009000	-0.96249300	2.29610900
H	-5.51938500	-1.33777300	-1.97065300
H	-6.66953400	-1.25580200	0.22111800
H	3.43418800	1.84920600	0.16108100
H	4.21506200	-3.69109700	-0.18625100
H	2.10504200	-2.38635100	-0.26048800

Cartesian coordinates of the optimized geometry for **3a-II** at PBE0-D3(SMD,MeOH)/def2-TZVP level of theory (number of imaginary frequencies = 0):

N	-1.90271500	0.46451800	0.14259600
C	-3.18286200	-0.05724500	-0.07704500
C	-4.14660300	0.56528300	-0.87385900
H	-3.89804100	1.50989600	-1.34300100
C	-5.37512100	-0.02814500	-1.07847100
H	-6.11290100	0.45222700	-1.71061000
C	-5.65423500	-1.25630300	-0.48006400
H	-6.61654700	-1.73046500	-0.63951400
C	-4.71274000	-1.88497600	0.31389700
H	-4.92112400	-2.83731500	0.78842500
C	-3.47008900	-1.29524300	0.51881200
C	-0.47699100	2.18418400	0.11863900
C	0.67966600	1.38490700	0.19423200
C	-0.37537700	3.57979800	0.15663200
C	1.90959600	2.02435500	0.33465100
C	0.85017400	4.19329000	0.29989600
H	-1.29025600	4.15736600	0.07858900
C	1.99509200	3.40616600	0.39140600
H	2.81955200	1.43951900	0.39826200
H	0.92084700	5.27414100	0.33803900
H	2.96837100	3.87216900	0.50271800
N	-1.77244600	1.69753200	-0.02115300
Te	0.55246700	-0.70696000	0.05215100
C	2.65398100	-0.99545400	-0.16181800
C	3.26877100	-0.81660500	-1.39912200
C	3.41521300	-1.40713800	0.92909200
C	4.63253100	-1.03687000	-1.53967300
H	2.68454800	-0.49969500	-2.25703500
C	4.77910200	-1.63179500	0.78523000
H	2.94699100	-1.54850900	1.89783200
C	5.38845900	-1.44487600	-0.44789300
H	6.45336200	-1.61897500	-0.55945500
H	5.10529100	-0.89214100	-2.50549800
H	5.36587400	-1.95038400	1.64047000
O	-2.58896100	-1.93742700	1.31053900
H	-1.78784800	-1.40084800	1.41723700

Cartesian coordinates of the optimized geometry for **3a-III** at PBE0-D3(SMD,MeOH)/def2-TZVP level of theory (number of imaginary frequencies = 0):

C	4.33533300	-2.59127600	-0.02956000
C	3.14676000	-1.92638300	-0.04345000
H	6.48606300	-2.39000700	0.03143900
C	5.54294700	-1.85249900	0.02082900
C	3.11295000	-0.51028100	-0.00823200

C	4.34069700	0.26305100	0.04338200
C	5.55344500	-0.48679900	0.05650100
H	6.48405800	0.06942200	0.09477500
O	4.32288700	1.53141600	0.07487100
N	1.89453600	0.05807900	-0.02580200
N	1.81092800	1.33007500	0.00045700
C	0.58619100	1.97689000	-0.01335700
C	-0.63467300	1.28445100	-0.02903000
C	0.63051200	3.37358900	-0.00979900
C	-1.80073500	2.04841200	-0.04508200
C	-0.53883200	4.10228800	-0.02440900
H	1.59719600	3.86674700	0.00325900
C	-1.75863300	3.43293600	-0.04293300
H	-2.76484300	1.55489800	-0.05875000
H	-0.50321500	5.18538300	-0.02228500
H	-2.68755200	3.99233800	-0.05558000
Te	-0.71827600	-0.82568100	-0.02236400
H	2.72829400	1.84163500	0.03417800
C	-2.84477500	-0.91197200	0.01381700
C	-3.52684800	-0.92469300	1.22870600
C	-3.56272100	-1.00552800	-1.17633200
C	-4.91221800	-1.02183600	1.25145700
H	-2.97781600	-0.85389800	2.16197000
C	-4.94822500	-1.10353900	-1.15123000
H	-3.04186700	-0.99717300	-2.12818600
C	-5.62344800	-1.11070500	0.06200800
H	-5.43659300	-1.02867000	2.20124500
H	-5.50046600	-1.17396300	-2.08245400
H	-6.70533200	-1.18756600	0.08084000
H	4.36455600	-3.67381000	-0.05675400
H	2.20016300	-2.45498200	-0.08205500

Cartesian coordinates of the optimized geometry for **3a-IV** at PBE0-D3(SMD,MeOH)/def2-TZVP level of theory (number of imaginary frequencies = 0):

N	-1.97285400	0.40834800	-0.05484900
C	-3.20546200	-0.08446000	0.02614700
C	-4.38489600	0.69348000	0.23733400
H	-4.32354500	1.76839900	0.37165400
C	-5.60357500	0.10155800	0.29524600
H	-6.49339900	0.69662100	0.46347800
C	-5.72098200	-1.31176300	0.14464800
H	-6.70758300	-1.76195400	0.19254100
C	-4.63181500	-2.09560900	-0.04834100
H	-4.72045600	-3.17131300	-0.15726700
C	-3.29939900	-1.54339100	-0.11564900
C	-0.46645800	2.19719300	-0.03928900
C	0.68122700	1.39303500	-0.08561300
C	-0.37277200	3.59229900	-0.03162300
C	1.91532200	2.04307800	-0.13592000
C	0.85977300	4.20531800	-0.07730500
H	-1.28398200	4.18100100	0.00957900
C	2.01010200	3.42370600	-0.13201300
H	2.82521000	1.45703300	-0.17797600
H	0.92617300	5.28704300	-0.07228600
H	2.98770700	3.89116100	-0.17189900
N	-1.74819200	1.66820300	0.01008200
Te	0.55544100	-0.71436600	-0.07724400
C	2.66576800	-0.98438400	0.05212300
C	3.41238400	-1.23089400	-1.09763400
C	3.30475500	-0.96133100	1.29036900
C	4.78275100	-1.44426500	-1.01078000
H	2.92652100	-1.25129900	-2.06775900
C	4.67493500	-1.17099000	1.37525200
H	2.73368800	-0.77307800	2.19385700

C	5.41486800	-1.41270700	0.22483600
H	6.48484700	-1.57829600	0.29209400
H	5.35681400	-1.63394400	-1.91175300
H	5.16510500	-1.14740800	2.34294100
O	-2.30268800	-2.26879200	-0.28759800
H	-2.51036900	2.33831300	0.09293500

Cartesian coordinates of the optimized geometry for **3b-I** at PBE0-D3(SMD,MeOH)/def2-TZVP level of theory (number of imaginary frequencies = 0):

C	4.13088500	-0.49994700	-1.30928900
C	2.78015300	-0.26251900	-1.19932800
H	6.08802200	0.09360800	-0.64047400
C	5.02139300	0.27497200	-0.56349300
C	2.29318800	0.74527000	-0.35658900
C	3.20444300	1.53600900	0.38486400
C	4.56792800	1.27780600	0.27168100
H	5.25664500	1.88496100	0.84845700
O	2.79616200	2.51639000	1.19333300
N	0.91795500	0.88931700	-0.30171900
N	0.45855700	1.83472000	0.37968400
C	-0.93533600	1.94100700	0.40251800
C	-1.80163500	1.03737800	-0.22634900
C	-1.45054200	3.02468700	1.10945200
C	-3.16609400	1.24271500	-0.14911400
C	-2.81971900	3.21926600	1.18357700
H	-0.75979300	3.70666700	1.59329000
C	-3.67801700	2.33056400	0.55349200
H	-3.83662200	0.53811000	-0.63025200
H	-3.21555600	4.06533600	1.73352200
H	-4.75114100	2.47622800	0.60840800
Te	-1.13955400	-0.68235000	-1.28983700
C	-0.53809900	-1.70704600	0.47396400
C	-1.28617500	-1.58564200	1.63858500
C	0.56859600	-2.54525700	0.42787200
C	-0.91129000	-2.29904600	2.76873400
H	-2.15681300	-0.93885900	1.67245600
C	0.93692700	-3.25756300	1.56192000
H	1.14824600	-2.64685600	-0.48322200
C	0.20044200	-3.13136500	2.73174600
H	-1.48944600	-2.20223400	3.68146800
H	1.80189100	-3.91128300	1.52917700
H	0.49177800	-3.68498900	3.61781200
H	1.80550400	2.55969600	1.11493100
O	-2.79908300	-1.44811500	-1.51984200
H	2.06084400	-0.84181000	-1.76675200
H	4.49873500	-1.27857300	-1.96641200

Cartesian coordinates of the optimized geometry for **3b-II** at PBE0-D3(SMD,MeOH)/def2-TZVP level of theory (number of imaginary frequencies = 0):

C	4.14295600	1.48780200	1.16508500
C	2.76786800	1.56055700	1.11440100
H	5.93581700	1.03421800	0.06393700
C	4.85377100	1.09854400	0.02914600
C	2.08485500	1.24506800	-0.06387100
C	2.80700900	0.85228600	-1.20182600
C	4.19507600	0.78441500	-1.14420600
H	4.73818400	0.48410300	-2.03307200
O	2.21176300	0.54783500	-2.36996100
N	0.69033000	1.22256600	-0.15850700
N	0.06895500	1.90248100	0.67955600
C	-1.32529200	1.77538500	0.64385500
C	-2.02610900	0.78945600	-0.06411500

C	-2.02417900	2.69670000	1.42125400
C	-3.40689200	0.76017500	-0.00319500
C	-3.40785900	2.66553400	1.47027200
H	-1.45878500	3.43485200	1.97944700
C	-4.10004400	1.69832500	0.75747700
H	-3.94819800	-0.01257100	-0.53910300
H	-3.94403700	3.39198400	2.07009200
H	-5.18303400	1.66036000	0.79768200
Te	-1.09509600	-0.71861400	-1.23844700
C	-0.11256500	-1.62411400	0.40836600
C	-0.79535000	-1.81985800	1.60201400
C	1.20131000	-2.04861700	0.25972100
C	-0.14606400	-2.43587000	2.66272600
H	-1.82490500	-1.49367300	1.70999600
C	1.84385700	-2.66521800	1.32535000
H	1.72960800	-1.89553200	-0.67580100
C	1.17204800	-2.85446600	2.52536500
H	-0.67064900	-2.58697700	3.60007800
H	2.87140200	-2.99491300	1.21647100
H	1.67658300	-3.33211300	3.35826500
H	1.25614500	0.68316700	-2.30201500
O	-2.55564700	-1.82584200	-1.38213100
H	2.18977500	1.83123800	1.98982700
H	4.66680600	1.71591800	2.08591300

Cartesian coordinates of the optimized geometry for **3b-III** at PBE0-D3(SMD,MeOH)/def2-TZVP level of theory (number of imaginary frequencies = 0):

C	-4.18874700	0.58268400	-1.27909500
C	-2.85411100	0.34634900	-1.19983500
H	-6.15018700	-0.00282000	-0.57923900
C	-5.08613900	-0.20588200	-0.50853900
C	-2.35376800	-0.68345400	-0.35603700
C	-3.26267100	-1.51390700	0.42724700
C	-4.65339100	-1.20755500	0.30791100
H	-5.34796800	-1.80635600	0.88699600
O	-2.83129700	-2.44090400	1.16525400
N	-1.03007000	-0.83295500	-0.32393700
N	-0.51474300	-1.76237700	0.38564300
C	0.86155400	-1.94969400	0.42311200
C	1.75132000	-1.09062700	-0.22768500
C	1.33377200	-3.04095000	1.14824100
C	3.10835200	-1.35317200	-0.15625600
C	2.69414900	-3.28496600	1.21347100
H	0.62598900	-3.69085200	1.65218200
C	3.58482500	-2.44568300	0.55931400
H	3.79918400	-0.68138600	-0.65549900
H	3.05759600	-4.13663400	1.77697200
H	4.65119300	-2.63457400	0.60748800
Te	1.17469500	0.65765500	-1.30333100
C	0.61913300	1.70482900	0.46242100
C	1.40459000	1.59943700	1.60376600
C	-0.49311500	2.53636800	0.44185700
C	1.06329200	2.32491000	2.73664500
H	2.27847600	0.95602800	1.61686300
C	-0.82869700	3.25973100	1.57907000
H	-1.10255400	2.62329100	-0.45112400
C	-0.05336800	3.15156700	2.72534400
H	1.67085900	2.24182700	3.63141100
H	-1.69803000	3.90829600	1.56706200
H	-0.31872800	3.71436900	3.61378300
O	2.86609500	1.33809400	-1.53374500
H	-1.18404500	-2.36130900	0.91655000
H	-2.13641200	0.91969200	-1.77626700
H	-4.57606600	1.36226800	-1.92370100

Cartesian coordinates of the optimized geometry for **3b-IV** at PBE0-D3(SMD,MeOH)/def2-TZVP level of theory (number of imaginary frequencies = 0):

C	4.39987600	2.16364300	0.53838400
C	3.05059700	2.21195400	0.60831100
H	6.13748100	1.06866900	-0.16492100
C	5.05280500	1.07648600	-0.12636200
C	2.25612900	1.17418700	0.02097100
C	2.90738700	0.04032400	-0.66509300
C	4.35305800	0.07179200	-0.70054000
H	4.84774200	-0.75184100	-1.20355000
O	2.26499800	-0.88438500	-1.18073500
N	0.93865300	1.13671100	0.02299400
N	0.22776800	2.05744900	0.56856100
C	-1.16124700	2.02016000	0.52117400
C	-1.85595400	1.00828900	-0.14421800
C	-1.85170500	3.03652200	1.17911700
C	-3.24151200	1.03331900	-0.14217900
C	-3.23431100	3.04176700	1.17411100
H	-1.29539200	3.81449700	1.69245900
C	-3.93547000	2.03965700	0.51602700
H	-3.77843700	0.24726200	-0.66382400
H	-3.76578300	3.83444600	1.68829500
H	-5.01945500	2.04052500	0.51303900
Te	-0.92437000	-0.55170200	-1.25392100
C	-0.43018400	-1.73838000	0.44099100
C	-0.74611800	-1.37337600	1.74301400
C	0.19506200	-2.95384300	0.18300300
C	-0.43013500	-2.22884700	2.79082700
H	-1.23993400	-0.43127800	1.95328100
C	0.50305600	-3.80638900	1.23399800
H	0.44845700	-3.23993600	-0.83274800
C	0.19340200	-3.44302200	2.53798100
H	-0.67625900	-1.94359500	3.80812200
H	0.99083000	-4.75401800	1.03230400
H	0.43857300	-4.10794300	3.35910400
O	-2.46074600	-1.43325000	-1.75870100
H	2.57989100	3.04815300	1.11372100
H	4.99921300	2.94908600	0.98333300
H	0.65293700	2.85095400	1.04279100

Cartesian coordinates of the optimized geometry for **3b-V** at PBE0-D3(SMD,MeOH)/def2-TZVP level of theory (number of imaginary frequencies = 0):

C	3.84071200	-0.30158700	-1.53962100
C	2.48742300	-0.40135300	-1.38371700
H	5.75828700	-0.92248500	-0.75691900
C	4.68062300	-1.00093900	-0.64750800
C	1.90212700	-1.20025700	-0.37462000
C	2.74695800	-1.95219200	0.54773300
C	4.15964700	-1.77659100	0.34735300
H	4.80862100	-2.31458400	1.03150900
O	2.31493500	-2.68047000	1.47530400
N	0.53771100	-1.15421100	-0.28867900
N	-0.11032200	-1.98353000	0.40619600
C	-1.48351200	-1.75197700	0.42816800
C	-2.08722300	-0.64901800	-0.18540000
C	-2.28036800	-2.67503400	1.10267800
C	-3.46019400	-0.48385100	-0.15306600
C	-3.65322500	-2.50266800	1.14661700
H	-1.80221700	-3.52152700	1.58275100
C	-4.24556600	-1.41339900	0.51992700
H	-3.92377500	0.36328200	-0.64421500

H	-4.26767300	-3.22537900	1.67162200
H	-5.32081500	-1.28077400	0.55386500
Te	-0.82564900	0.66840300	-1.19546100
C	0.01408300	1.66558600	0.46008800
C	-0.52736500	1.52746900	1.73099800
C	1.11944300	2.47494700	0.22853400
C	0.05463400	2.21299800	2.78779200
H	-1.38870000	0.89213200	1.90545900
C	1.69205800	3.15486600	1.29456500
H	1.53741900	2.57563900	-0.76713600
C	1.16178900	3.02266400	2.57059400
H	-0.35894400	2.10932100	3.78488300
H	2.55635300	3.78714300	1.12441300
H	1.61437200	3.55262900	3.40145000
O	-2.24977200	2.02412400	-1.45188800
H	-2.61984100	2.32247200	-0.61026100
H	1.83437700	0.12093800	-2.07453100
H	4.26097000	0.30121300	-2.33556300

Cartesian coordinates of the optimized geometry for **3b-VI** at PBE0-D3(SMD,MeOH)/def2-TZVP level of theory (number of imaginary frequencies = 0):

C	4.09686200	-2.33647700	0.72950300
C	2.73181200	-2.30257400	0.77684200
H	5.87712400	-1.37043100	-0.02526400
C	4.79246800	-1.33778500	0.01057100
C	2.02030100	-1.27757700	0.11942000
C	2.70302000	-0.23895700	-0.60758500
C	4.12893600	-0.33431300	-0.63735000
H	4.66672400	0.43215500	-1.18556600
O	2.08104100	0.70484600	-1.18339500
N	0.65569900	-1.21516200	0.11873800
N	-0.03048000	-2.09058300	0.71163600
C	-1.40400100	-1.90466700	0.58184800
C	-1.94485300	-0.86245600	-0.17749700
C	-2.24859100	-2.79693700	1.24106900
C	-3.31647900	-0.72190400	-0.28744700
C	-3.61974500	-2.64186600	1.14039500
H	-1.81168200	-3.59982100	1.82469800
C	-4.15525100	-1.60849200	0.37945300
H	-3.73941900	0.07286200	-0.89081800
H	-4.27816100	-3.33266000	1.65499000
H	-5.23020800	-1.49134300	0.30032100
Te	-0.55809100	0.35450500	-1.19130500
C	-0.20115200	1.71451200	0.38622400
C	-0.39095200	1.36025200	1.71314000
C	0.21348400	2.99472100	0.03917700
C	-0.15966000	2.30319400	2.70679800
H	-0.72199600	0.36620800	1.99076500
C	0.43808600	3.92976500	1.03903300
H	0.36326000	3.26712100	-0.99982500
C	0.25401900	3.58438700	2.37163600
H	-0.30805600	2.03069200	3.74597600
H	0.76266900	4.92991300	0.77352900
H	0.43288600	4.31707100	3.15093900
O	-1.99959200	1.55874500	-1.90524100
H	-2.46899600	2.01973100	-1.19805500
H	2.17352600	-3.05863700	1.31593900
H	4.64511600	-3.12257300	1.23529100

6. Crystal Structure Data

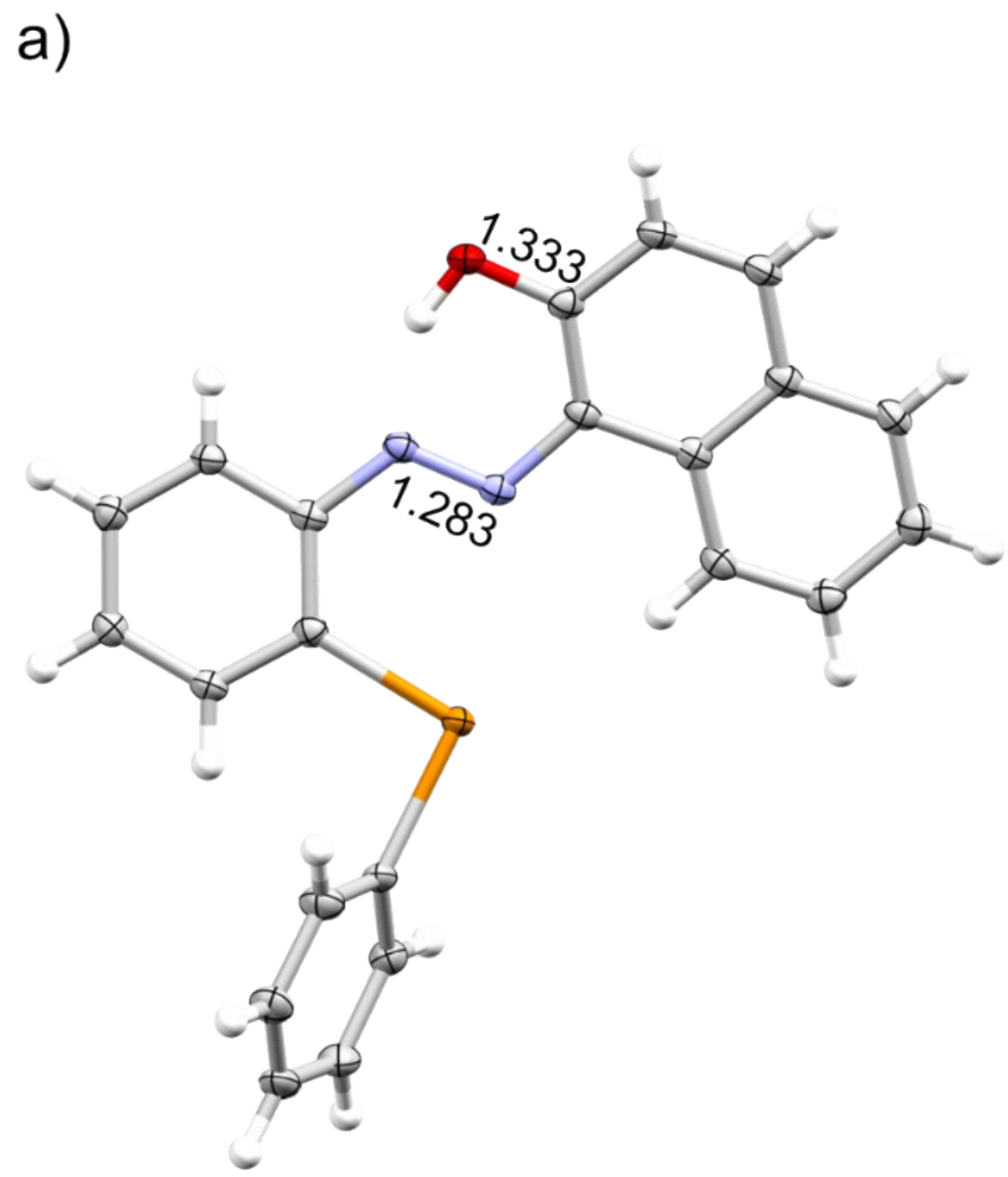

Figure S54. Solid state structures of *o*-**2a** (a) and **1-S** (b). The bond distances are given in angstroms.

Crystal Structure Data of *o*-2a

Table S5. Crystal structure data and structure refinement for *o*-**2a.**

Deposition number	2504631
Empirical formula	$C_{22}H_{16}N_2OTe$
Formula weight	451.97
Density (calculated)	1.730 g·cm^{-3}
$F(000)$	888
Temperature	100(2) K
Crystal size	0.222 × 0.079 × 0.062 mm
Crystal color	red
Crystal description	prism
Wavelength	1.54178 Å
Crystal system	monoclinic
Space group	$P2_1/c$
Unit cell dimensions	
a [Å]	17.7414(8)
b [Å]	4.6023(2)
c [Å]	21.6096(10)
α [°]	90
β [°]	100.3548(18)
γ [°]	90
Volume	1735.71(14) Å^3
Z	4
Cell measurement reflections used	9961
Cell measurement θ min/max	2.53°/79.26°
Diffractometer control software	Bruker APEX3(v2017.3-0)
Diffractometer measurement device	Bruker D8 Venture (Photon II detector)
Diffractometer measurement method	Data collection strategy APEX 3/Queen
θ range for data collection	2.532°- 80.480°
Completeness to θ = 67.679°	99.8%
Completeness to θ_{max} = 80.480°	99.4%
Index ranges	$-22 \leq h \leq 22$
	$-5 \leq k \leq 4$
	$-27 \leq l \leq 27$
Computing data reduction	Bruker APEX3(v2017.3-0)
Absorption coefficient	13.629 mm^{-1}
Absorption correction	Semi-empirical from equivalents
Computation absorption correction	SADABS
Max./min. Transmission	0.75/0.39
R_{merg} before/after correction	0.1435/0.0703
Computing structure solution	Bruker APEX3(v2017.3-0)
Computing structure refinement	SHELXL-2017/1 (Sheldrick, 2017)
Refinement method	Full-matrix least-squares on F^2
Reflections collected	53531
Independent reflections	3765
R_{int}	0.0694

Reflections with $I > 2\sigma(I)$	3356
Restraints	0
Parameter	239
GooF	1.105
Weighting details	$w = 1/[\sigma^2(F_{obs}^2) + (0.0288P)^2 + 1.9574P]$ where $P = (F_{obs}^2 + 2F_{calc}^2)/3$
R_1 [$I > 2\sigma(I)$]	0.0238
wR_2 [$I > 2\sigma(I)$]	0.0572
R_1 [all data]	0.0294
wR_2 [all data]	0.0607
Largest diff. peak and hole	0.999/-0.691

Comments

Treatment of hydrogen atoms

Riding model on idealized geometries with the 1.2-fold isotropic displacement parameters of the equivalent U_{ij} of the corresponding carbon atom. The OH hydrogen atom was refined freely.

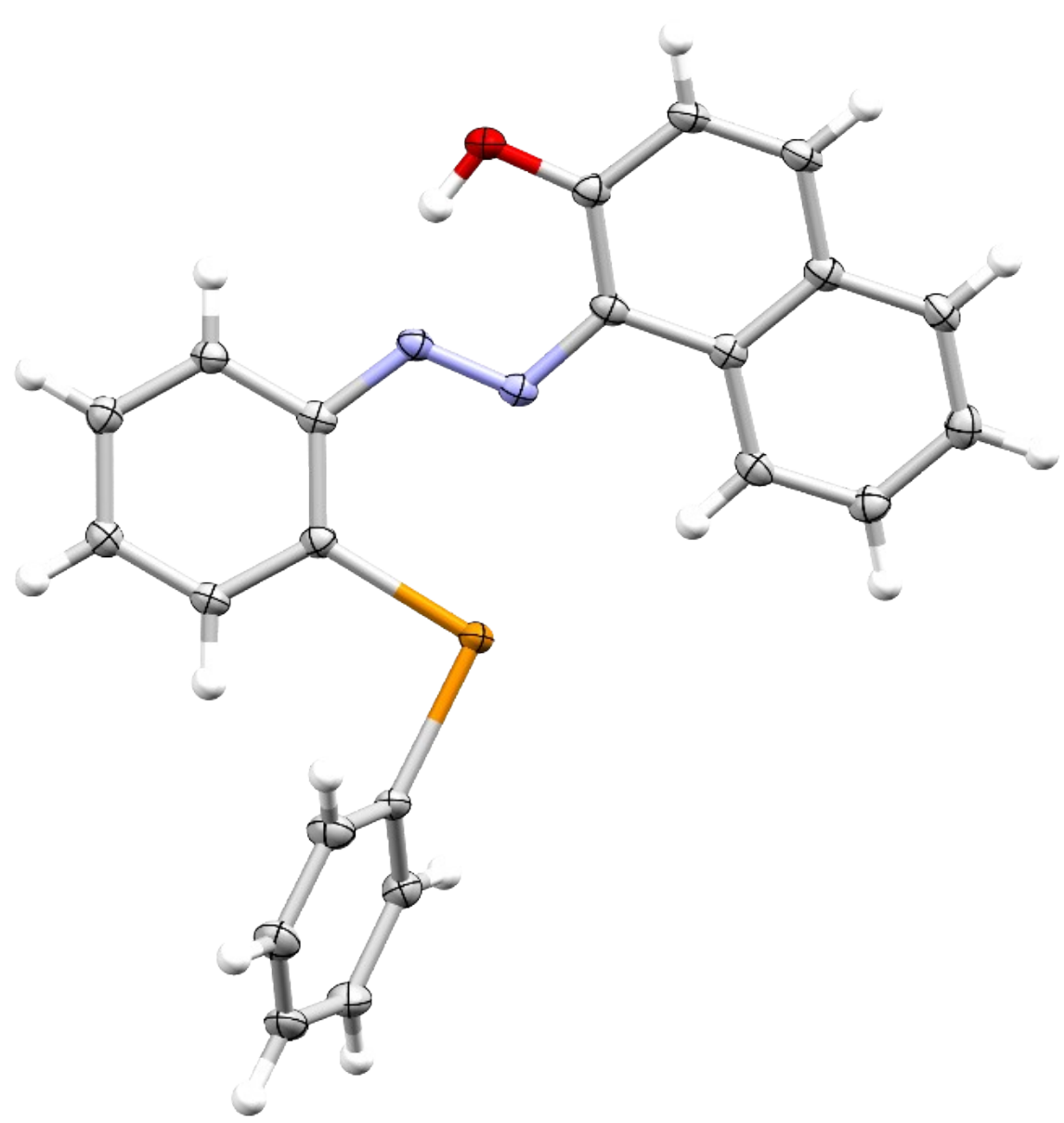

Figure S55. Molecular structure of *o*-**2a** with thermal ellipsoids at 50% probability level.

Crystal Structure Data of 1-S

Table S6. Crystal structure data and structure refinement for **1-S**.

Deposition number	2504630
Empirical formula	$C_{22}H_{16}N_2OS$
Formula weight	356.43
Density (calculated)	1.394 g·cm^{-3}
F(000)	372
Temperature	100(2) K
Crystal size	0.238 × 0.156 × 0.084 mm
Crystal color	orange
Crystal description	tablet
Wavelength	0.71073 Å
Crystal system	monoclinic
Space group	$P2_1$
Unit cell dimensions	
a [Å]	5.5363(8)
b [Å]	18.159(3)
c [Å]	8.4460(12)
α [°]	90
β [°]	91.035(3)
γ [°]	90
Volume	849.0(2) Å^3
Z	2
Cell measurement reflections used	9986
Cell measurement *θ* min/max	2.24°/33.29°
Diffractometer control software	BRUKER APEX3(v2019.1-0)
Diffractometer measurement device	Bruker D8 KAPPA II (APEX II detector)
Diffractometer measurement method	Data collection strategy APEX 3/Queen
θ range for data collection	2.243°- 33.328°
Completeness to *θ* = 25.242°	100.0%
Completeness to θ_{max} = 33.328°	99.9%
Index ranges	-8 ≤ *h* ≤ 8<br>-28 ≤ *k* ≤ 28<br>-13 ≤ *l* ≤ 13
Computing data reduction	BRUKER APEX3(v2019.1-0)
Absorption coefficient	0.204 mm^{-1}
Absorption correction	Semi-empirical from equivalents
Computation absorption correction	SADABS
Max./min. Transmission	0.75/0.72
R_{merg} before/after correction	0.0478/0.0443
Computing structure solution	BRUKER APEX3(v2019.1-0)
Computing structure refinement	SHELXL-2017/1 (Sheldrick, 2017)
Refinement method	Full-matrix least-squares on F^2
Reflections collected	31385
Independent reflections	6551
R_{int}	0.0264

Reflections with $I > 2\sigma(I)$	6242
Restraints	1
Parameter	239
GooF	1.065
Weighting details	$w = 1/[\sigma^2(F_{obs}^2) + (0.0616P)^2 + 0.0217P]$ where $P = (F_{obs}^2 + 2F_{calc}^2)/3$
R_1 [$I > 2\sigma(I)$]	0.0326
wR_2 [$I > 2\sigma(I)$]	0.0854
R_1 [all data]	0.0350
wR_2 [all data]	0.0872
Absolute structure parameter	0.008(13)
Largest diff. peak and hole	0.461/-0.201

Comments

Treatment of hydrogen atoms

Riding model on idealized geometries with the 1.2-fold isotropic displacement parameters of the equivalent U_{ij} of the corresponding carbon atom. The NH hydrogen atom was refined freely.

Absolute structure

The absolute structure could be determined reliably. Parsons' quotient method was used to determine the absolute structure parameter x. For more details, see references [13] and [14].

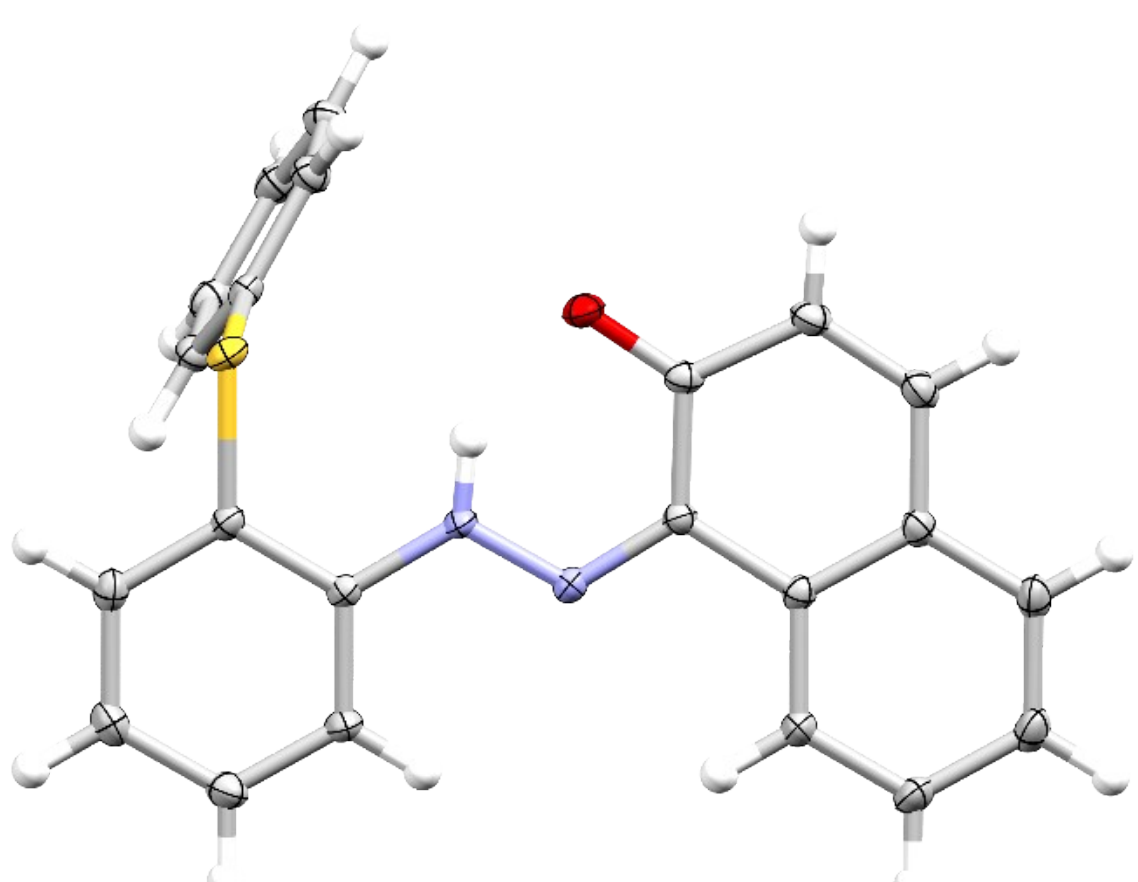

Figure S56. Molecular structure of **1-S** with thermal ellipsoids at 50% probability level.

7. ^{1}H NMR, ^{13}C NMR ^{77}Se NMR and ^{125}Te NMR Spectra of the New Compounds

The field strengths and solvents of the following spectra are given in the Experimental Section.

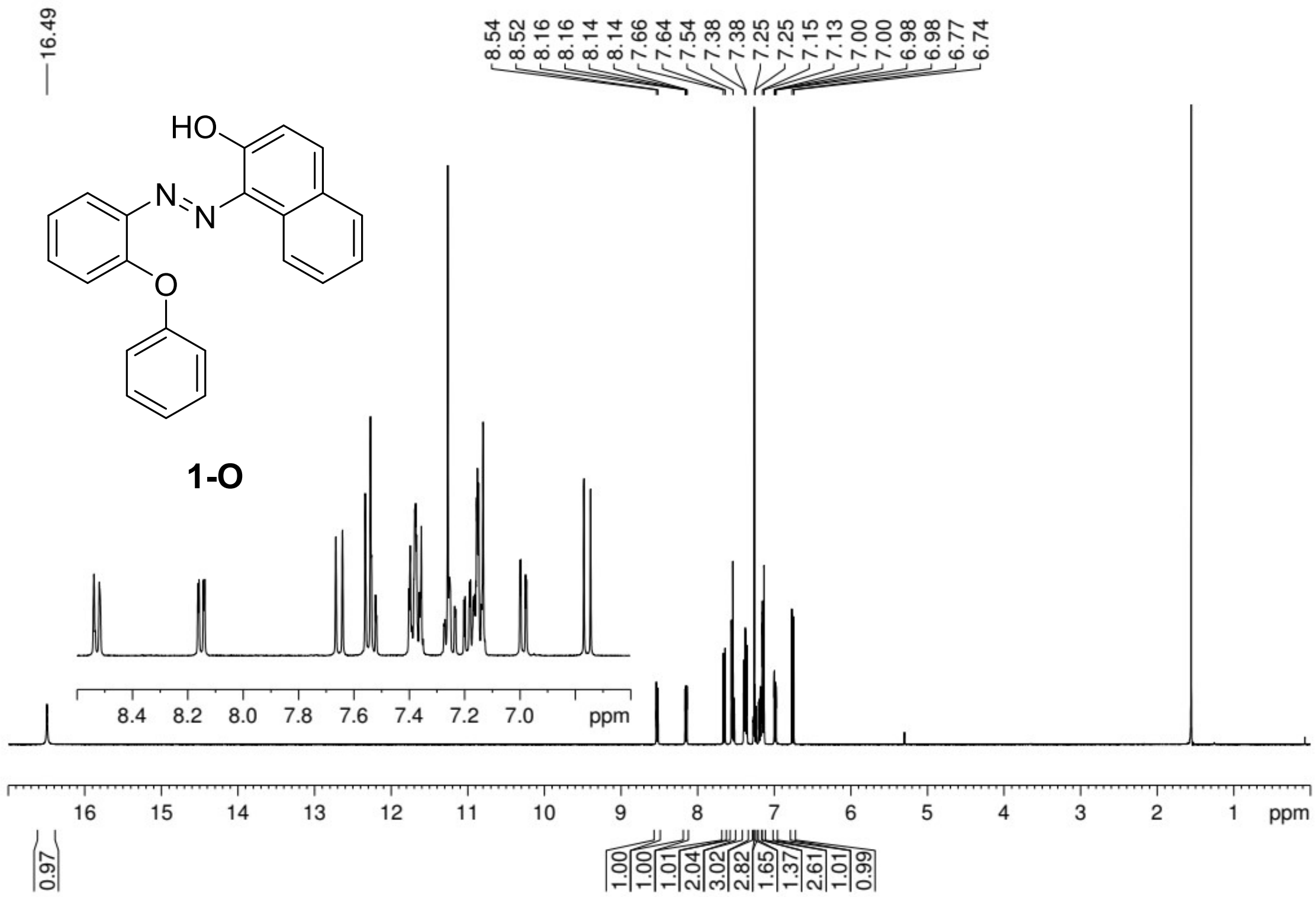

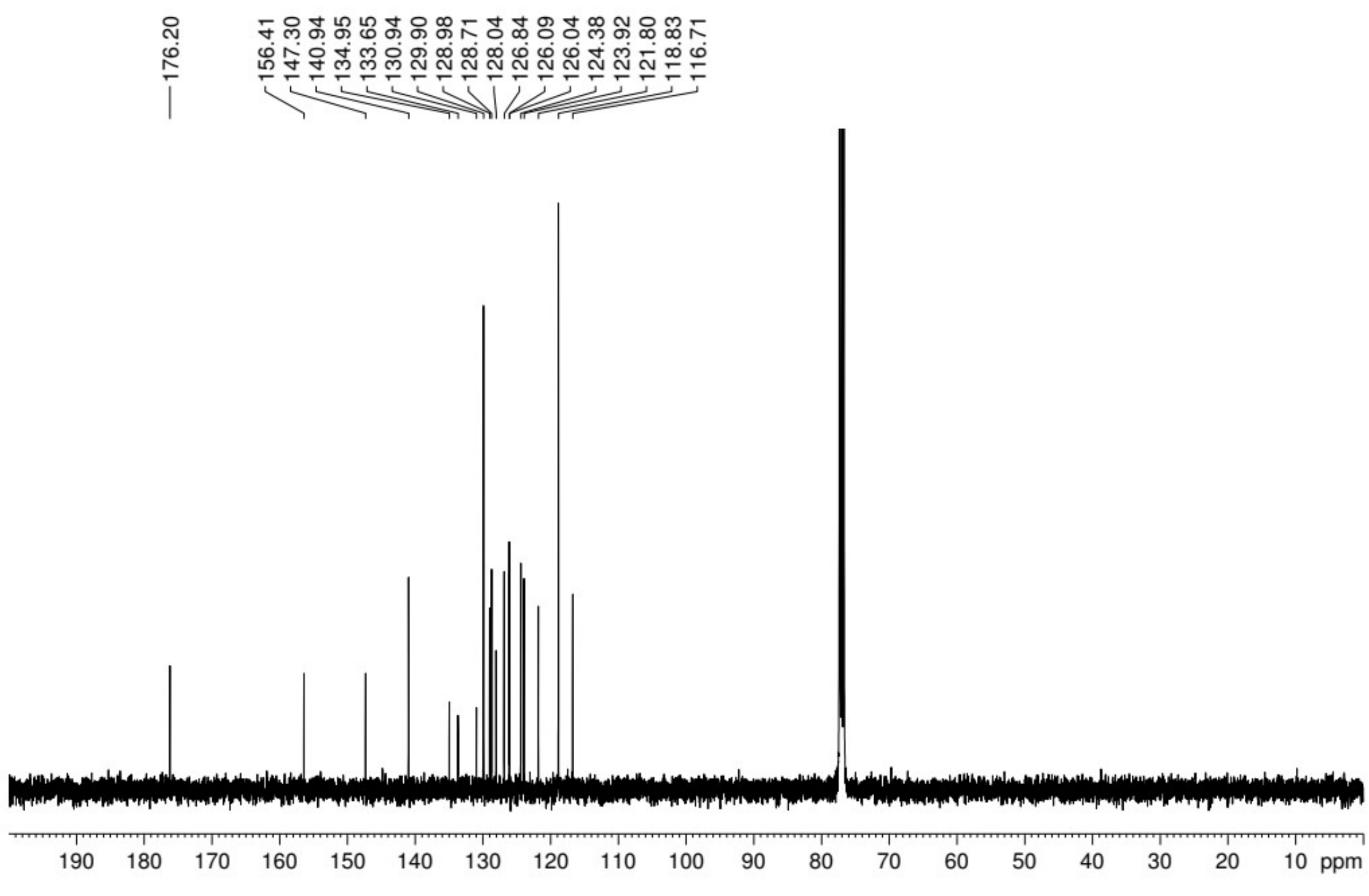

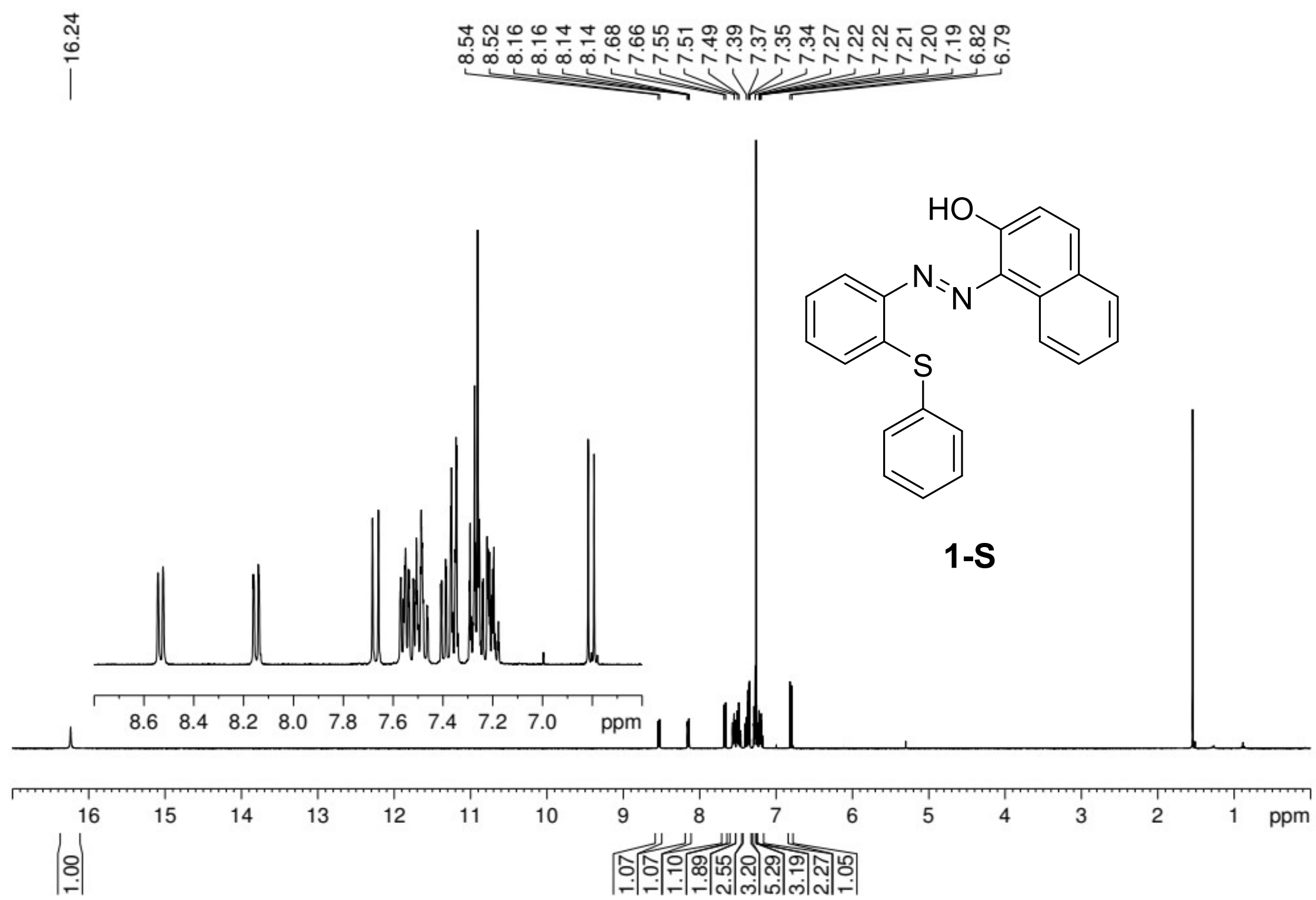
16.24
8.54 8.52 8.16 8.16 8.14 8.14 7.68 7.66 7.55 7.51 7.49 7.39 7.37 7.35 7.34 7.27 7.22 7.22 7.21 7.20 7.19 6.82 6.79
HO
N
N
S
1-S
8.6 8.4 8.2 8.0 7.8 7.6 7.4 7.2 7.0 ppm
16 15 14 13 12 11 10 9 8 7 6 5 4 3 2 1 ppm
1.00
1.07 1.07 1.10 1.89 2.55 3.20 5.29 3.19 2.27 1.05

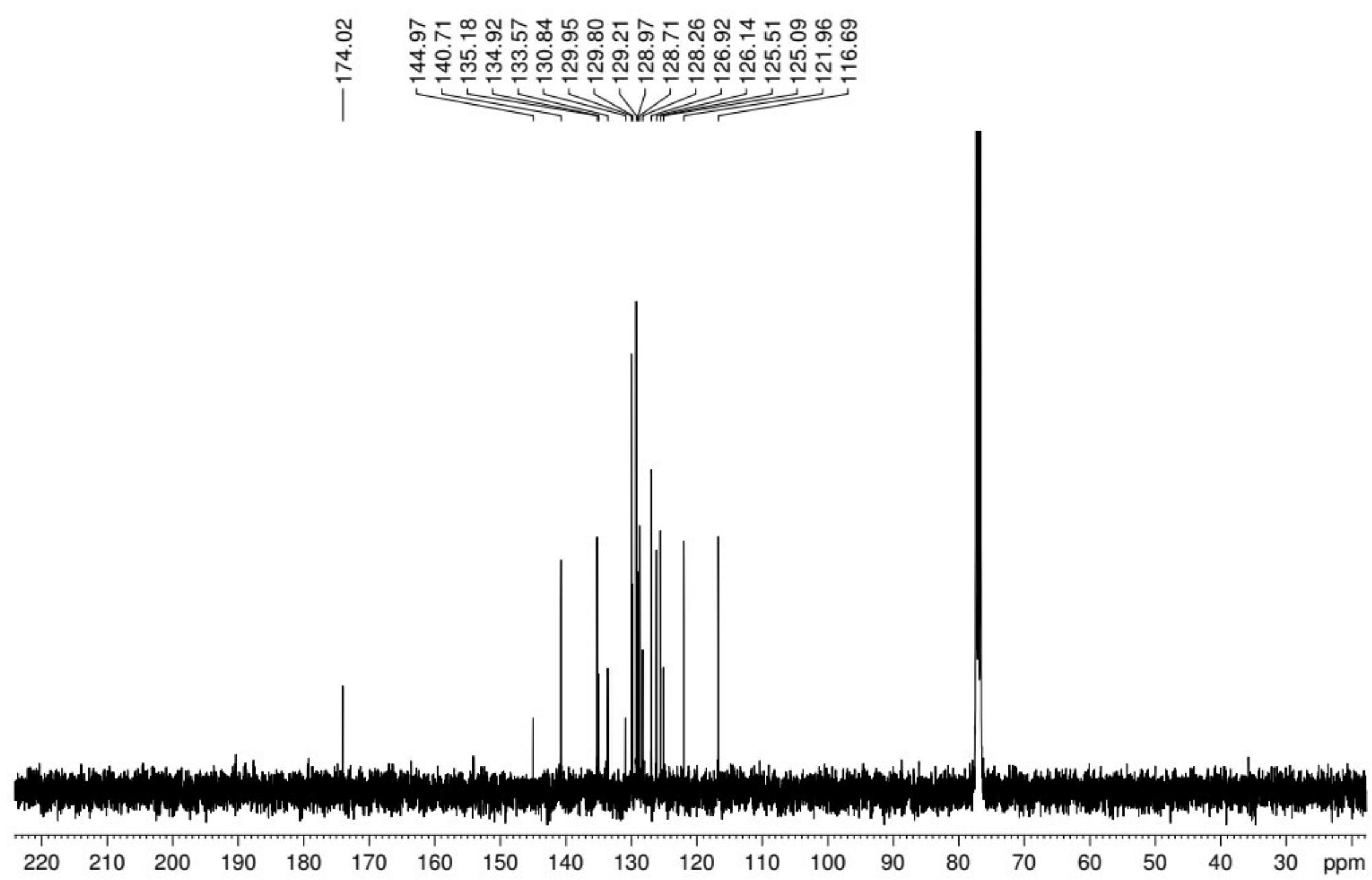
174.02
144.97 140.71 135.18 134.92 133.57 130.84 129.95 129.80 129.21 128.97 128.71 128.26 126.92 126.14 125.51 125.09 121.96 116.69
220 210 200 190 180 170 160 150 140 130 120 110 100 90 80 70 60 50 40 30 ppm

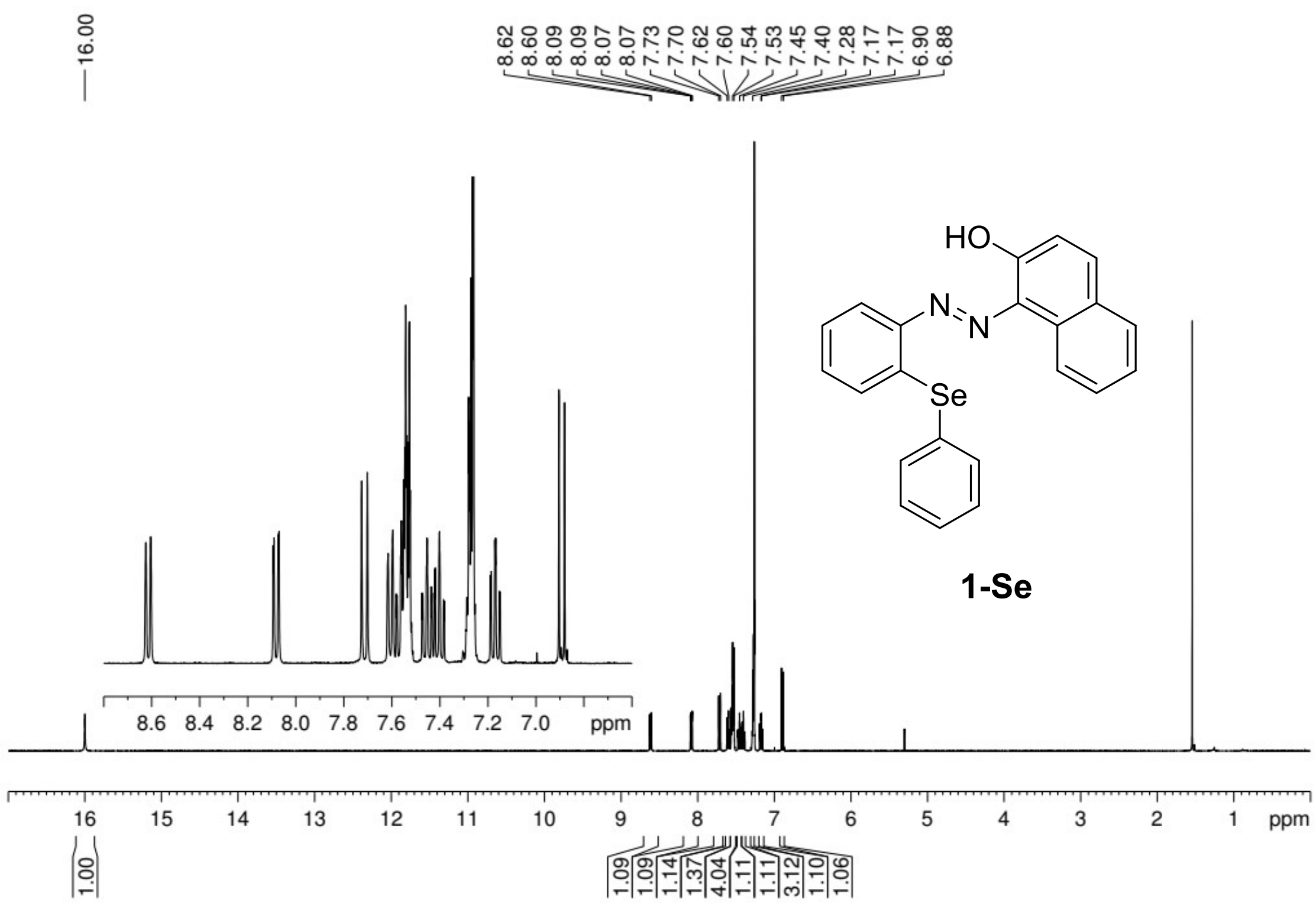
16.00
8.62
8.60
8.09
8.09
8.07
8.07
7.73
7.70
7.62
7.60
7.54
7.53
7.45
7.40
7.28
7.17
7.17
6.90
6.88
HO
N
N
Se
1-Se
8.6 8.4 8.2 8.0 7.8 7.6 7.4 7.2 7.0 ppm
16 15 14 13 12 11 10 9 8 7 6 5 4 3 2 1 ppm
1.00
1.09
1.09
1.14
1.37
4.04
1.11
1.11
3.12
1.10
1.06

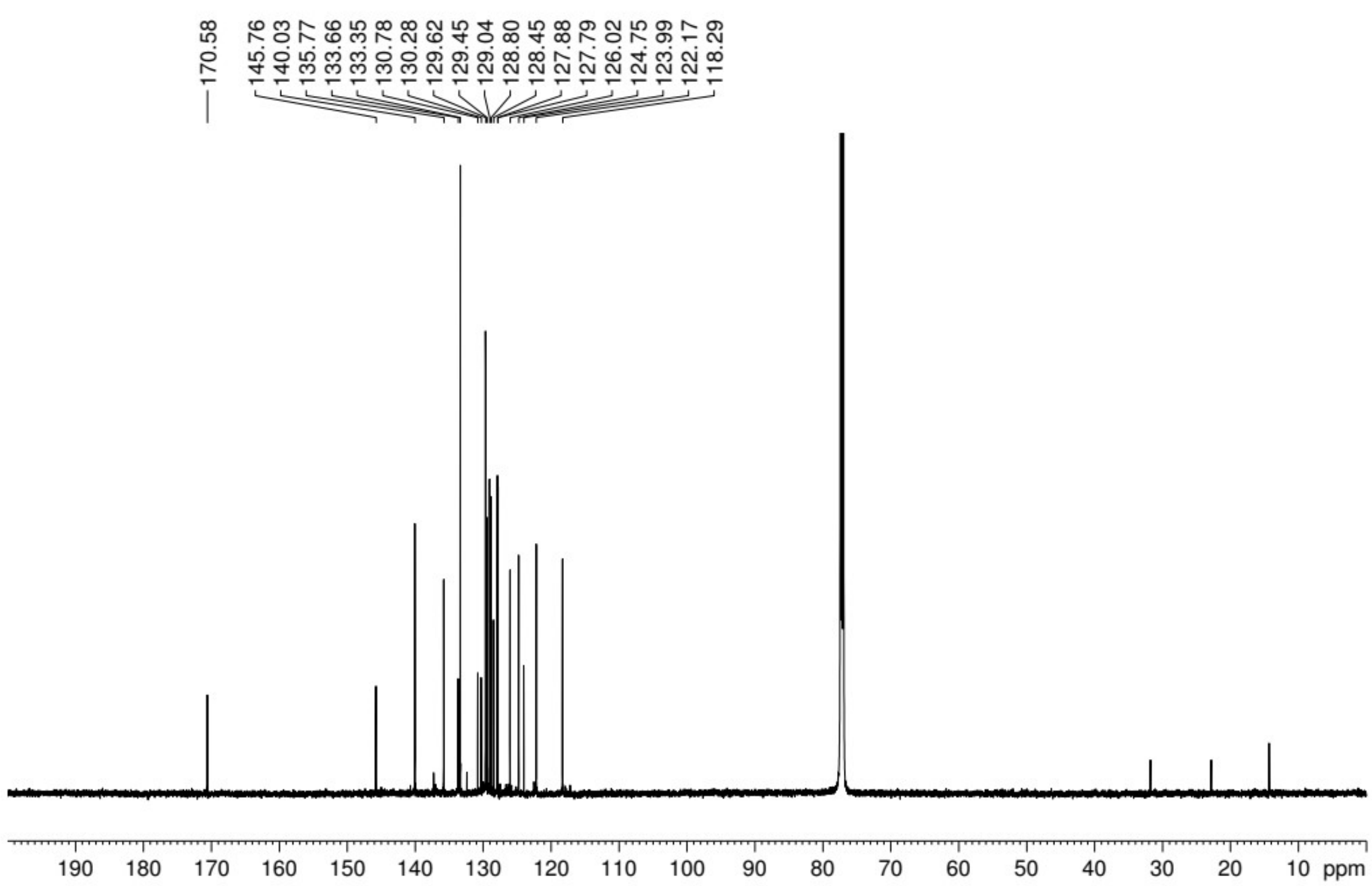
170.58
145.76
140.03
135.77
133.66
133.35
130.78
130.28
129.62
129.45
129.04
128.80
128.45
127.88
127.79
126.02
124.75
123.99
122.17
118.29
190 180 170 160 150 140 130 120 110 100 90 80 70 60 50 40 30 20 10 ppm

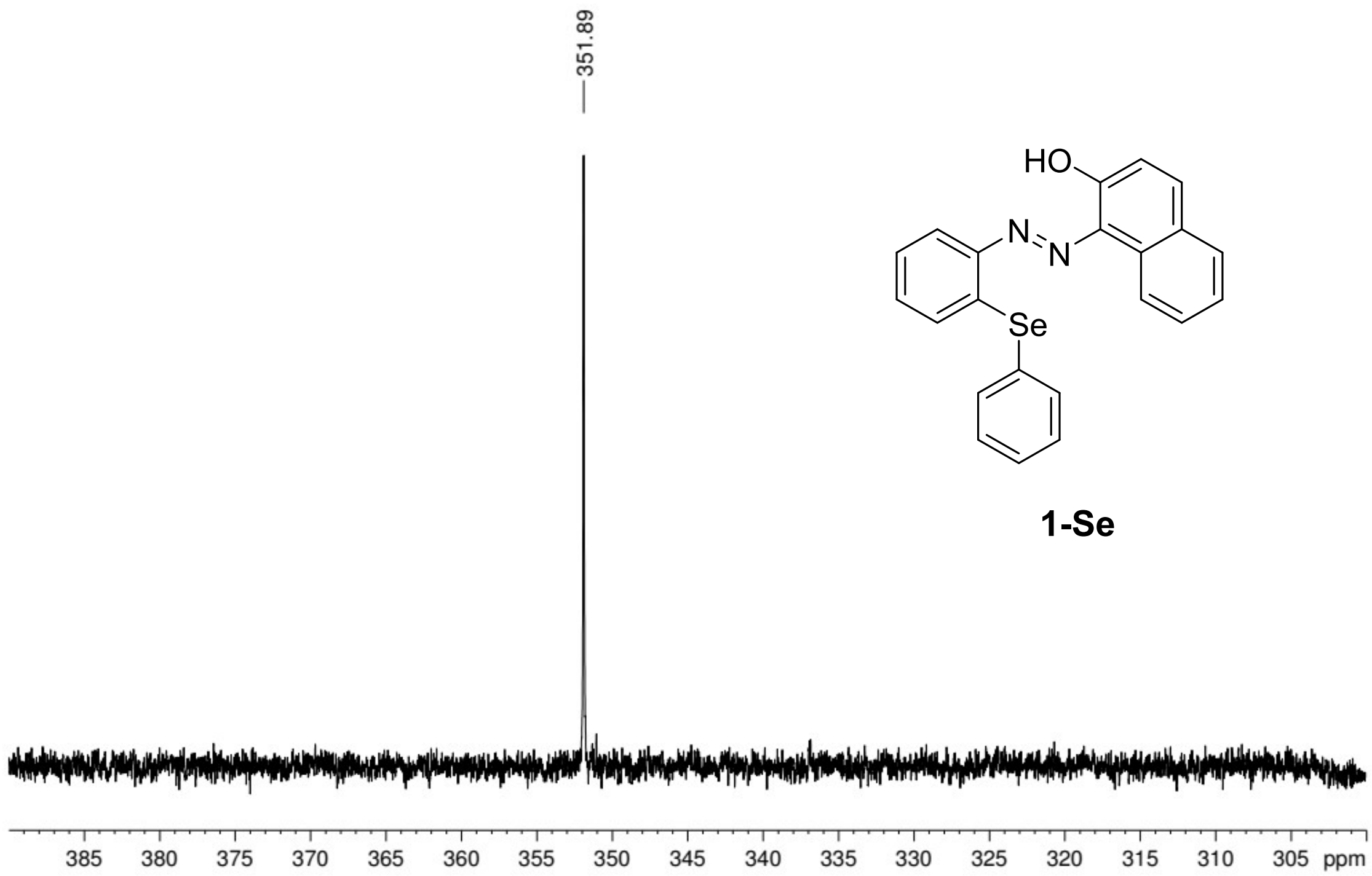
351.89
HO
N
N
Se
385
380
375
370
365
360
355
350
345
340
335
330
325
320
315
310
305
ppm

1-Se

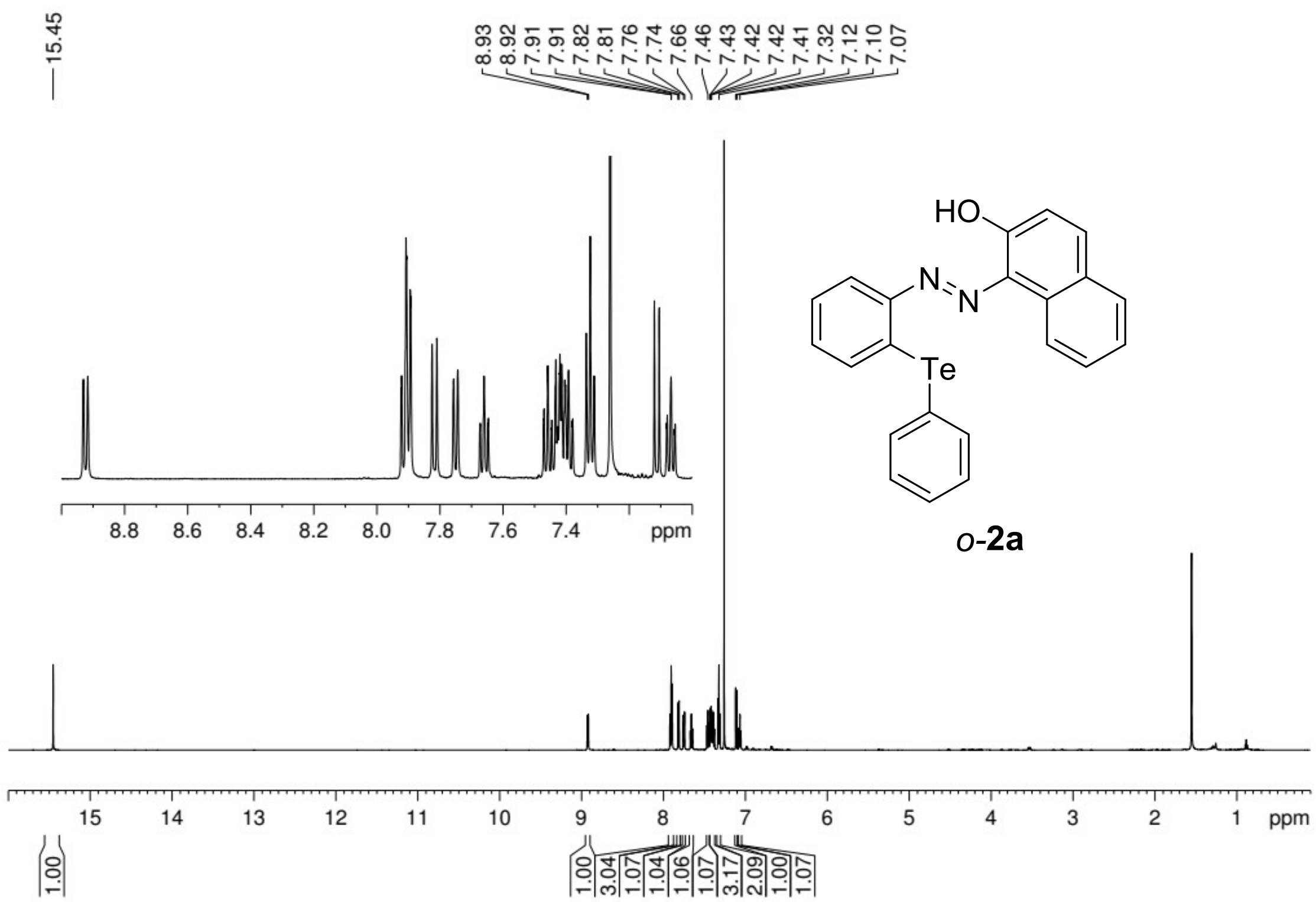
15.45
8.93 8.92 7.91 7.91 7.82 7.81 7.76 7.74 7.66 7.46 7.43 7.42 7.42 7.41 7.32 7.12 7.10 7.07
8.8 8.6 8.4 8.2 8.0 7.8 7.6 7.4 ppm
15 14 13 12 11 10 9 8 7 6 5 4 3 2 1 ppm
1.00
1.00 3.04 1.07 1.04 1.06 1.07 3.17 2.09 1.00 1.07
HO
N
N
Te

o-**2a**

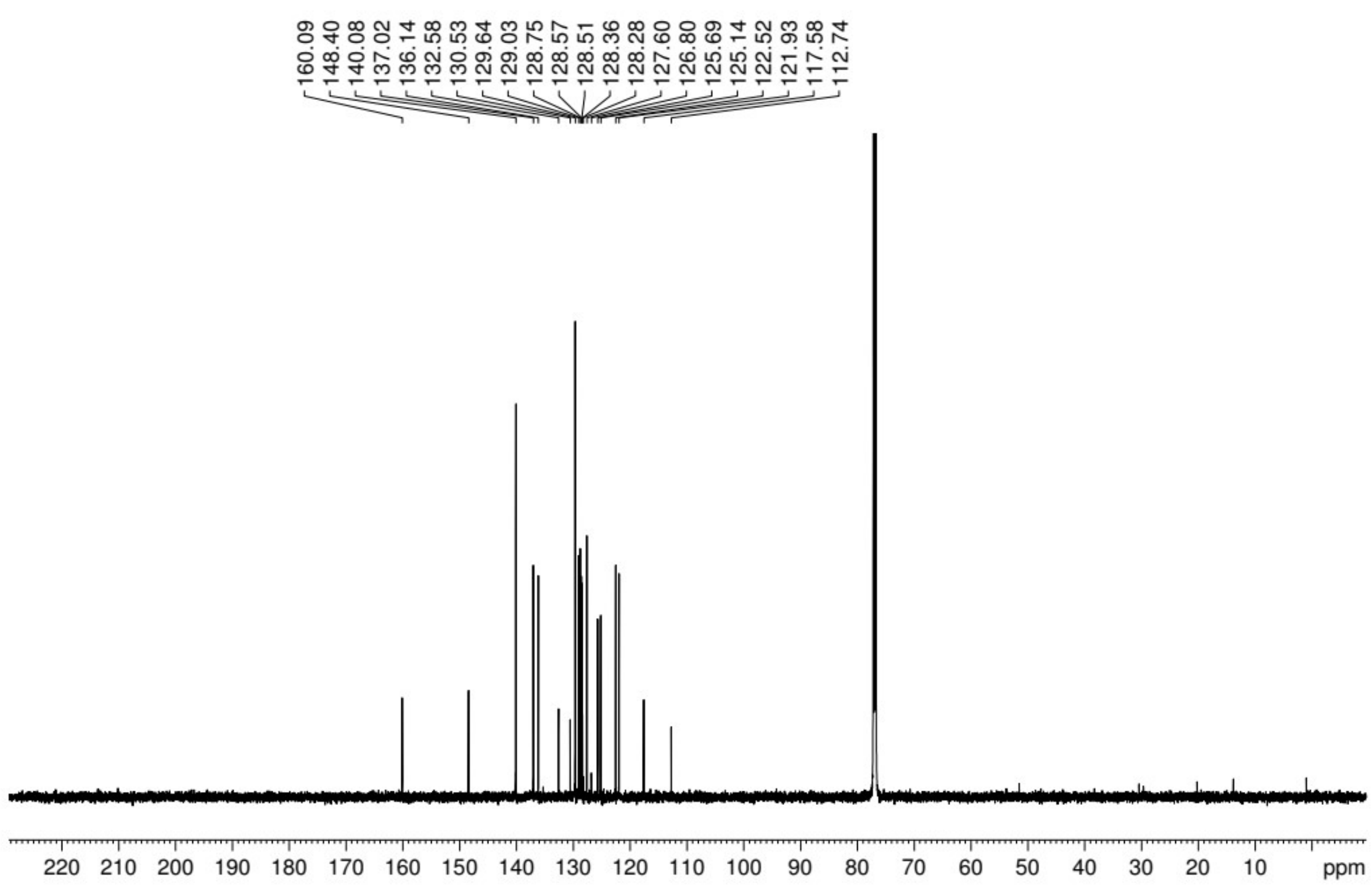
160.09 148.40 140.08 137.02 136.14 132.58 130.53 129.64 129.03 128.75 128.57 128.51 128.36 128.28 127.60 126.80 125.69 125.14 122.52 121.93 117.58 112.74
220 210 200 190 180 170 160 150 140 130 120 110 100 90 80 70 60 50 40 30 20 10 ppm

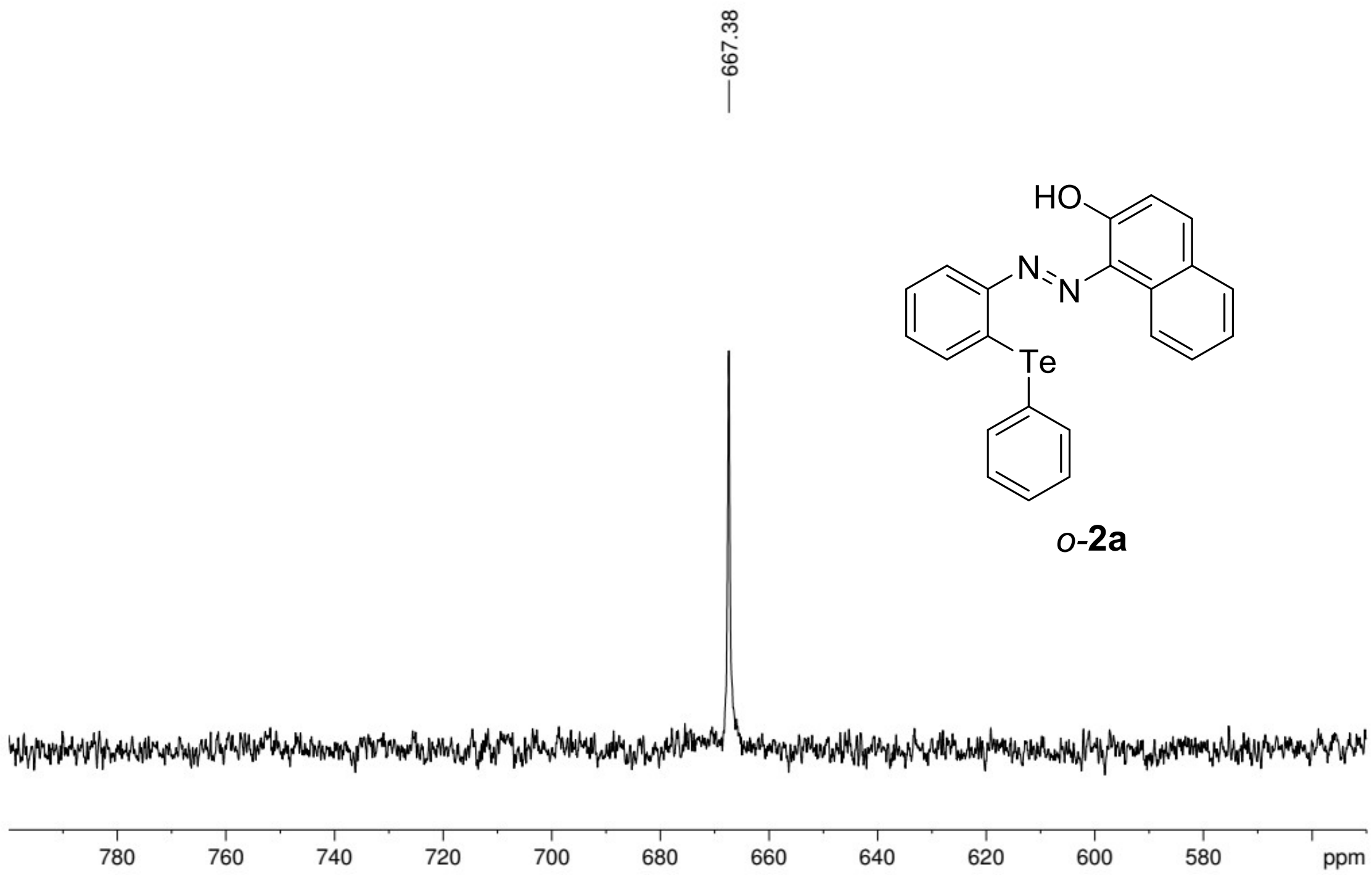

o-**2a**

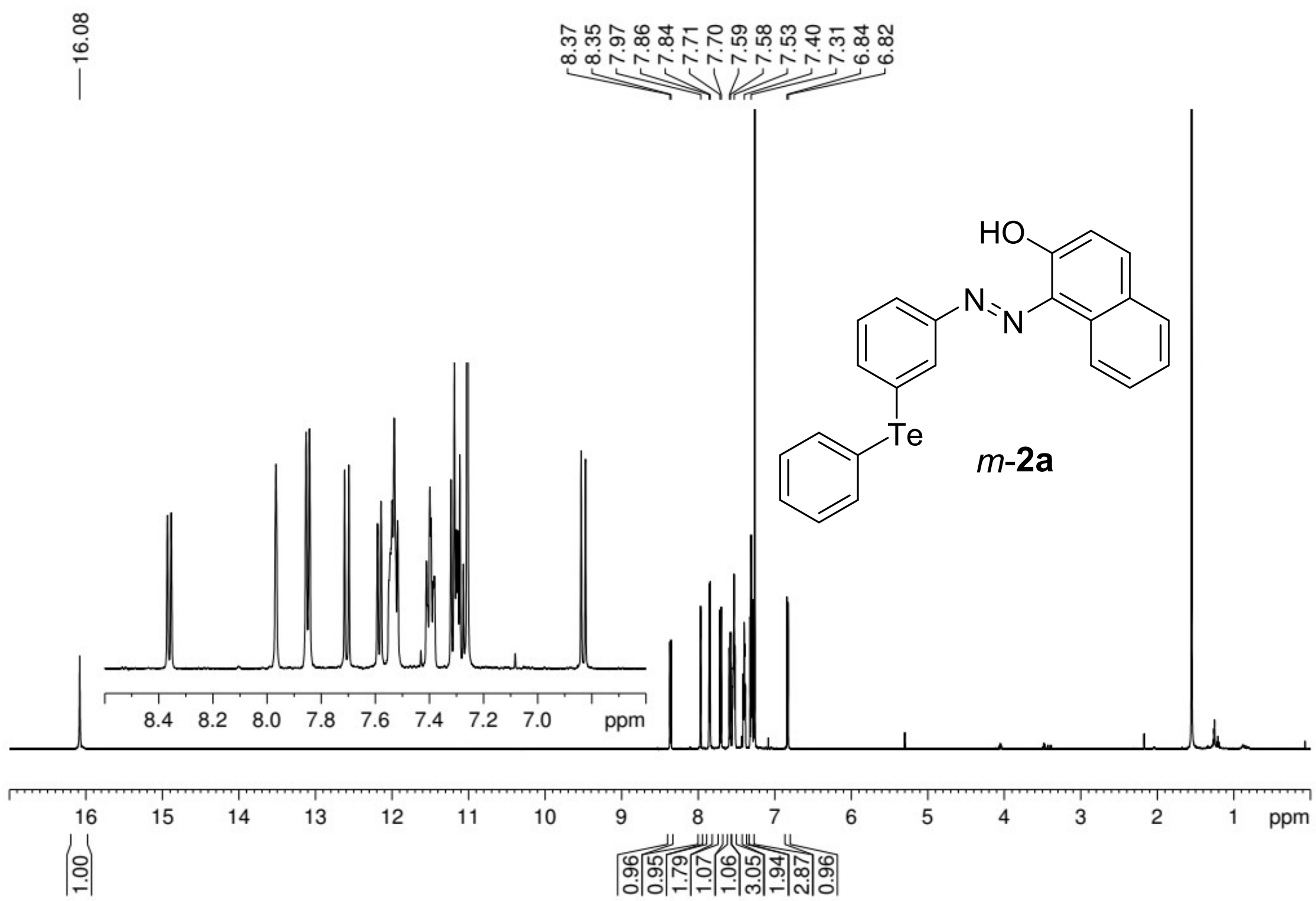

16.08
8.37
8.35
7.97
7.86
7.84
7.71
7.70
7.59
7.58
7.53
7.40
7.31
6.84
6.82
HO
N
N
Te
m-2a
8.4 8.2 8.0 7.8 7.6 7.4 7.2 7.0 ppm
16 15 14 13 12 11 10 9 8 7 6 5 4 3 2 1 ppm
1.00
0.96
0.95
1.79
1.07
1.06
3.05
1.94
2.87
0.96

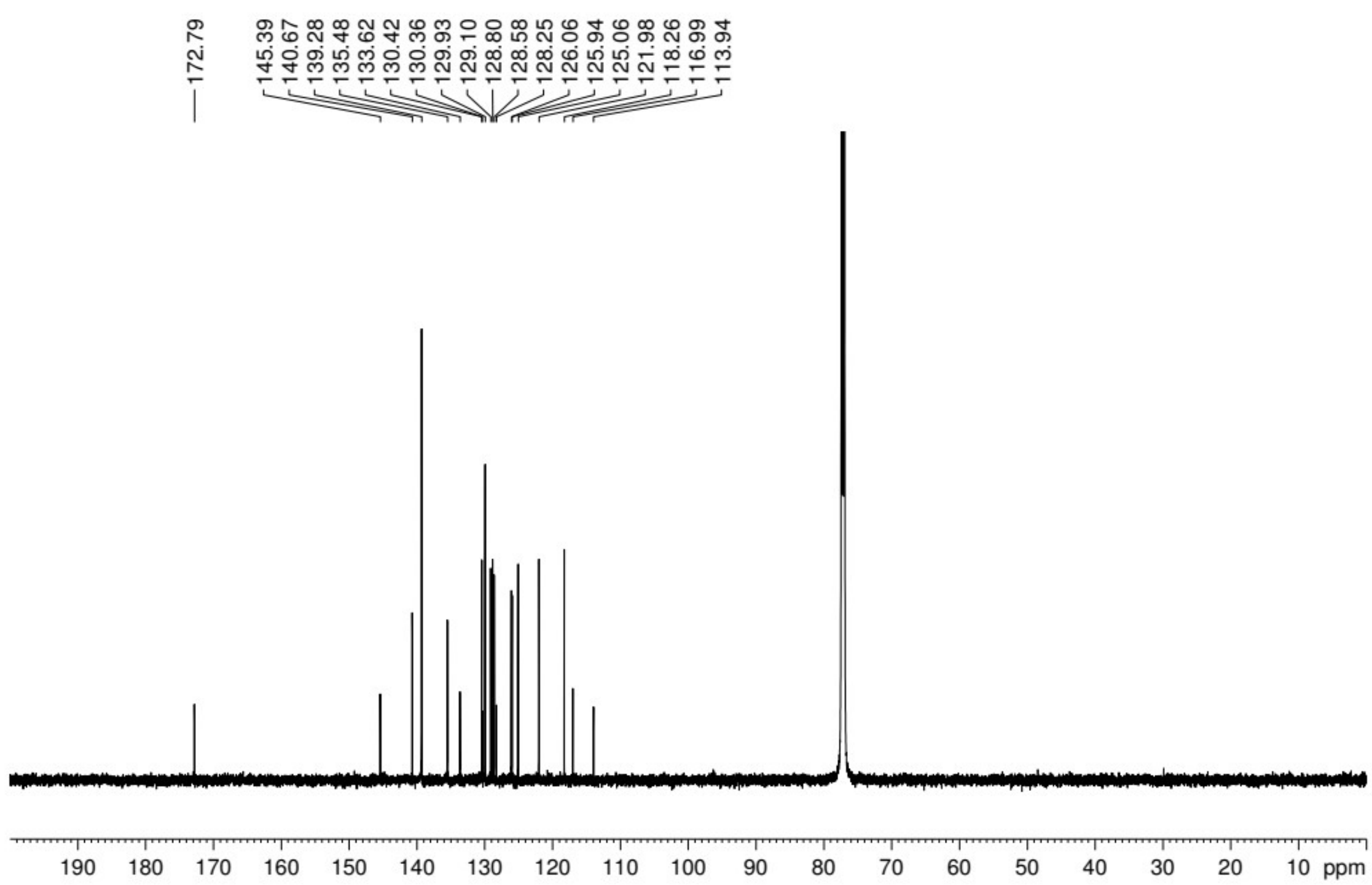

172.79
145.39
140.67
139.28
135.48
133.62
130.42
130.36
129.93
129.10
128.80
128.58
128.25
126.06
125.94
125.06
121.98
118.26
116.99
113.94
190 180 170 160 150 140 130 120 110 100 90 80 70 60 50 40 30 20 10 ppm

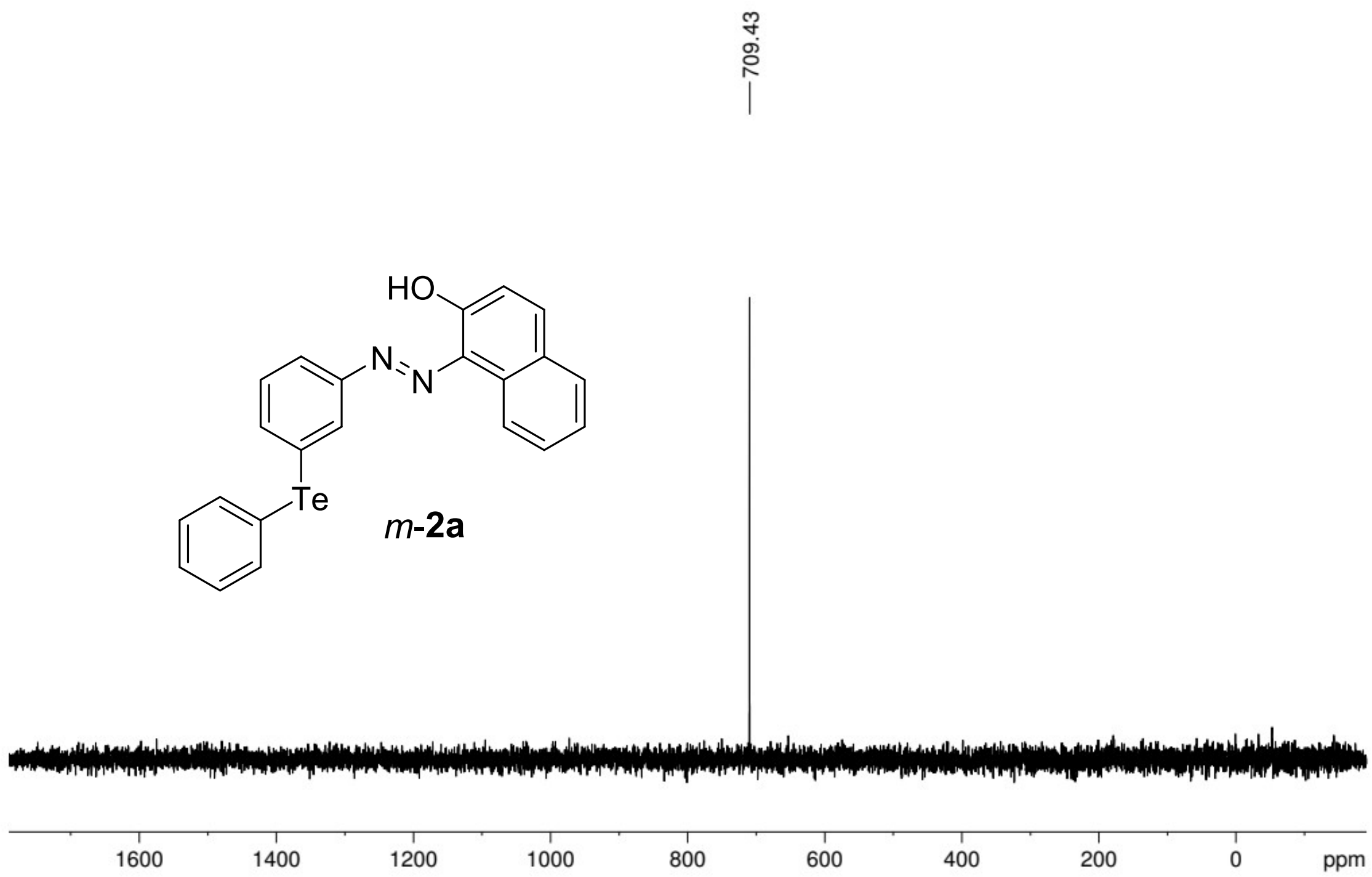
709.43
HO
N
N
Te
m-2a
1600
1400
1200
1000
800
600
400
200
0
ppm

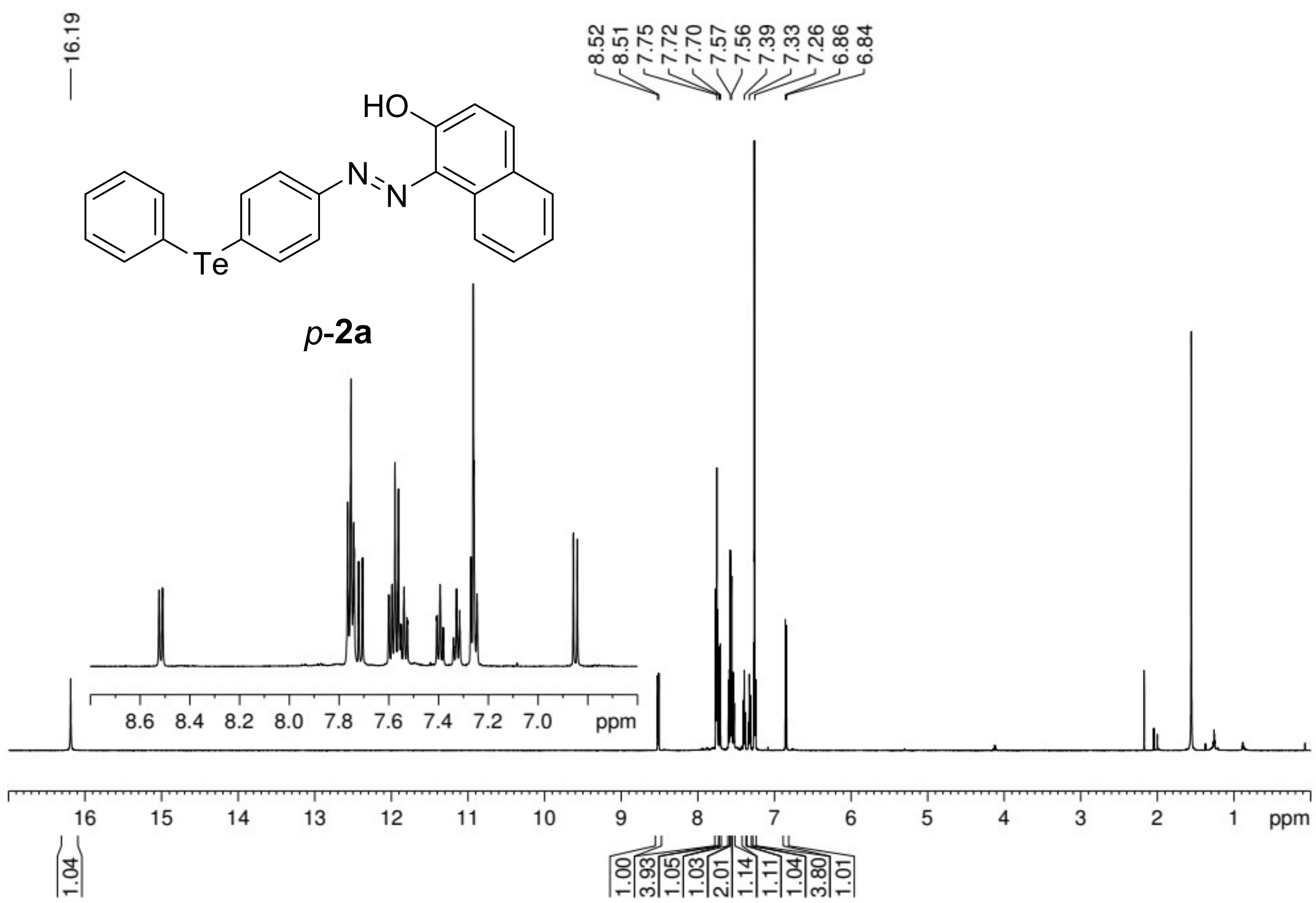
16.19
8.52
8.51
7.75
7.72
7.70
7.57
7.56
7.39
7.33
7.26
6.86
6.84
HO
N
N
Te
8.6 8.4 8.2 8.0 7.8 7.6 7.4 7.2 7.0 ppm
16 15 14 13 12 11 10 9 8 7 6 5 4 3 2 1 ppm
1.04
1.00
3.93
1.05
1.03
2.01
1.14
1.11
1.04
3.80
1.01

p-**2a**

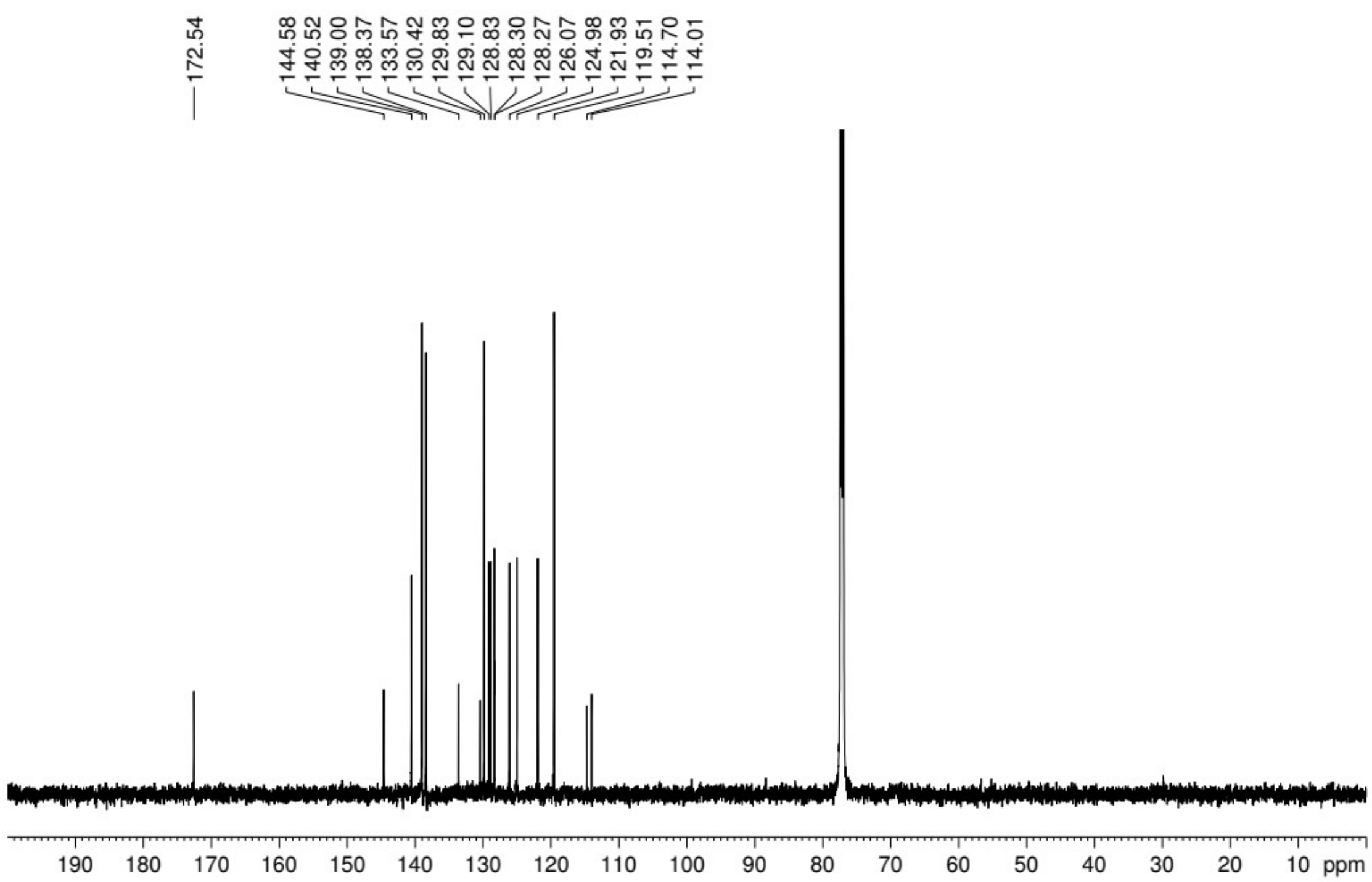
172.54
144.58
140.52
139.00
138.37
133.57
130.42
129.83
129.10
128.83
128.30
128.27
126.07
124.98
121.93
119.51
114.70
114.01
190 180 170 160 150 140 130 120 110 100 90 80 70 60 50 40 30 20 10 ppm

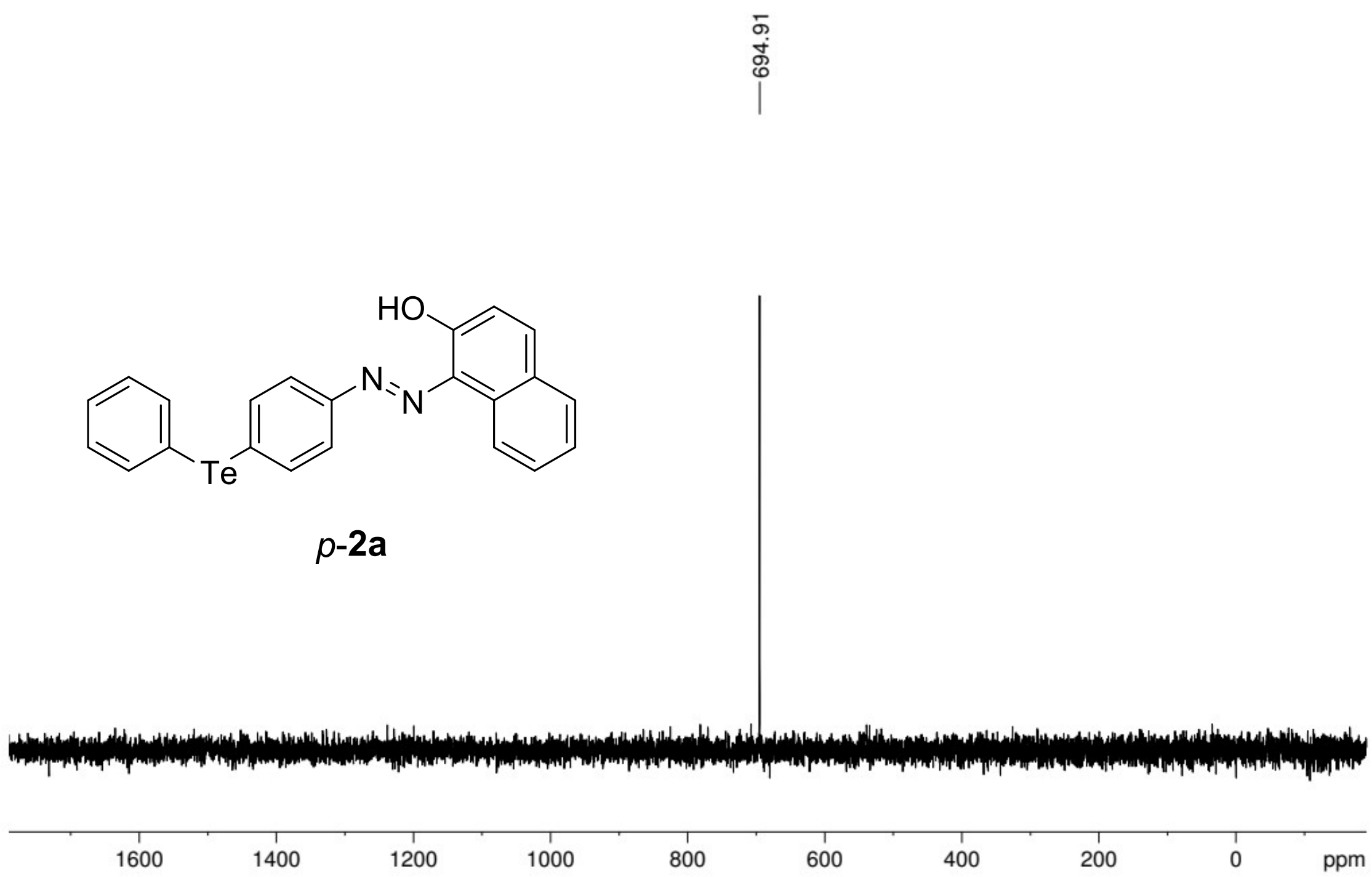
694.91
HO
N
N
Te
p-2a
1600
1400
1200
1000
800
600
400
200
0
ppm

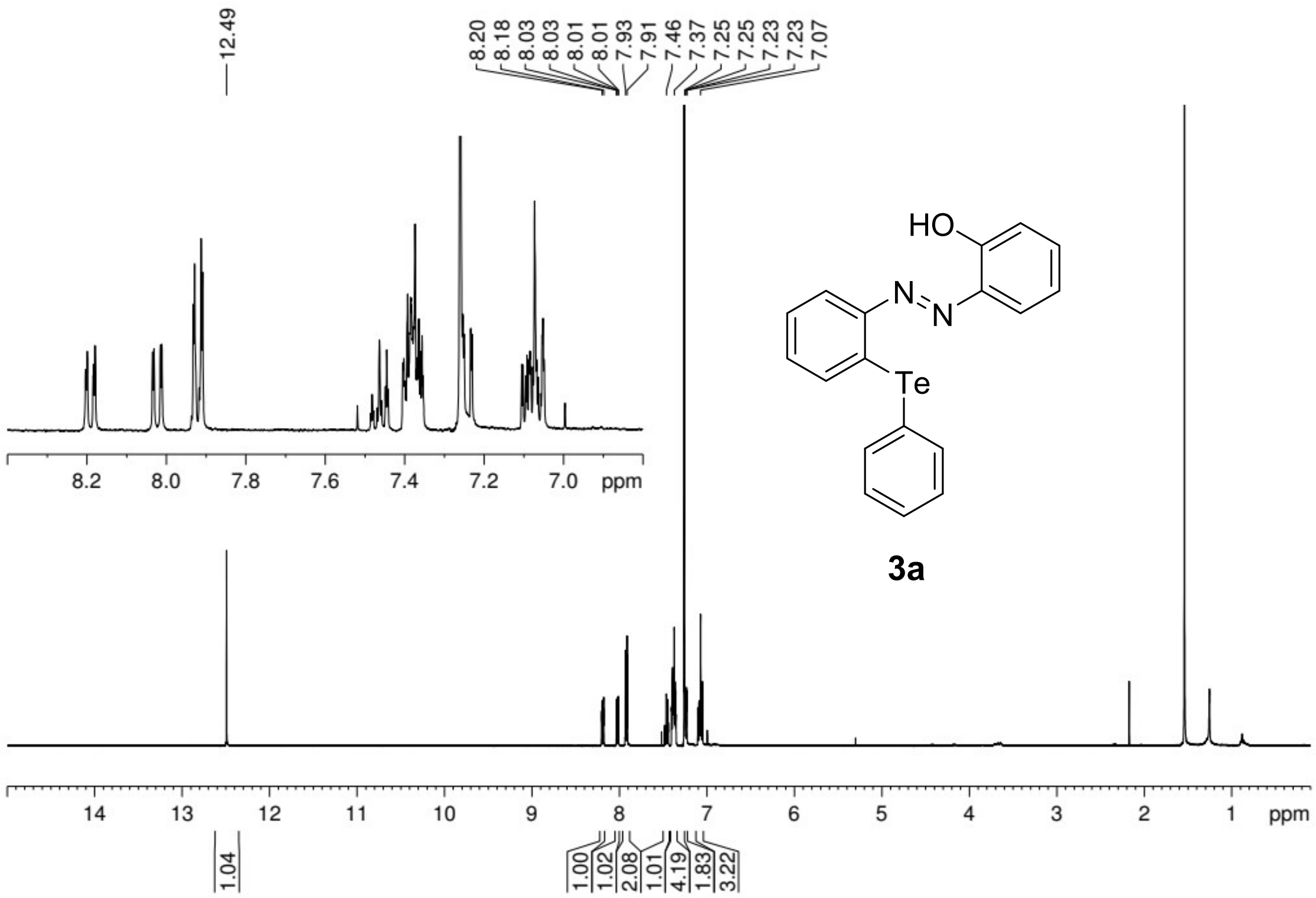
12.49
8.20
8.18
8.03
8.03
8.01
8.01
7.93
7.91
7.46
7.37
7.25
7.25
7.23
7.23
7.07
HO
N
N
Te
3a
8.2 8.0 7.8 7.6 7.4 7.2 7.0 ppm
14 13 12 11 10 9 8 7 6 5 4 3 2 1 ppm
1.04
1.00
1.02
2.08
1.01
4.19
1.83
3.22

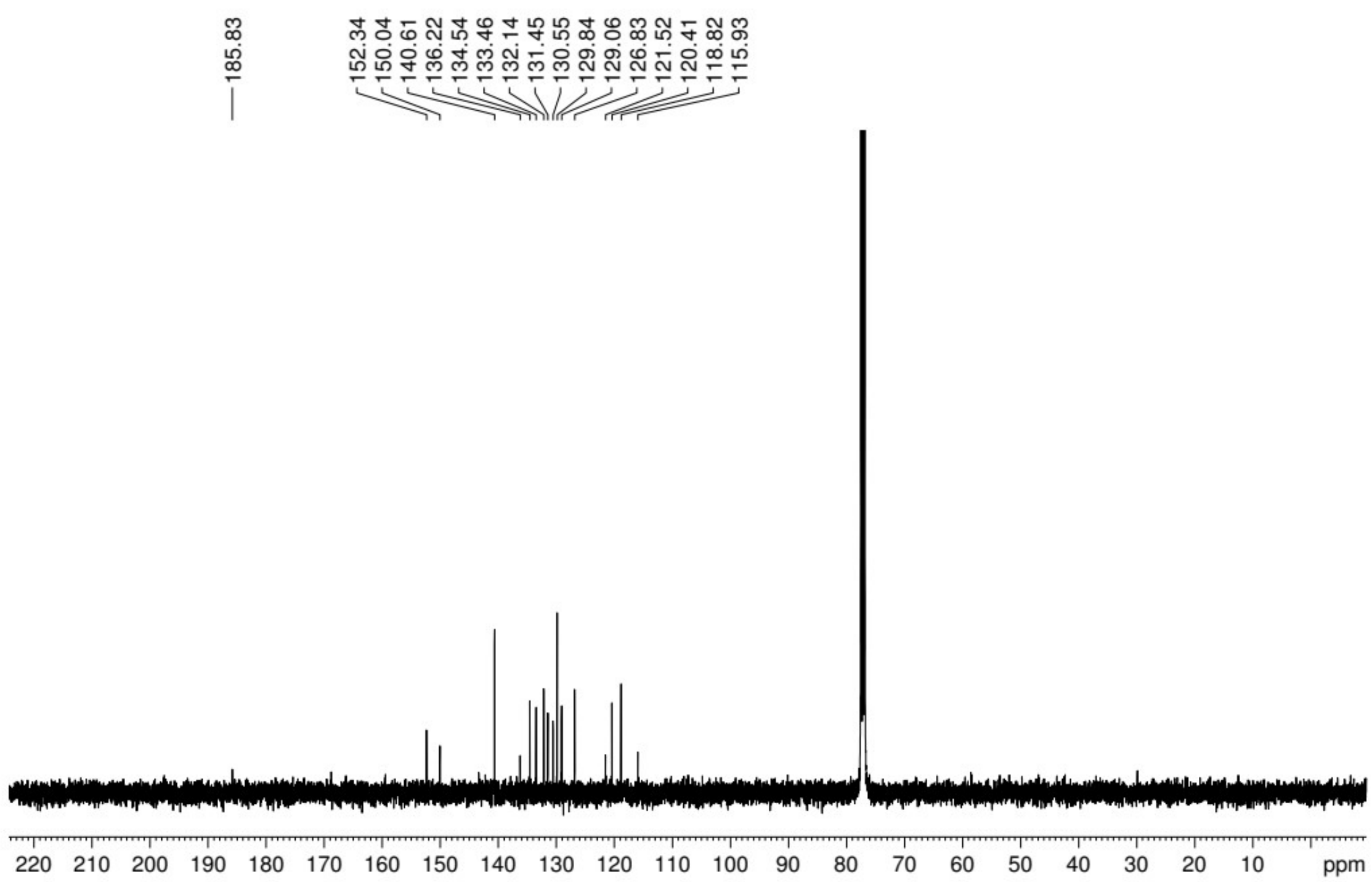
185.83
152.34
150.04
140.61
136.22
134.54
133.46
132.14
131.45
130.55
129.84
129.06
126.83
121.52
120.41
118.82
115.93
220 210 200 190 180 170 160 150 140 130 120 110 100 90 80 70 60 50 40 30 20 10 ppm

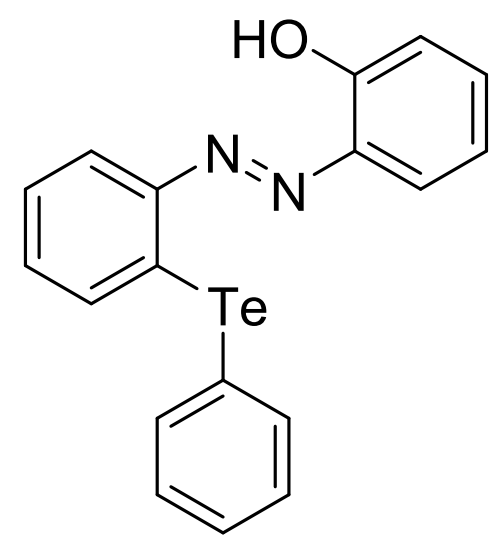

3a

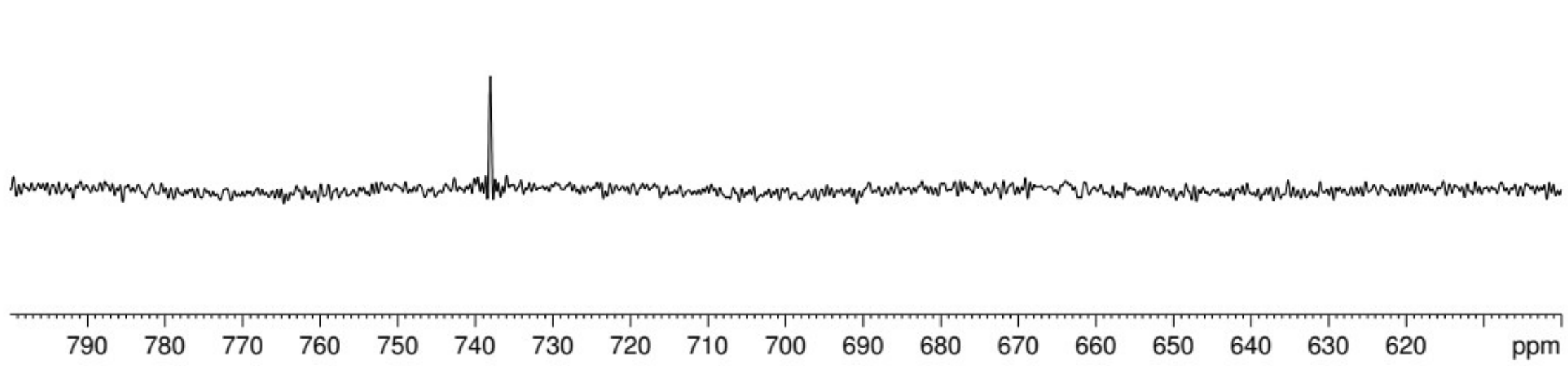

737.99
790
780
770
760
750
740
730
720
710
700
690
680
670
660
650
640
630
620
ppm

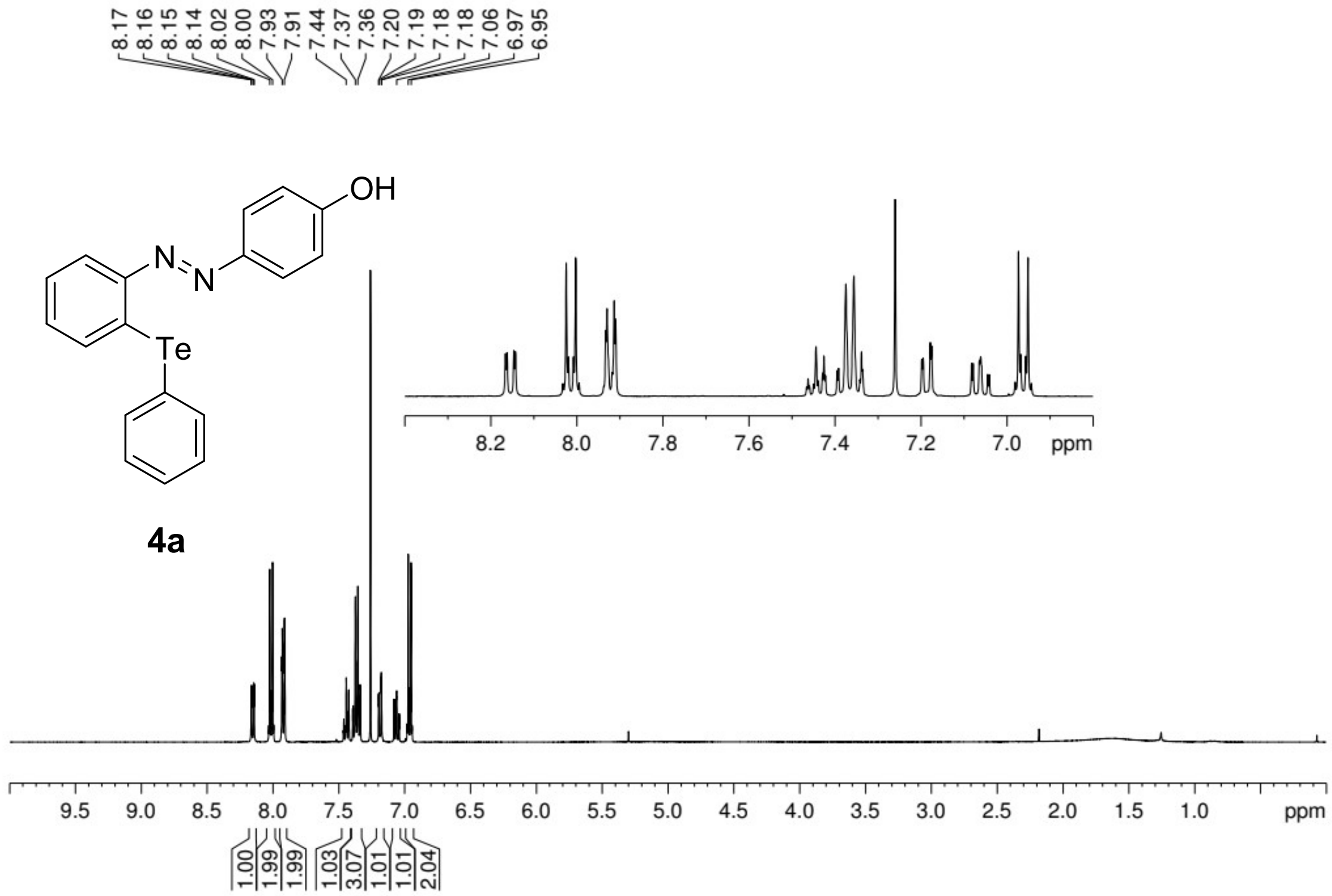
OH
N
N
Te
8.17
8.16
8.15
8.14
8.02
8.00
7.93
7.91
7.44
7.37
7.36
7.20
7.19
7.18
7.18
7.06
6.97
6.95
8.2 8.0 7.8 7.6 7.4 7.2 7.0 ppm
9.5 9.0 8.5 8.0 7.5 7.0 6.5 6.0 5.5 5.0 4.5 4.0 3.5 3.0 2.5 2.0 1.5 1.0 ppm
1.00
1.99
1.99
1.03
3.07
1.01
1.01
2.04

4a

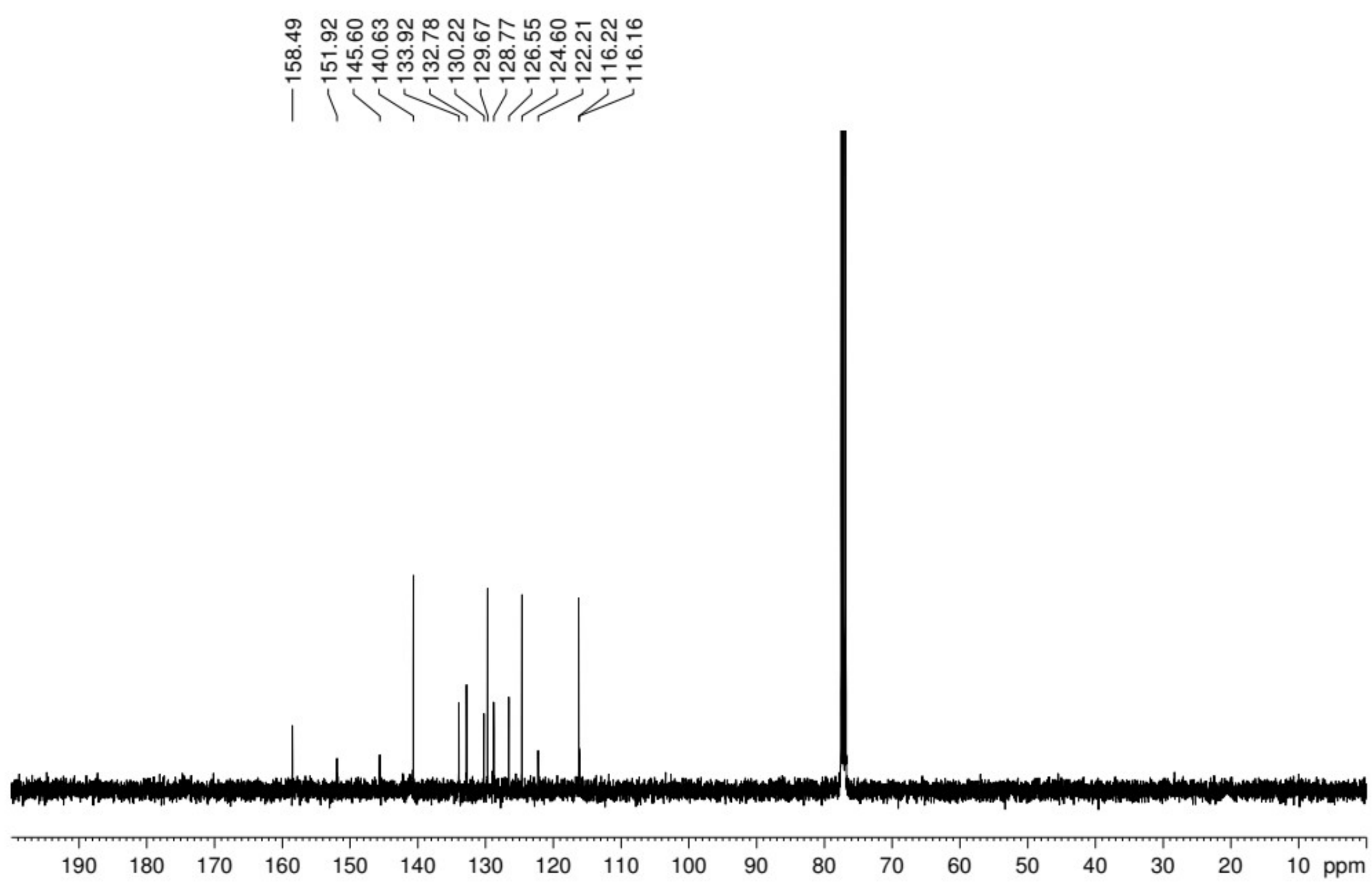
158.49
151.92
145.60
140.63
133.92
132.78
130.22
129.67
128.77
126.55
124.60
122.21
116.22
116.16
190 180 170 160 150 140 130 120 110 100 90 80 70 60 50 40 30 20 10 ppm

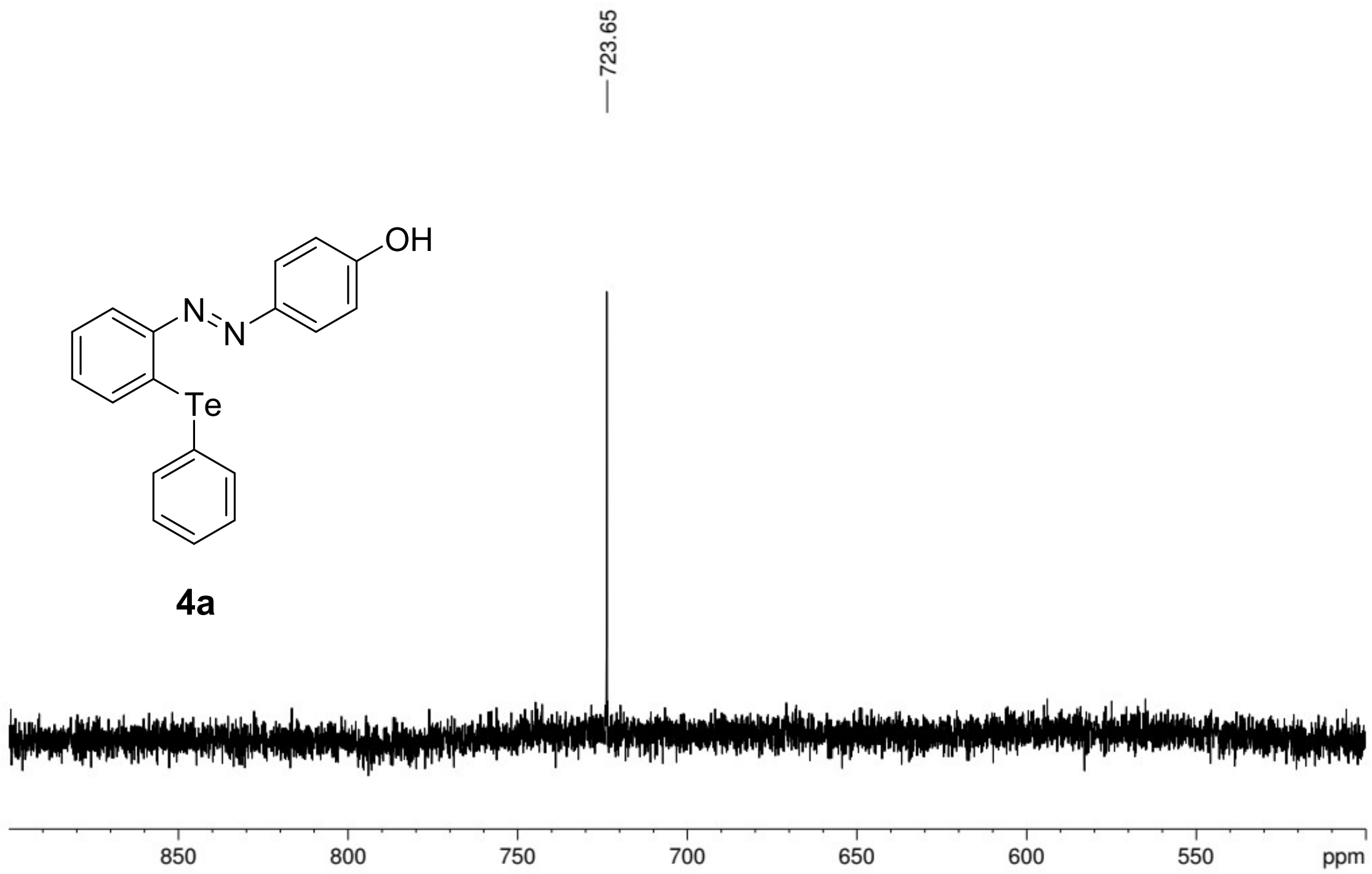
723.65
OH
N
N
Te
4a
850
800
750
700
650
600
550
ppm

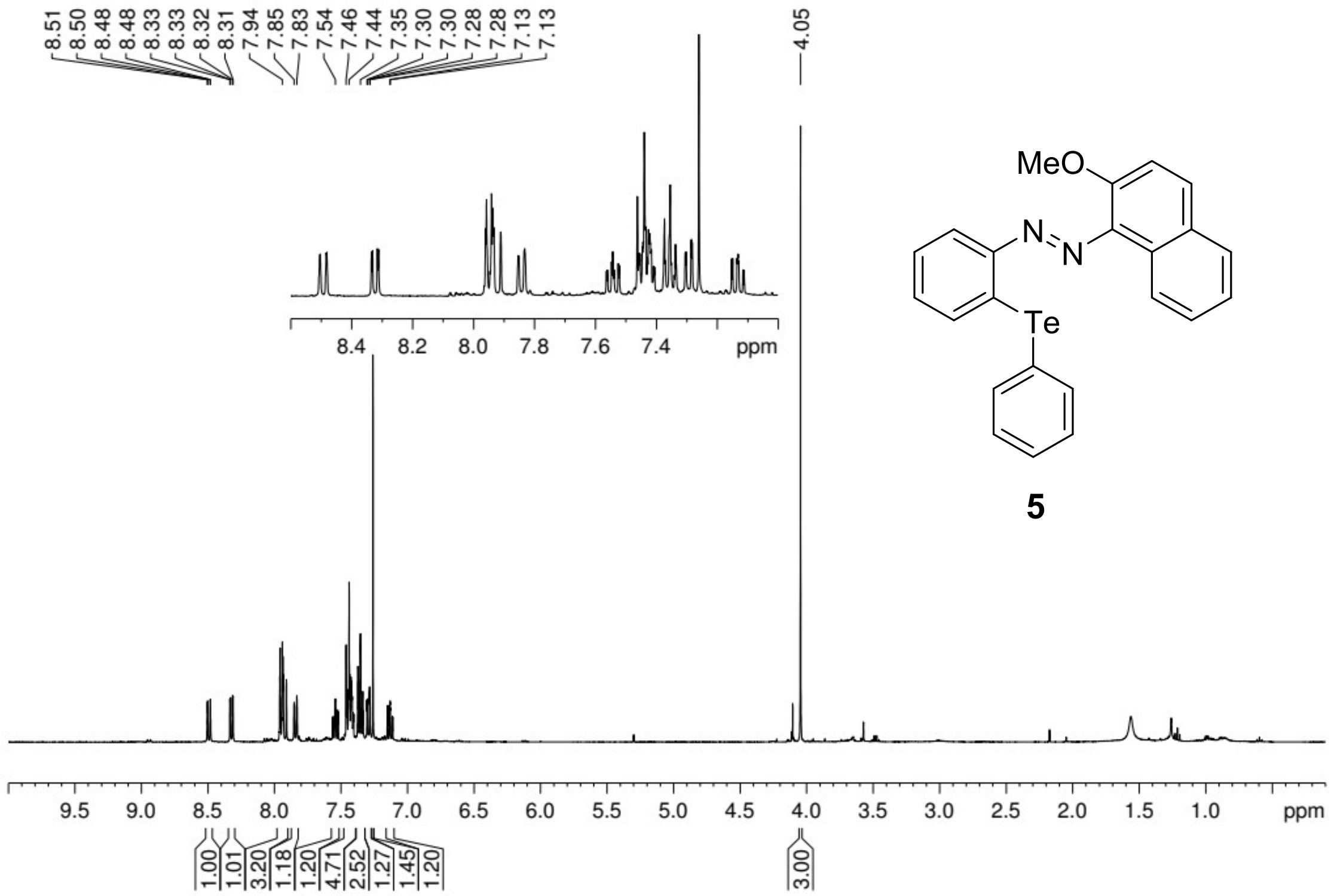
8.51 8.50 8.48 8.48 8.33 8.33 8.32 8.31 7.94 7.85 7.83 7.54 7.46 7.44 7.35 7.30 7.30 7.28 7.28 7.13 7.13
4.05
8.4 8.2 8.0 7.8 7.6 7.4 ppm
9.5 9.0 8.5 8.0 7.5 7.0 6.5 6.0 5.5 5.0 4.5 4.0 3.5 3.0 2.5 2.0 1.5 1.0 ppm
1.00 1.01 3.20 1.18 1.20 4.71 2.52 1.27 1.45 1.20
3.00
MeO
N
N
Te

5

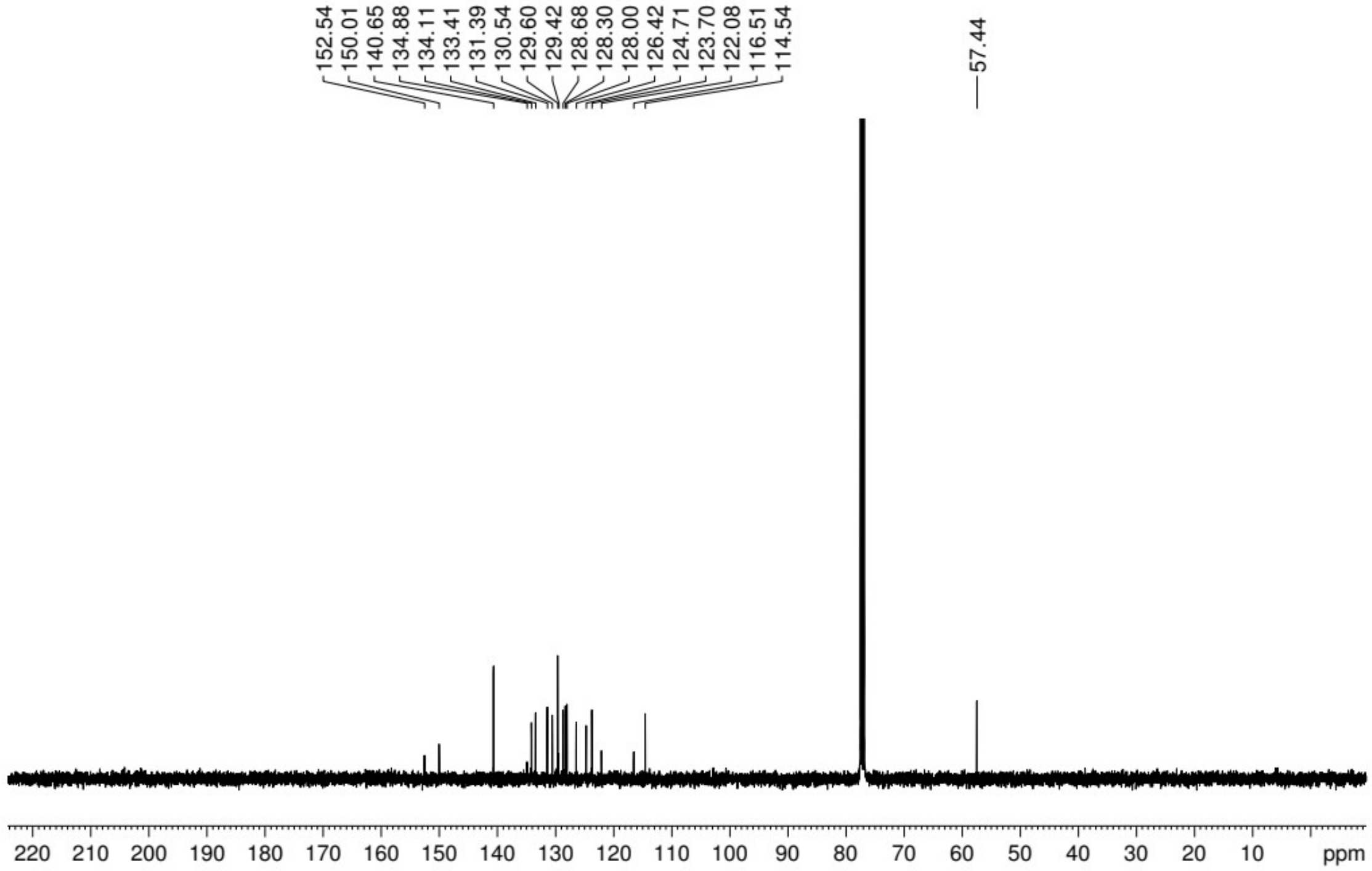
152.54 150.01 140.65 134.88 134.11 133.41 131.39 130.54 129.60 129.42 128.68 128.30 128.00 126.42 124.71 123.70 122.08 116.51 114.54
57.44
220 210 200 190 180 170 160 150 140 130 120 110 100 90 80 70 60 50 40 30 20 10 ppm

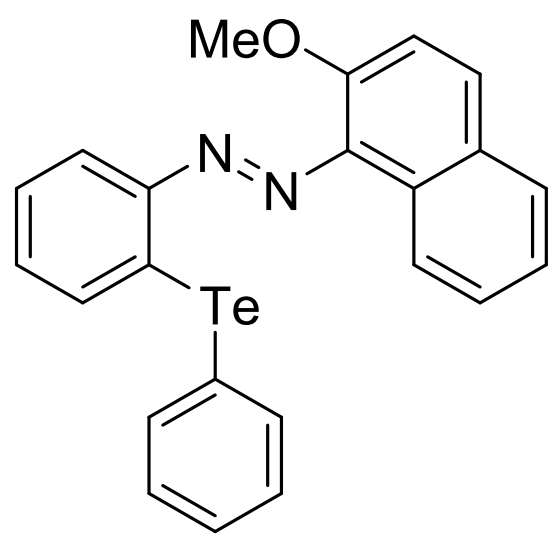

5

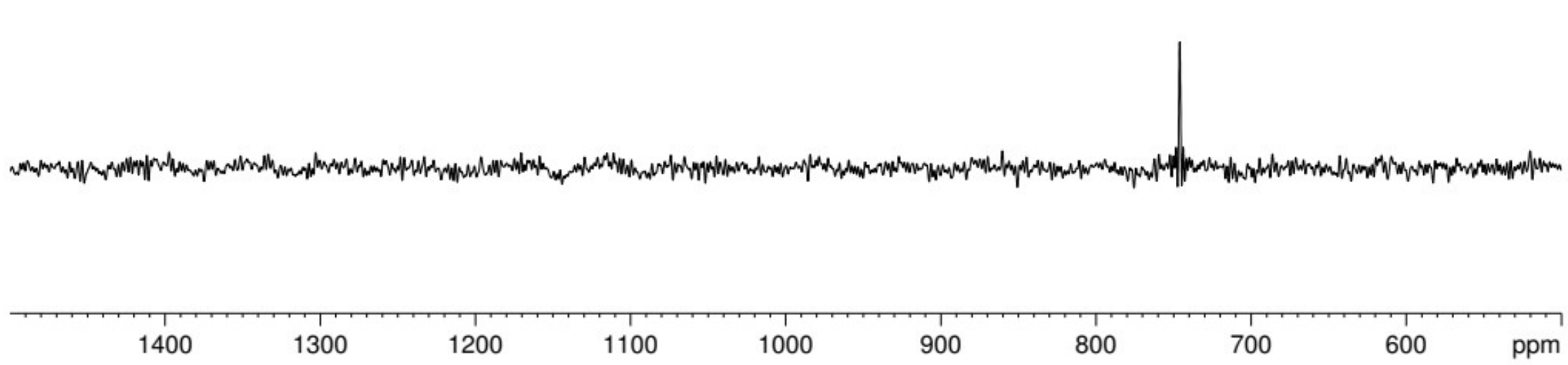

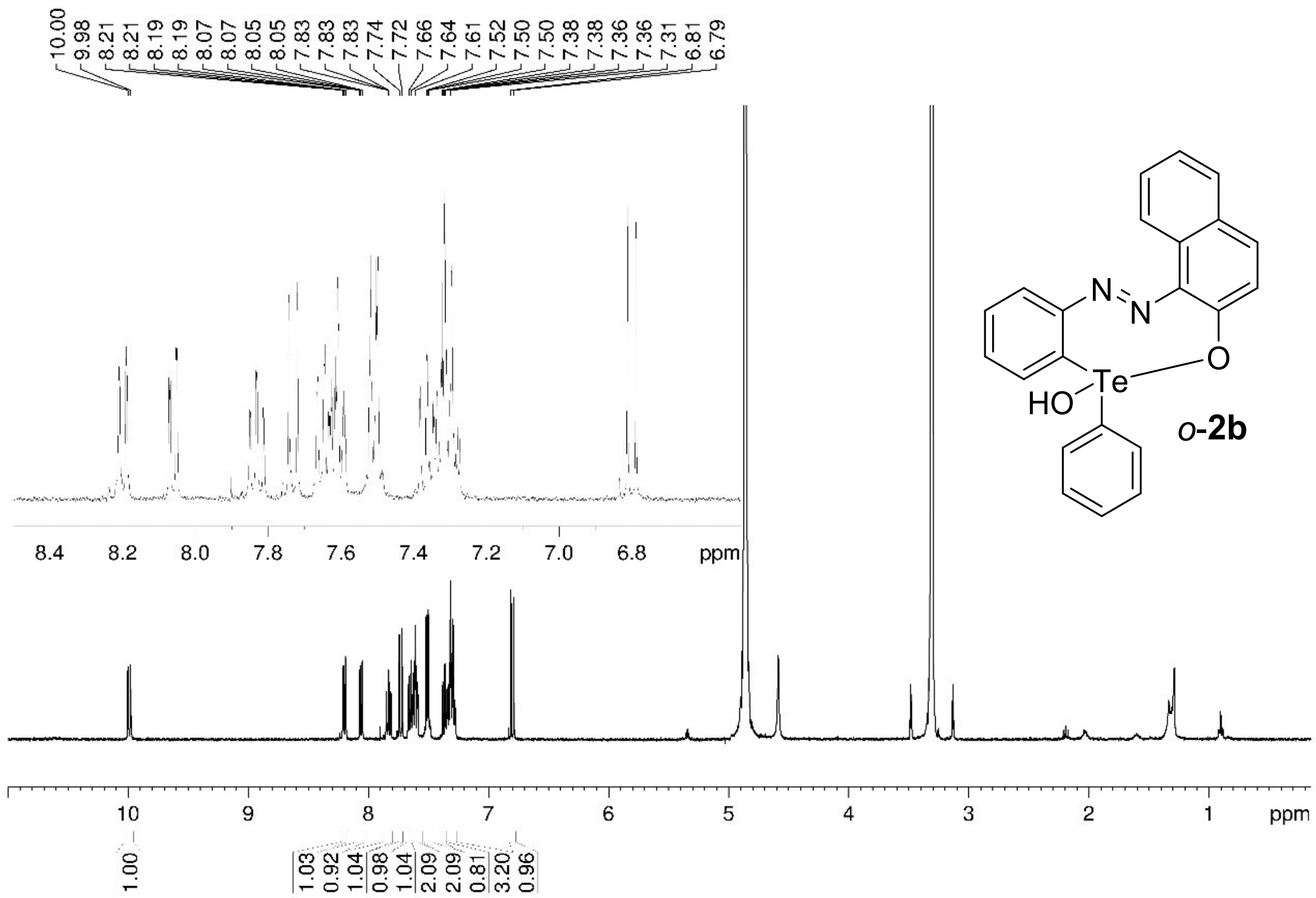
10.00
9.98
8.21
8.21
8.19
8.19
8.07
8.05
8.05
7.83
7.83
7.83
7.74
7.72
7.66
7.64
7.61
7.52
7.50
7.50
7.38
7.38
7.36
7.36
7.31
6.81
6.79
8.4 8.2 8.0 7.8 7.6 7.4 7.2 7.0 6.8 ppm
10 9 8 7 6 5 4 3 2 1 ppm
1.00
1.03
0.92
1.04
0.98
1.04
2.09
2.09
0.81
3.20
0.96
N
N
O
Te
HO
o-2b

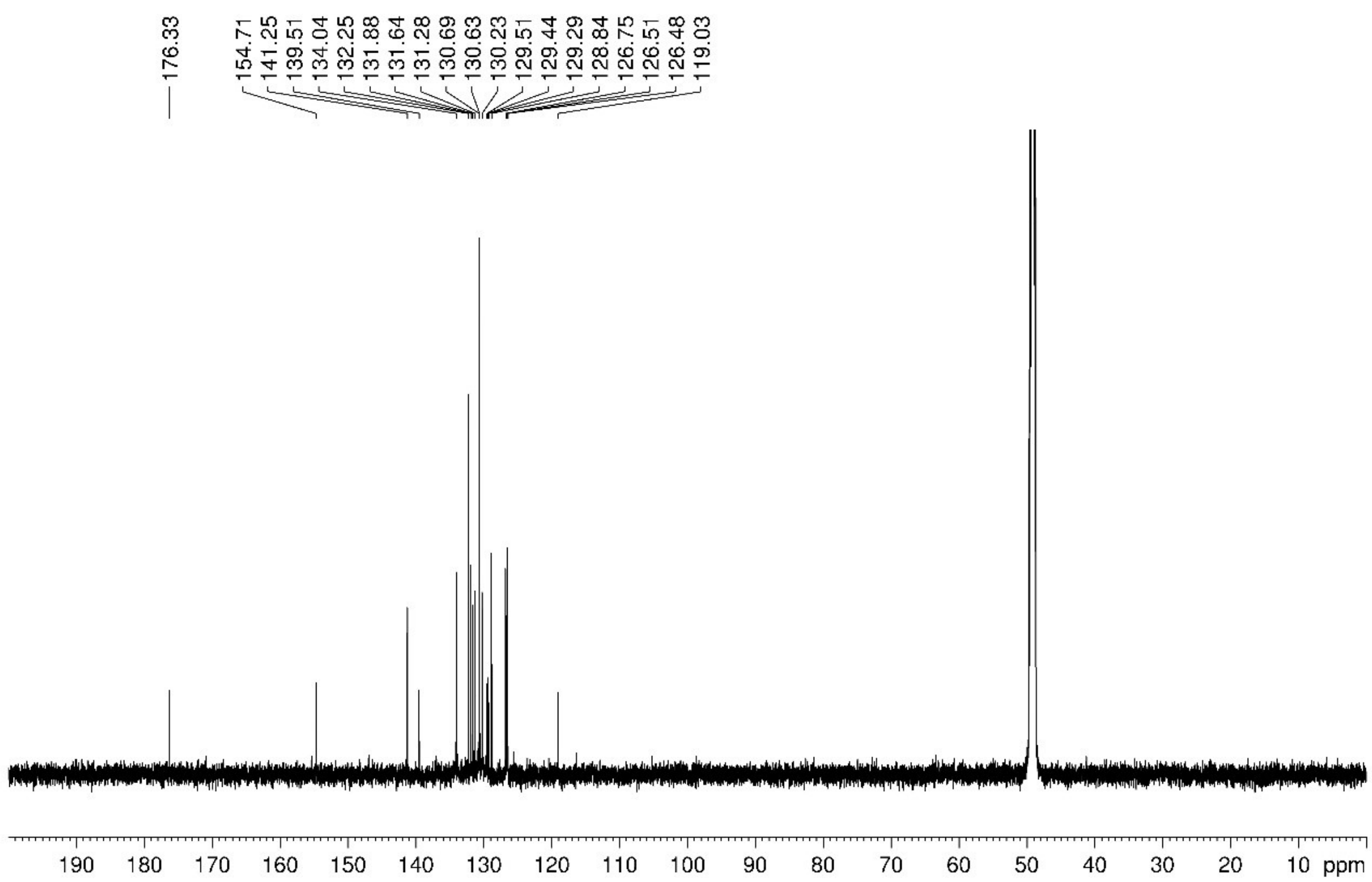
176.33
154.71
141.25
139.51
134.04
132.25
131.88
131.64
131.28
130.69
130.63
130.23
129.51
129.44
129.29
128.84
126.75
126.51
126.48
119.03
190 180 170 160 150 140 130 120 110 100 90 80 70 60 50 40 30 20 10 ppm

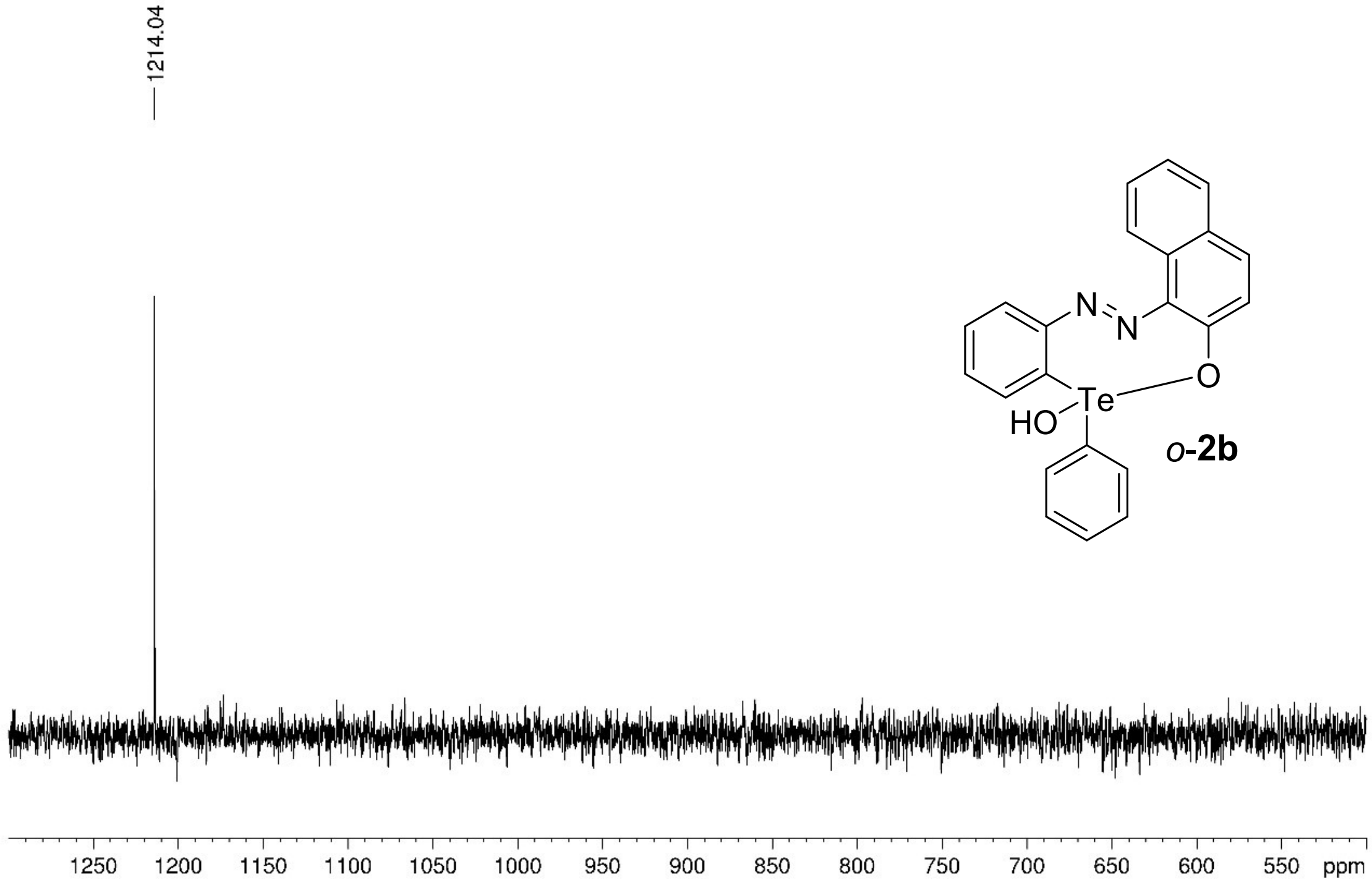
1214.04
N
N
O
Te
HO
o-2b
1250 1200 1150 1100 1050 1000 950 900 850 800 750 700 650 600 550 ppm

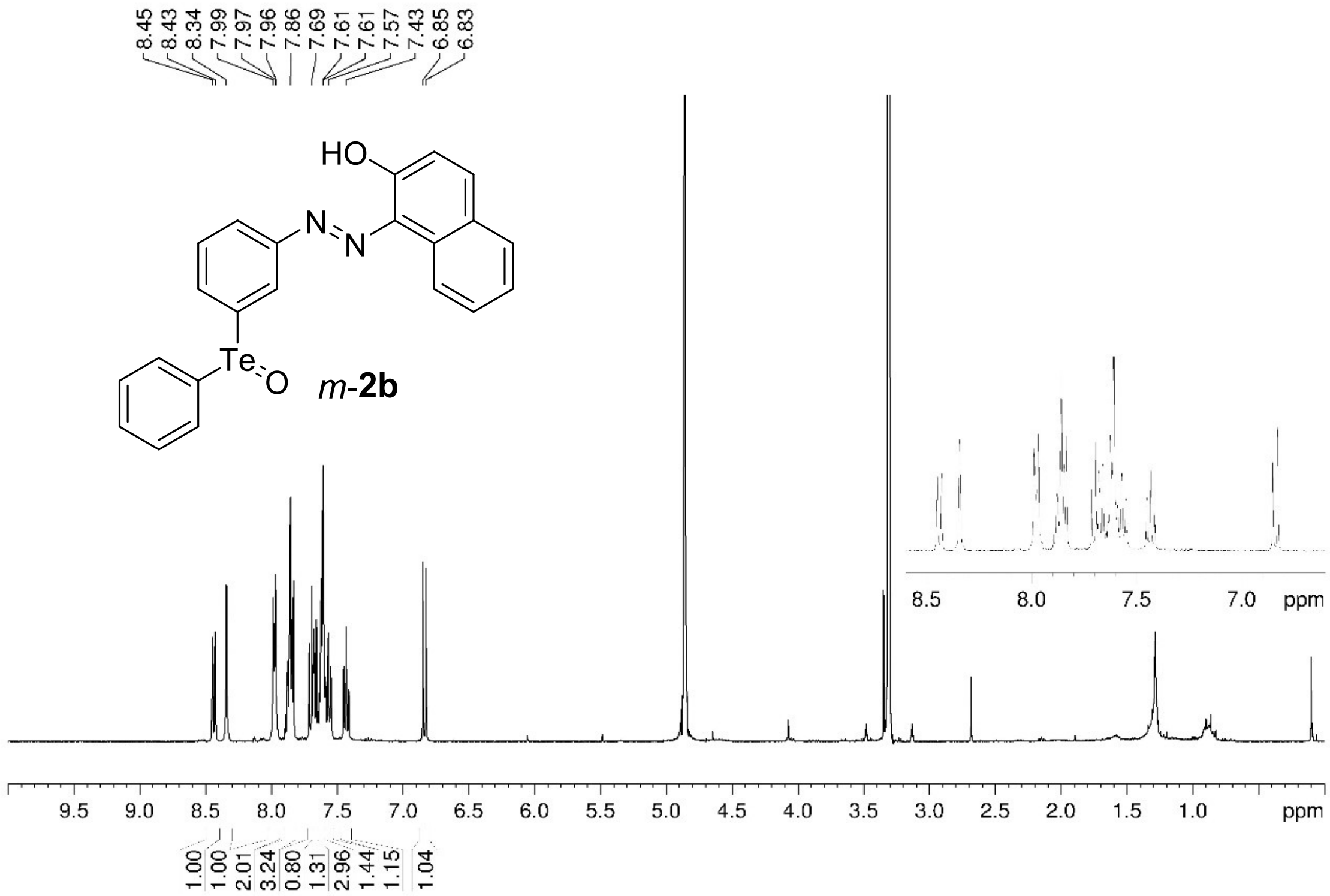
8.45
8.43
8.34
7.99
7.97
7.96
7.86
7.69
7.61
7.61
7.57
7.43
6.85
6.83
HO
N
N
Te
O
m-**2b**
8.5
8.0
7.5
7.0
ppm
9.5
9.0
8.5
8.0
7.5
7.0
6.5
6.0
5.5
5.0
4.5
4.0
3.5
3.0
2.5
2.0
1.5
1.0
ppm
1.00
1.00
2.01
3.24
0.80
1.31
2.96
1.44
1.15
1.04

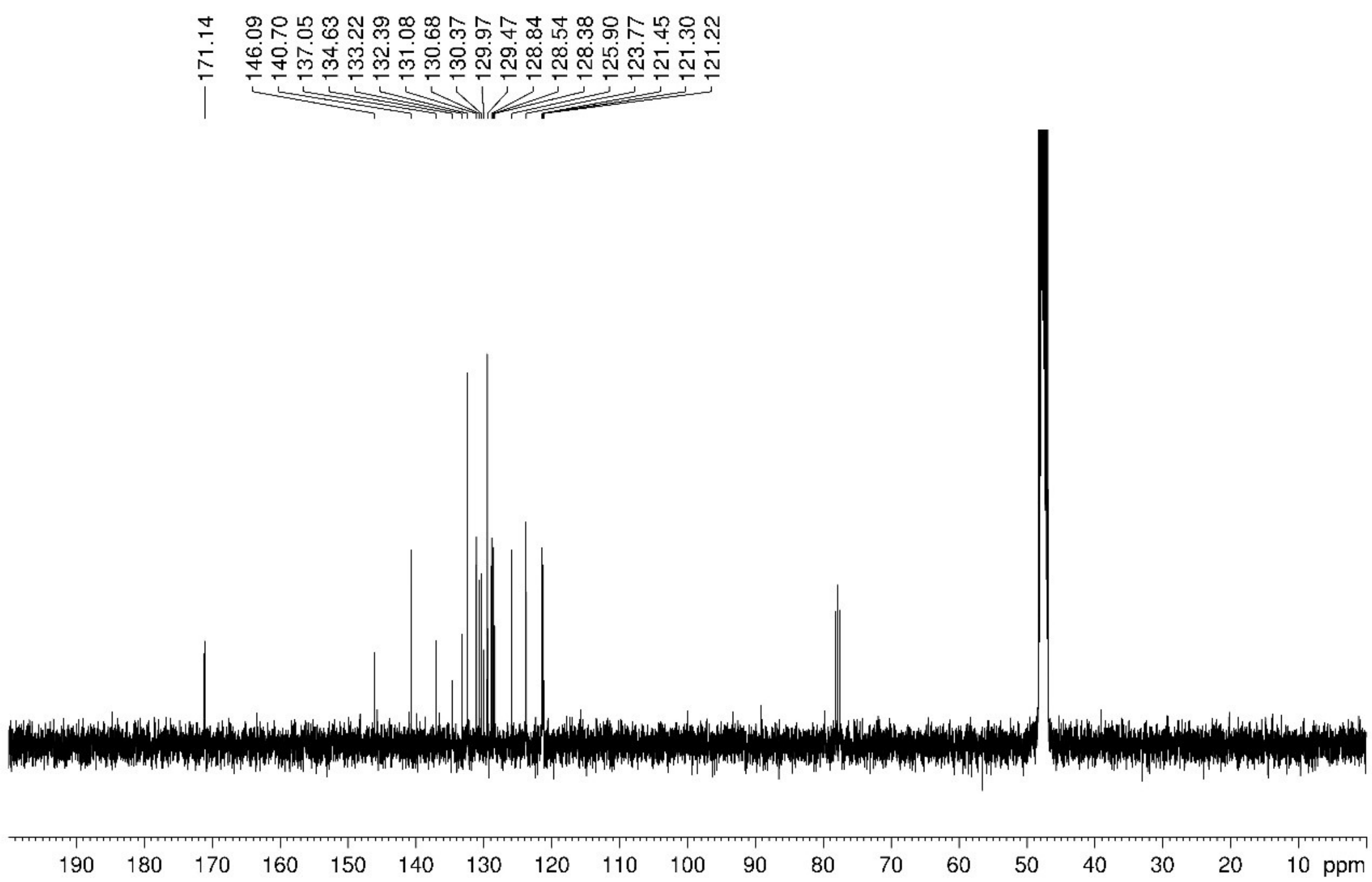
171.14
146.09
140.70
137.05
134.63
133.22
132.39
131.08
130.68
130.37
129.97
129.47
128.84
128.54
128.38
125.90
123.77
121.45
121.30
121.22
190
180
170
160
150
140
130
120
110
100
90
80
70
60
50
40
30
20
10
ppm

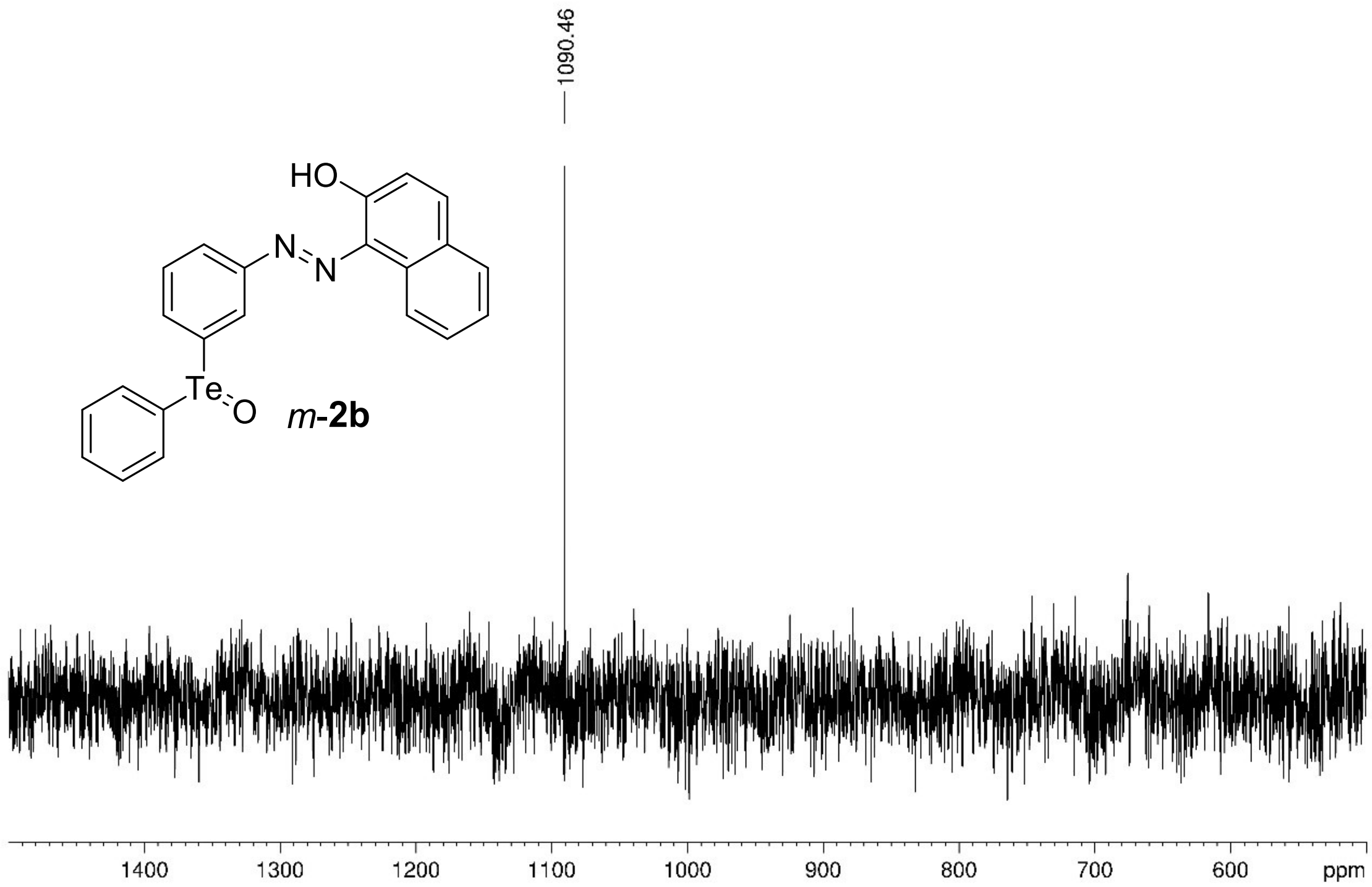

m-**2b**
1090.46
1400
1300
1200
1100
1000
900
800
700
600
ppm

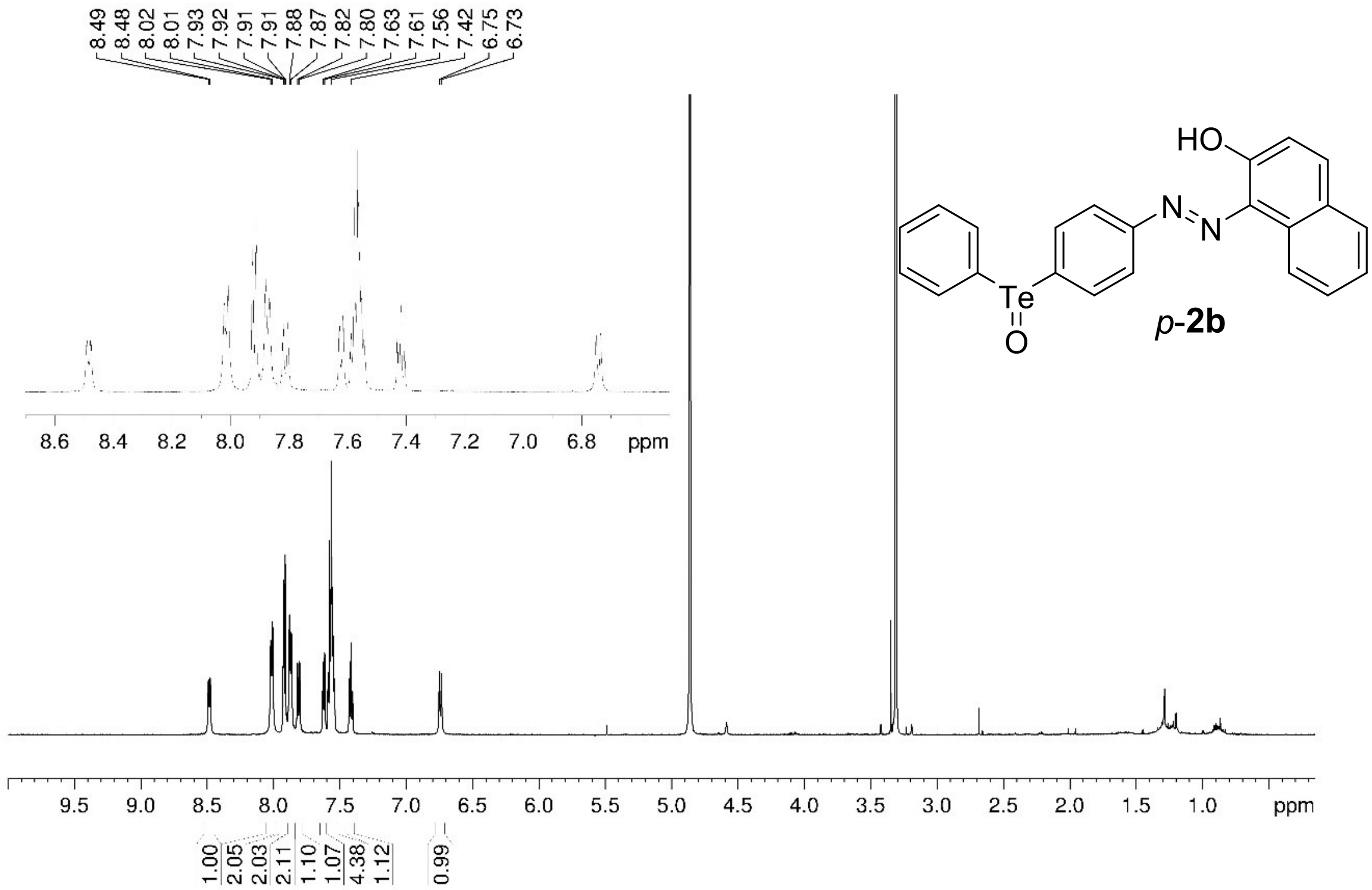
8.49 8.48 8.02 8.01 7.93 7.92 7.91 7.91 7.88 7.87 7.82 7.80 7.63 7.61 7.56 7.42 6.75 6.73
8.6 8.4 8.2 8.0 7.8 7.6 7.4 7.2 7.0 6.8 ppm
HO
N
N
Te
O
9.5 9.0 8.5 8.0 7.5 7.0 6.5 6.0 5.5 5.0 4.5 4.0 3.5 3.0 2.5 2.0 1.5 1.0 ppm
1.00 2.05 2.03 2.11 1.10 1.07 4.38 1.12 0.99

p-**2b**

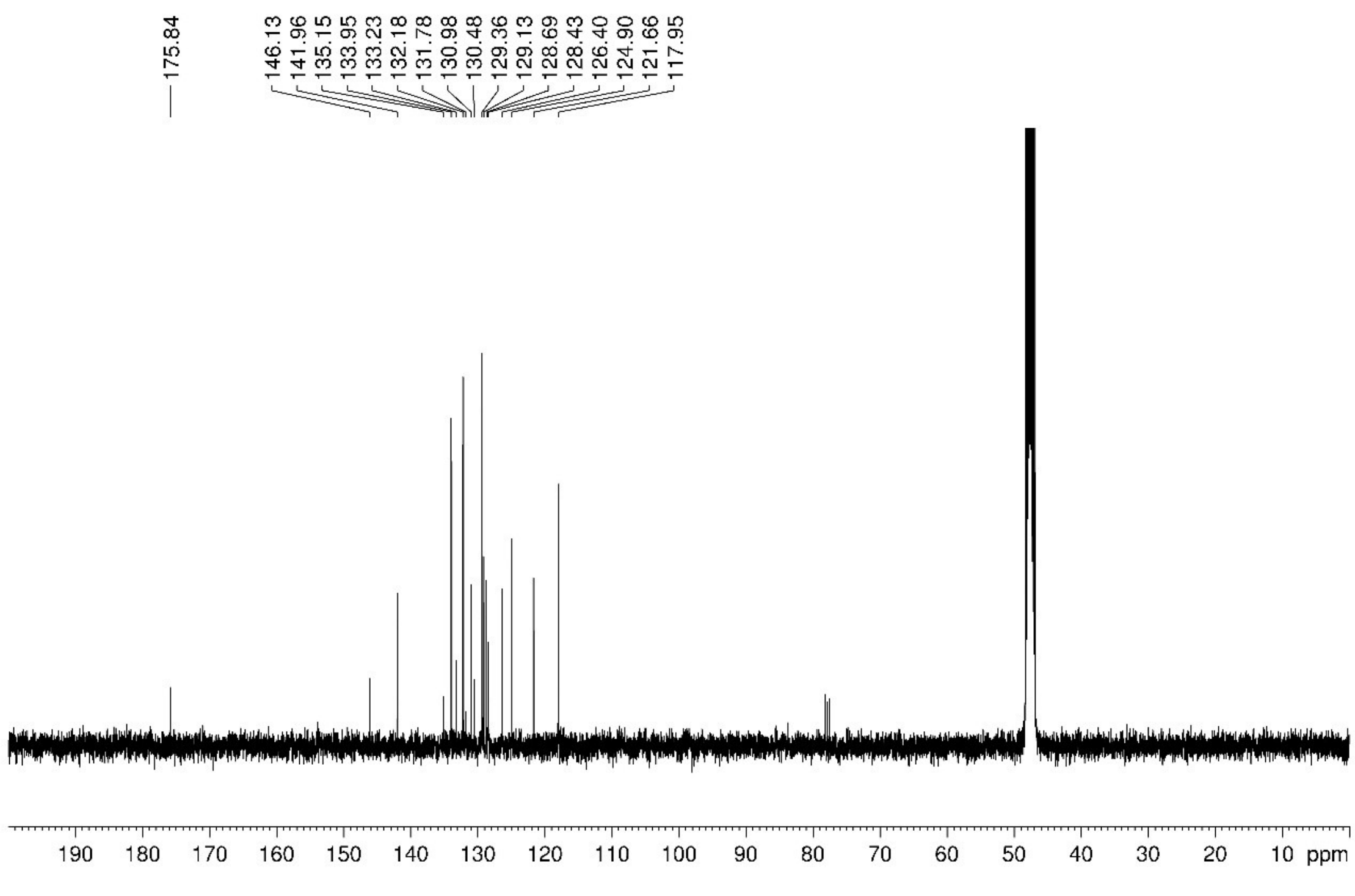
175.84
146.13 141.96 135.15 133.95 133.23 132.18 131.78 130.98 130.48 129.36 129.13 128.69 128.43 126.40 124.90 121.66 117.95
190 180 170 160 150 140 130 120 110 100 90 80 70 60 50 40 30 20 10 ppm

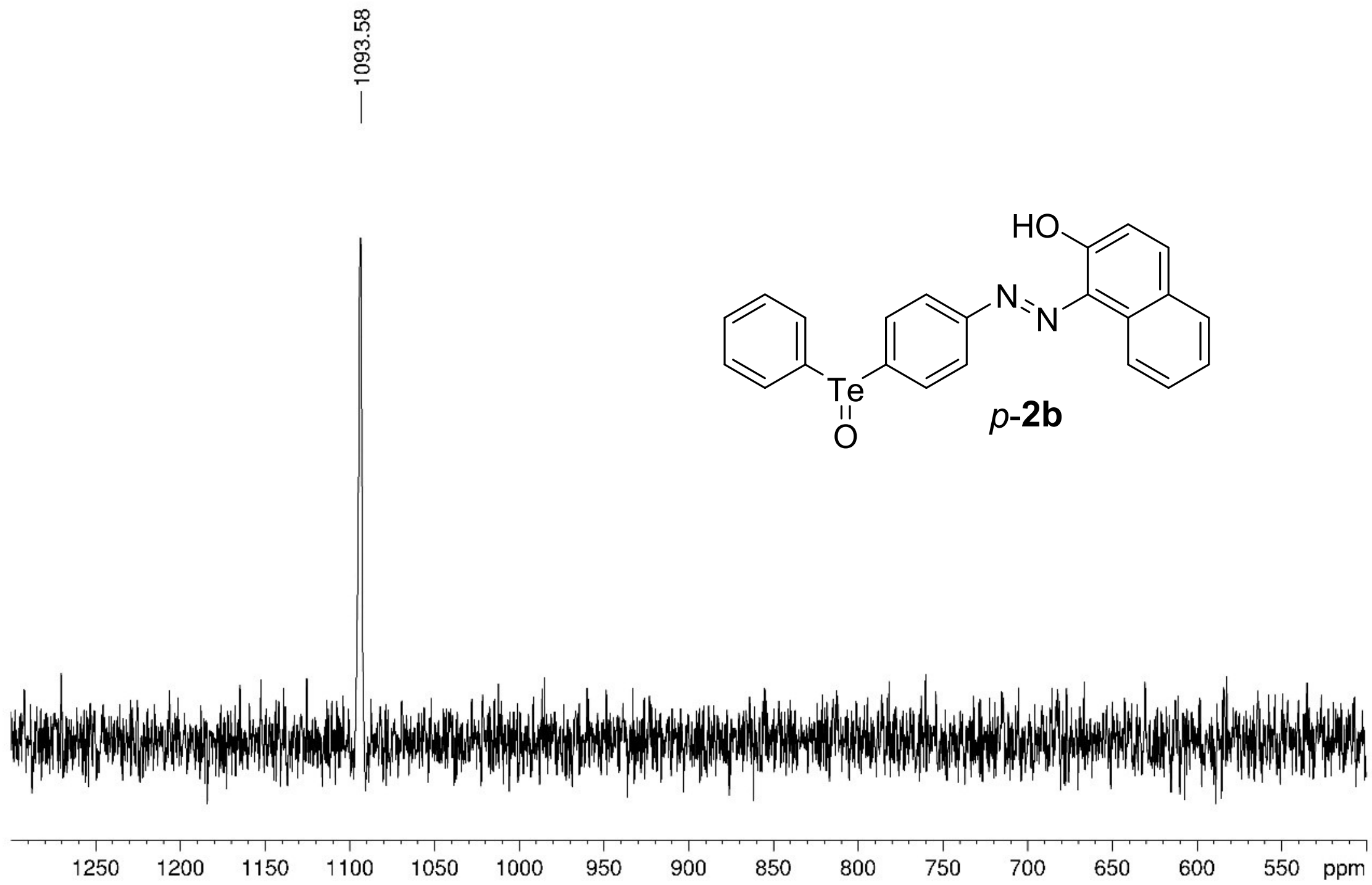
1093.58
HO
N
N
Te
O
p-2b
1250 1200 1150 1100 1050 1000 950 900 850 800 750 700 650 600 550 ppm

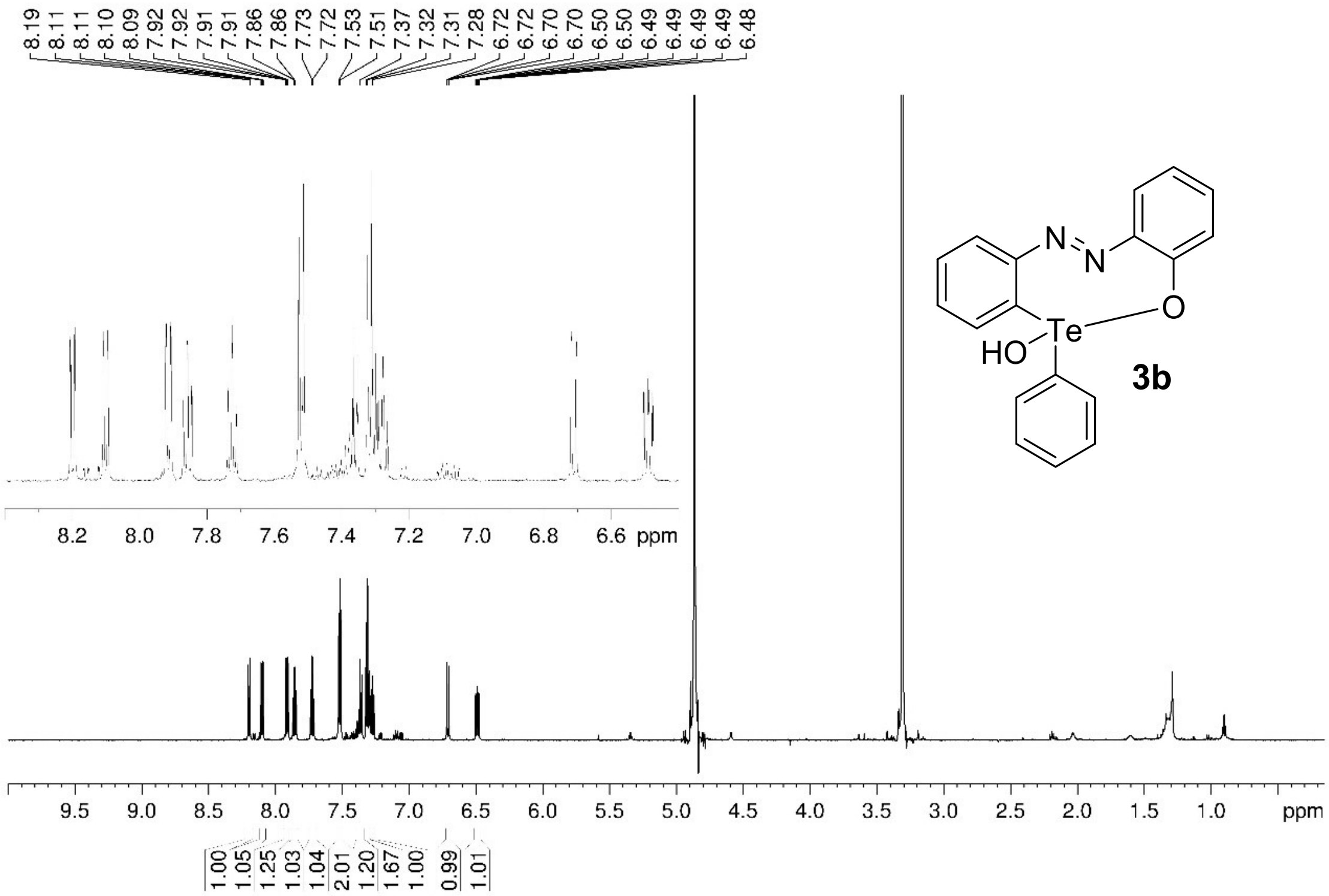
8.19 8.11 8.11 8.10 8.09 7.92 7.92 7.91 7.91 7.86 7.86 7.73 7.72 7.53 7.51 7.37 7.32 7.31 7.28 6.72 6.72 6.70 6.70 6.50 6.50 6.49 6.49 6.49 6.49 6.48
8.2 8.0 7.8 7.6 7.4 7.2 7.0 6.8 6.6 ppm
N N O Te HO
3b
9.5 9.0 8.5 8.0 7.5 7.0 6.5 6.0 5.5 5.0 4.5 4.0 3.5 3.0 2.5 2.0 1.5 1.0 ppm
1.00 1.05 1.25 1.03 1.04 2.01 1.20 1.67 1.00 0.99 1.01

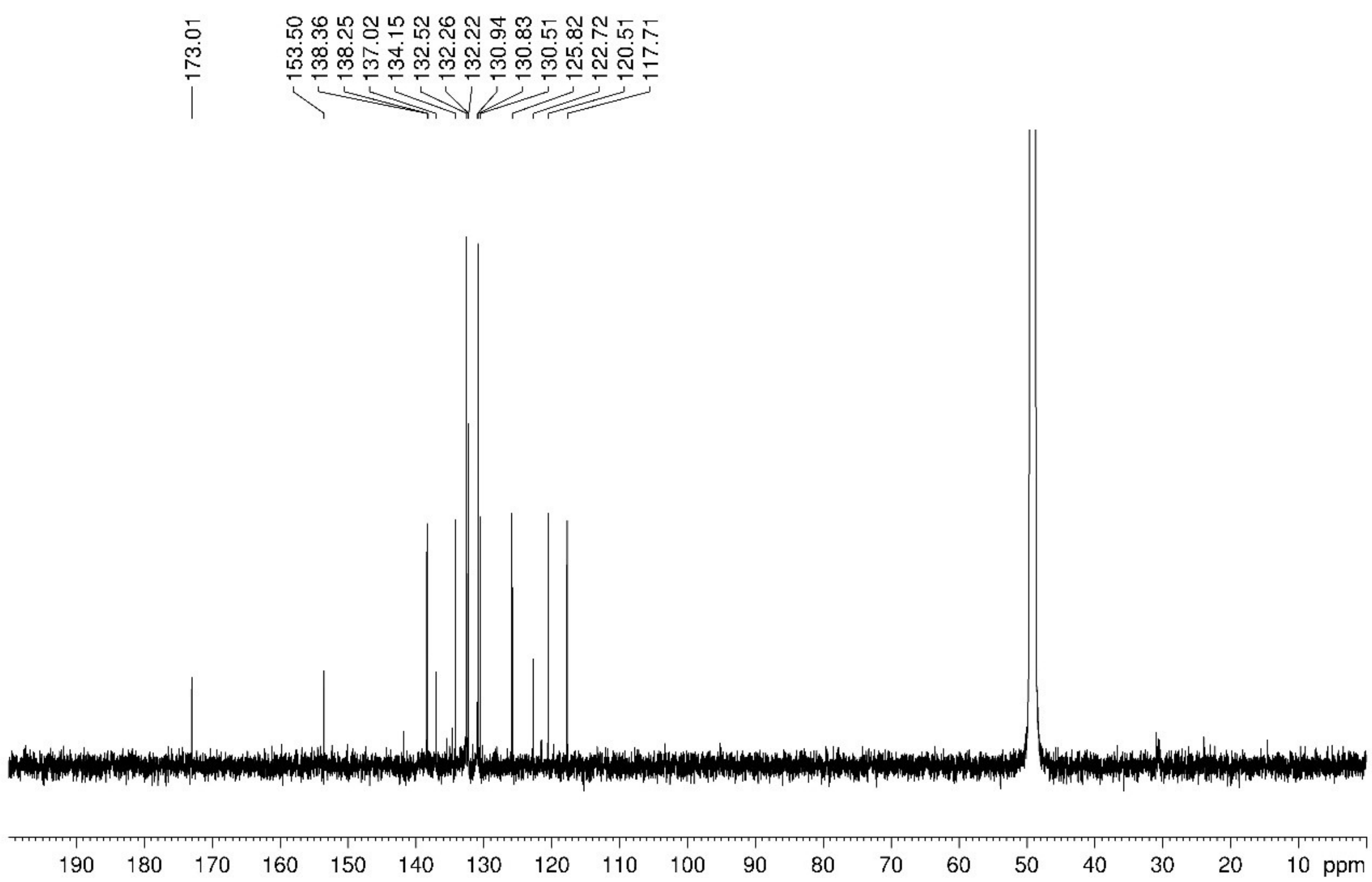
173.01
153.50 138.36 138.25 137.02 134.15 132.52 132.26 132.22 130.94 130.83 130.51 125.82 122.72 120.51 117.71
190 180 170 160 150 140 130 120 110 100 90 80 70 60 50 40 30 20 10 ppm

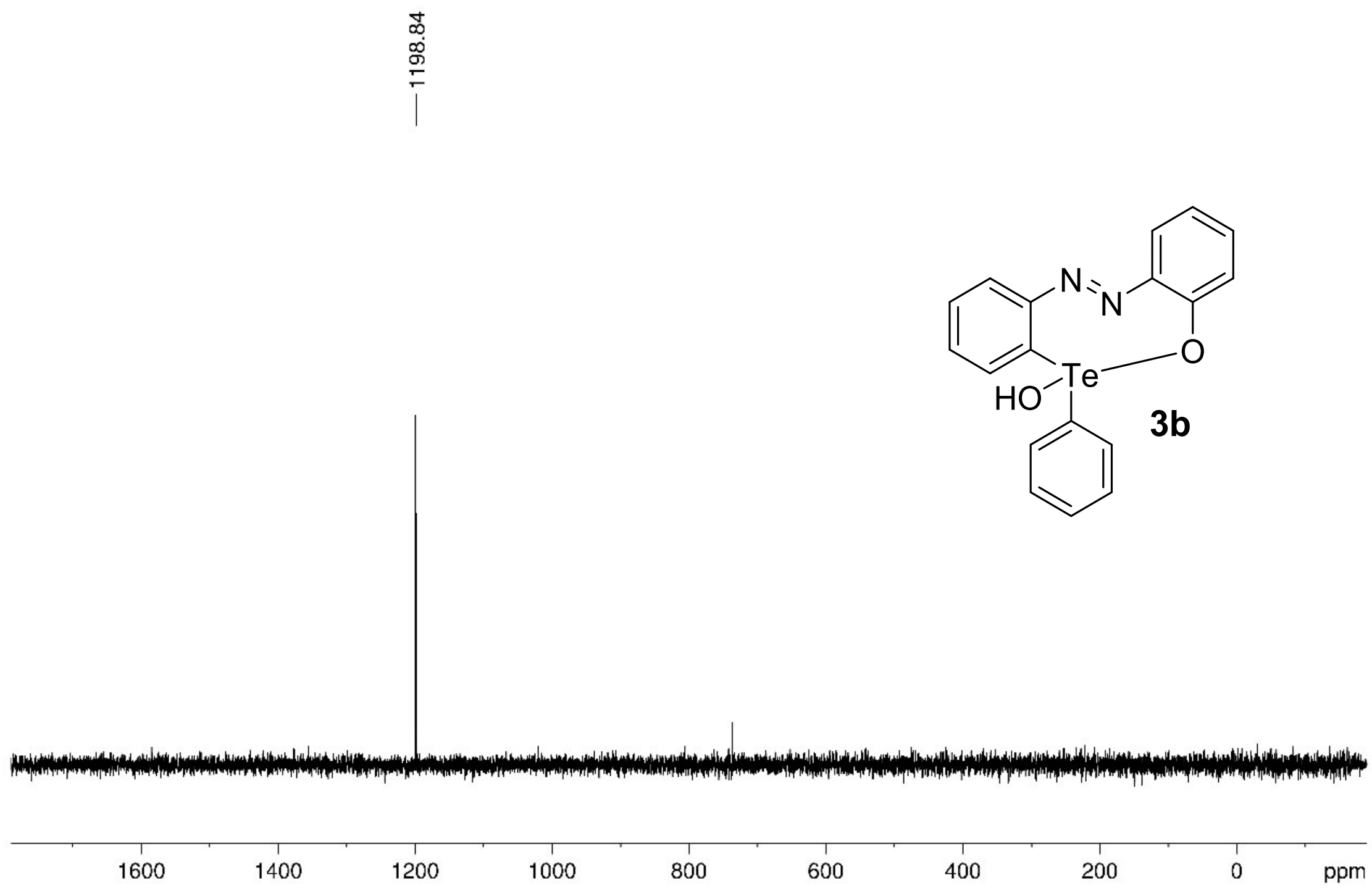
1198.84
N
N
O
Te
HO
3b
1600
1400
1200
1000
800
600
400
200
0
ppm

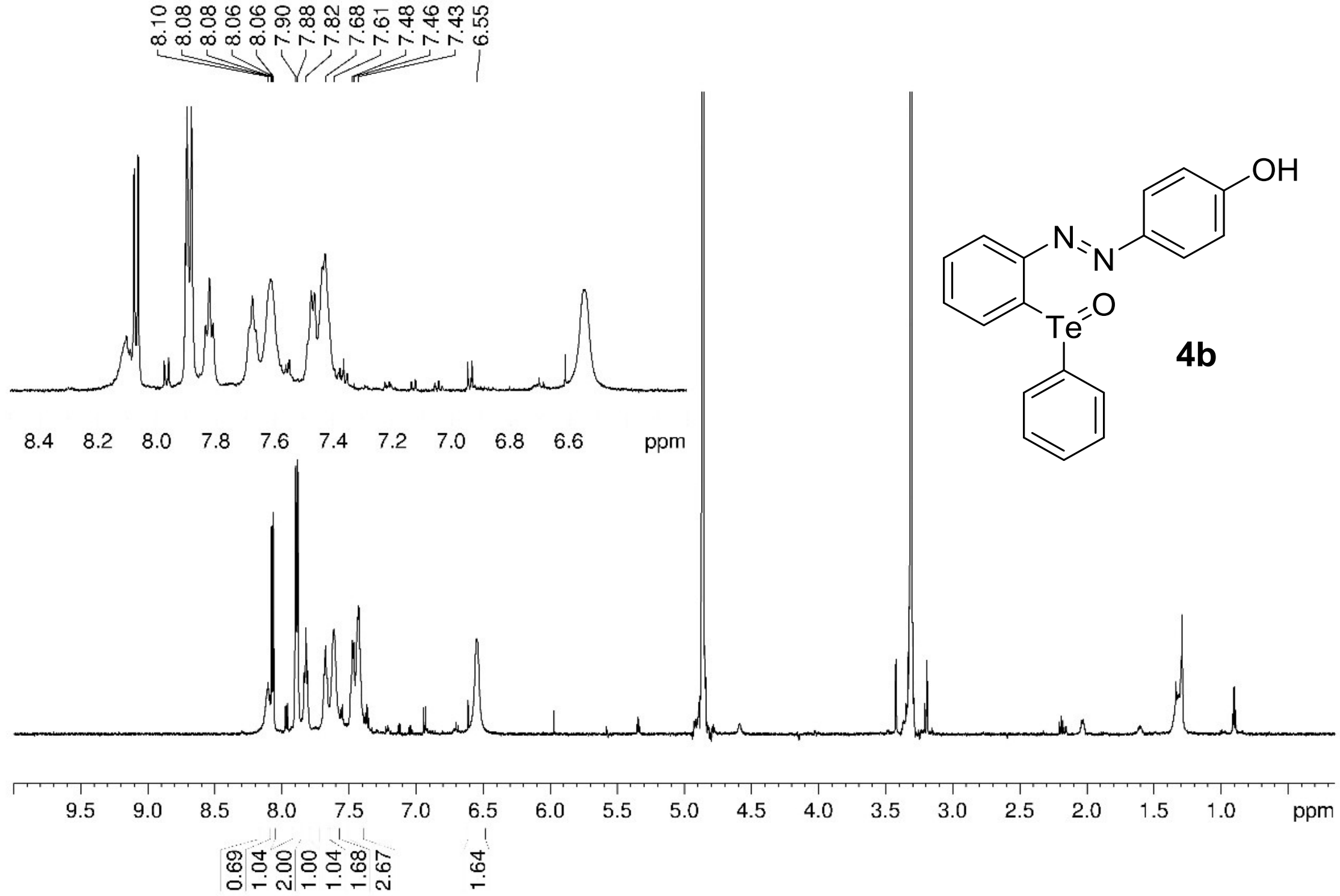
8.10
8.08
8.08
8.06
8.06
7.90
7.88
7.82
7.68
7.61
7.48
7.46
7.43
6.55
8.4 8.2 8.0 7.8 7.6 7.4 7.2 7.0 6.8 6.6 ppm
OH
N
N
Te
O
4b
9.5 9.0 8.5 8.0 7.5 7.0 6.5 6.0 5.5 5.0 4.5 4.0 3.5 3.0 2.5 2.0 1.5 1.0 ppm
0.69
1.04
2.00
1.00
1.04
1.68
2.67
1.64

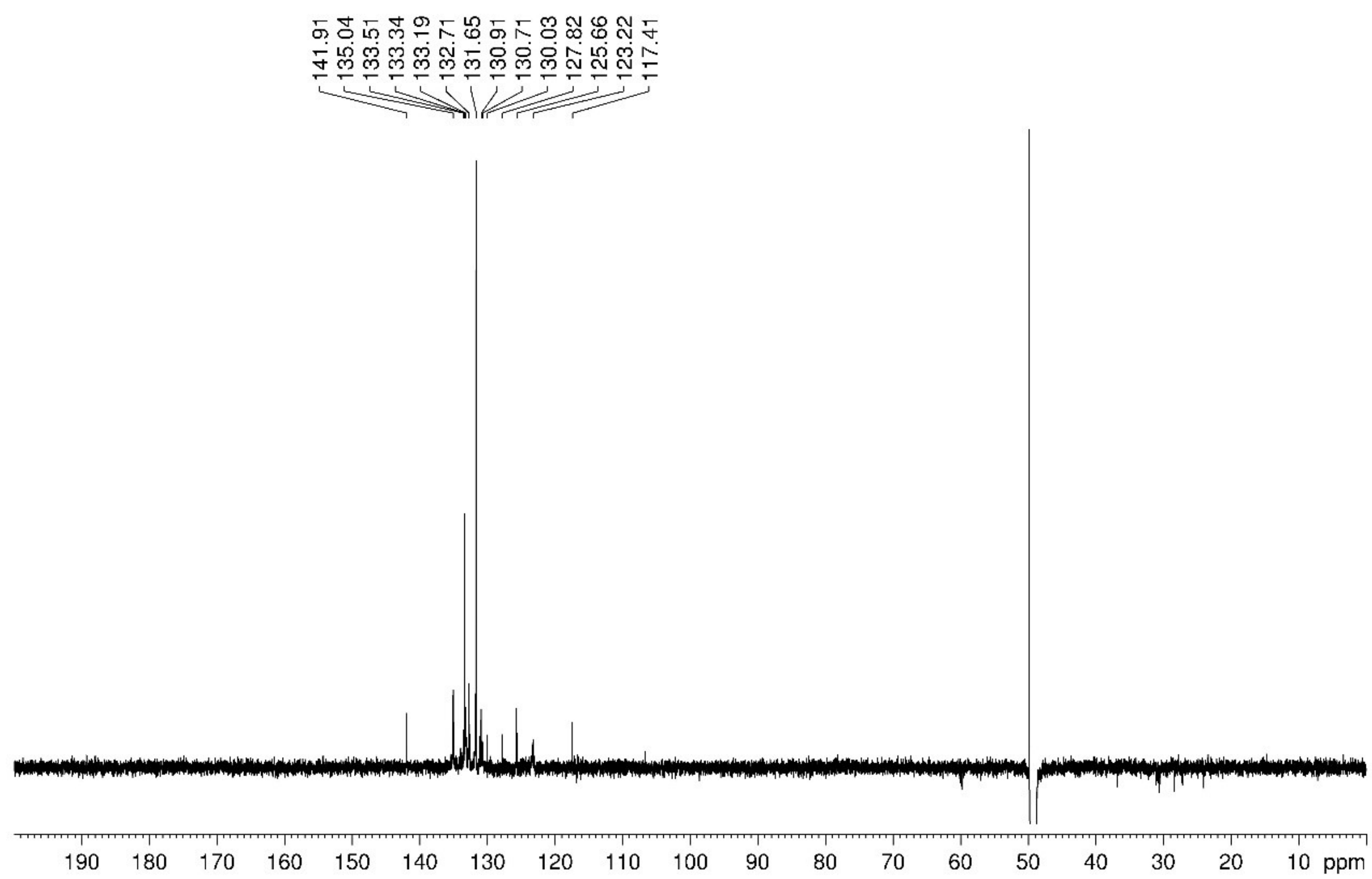
141.91
135.04
133.51
133.34
133.19
132.71
131.65
130.91
130.71
130.03
127.82
125.66
123.22
117.41
190 180 170 160 150 140 130 120 110 100 90 80 70 60 50 40 30 20 10 ppm

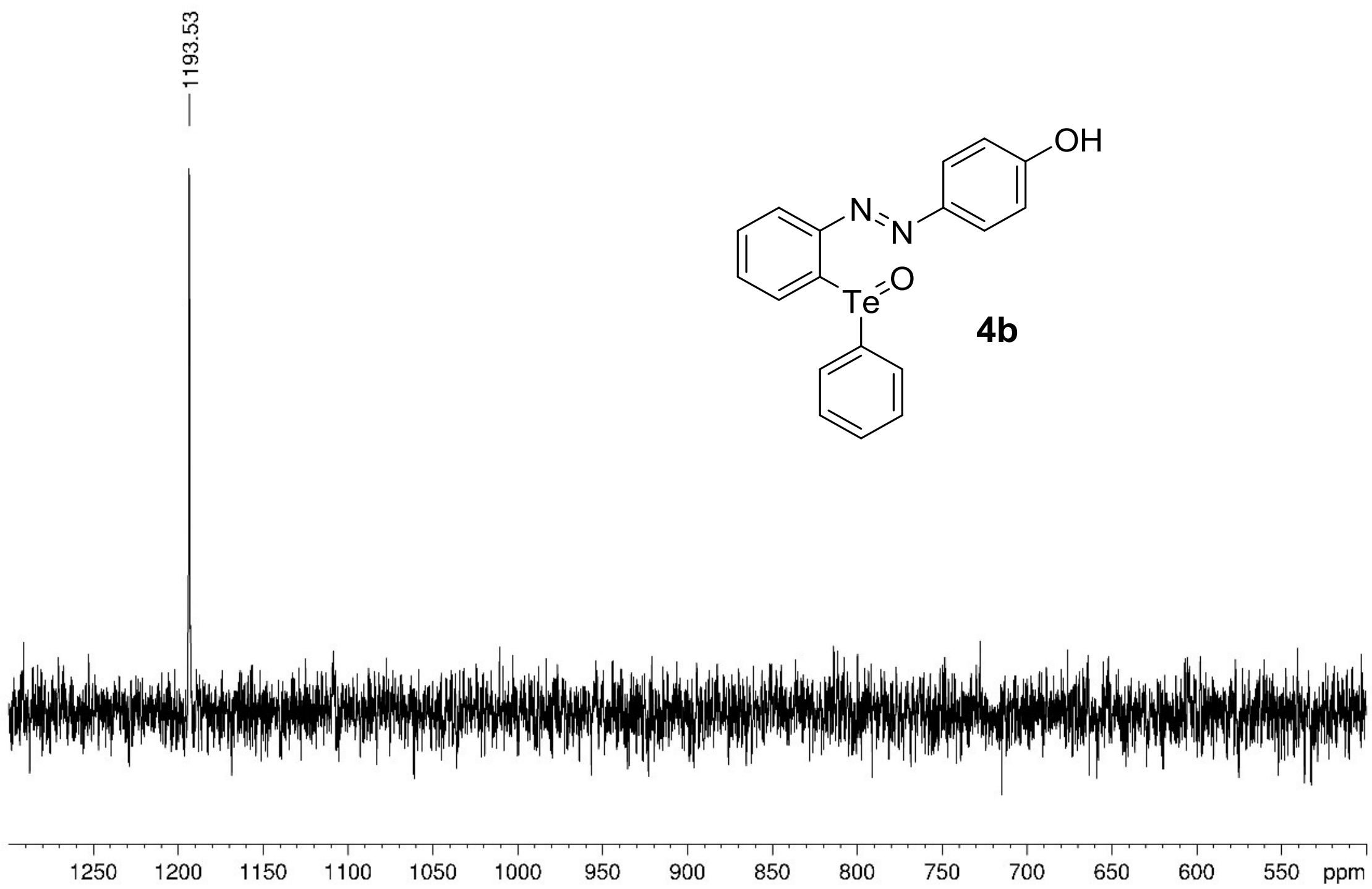
1193.53
OH
N
N
Te
O
4b
1250
1200
1150
1100
1050
1000
950
900
850
800
750
700
650
600
550
ppm

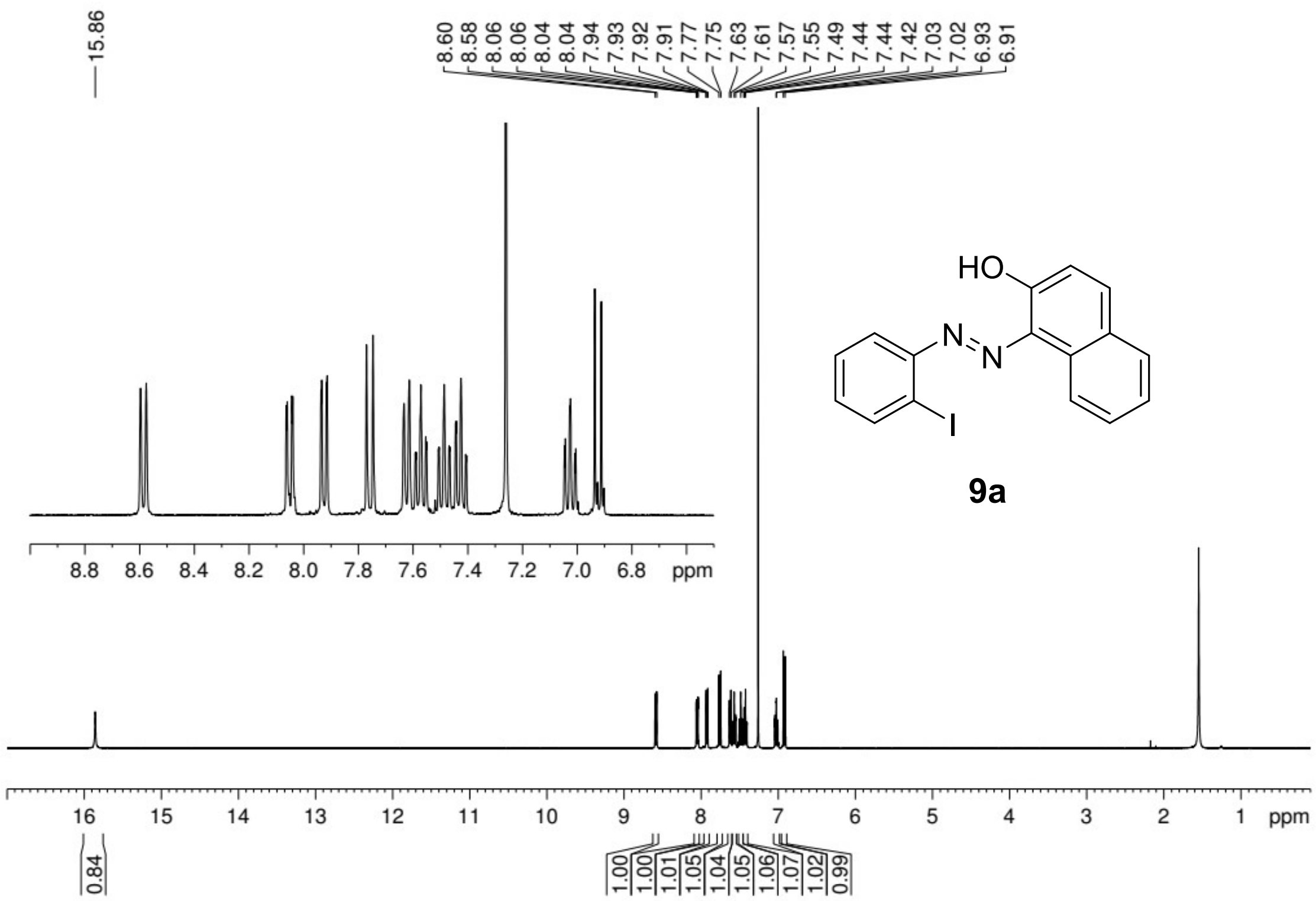
15.86
8.60
8.58
8.06
8.06
8.04
8.04
7.94
7.93
7.92
7.91
7.77
7.75
7.63
7.61
7.57
7.55
7.49
7.44
7.44
7.42
7.03
7.02
6.93
6.91
HO
N
N
I
ppm
0.84
1.00
1.00
1.01
1.05
1.04
1.05
1.06
1.07
1.02
0.99

9a

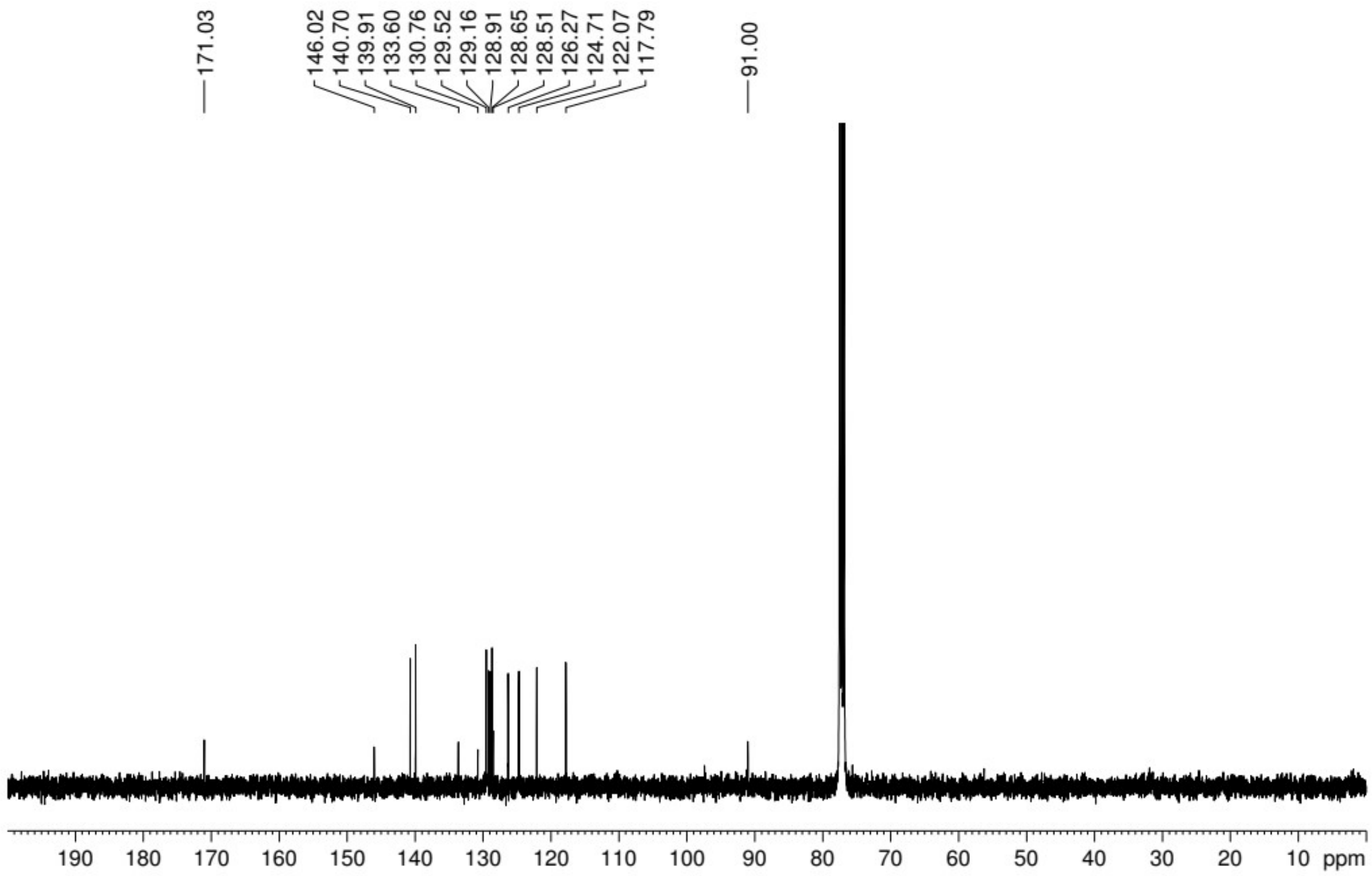
171.03
146.02
140.70
139.91
133.60
130.76
129.52
129.16
128.91
128.65
128.51
126.27
124.71
122.07
117.79
91.00
190 180 170 160 150 140 130 120 110 100 90 80 70 60 50 40 30 20 10 ppm

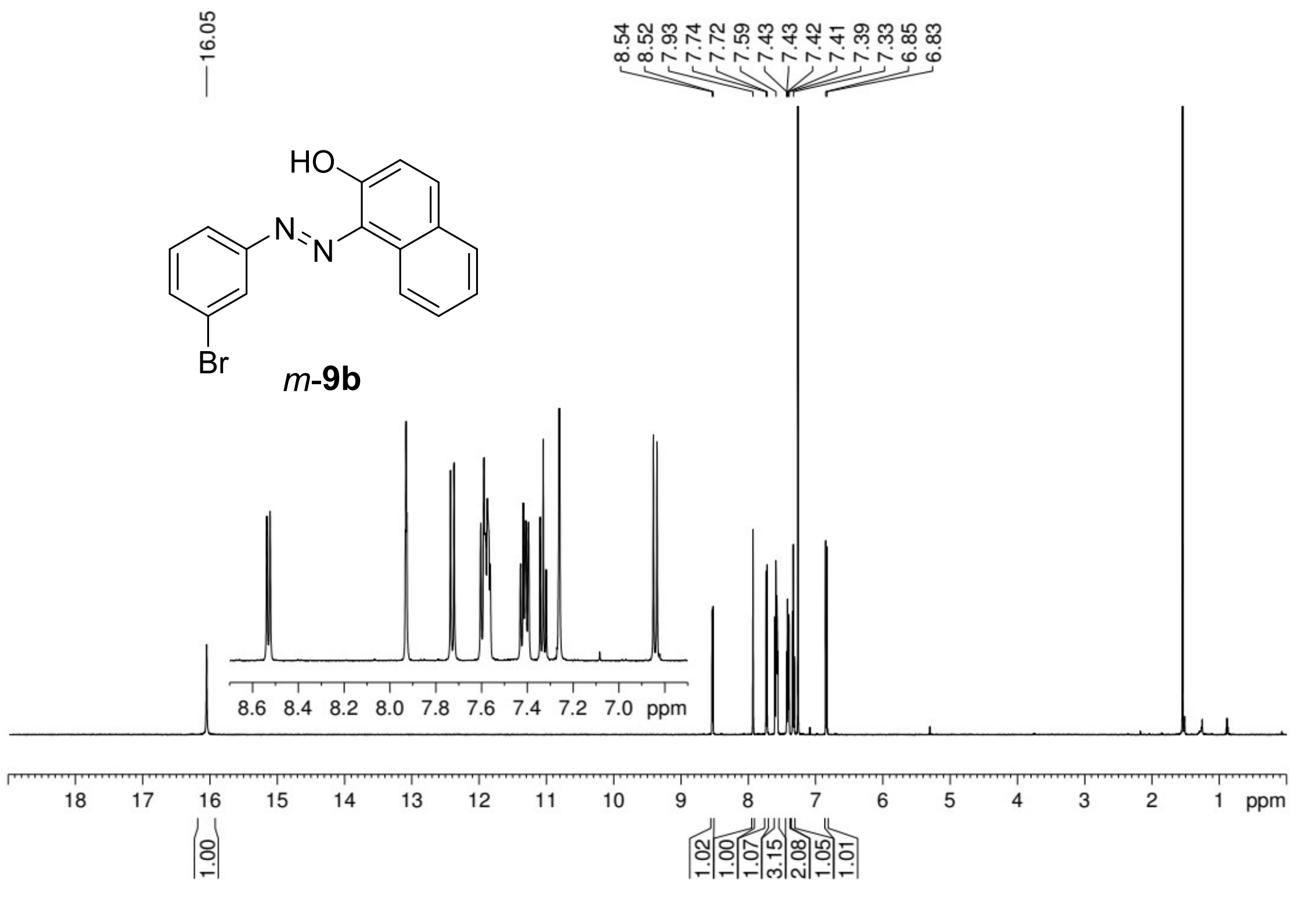
16.05
8.54
8.52
7.93
7.74
7.72
7.59
7.43
7.43
7.42
7.41
7.39
7.33
6.85
6.83
HO
N
N
Br
m-9b
8.6 8.4 8.2 8.0 7.8 7.6 7.4 7.2 7.0 ppm
18 17 16 15 14 13 12 11 10 9 8 7 6 5 4 3 2 1 ppm
1.00
1.02
1.00
1.07
3.15
2.08
1.05
1.01

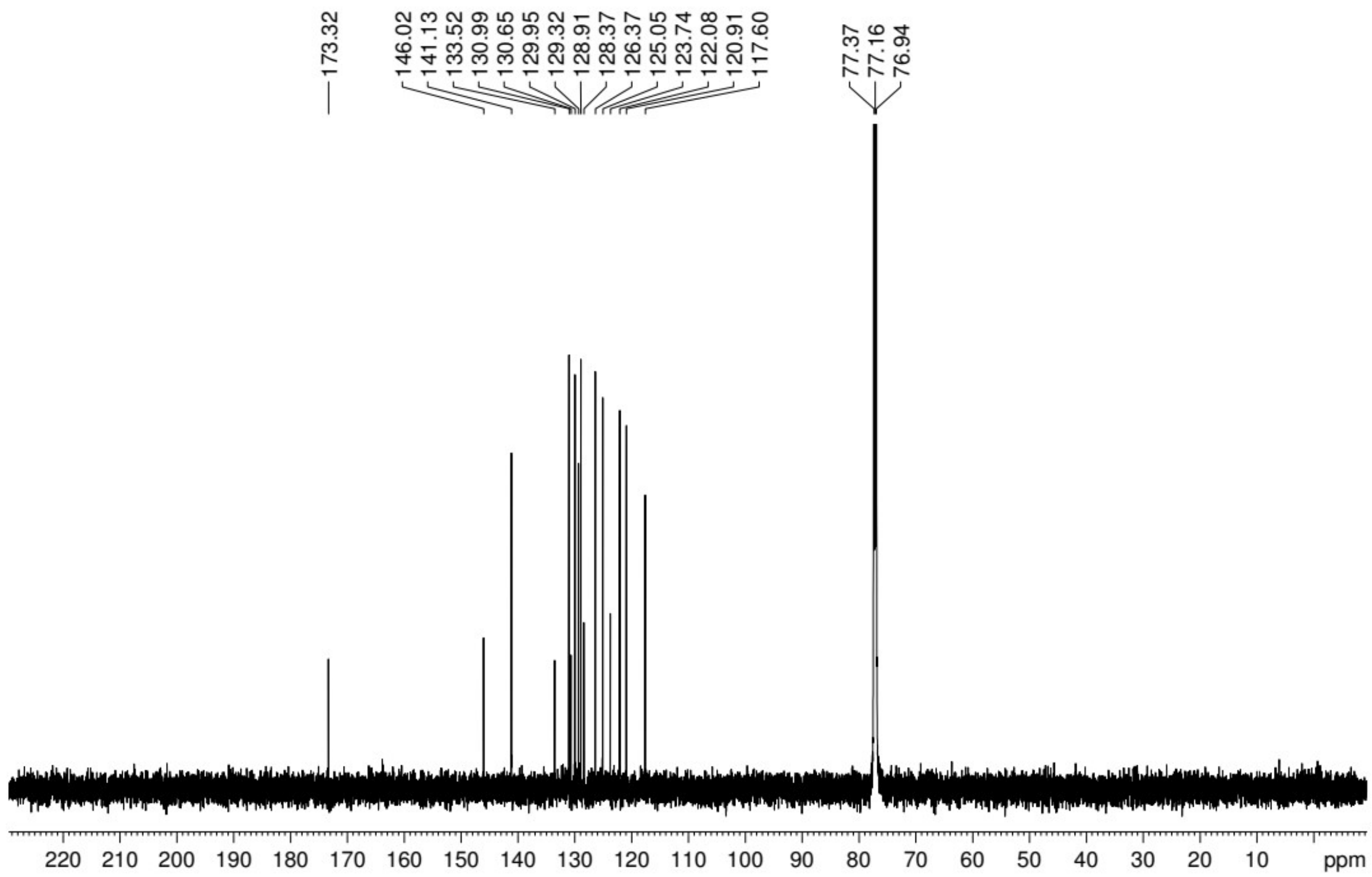

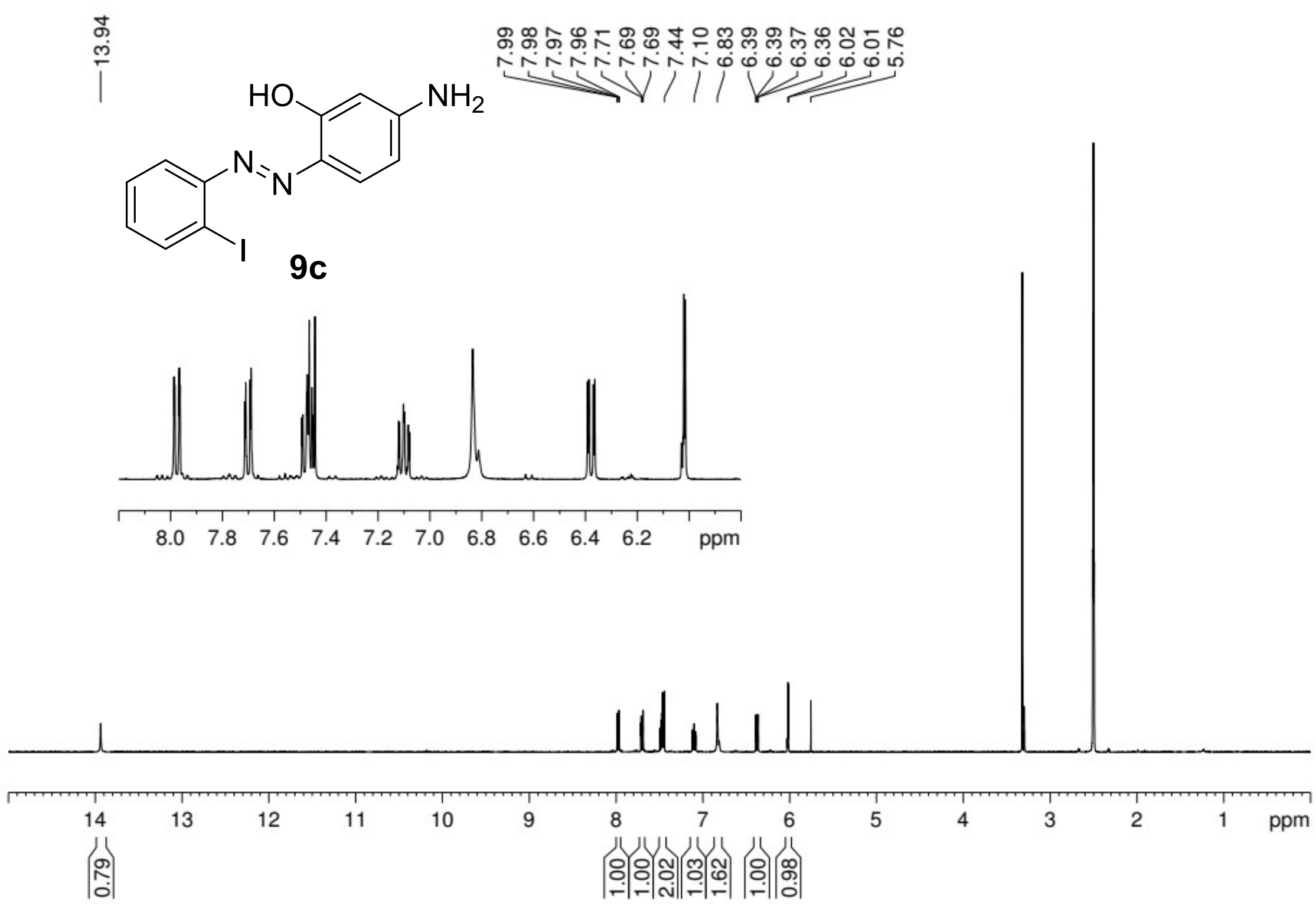

9c

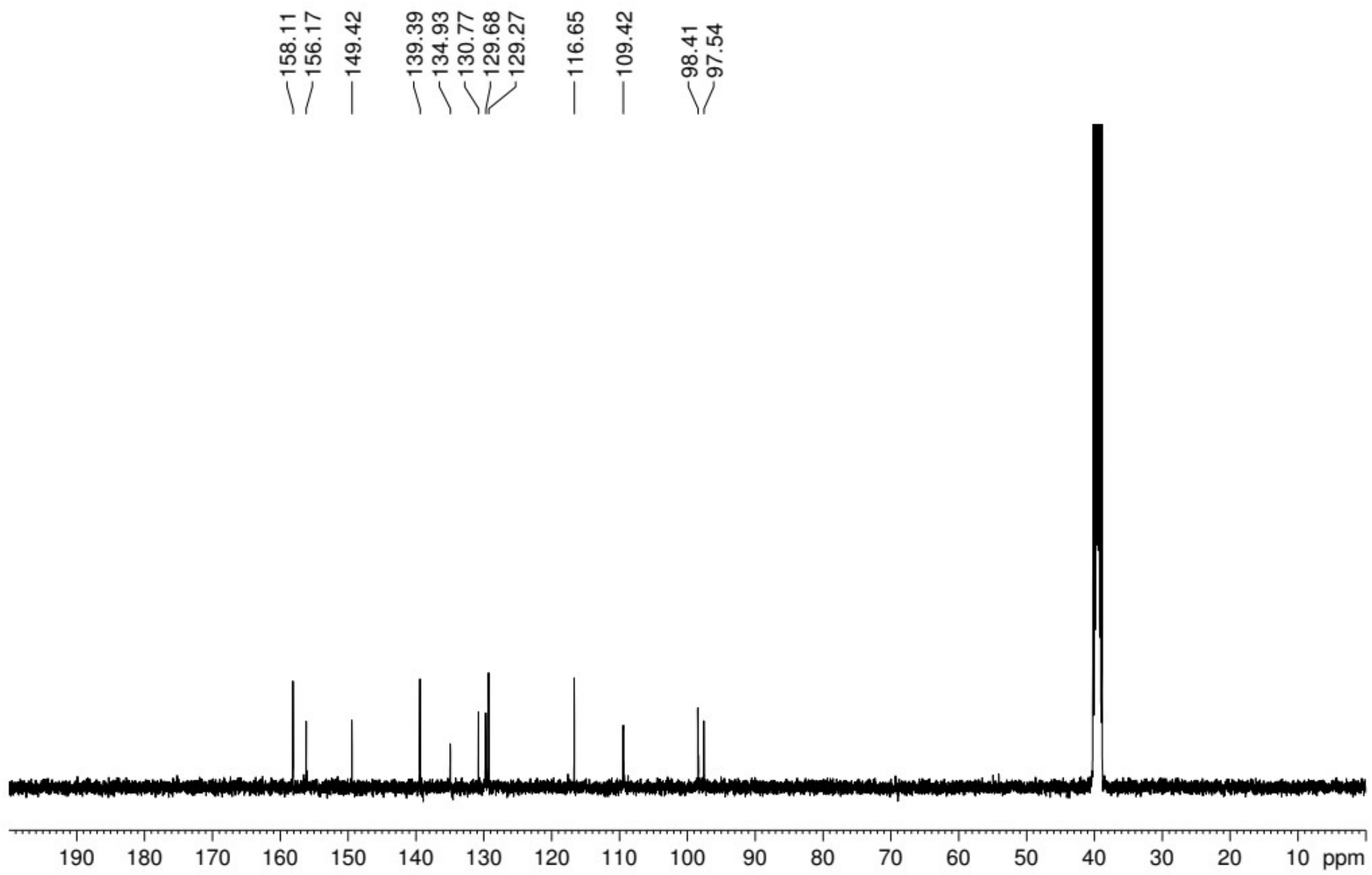
158.11
156.17
149.42
139.39
134.93
130.77
129.68
129.27
116.65
109.42
98.41
97.54
190 180 170 160 150 140 130 120 110 100 90 80 70 60 50 40 30 20 10 ppm

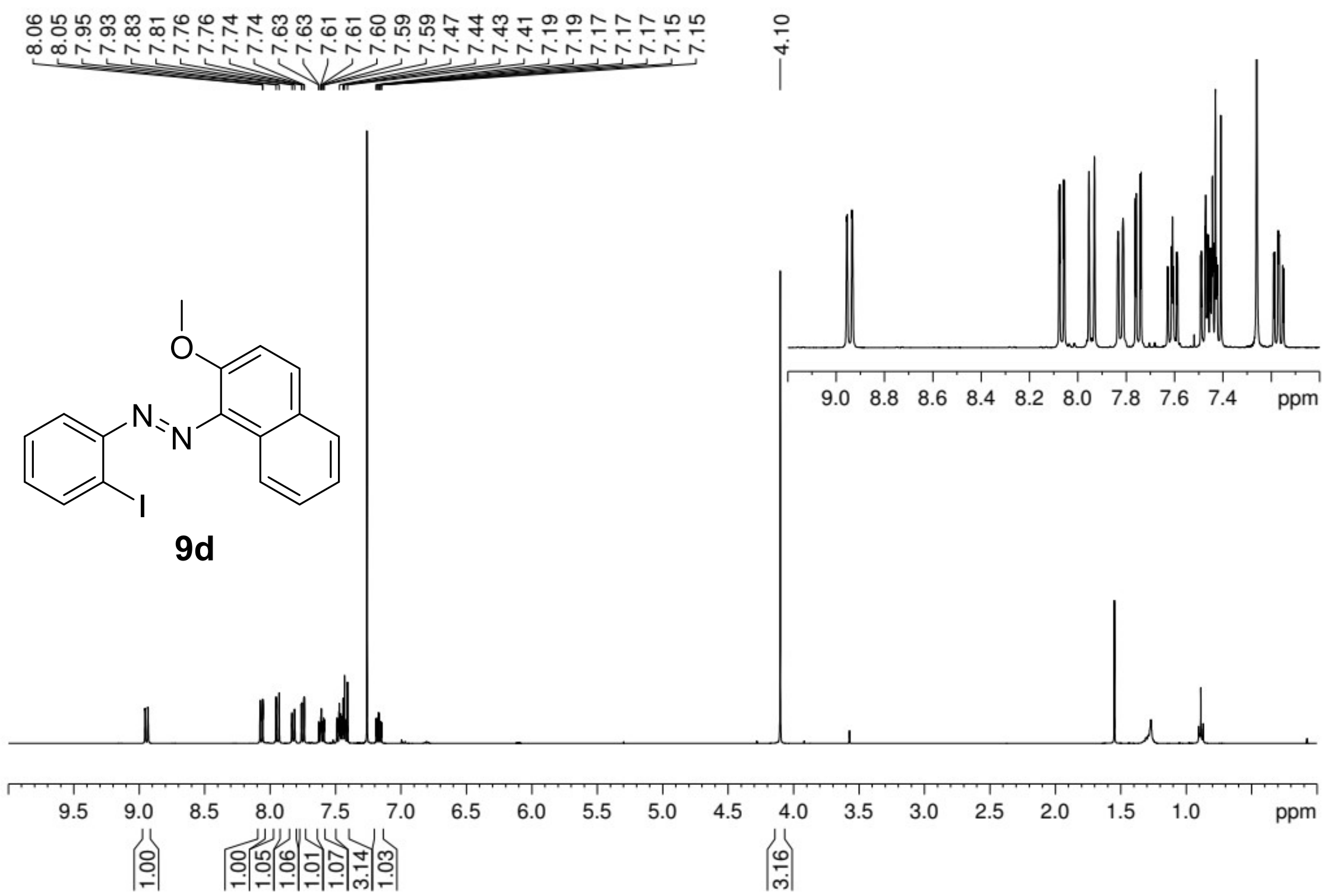
8.06
8.05
7.95
7.93
7.83
7.81
7.76
7.76
7.74
7.74
7.63
7.63
7.61
7.61
7.60
7.59
7.59
7.47
7.44
7.43
7.41
7.19
7.19
7.17
7.17
7.17
7.15
7.15
4.10
9.0 8.8 8.6 8.4 8.2 8.0 7.8 7.6 7.4 ppm
9.5 9.0 8.5 8.0 7.5 7.0 6.5 6.0 5.5 5.0 4.5 4.0 3.5 3.0 2.5 2.0 1.5 1.0 ppm
1.00
1.00
1.05
1.06
1.01
1.07
3.14
1.03
3.16
O
N
N
I

9d

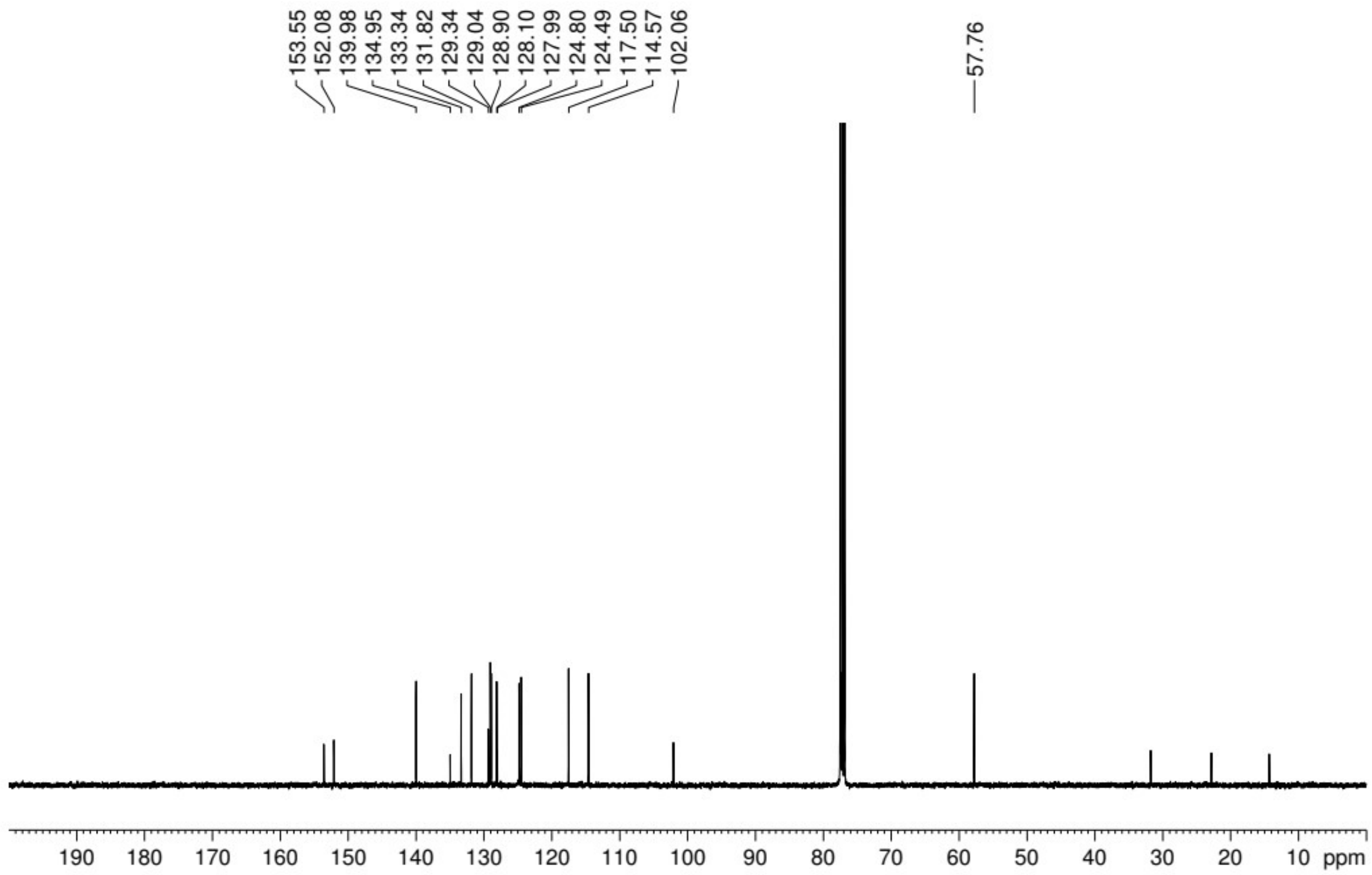

153.55
152.08
139.98
134.95
133.34
131.82
129.34
129.04
128.90
128.10
127.99
124.80
124.49
117.50
114.57
102.06
57.76
190
180
170
160
150
140
130
120
110
100
90
80
70
60
50
40
30
20
10 ppm

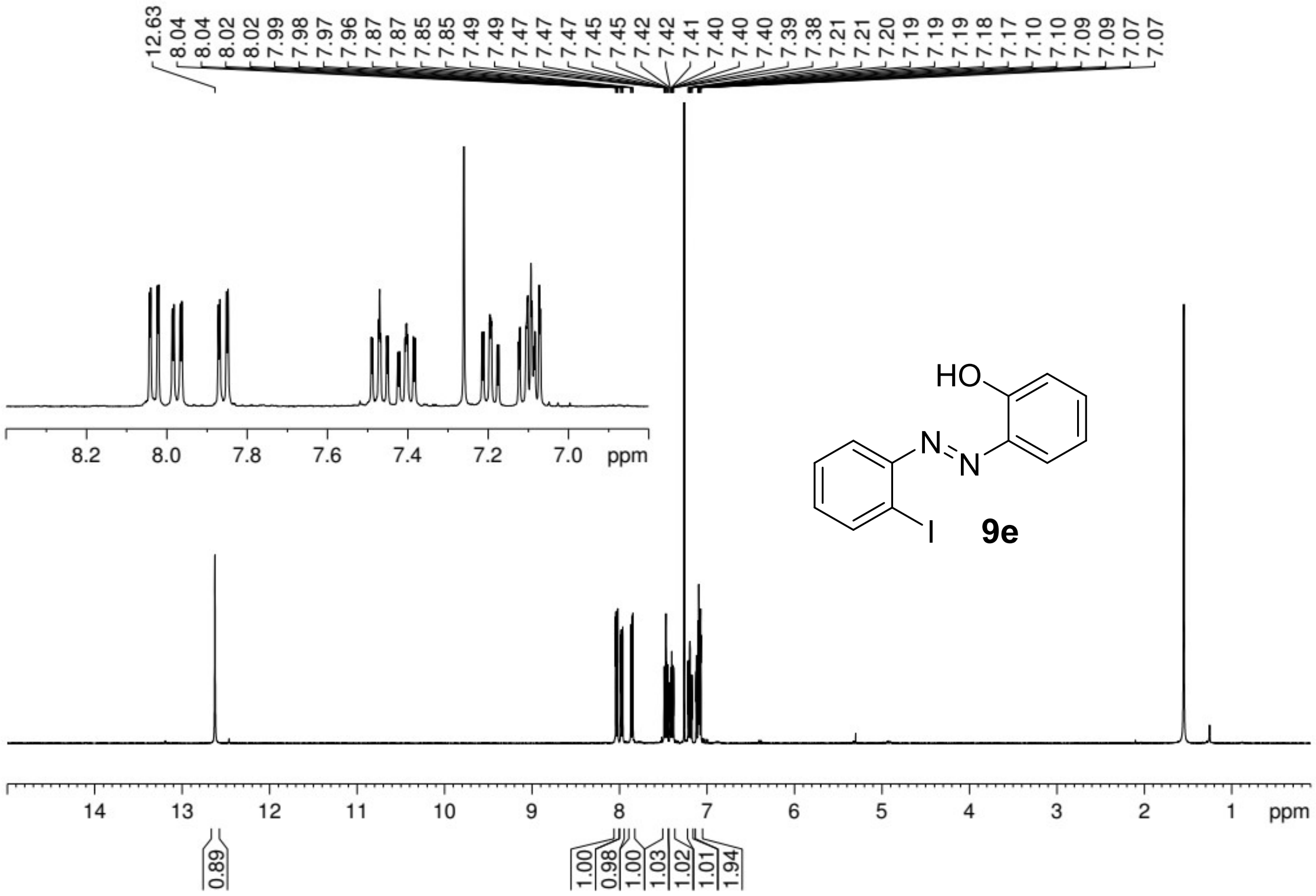
12.63
8.04 8.04 8.02 8.02 7.99 7.98 7.97 7.96 7.87 7.87 7.85 7.85 7.49 7.49 7.47 7.47 7.47 7.45 7.45 7.42 7.42 7.41 7.40 7.40 7.40 7.39 7.38 7.21 7.21 7.20 7.19 7.19 7.19 7.18 7.17 7.10 7.10 7.09 7.09 7.07 7.07
HO
N
N
I
9e
8.2 8.0 7.8 7.6 7.4 7.2 7.0 ppm
14 13 12 11 10 9 8 7 6 5 4 3 2 1 ppm
0.89
1.00 0.98 1.00 1.03 1.02 1.01 1.94

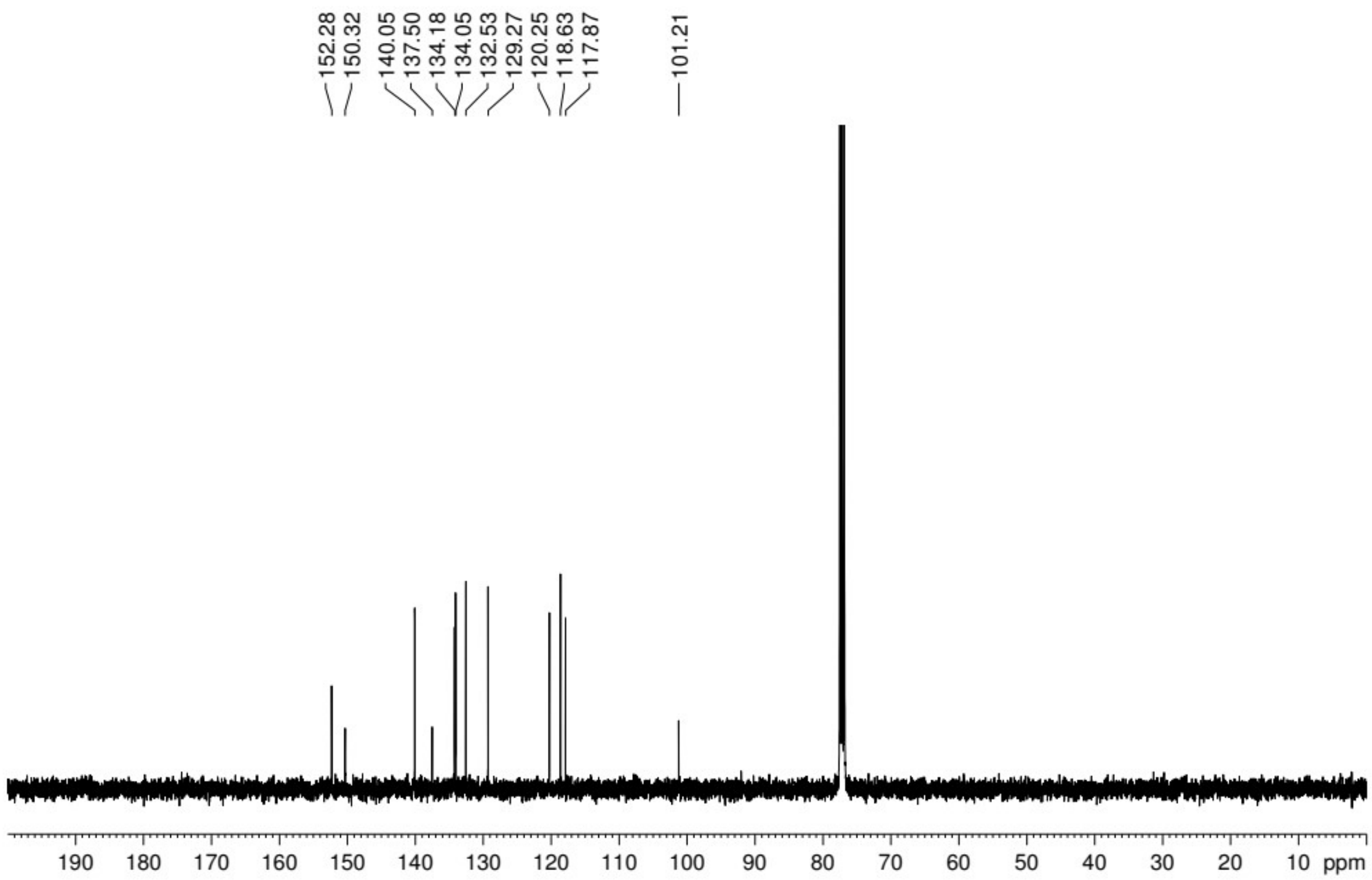
152.28 150.32 140.05 137.50 134.18 134.05 132.53 129.27 120.25 118.63 117.87 101.21
190 180 170 160 150 140 130 120 110 100 90 80 70 60 50 40 30 20 10 ppm

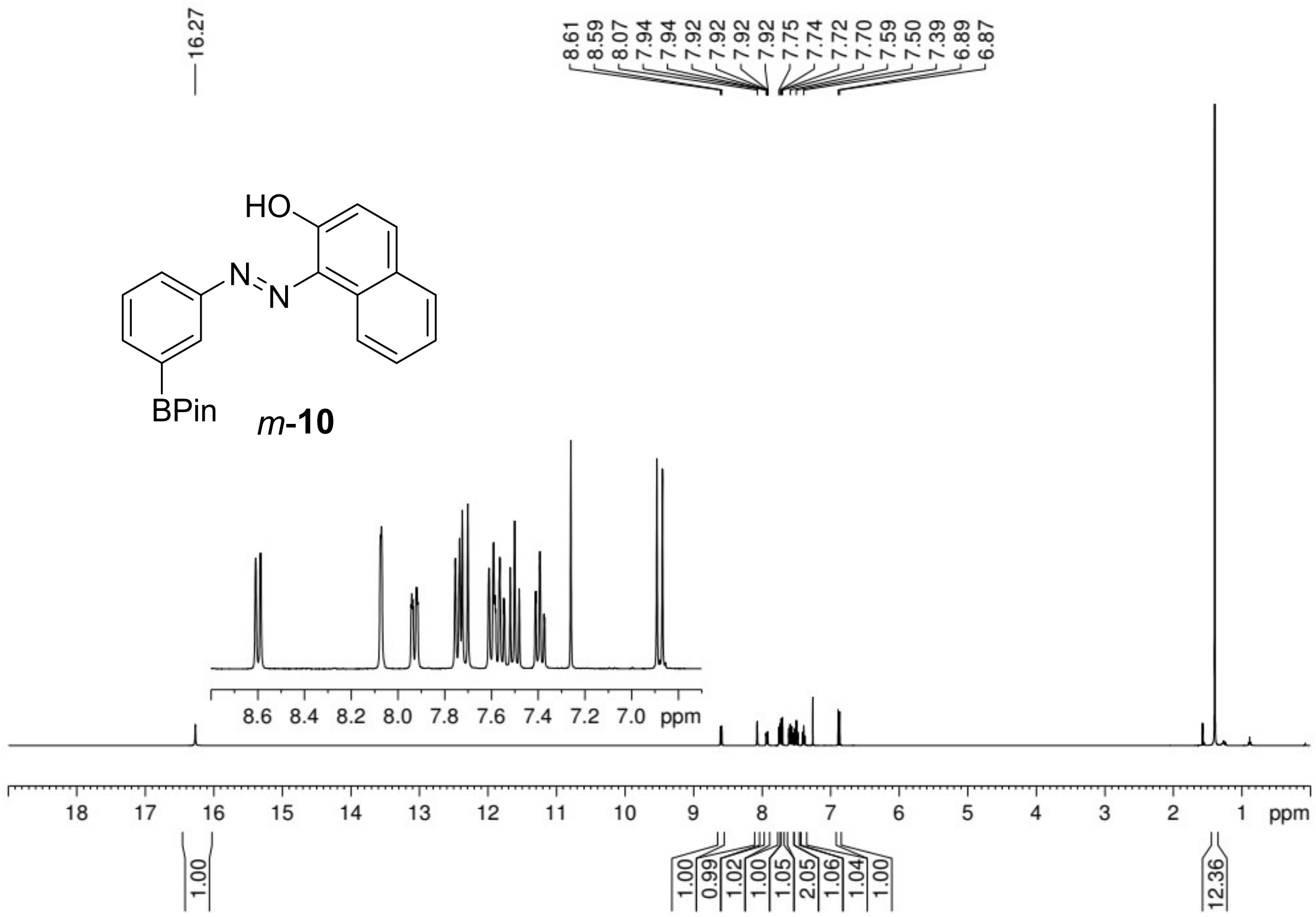

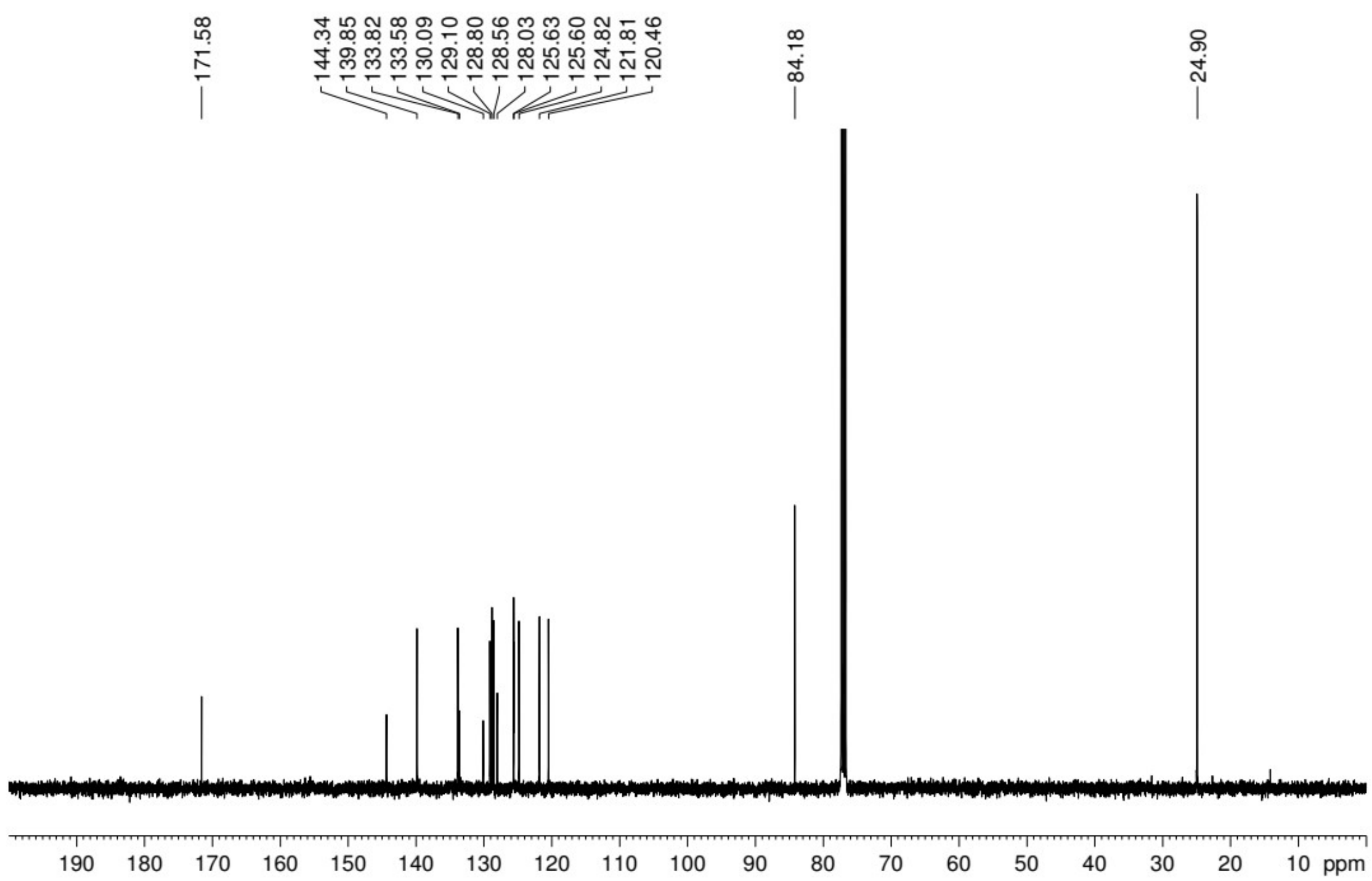

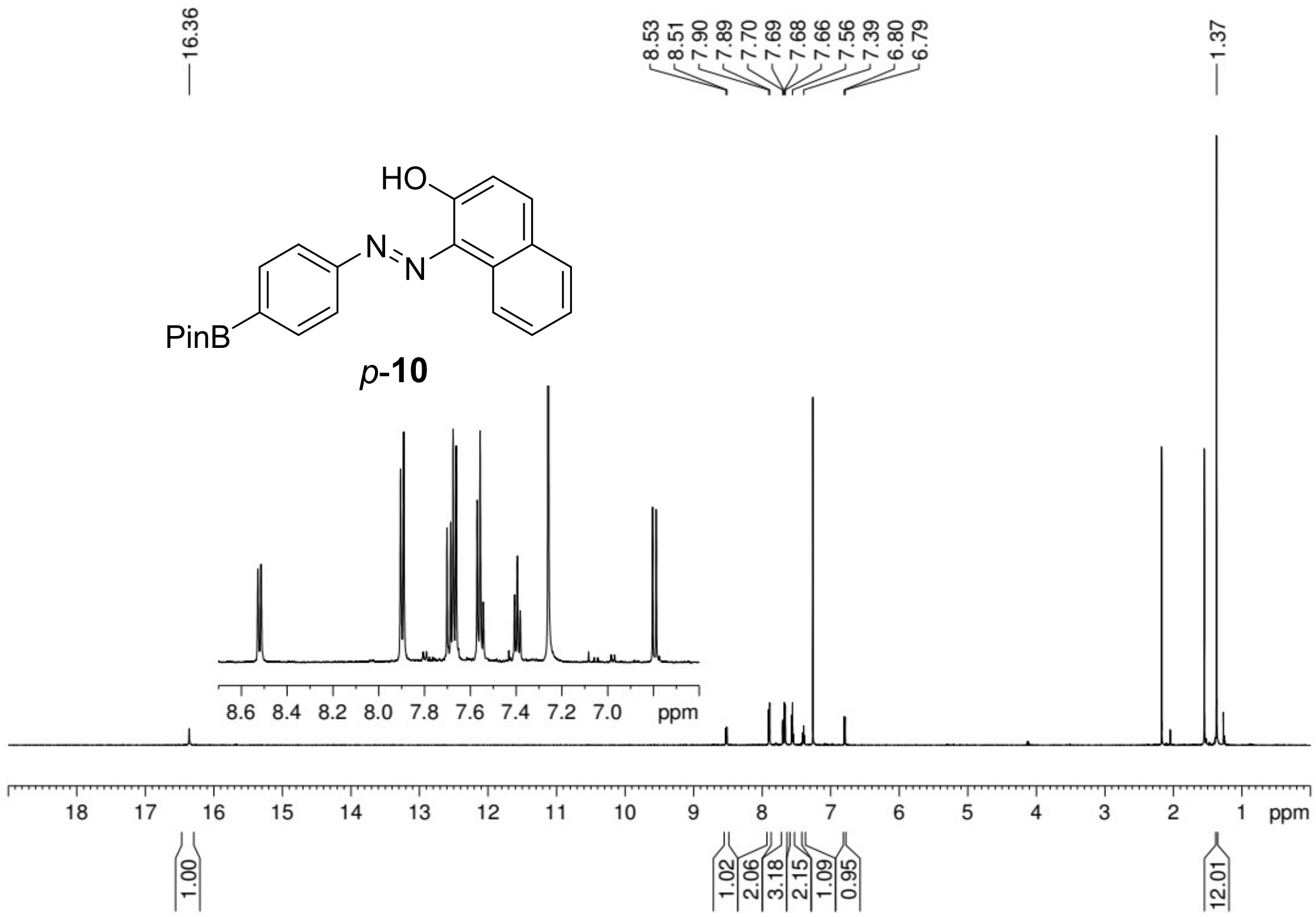
16.36
8.53
8.51
7.90
7.89
7.70
7.69
7.68
7.66
7.56
7.39
6.80
6.79
1.37
HO
N
N
PinB
p-10
8.6 8.4 8.2 8.0 7.8 7.6 7.4 7.2 7.0 ppm
18 17 16 15 14 13 12 11 10 9 8 7 6 5 4 3 2 1 ppm
1.00
1.02
2.06
3.18
2.15
1.09
0.95
12.01

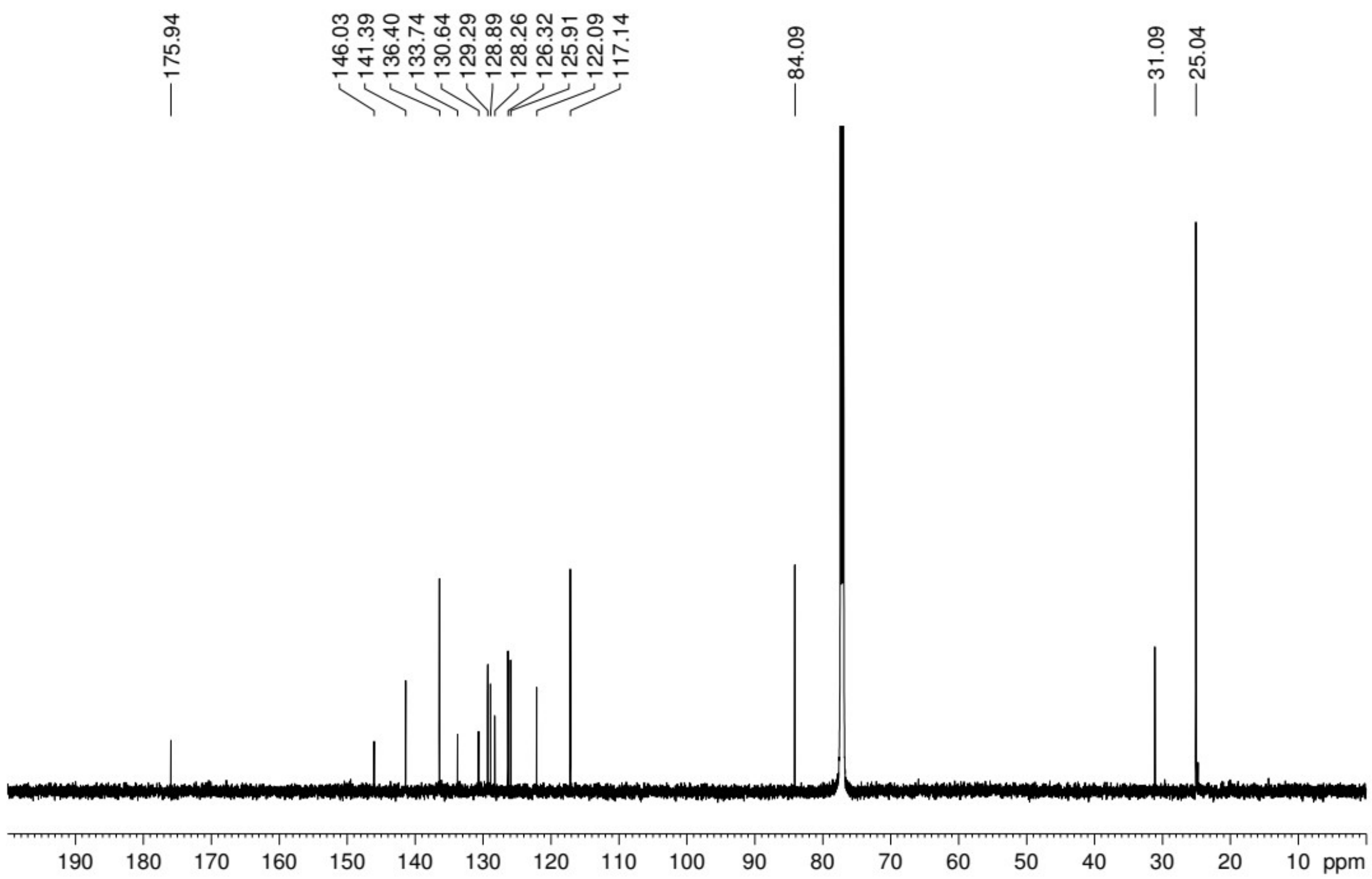
175.94
146.03
141.39
136.40
133.74
130.64
129.29
128.89
128.26
126.32
125.91
122.09
117.14
84.09
31.09
25.04
190 180 170 160 150 140 130 120 110 100 90 80 70 60 50 40 30 20 10 ppm

8. Supporting Information References

1. G. S. Shankarling, P. P. Deshmukh and A. R. Joglekar, *J. Environ. Chem. Eng.*, 2017, **5**, 3302-3308.
2. S. Steinwand, T. Halbritter, D. Rastädter, J. M. Ortiz-Sánchez, I. Burghardt, A. Heckel and J. Wachtveitl, *Chem. Eur. J.*, 2015, **21**, 15720-15731.
3. J. Yoshino, N. Kano and T. Kawashima, *Chem. Commun.*, 2007, DOI: 10.1039/B615966D, 559-561.
4. D. A. Pethsangave, R. V. Khose, A. C. Chaskar, S. C. Jun and S. Some, *ChemistrySelect*, 2016, **1**, 6933-6940.
5. R. M. Claramunt, D. Sanz, M. Pérez-Torralba, E. Pinilla, M. R. Torres and J. Elguero, *Eur J Org Chem*, 2004, DOI: 10.1002/ejoc.200400389, 4452-4466.
6. J. E. Kuder, *Tetrahedron*, 1972, **28**, 1973-1981.
7. M. J. Frisch, G. W. Trucks, H. B. Schlegel, G. E. Scuseria, M. A. Robb, J. R. Cheeseman, G. Scalmani, V. Barone, G. A. Petersson, H. Nakatsuji, X. Li, M. Caricato, A. V. Marenich, J. Bloino, B. G. Janesko, R. Gomperts, B. Mennucci, H. P. Hratchian, J. V. Ortiz, A. F. Izmaylov, J. L. Sonnenberg, D. Williams-Young, F. Ding, F. Lipparini, F. Egidi, J. Goings, B. Peng, A. Petrone, T. Henderson, D. Ranasinghe, V. G. Zakrzewski, J. Gao, N. Rega, G. Zheng, W. Liang, M. Hada, M. Ehara, K. Toyota, R. Fukuda, J. Hasegawa, M. Ishida, T. Nakajima, Y. Honda, O. Kitao, H. Nakai, T. Vreven, K. Throssell, J. A. Montgomery, Jr.; , J. E. Peralta, F. Ogliaro, M. J. Bearpark, J. J. Heyd, E. N. Brothers, K. N. Kudin, V. N. Staroverov, T. A. Keith, R. Kobayashi, J. Normand, K. Raghavachari, A. P. Rendell, J. C. Burant, S. S. Iyengar, J. Tomasi, M. Cossi, J. M. Millam, M. Klene, C. Adamo, R. Cammi, J. W. Ochterski, R. L. Martin, K. Morokuma, O. Farkas, J. B. Foresman and D. J. Fox, *Journal*, 2016.
8. C. Adamo and V. Barone, *J. Chem. Phys.*, 1999, **110**, 6158-6170.
9. S. Grimme, J. Antony, S. Ehrlich and H. Krieg, *J. Chem. Phys.*, 2010, **132**, 154104.
10. F. Weigend and R. Ahlrichs, *Phys. Chem. Chem. Phys.*, 2005, **7**, 3297-3305.
11. A. V. Marenich, C. J. Cramer and D. G. Truhlar, *J. Phys. Chem. B*, 2009, **113**, 6378-6396.
12. J. R. Cheeseman, G. W. Trucks, T. A. Keith and M. J. Frisch, *J. Chem. Phys.*, 1996, **104**, 5497-5509.
13. S. Parsons and H. Flack, *Acta Cryst. A*, 2004, **60**, S61.
14. S. Parsons, H. D. Flack and T. Wagner, *Acta Cryst. B*, 2013, **69**, 249-259.
